# Genome-scale reconstruction and *in silico* analysis of *Klebsiella oxytoca* for 2,3-butanediol production

**DOI:** 10.1186/1475-2859-12-20

**Published:** 2013-02-23

**Authors:** Jong Myoung Park, Hyohak Song, Hee Jong Lee, Doyoung Seung

**Affiliations:** 1 Research and Development Center, GS Caltex Corporation, 104-4 Munji-dong, Yuseong-gu, Daejeon, 305-380, Republic of Korea

## Abstract

**Background:**

*Klebsiella oxytoca*, a Gram-negative, rod-shaped, and facultative anaerobic bacterium, is one of the most promising 2,3-butanediol (2,3-BD) producers. In order to improve the metabolic performance of *K. oxytoca* as an efficient biofactory, it is necessary to assess its metabolic characteristics with a system-wide scope, and to optimize the metabolic pathways at a systems level. Provision of the complete genome sequence of *K. oxytoca* enabled the construction of genome-scale metabolic model of *K. oxytoca* and its *in silico* analyses.

**Results:**

The genome-scale metabolic model of *K. oxytoca* was constructed using the annotated genome with biochemical and physiological information. The stoichiometric model, KoxGSC1457, is composed of 1,457 reactions and 1,099 metabolites. The model was further refined by applying biomass composition equations and comparing *in silico* results with experimental data based on constraints-based flux analyses. Then, the model was applied to *in silico* analyses to understand the properties of *K. oxytoca* and also to improve its capabilities for 2,3-BD production according to genetic and environmental perturbations. Firstly, *in silico* analysis, which tested the effect of augmenting the metabolic flux pool of 2,3-BD precursors, elucidated that increasing the pyruvate pool is primarily important for 2,3-BD synthesis. Secondly, we performed *in silico* single gene knockout simulation for 2,3-BD overproduction, and investigated the changes of the *in silico* flux solution space of a *ldhA* gene knockout mutant in comparison with that of the wild-type strain. Finally, the KoxGSC1457 model was used to optimize the oxygen levels during fermentation for 2,3-BD production.

**Conclusions:**

The genome-scale metabolic model, KoxGSC1457, constructed in this study successfully investigated metabolic characteristics of *K. oxytoca* at systems level. The KoxGSC1457 model could be employed as an useful tool to analyze its metabolic capabilities, to predict its physiological responses according to environmental and genetic perturbations, and to design metabolic engineering strategies to improve its metabolic performance.

## Background

There is growing interest in the production of 2,3-butanediol (2,3-BD) by microbial fermentation, as it can be easily converted to methyl ethyl ketone and tetramethyl ether, blending agents for gasoline, and 1,3-butadiene, an intermediate in synthetic rubber manufacture [1-4]. *Klebsiella oxytoca* is known as one of the most promising 2,3-BD producers [1-10], and its whole genome sequences have been reported recently. The genome of *K. oxytoca* KCTC1686 consists of a chromosome of 5,974,109 bp with a 56.05% GC content, including 5,488 coding genes [11]. The genome of *K. oxytoca* E718 is composed of a chromosome of 6,097,032 bp and two plasmids of 324,906 bp and 110,781 bp with a 55.5% GC content, including 5,909 coding genes [12]. More recently, we isolated *K. oxytoca* KCTC12133BP from a cattle farm and sequenced its whole genome, but the sequence information, which has not yet been published, consists of a chromosome of 5,903,932 bp and a plasmid of 109,773 bp with a 55.4% GC content, including 5,793 coding genes.

The most important characteristics of *K. oxytoca* is to produce large amounts of C3/C4 diols, 1,3-propanediol (1,3-PD) and 2,3-BD, using various carbon sources [10,13-20]. For example, a lactate deficient mutant of *K*. *oxytoca* coproduced 83.6 and 60.1 g/L of 1,3-PD and 2,3-BD, respectively, in a fed-batch fermentation utilizing mixed substrates of glycerol and sucrose [13]. *K. oxytoca* also could produce more than 95 g/L of 2,3-BD from glucose with a yield of 0.478 g/g (95.6% of the theoretical maximum yield) and a productivity of 1.71 g/L/h in a batch fermentation, in which the agitation speed was switched from 300 to 200 rpm during the fermentation [10]. The availability of oxygen to *K. oxytoca* significantly affects its physiology and 2,3-BD production [21-23]. Another striking aspect of *K. oxytoca* is to readily metabolize glycerol, an inevitable by-product of biodiesel production, into biomass and products of value [13,24,25].

The aforementioned advantages make *K*. *oxytoca* an attractive host for industrial applications. Furthermore, the U.S. National Institute of Health (NIH, Guidelines for Research Involving Recombinant DNA Molecules, 2002) has reported that *K. oxytoca* belongs to risk group 1 (RG 1), recognizing it as a GRAS (Generally Regarded As Safe) organism. However, in order to use this organism on an industrial scale, the strain should be further developed. Systems metabolic engineering allows the rational design of metabolic networks for the overproduction of target compounds and the creation of industrially useful microorganisms [26-31]. Here, and *in silico* genome-scale metabolic model of *K. oxytoca*, KoxGSC1457, was constructed based on genome information, databases, and experimental data. The KoxGSC1457 model is composed of 1,457 reactions and 1,099 metabolites (Additional file 1 and Additional file 2). The model was carefully examined by *in silico* analyses for genetic and environmental perturbations. The *in silico* analysis using the model predicted that the pyruvate pool is mostly important for 2,3-BD synthesis, and this was verified by fermentation of the *ldhA* gene knockout mutant. Also, the model showed that the availability of oxygen strongly affected the production of 2,3-BD by *K*. *oxytoca*.

## Results and discussion

### Genome-scale reconstruction and general features of *K. oxytoca* metabolic network

**Figure 1:**
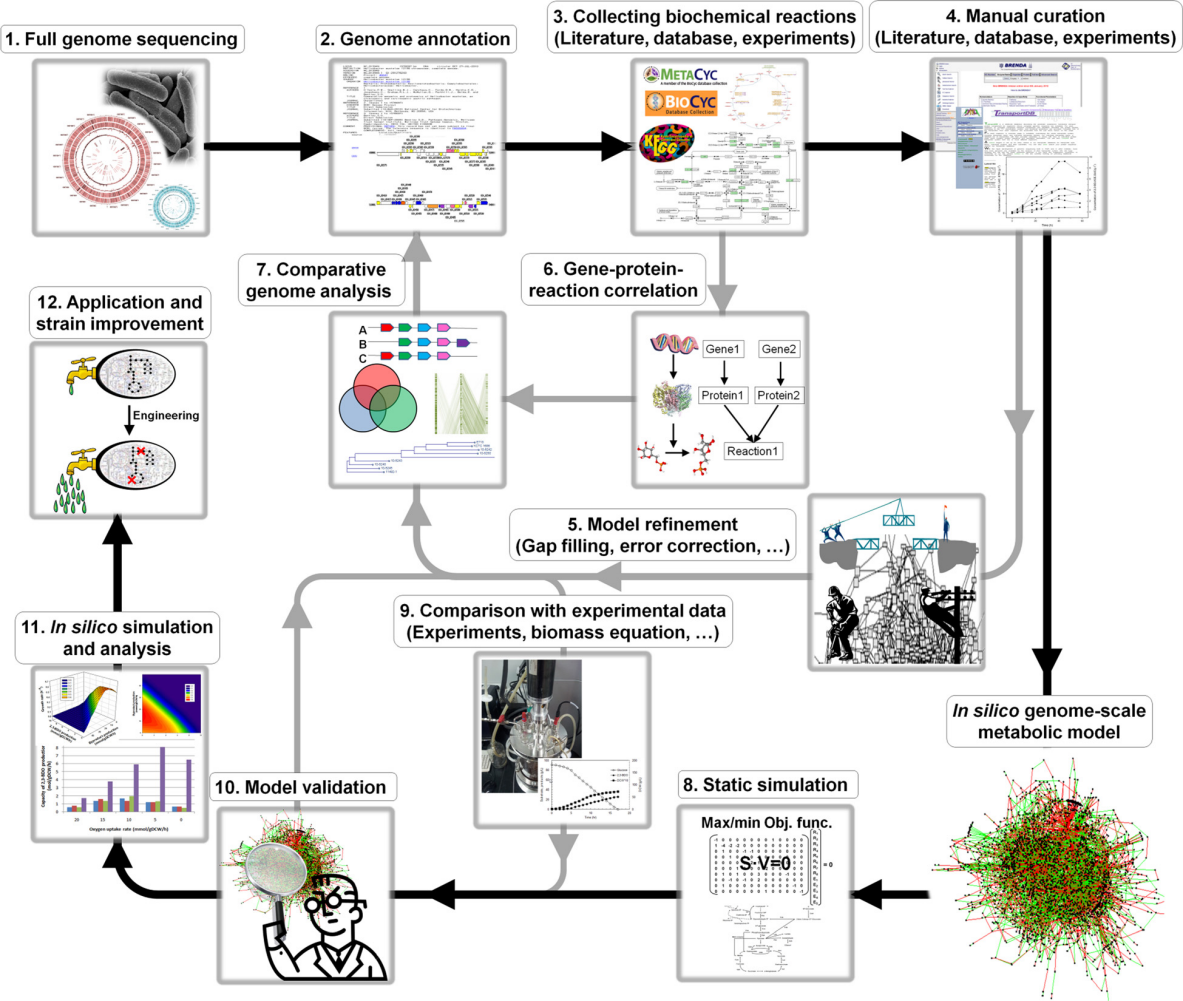
**Schematic processes of genome-scale metabolic model reconstruction for *K. oxytoca.*** (**1**-**2**-**3**) Reconstruction of metabolic network using genome annotation data. (**3**-**6**-**7**-**2** or **4**-**5**-**7**-**2**) Manual curation and model refinement of metabolic network based on literatures, databases, gene-protein-reaction correlation, comparative genome analysis, error corrections, and gap filling. (**8**-**9**-**10**) Model validation by comparing with experimental data and determination of biomass composition to be applied to the model. (**11**–**12**) Strain improvement and metabolic engineering by combining with *in silico* simulation and analysis. The thick and black arrows indicate the main process for model reconstruction. The thin and gray arrows indicated the process of model refinement and improvement.

**Figure 2:**
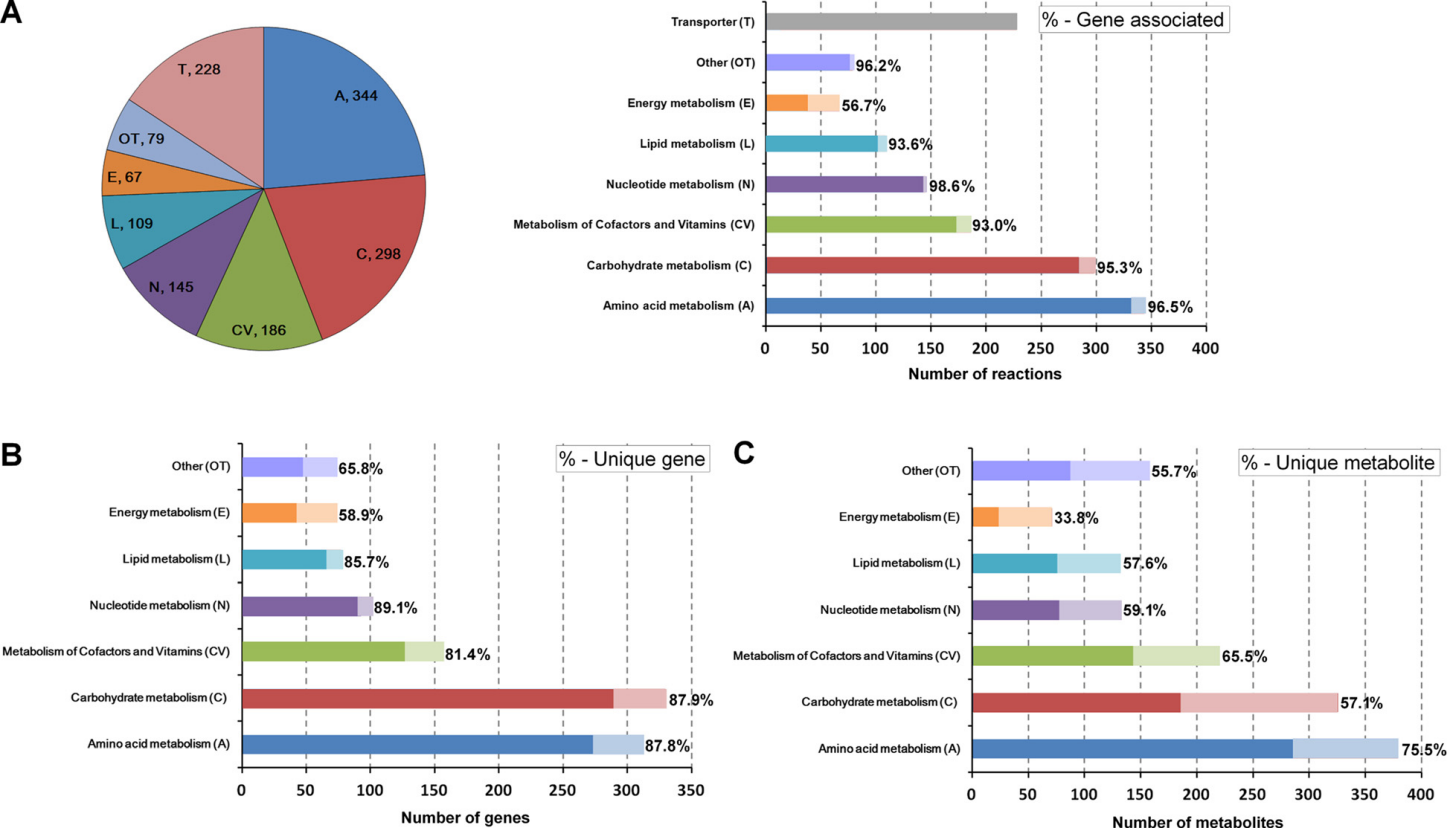
**Metabolic network properties of KoxGSC1457.** (**A**) The number of reactions in each of 8 functional categories. The percentages mean the ratio of reactions assigned with genes. (**B**) The number of genes in each category. The percentages mean the ratio of genes unique to each category. (**C**) The number of metabolites in each category. The percentages mean the ratio of metabolites unique to each category.

The metabolic network of *K. oxytoca* was initially reconstructed based on genome annotation information and metabolic pathway databases [32,33]. The reconstructed metabolic network was further refined by comparing it with experimental data and incorporating biomass formation reactions. The composition of biomass components, including carbohydrates, amino acids, and lipids, was experimentally determined using cells collected in the mid-exponential growth phase (Additional file 1 and Additional file 3). After validation of the genome-scale metabolic model of *K. oxytoca* (KoxGSC1457) was completed, 1,457 metabolic reactions and 1,099 metabolites were included in the final construction (Figure 1 and 2, Table 1 and Additional file 1 and Additional file 2). The KoxGSC1457 model consists of 1,228 intracellular metabolic reactions and 229 transport reactions. 19.57% of the total open reading frames (ORFs), corresponding to 1,074 genes of 5,488 ORFs, was incorporated into the model (Table 1).

**Table 1:** **Features of the *in silico *genome-scale metabolic model of *K. oxytoca***

**Features**	**Number**
Genome feature	
Genome size (base pairs, bp)	
Chromosome 1	5,974,109 (56.05)^a^
No. of open reading frames (ORFs)	
Chromosome 1	5,488
*In silico* metabolic model	
No. of reactions (redundant) included in the model	1,457
No. of biochemical reactions	1,228
No. of transport reactions	229
No. of reactions (unique) included in the model	1,437
No. of metabolites	1,099
No. of ORFs assigned in metabolic network	1,074
ORF coverage^b^ (%)	19.57

^a^The parenthesis includes the value of G + C content (%).

^b^The number of ORFs participated in the KoxGSC1457 model divided by the total number of ORFs in the *K. oxytoca* genome.

The metabolic reactions in KoxGSC1457 were classified into 8 different subsystems, comprising 97 sub-metabolisms: amino acid metabolism, carbohydrate metabolism, metabolism of cofactors and vitamins, nucleotide metabolism, lipid metabolism, energy metabolism, transporters, and others (Figure 2 and Additional file 1). Figure 2A details the numbers and percentages of reactions associated with genes for each subsystems. Amino acid metabolism ranks as the largest subsystem in KoxGSC1457, followed by carbohydrate metabolism. The sum of the three largest subsystems, amino acid metabolism, carbohydrate metabolism, and metabolism of cofactors and vitamins, account for more than a half of the total number of reactions. The percentage of reactions assigned to ORFs in KoxGSC1457 was 79.7%. The remaining 20.3% of reactions included in the model contained lumped multi-step reactions, spontaneous reactions, reactions added to fill missing links, or several transport reactions (Table 1). More than 90% of the reactions in 6 subsystems, except for transporters and energy metabolism are associated with genes (Figure 2A). Figure 2B and C describe the classification of genes and metabolites in KoxGSC1457. The smaller percentages of unique metabolites for each category compared with those of unique genes imply that several metabolites participate with reactions in different subsystems, while most genes function to a specific subsystem (Figure 2B).

The KoxGSC1457 model contains the reactions for 2,3-BD biosynthesis catalyzed by acetolactate synthase (E.C. 2.2.1.6), acetolactate decarboxylase (E.C. 4.1.1.5), and acetoin reductase (E.C. 1.1.1.4). The model also includes a spontaneous reaction that converts α-acetolactate into diacetyl (C_4_H_6_O_2_) and CO_2_ in the presence of oxygen. Diacetyl can then be catalyzed into acetoin (C_4_H_8_O_2_), which is the precursor of 2,3-BD, by diacetyl reductase (E.C. 1.1.1.303). *K. oxytoca* has its specialty for glycerol utilization by oxidative and reductive routes. Thus, the model encompasses the reactions for glycerol utilization, which are catalyzed by glycerol kinase (E.C. 2.7.1.30) and glycerol-3-phosphate dehydrogenase (E.C. 1.1.5.3) of the oxidative route, and NAD-dependent glycerol dehydrogenase (E.C. 1.1.1.6) and dihydroxyacetone kinase (E.C. 2.7.1.29) of the reductive route. Finally, the refined KoxGSC1457 model was used to understand the metabolic characteristics of *K oxytoca* and was applied to analyze the strategies for 2,3-BD production.

### Effects of increasing the metabolic flux pools of 2,3-BD precursors in *K. oxytoca*

**Figure 3:**
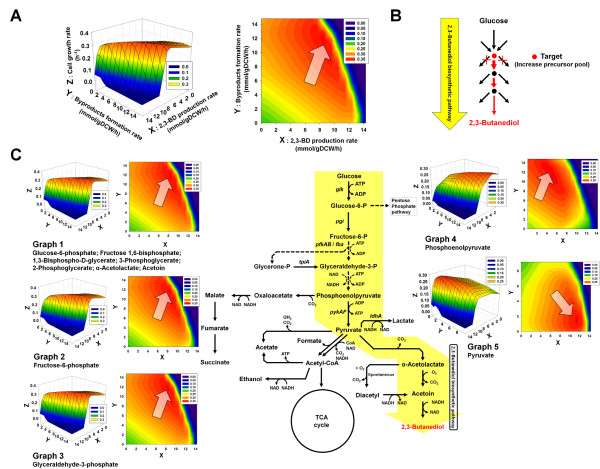
**Effects of increasing the metabolic flux pools of precursors for 2,3-BD production in *K. oxytoca*.** (**A**) 3D mesh plot graph as a continuous surface for *in silico* flux solution space of *K. oxytoca* wild-type strain correlated with cell growth rate, byproducts formation rate, and 2,3-BD production rate and its corresponding contour graph. (**B**) Method to examine the effects of increasing the metabolic flux pools of 2,3-BD precursors. In order to increase the metabolic flux pools of 2,3-BD precursors, all reactions that consume the target precursor were eliminated except for reactions that exist in 2,3-BD biosynthetic pathway and essential reactions for growth on the basis of the wild-type strain of KoxGSC1457. Because the first priority of a actual cell is survival, the maximal point of *in silico* cell growth rate is generally useful to predict the actual state of a cell, which is indicated by an arrow. Byproduct contains ethanol and several organic acids: lactic acid, acetic acid, succinic acid, and formic acid. (**C**) Schematic metabolic pathway related with 2,3-BD production and 3D mesh plot graphs as a continuous surface for *in silico* flux solution spaces of cases that increase each metabolic flux pool of 2,3-BD precursors and its corresponding contour graphs. The colors changed by gradation in graph indicate the value of cell growth rate. The red color means larger value of cell growth rate than other colors.

*K. oxytoca* is naturally capable of producing 2,3-BD by metabolizing various carbon sources such as lactose, galactose, glucose, xylose, and glycerol [9,10,13-16,18]. 2,3-BD is synthesized from pyruvate by enzymes encoded by the *budB*, *budA*, and *budC* genes in a operon [2,34]. Two pyruvates from glycolysis are converted to 2-acetolactate by releasing CO_2_ (*budB*). 2-acetolactate is then transformed into acetoin by decarboxylation to CO_2_ (*budA*). 2,3-BD is then synthesized from acetoin with oxidation of NADH into NAD^+^ by acetoin reductase (*budC*) (Figure 3). For the synthesis of one mole of 2,3-BD from pyruvate, two moles of CO_2_ are released and one mole of NADH is oxidized. The respective reactions for 2,3-BD synthesis are reflected in the KoxGSC1457 model. The model was then employed to design the strategy of 2,3-BD overproduction and to understand its metabolic characteristics.

**Figure 4:**
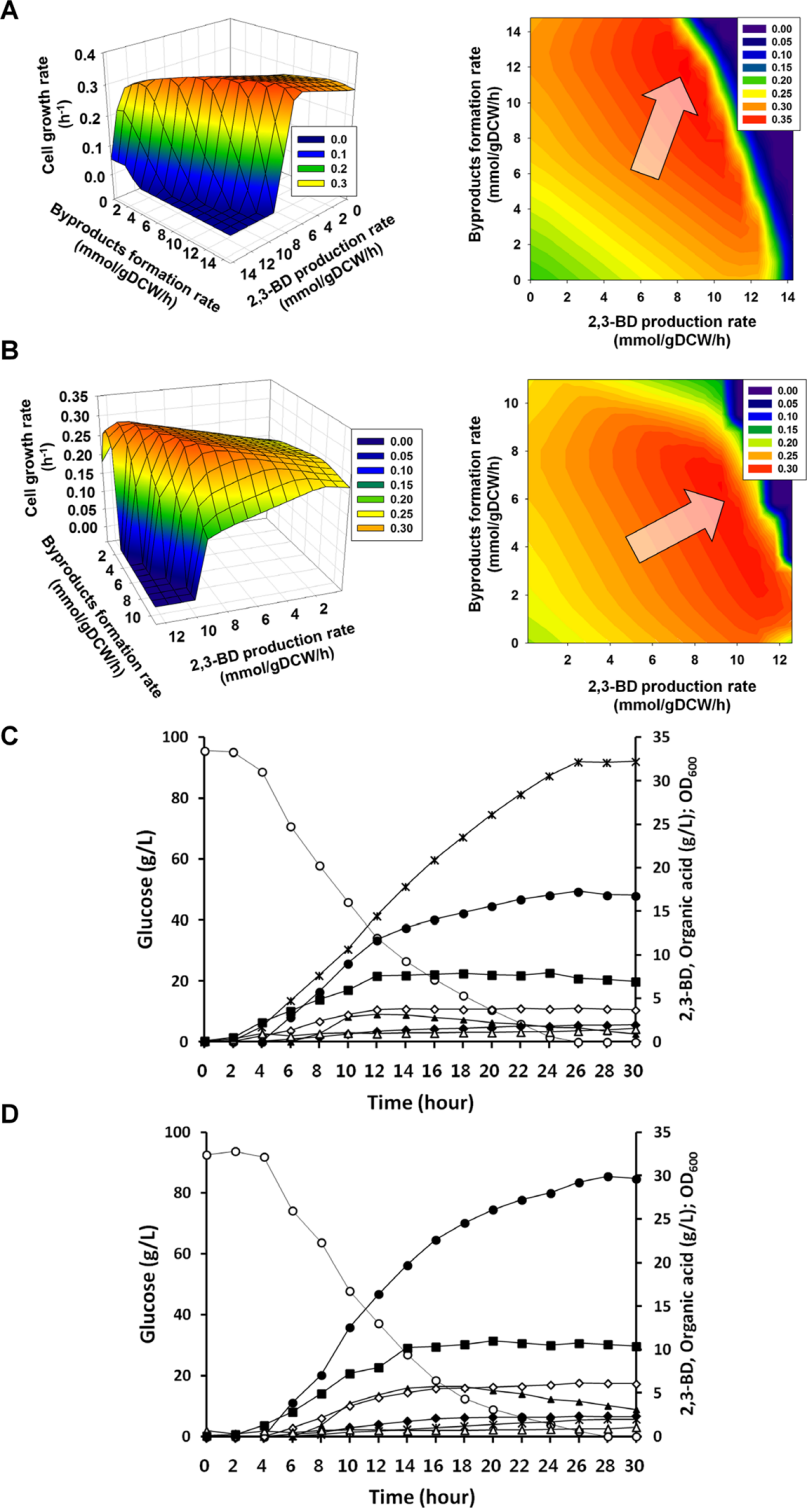
**Flux solution spaces and batch fermentation results of wild-type and mutant (*ΔldhA*) strains of *K. oxytoca*.** (**A**) 3D mesh plot graph as a continuous surface for *in silico* flux solution space of *K. oxytoca* wild-type strain correlated with cell growth rate, byproducts formation rate, and 2,3-BD production rate and its corresponding contour graph. (**B**) 3D mesh plot graph as a continuous surface for *in silico* flux solution space of *ldhA* gene knockout mutant of *K. oxytoca* correlated with cell growth rate, byproducts formation rate, and 2,3-BD production rate and its corresponding contour graph. The colors changed by gradation in graph indicate the value of cell growth rate. The red color means larger value of cell growth rate than other colors. (**C**) Batch fermentation result of *K. oxytoca* wild-type strain. (**D**) Batch fermentation result of *ldhA* gene knockout mutant of *K. oxytoca*. The arrow indicates the position of *in silico* optimal cell growth rate. Based on the flux solution space of wild-type strain, the large amount of byproducts were produced during 2,3-BD production. After *in silico* knockout of *ldhA* gene, byproducts formation rate was decreased, while 2,3-BD production rate was increased in comparison with wild-type strain. The fermentation was performed at 150 rpm, pH 6.5, and 37°C in 3 L working volume. The symbols in fermentation profiles indicates the concentration of glucose (○), 2,3-BD (●), OD_600_ (■), ethanol (▲), succinic acid (♦), lactic acid (*), formic acid (◊), and acetic acid (∆).

Firstly, the flux solution space of the wild-type strain of *K. oxytoca* correlated with cell growth, 2,3-BD production, and byproducts formation rates were analyzed in order to understand the current state of the wile-type by constraints-based flux analysis simulation using KoxGSC1457 (Figure 3A). As the first priority of cells is survival, the maximal point of an *in silico* cell growth rate is generally used to predict the actual state of a cell [35-37], which is indicated by an arrow in Figure 3. Byproducts include ethanol and several organic acids, such as lactic acid, acetic acid, succinic acid, and formic acid. The model predicted that the wild-type strain of *K. oxytoca* produces 2,3-BD along with large amounts of byproducts. This prediction was well supported by the previous studies that the wild-type strains yielded not only 2,3-BD but also lactic acid, formic acid, and ethanol as fermentation end-products (Figure 4C) [9,10,19]. Thus, genetic manipulations like gene knockout have been attempted to prevent the formation of byproducts in order to enhance 2,3-BD production [9,13].

Likewise, the KoxGSC1457 model was used to examine the importance of 2,3-BD precursors, and it gave better insights into augmenting the metabolic flux pools of 2,3-BD precursors for 2,3-BD overproduction (Figure 3B and C). In order to increase the metabolic flux pools of 2,3-BD precursors as well as to investigate its effects on 2,3-BD overproduction, all reactions participating in the consumption of target precursors were eliminated except for reactions involved in 2,3-BD biosynthetic pathways and essential reactions required for cell growth on the basis of the wild-type strain of KoxGSC1467, as described in Figure 3B. Accordingly, the flux solution spaces were scanned by increasing the flux pool of each precursor (Figure 3C). As a result, increasing the pools of most precursors had no effects on the production of 2,3-BD compared to that of the wild-type strain, as determined through comparison of the flux solution spaces. The model predicted that the surplus fluxes from artificially increased flux pools of most 2,3-BD precursors were not redirected to 2,3-BD synthesis but went toward other pathways for survival related with biomass synthesis, such as amino acids, nucleotides, lipids, and cofactors, prior to arriving at 2,3-BD biosynthetic reactions. In some cases, artificially increasing the flux pool of a 2,3-BD precursor (e.g. phosphoenolpyruvate) had negative influences on both cell growth and 2,3-BD synthesis by unbalancing the precursor requirements for biomass synthesis. However, if the pyruvate pool was augmented sufficiently, the flux solution space was altered so that KoxGSC1457 was enabled to overproduce 2,3-BD by lowering byproducts formation rates at an optimal cell growth rate (Figure 3C). The surplus of the pyruvate pool was sufficiently redirected to 2,3-BD synthesis, resulting in an approximate doubling of the 2,3-BD production rate. Correspondingly, byproducts formation rates decreased dramatically compared with those of the wild-type strain, and consequently, augmentation of the pyruvate pool is one of most important keys for 2,3-BD overproduction.

### *In silico* single gene knockout simulation and changes of flux solution space by *ldhA* gene knockout in *K. oxytoca*

During fermentation by the *K. oxytoca* wild-type strain, several byproducts, including lactic acid, ethanol, and formic acid were produced (Figure 4). For the overproduction of 2,3-BD in *K. oxytoca* by reducing the formation of byproducts and redirecting the remaining metabolic fluxes towards the synthesis of 2,3-BD, *in silico* single gene knockout simulation was performed using flux balance analysis (FBA). As a result, the knockout of *ldhA* gene encoding lactate dehydrogenase, which converts pyruvate into lactic acid with NADH oxidation, was targeted with top priority for a single gene knockout strategy based on the criteria of the maximization of 2,3-BD production rate and minimization of byproducts formation rates. Then, the KoxGSC1457 model investigated the metabolic characteristics of the *ldhA* gene knockout mutant by examining the changes of flux solution space compared with those of the wild-type strain (Figure 4A and B). In the wild-type strain, the model predicted that *K. oxytoca* produces large amounts of byproducts along with 2,3-BD. In the *ldhA* gene knockout mutant, the optimal point shifted to an increase of 2,3-BD production but to a decreased formation of byproducts, including lactic acid. *ldhA* gene knockout significantly increased the pyruvate pool that redirected the metabolic flux to 2,3-BD synthesis. This prediction was validated by batch fermentations of *K. oxytoca* wild-type and *ldhA* gene knockout strains (Figure 4C and D). *K. oxytoca* wild-type strain produced 32 g/L of lactic acid and 17 g/L of 2,3-BD by consuming 90 g/L of glucose. However, the *ldhA* gene knockout mutant dramatically decreased lactic acid production to 1.9 g/L, which is about 6% of that produced by the wild-type strain (Figure 4D). Accordingly, 2,3-BD production in the mutant was increased to about 30 g/L, which is about 176% of that produced by the wild-type strain. However, cell growth and glucose uptake rates of the *ldhA* gene knockout mutant were maintained in comparison with those of the wild-type strain because the redox balance of NAD^+^/NADH was not disrupted (Figure 4C and D). The deletion of lactate dehydrogenase forming lactic acid with the oxidation of NADH seemed to be compensated with 2,3-BD dehydrogenase, producing 2,3-BD with the oxidation of NADH as well, which made 2,3-BD production increase without any retardation of growth. When we compared the *in silico* flux solution space of the *ldhA* gene knockout mutant in Figure 4B with that of the increasing highly pyruvate pool in Figure 3C, more improvements for 2,3-BD overproduction could be made by further genetic manipulation. In particular, the fermentation results of the *ldhA* gene knockout mutant in Figure 4D suggests the construction of a mutant preventing the formation of ethanol, formic acid, and succinic acid. For this, the KoxGSC1457 model will be applicable to identify suitable candidates for further genetic manipulations.

### Effects of varying oxygen uptake rate for 2,3-BD production in *K. oxytoca*

**Figure 5:**
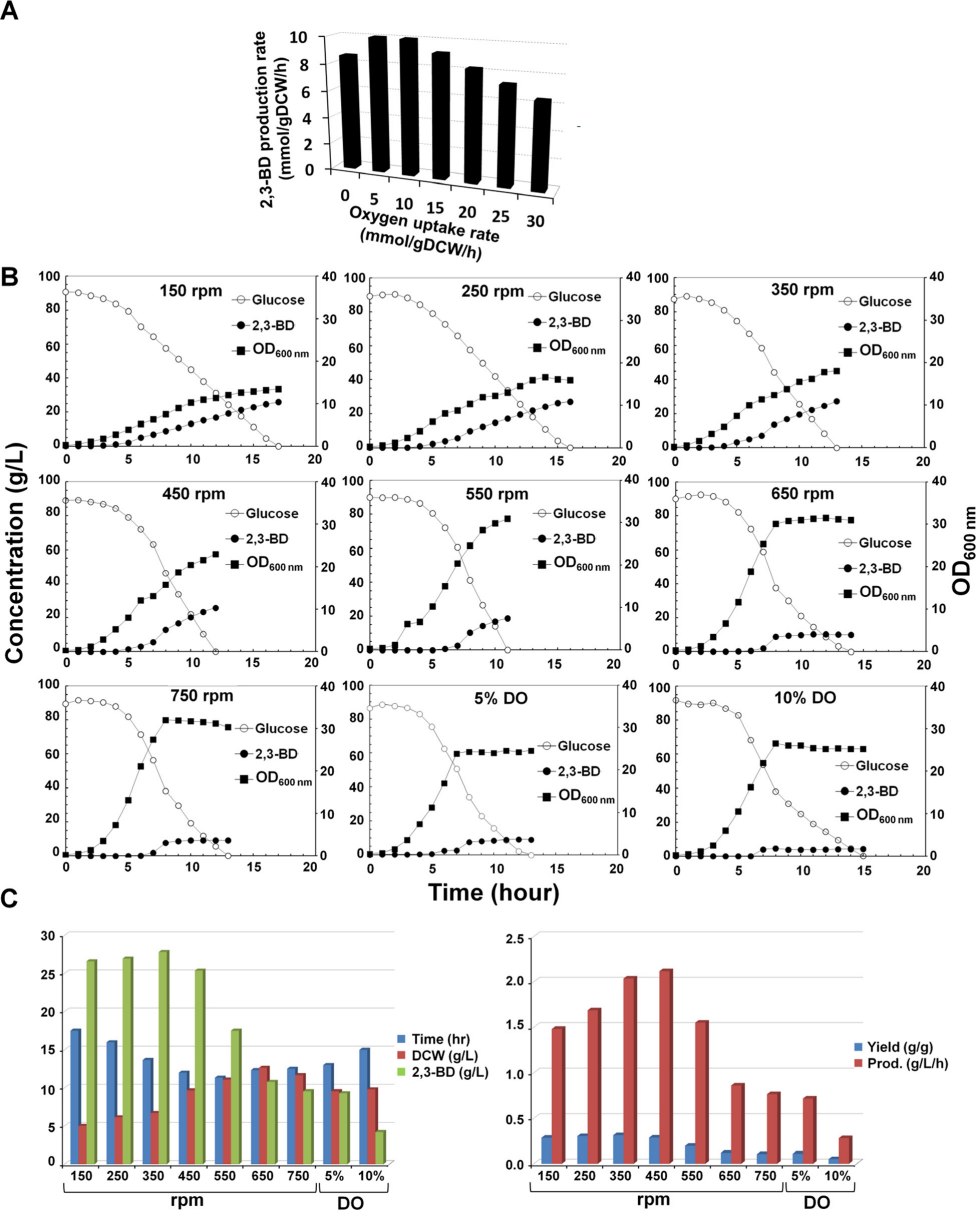
**Effects of varying oxygen uptake rate for 2,3-BD production.** (**A**) *In silico* prediction to examine the response of 2,3-BD production rate to varying oxygen uptake rate from 0 to 30 mmol/gDCW/h. (**B**) Fermentation profiles for different agitation speeds (150, 250, 350, 450, 550, 650, and 750 rpms) and DO levels (5% and 10%) in *ldhA* gene knockout mutant of *K. oxytoca*. The open circle (○), filled circle (●), and filled square (■) indicate the concentrations of glucose, 2,3-BD, and OD_600_, respectively. (**C**) Summary of fermentation results that include ending time of fermentation (hour), final DCW (g/L), 2,3-BD concentration (g/L), yield (g/g glucose), and productivity (g/L/h).

For 2,3-BD production by *K. oxytoca*, the dissolved oxygen (DO) level in medium is known as one of the most important environmental factors [21-23]. In order to gain an insight into the effect of oxygen uptake rate on 2,3-BD production in *K. oxytoca*, the oxygen uptake rate was perturbed by constraints-based flux analysis between 0 and 30 mmol/gDCW (gram dry cell weight)/h and glucose uptake rate was fixed to 10 mmol/gDCW/h, with maximization and minimization of 2,3-BD production rate as the objective function (Figure 5A). As shown in Figure 5A, the predicted 2,3-BD production rate increased as the oxygen uptake rate was raised to 5 and 10 mmol/gDCW/h. On the other hand, the 2,3-BD production rate gradually decreased with further increasing oxygen uptake rate (Figure 5A).

This simulation was validated by batch fermentation experiments under different agitation speeds (rpm) and DO levels (Figure 5B and C). The agitation speeds were a150, 250, 350, 450, 550, 650, and 750 rpm. DO levels were maintained at 5% and 10% by automatically adjusting the agitation speed between 50 and 1,000 rpm. The aeration rate was set at 1 vvm (air volume∙ working volume^-1^∙ minute^-1^) during the whole period of fermentation. The fermentations were performed by using the *ldhA* gene knockout mutant of *K. oxytoca*, as the most productive 2,3-BD producer up to this point. As a result, the final 2,3-BD concentration (g/L) and yield (g/g glucose) increased through increasing the agitation speed to 350 rpm, but further increasing agitation speed caused a decrease of the final 2,3-BD concentration and yield. Meanwhile, the maximal value of 2,3-BD productivity of 2.1 g/L/h was observed at 450 rpm. The highest glucose consumption was observed at 550 rpm, however, most consumption of carbon source was used to synthesize not 2,3-BD but biomass at 550 rpm and agitation speeds greater than 550 rpm. In addition, fermentation at 10% DO level showed less 2,3-BD concentration, yield, and productivity than at 5% DO. These results are a strong indication that excessive oxygen exposure during fermentation impairs 2,3-BD overproduction. Since the optimal oxygen level exists beyond the scope of DO as represented in Figure 5, it is recommended to adjust the agitation speed depending on cellular status for 2,3-BD overproduction. These observations were also supported by a previous study, in which the optimal agitation speed existed on 2,3-BD production [10].

## Conclusions

*K. oxytoca* is a promising microorganism which has great potential in the industrial production of 2,3-BD. To make the better use of this organism and to improve industrial applicability, we constructed a genome-scale metabolic model of *K. oxytoca,* KoxGSC1457, consisting of 1,457 reactions and 1,099 metabolites based on information obtained from genome annotation data, database, literature and validation experiments. Using the KoxGSC1457 model, *in silico* metabolic fluxes were analyzed to investigate the effects of environmental and genetic variation. First, the effects of increasing the metabolic flux pools of 2,3-BD precursors were examined to understand the most important precursor to be augmented for 2,3-BD overproduction. *In silico* single gene knockout simulation was then performed to identify gene knockout candidates for the reduction of byproduct formation, and the change of flux solution space was then analyzed by elimination of *ldhA* gene resulting in the *K. oxytoca ldhA* gene knockout mutant, which is one of organisms that shows best performance for 2,3-BD production so far. Finally, to design strategies for better 2,3-BD overproduction, *in silico* metabolic flux analyses of KoxGSC1457 were executed to examine the effects of varying oxygen uptake rate during 2,3-BD production, and to determine the optimal agitation speed. Through the successful use of the KoxGSC1457 genome-scale metabolic model like those, it gave us the confidence that the model was thoroughly validated in comparison with various experimental data. There are still many difficulties associated with the overall economics of producing 2,3-BD through *K. oxytoca* on an industrial scale. Hence, it is expected that the model constructed in this study will support us to understand cellular physiology on systems level, broaden our insight on this organism, and conquer problems more systematically.

## Methods

### Reconstruction of genome-scale metabolic model

Physiological data and biochemical reactions of *K. oxytoca* were collected from various sources, such as experiments, literatures, and public databases, in order to reconstruct the genome-scale metabolic model of *K. oxytoca*. First, information of genome annotation was gained from genome data in NCBI (http://www.ncbi.nlm.nih.gov/). Then, the biochemical reactions of *K. oxytoca* were assembled from various literatures, experiments, and public databases, including the Kyoto Encyclopedia of Genes and Genomes (KEGG, [32]), Biocyc [33], Metacyc [33], TCDB [38], and TransportDB [39]. Second, the draft version of the metabolic model was manually curated and refined based on experimentation, literature, databases, gene-protein-reaction correlations, comparative genome analysis, error corrections, and gap filling. Third, the refined model was validated through comparison with experimental data, including fermentation data. The biomass composition data of *K. oxytoca* were then analyzed and applied to the model. Finally, the validated model was used to analyze the physiology of *K. oxytoca* and to apply to *in silico* simulations for strain improvement and fermentation optimization (Figure 1).

### Biomass composition

The composition of biomass components was experimentally measured to construct the biomass formation reactions of *K. oxytoca* more accurately. The cells of *K. oxytoca* were cultured in minimal culture medium containing no yeast extract, and the samples were collected in the mid-exponential growth phase. The carbohydrate compositions were determined for neutral and amino sugars (Korea Basic Science Institute, Daejeon, Korea). The neutral and amino sugars were extracted from cells by treating them with 2 M trifluoroacetic acid and 6 N HCl, respectively, at 100°C for 4 hours. The carbohydrates were analyzed by ICD-5000 (Dionex, Sunnyvale, CA, USA) equipped with a CarboPac PA10 column (4.5 × 250 mm, Dionex) and a CarboPac PA10 cartridge (4 × 50 mm). 16 mM NaOH was used as a mobile phase with a flow rate of 1.0 mL∙ min^-1^. Then, data were analyzed by Chromelon ver 6.8 software. Amino acid compositions were analyzed by a Hewlett Packard 1100 series HPLC systems equipped with Waters Nova-Pak C18 4 um column (3.9 × 300 mm) (Korea Basic Science Institute, Daejeon, Korea). The fatty acid composition of *K. oxytoca* was determined by Sherlock microbial identification system of Sherlock version 6.1 (Korea Research Institute of Bioscience and Biotechnology, Daejeon, Korea). DNA and RNA compositions were analyzed based on genome information of *K. oxytoca*. The DNA composition was calculated by using the GC content (53%) of *K. oxytoca*. The RNA composition was calculated based on nucleotide sequences for rRNA and tRNA derived from genome information. The compositions for other components were obtained from literature or assumed reasonably described in Additional file 3.

### Constraints-based flux analysis

In order to perform *in silico* simulations, and to predict the metabolic characteristics of *K. oxytoca*, constraints-based flux analysis, including FBA, was carried out under the assumption of a pseudo-steady state [35,40-43]. In order to simulate the *in silico* model more accurately, the limits of uptake and secretion rates for some metabolites, including amino acids and organic acids such as acetic acid, ethanol, formic acid, lactic acid, pyruvic acid, and succinic acid, were constrained by experimentally measured flux values. Likewise, the limits related with secretion for some metabolites, which were not produced during fermentation such amino acids, were constrained to zero.

The effect of 2,3-BD production rate in response to varying oxygen uptake rate was examined by flux response analysis [44-46]. The 2,3-BD production rate, as objective function, was maximized and minimized according to the changes of oxygen uptake rate from 0 to 30 mmol/gDCW/h with a 10 mmol/gDCW/h glucose uptake rate.

### Fermentation

For seed preparation, suspended cells from single colonies on Luria-Bertani (LB) agar (Difco Laboratories, Detroit, MI) plates were precultured in 20 mL test tubes containing 5 mL culture medium at 37°C for 5 h. 1 mL aliquots of the preculture were then transferred to 500 mL Erlenmeyer flasks containing 300 mL culture medium, and the cells were cultivated to an optical density of 1.5 ~ 2.0 at 600 nm (OD_600_). The tube and flask cultivations were placed in a rotary shaker at 150 rpm and 37°C (JEIO Tech. Co. SI-900R). The culture medium contained per liter: yeast extract (Becton Dickinson, Le Pont de Claix, France), 5 g; FeSO_4_ · 7H_2_O, 0.05 g; ZnSO_4_ · 7H_2_O, 0.001 g, MnSO_4_ · H_2_O, 0.001 g; CaCl_2_∙ 2H_2_O, 0.001 g; MgSO_4_∙ 7H_2_O, 0.25 g; (NH_4_)_2_SO_4_, 6.6 g; K_2_HPO_4_, 8.7 g; KH_2_PO_4_, 6.8 g; trace metal solution, 10 mL. The trace metal solution contains per liter: FeSO_4_∙ 7H_2_O, 5 g; ZnSO_4_∙ 7H_2_O, 0.1 g; MnSO_4_∙ H_2_O, 0.1 g; CaCl_2_∙ 2H_2_O, 0.1 g; HCl 10 mL.

Batch fermentations were performed in a 5-L BIOFLO & CELLIGEN 310 bioreactor (New Brunswick. Scientific Co., Edison, NJ) with 3 L culture medium containing 90 g/L of D-glucose and 300 mL seed culture. The fermenter was continuously aerated through a 0.2 μm membrane filter at a flow rate of 1 vvm (air volume∙ working volume^-1^∙ minute^-1^). The temperature was maintained at 37°C. The pH was controlled at 6.5 ± 0.1 by the automatic feeding of 5 N NaOH. Foaming was controlled by the addition of Antifoam 289 (Sigma, St. Louis, MO). For biomass composition analysis, the cells were cultured in a medium in which only yeast extract was removed from the culture medium. All fermentations were performed at least three times independently, and the representative results are shown in Figures.

### Analytical procedures

The concentrations of D-glucose and metabolites, including 2,3-BD, formic acid, ethanol, acetic acid, lactic acid, succinic acid, and acetoin, were determined by a high-performance liquid chromatography equipped with UV/VIS and RI detectors (Agilent 1260 series, Agilent Technologies, Waldbronn, Germany). An Aminiex HPX-87H column (300 mm × 7.8 mm, Bio-Rad, Hercules, CA) was isocratically eluted with 0.01 N H_2_SO_4_ at 80°C and a flow rate of 0.6 mL/min. The OD_600_ was measured using a UV–vis spectrophotometry (DR5000, Hach Company, CO) to monitor cell growth. Cell concentration, DCW per liter of culture broth, was calculated from the pre-determined standard curve relating OD_600_ to DCW (1 OD_600_ = 0.3877 ± 0.0136 g DCW∙ L^-1^). DCW was determined by filtering the culture broth through a 0.45 μm membrane and washing it twice with an equal volume of deionized distilled water. The filtered cells were then dried at 80 ± 5°C overnight and cooled to room temperature in a desiccator prior to weighting.

## Abbreviations

1,3-PD: 1,3-propanediol; 2,3-BD: 2,3-butanediol; ORF: Open reading frame; FBA: Flux balance analysis; KEGG: Kyoto encyclopedia of genes and genomes; LB: Luria-bertani; vvm: Air volume• working volume^-1^• minute^-1^; gDCW: Gram dry cell weight; DO: Dissolved oxygen; rpm: Revolutions per minute

## Competing interests

The authors declare that they have no competing interests.

## Authors’ contributions

JMP, HS, HJL, and DS generated ideas. JMP and HS designed the research. JMP performed the research. JMP performed analytical experiments. JMP analyzed data. JMP, HS, HJL, and DS wrote the paper. All authors read and approved the final manuscript.

## Supplementary Material

Additional file 1 **List of metabolic reactions in the genome-scale metabolic model of *Klebsiella oxytoca.*** Click here for file

Additional file 2 **List of metabolites in the genome-scale metabolic model of *Klebsiella oxytoca.*** Click here for file

Additional file 3 **Biomass composition of *Klebsiella oxytoca.*** Click here for file
