# Supplementary material for: Genome-scale reconstruction and in silico analysis of Klebsiella oxytoca for 2,3-butanediol production
Source: Microb Cell Fact. 2013 Feb 23;12:20. doi: 10.1186/1475-2859-12-20 (PMC3602198; doi:10.1186/1475-2859-12-20)
Supplement: Additional file 1 — List of metabolic reactions in the genome-scale metabolic model of Klebsiella oxytoca. [file 1475-2859-12-20-S1.pdf]

Additional file 1. List of metabolic reactions in the genome-scale metabolic model of *Klebsiella oxytoca*

Reaction name	Metabolism	Enzyme	E.C. number	Locus(KOX)	Reaction (KOX)
ACLDn	Naphthalene degradation	alcohol dehydrogenase	1.1.1.1	KOX_19595/KOX_20090/KOX_23025	hmnapth + nad -> napthah + nadh
SALCH3	Naphthalene degradation	salicylate hydroxylase	1.14.13.1	KOX_25675	msalc + o2 + nadh -> dhtolen + nad + co2
SALCH4	Naphthalene degradation	salicylate hydroxylase	1.14.13.1	KOX_25675	msalc4 + o2 + nadh -> 4mctch + nad + co2
ACLDnp	Naphthalene degradation	alcohol dehydrogenase	1.1.1.1	KOX_19595/KOX_20090/KOX_23025	2napthm + nad -> 2napald + nadh
SALCH5	Naphthalene degradation	salicylate hydroxylase	1.14.13.1	KOX_25675	4hmsalc + o2 + nadh -> 4hmcatech + nad + co2
ALHD15	1,2-Dichloroethane degradation	Chloroalkane and chloroalkene degradation	1.2.1.3	KOX_00375	chacald + nad -> chac + nadh
CMBLD3	Chlorocyclohexane and chlorobenzene degradation	carboxymethylenebutenolidase	3.1.1.45	KOX_02855/KOX_07760	c2ch4cmo -> 2chmac
CMBLD4	Chlorocyclohexane and chlorobenzene degradation	carboxymethylenebutenolidase	3.1.1.45	KOX_02855/KOX_07760	c4cmbo -> 2mac
CMBLD5	Chlorocyclohexane and chlorobenzene degradation	carboxymethylenebutenolidase	3.1.1.45	KOX_02855/KOX_07760	protmn -> cacac
ALCDt	Chloroalkane and chloroalkene degradation	alcohol dehydrogenase	1.1.1.1	KOX_19595/KOX_20090/KOX_23025	t3chp + nad -> 3chroald + nadh
ALHD13	Chloroalkane and chloroalkene degradation	aldehyde dehydrogenase (NAD+)	1.2.1.3	KOX_00375	3chroald -> t3chc
ACLDc	Chloroalkane and chloroalkene degradation	alcohol dehydrogenase	1.1.1.1	KOX_19595/KOX_20090/KOX_23025	c3chp + nad -> c3chroald + nadh
ALHD14	Chloroalkane and chloroalkene degradation	aldehyde dehydrogenase (NAD+)	1.2.1.3	KOX_00375	c3chroald -> c3chroc
DADH	Alanine and Aspartate Metabolism	D-Amino acid dehydrogenase	1.4.99.1	KOX_23495	dala + fad -> fadh2 + nh4 + pyr
ASPTA	Alanine and Aspartate Metabolism	aspartate transaminase	2.6.1.1	KOX_16370	akg + asp <-> glu + oaa
ALATA	Alanine and Aspartate Metabolism	alanine transaminase	2.6.1.2	KOX_26405	ala + akg <-> pyr + glu
ASNN	Alanine and Aspartate Metabolism	L-asparaginase	3.5.1.1	KOX_17835/KOX_18055(ansA)	asn -> asp + nh4
ASPAML1	Alanine and Aspartate Metabolism	aspartate--ammonia ligase	6.3.1.1	KOX_06770	asp + atp + nh4 -> asn + amp + ppi
ALAR	D-Alanine Metabolism	alanine racemase	5.1.1.1	KOX_08305(alr)/KOX_23490	ala <-> dala
ASNS	Alanine and Aspartate Metabolism	asparagine synthase (glutamine-hydrolysing)	6.3.5.4	KOX_14335(asnB)	asp + atp + gln -> amp + asn + glu + ppi
ARGSUCS	Alanine and Aspartate Metabolism	argininosuccinate synthase	6.3.4.5	KOX_03675	asp + atp + citr -> amp + argsucc + ppi
4AMBUAT	Alanine and Aspartate Metabolism	4-aminobutyrate aminotransferase	2.6.1.19	KOX_04875	bala + akg <-> 3opp + glu
T2AKGTA	beta-Alanine Metabolism	taurine---2-oxoglutarate transaminase	2.6.1.55	KOX_13775	bala + akg <-> 3opp + glu
PRIAMOX	beta-Alanine Metabolism	primary-amine oxidase	1.4.3.21	KOX_19410(tynA)	13dapro + o2 -> bapa + nh4 + h2o2
ASPR	Alanine and Aspartate Metabolism	aspartate racemase	5.1.1.13	KOX_18745	asp <-> asp-D
ASPOX1	Alanine and Aspartate Metabolism	L-aspartate oxidase	1.4.3.16	KOX_27585	asp + o2 <-> oaa + nh4 + h2o2
SUCSD1	Alanine and Aspartate Metabolism	succinate-semialdehyde dehydrogenase (NADP)	1.2.1.16	KOX_09980/KOX_11825(gabD)/KOX_24465	nadp + sucsal <-> nadph + succ
ASP1DCB	Alanine and Aspartate Metabolism	aspartate 1-decarboxylase	4.1.1.11	KOX_11205	asp -> bala + co2
GTADT2	Aminoacyl-tRNA biosynthesis	aspartyl-tRNA(Asn)/glutamyl-tRNA (Gln) amidotransferase	6.3.5.6	KOX_16785	asptrna + gln + atp -> asntrna + glu + pi + adp
ASPAML2	Alanine and aspartate metabolism	aspartate ammonia-lyase	4.3.1.1	KOX_08650(aspA)	asp -> fum + nh4
METTRFT	Aminoacyl-tRNA Biosynthesis	Methionyl-tRNA formyltransferase	2.1.2.9	KOX_04210(fmt)	fthf + mettrna -> fmettrna + thf
TYRTRS	Aminoacyl-tRNA Biosynthesis	tyrosyl-tRNA synthetase	6.1.1.1	KOX_22025	atp + trnatyr + tyr -> amp + ppi + tyrtrna
METTRS	Aminoacyl-tRNA Biosynthesis	Methionyl-tRNA synthetase	6.1.1.10	KOX_25560(metG)	atp + met + trnamet -> amp + mettrna + ppi
SERTRS	Aminoacyl-tRNA Biosynthesis	Seryl-tRNA synthetase	6.1.1.11	KOX_15915	atp + ser + trnaser -> amp + ppi + sertrna
SECTRS	Aminoacyl-tRNA Biosynthesis	Seryl-tRNA synthetase	6.1.1.11	KOX_15915	atp + ser + trnasec -> amp + ppi + sectrna
ASPTRS	Aminoacyl-tRNA Biosynthesis	Aspartyl-tRNA synthetase	6.1.1.12	KOX_23945(aspS)	asp + atp + trnaasp -> amp + asptrna + ppi
ASNTRS	Aminoacyl-tRNA Biosynthesis	Asparaginyl-tRNA synthetase	6.1.1.22	KOX_16105(asnC)	atp + asn + trnaasn -> amp + asntrna + ppi
GLYTRS	Aminoacyl-tRNA Biosynthesis	Glycyl-tRNA synthetase	6.1.1.14	KOX_05595(glyS)/KOX_05600(glyQ)	atp + gly + trnagly -> amp + glytrna + ppi
PROTRS	Aminoacyl-tRNA Biosynthesis	Prolyl-tRNA synthetase	6.1.1.15	KOX_11580	atp + pro + trnapro -> amp + ppi + protrna
CYSTRS	Aminoacyl-tRNA Biosynthesis	Cysteinyl-tRNA synthetase	6.1.1.16	KOX_13215(cysS)	atp + cys + trnacys -> amp + cystrna + ppi
GLNTRS	Aminoacyl-tRNA Biosynthesis	Glutaminyl-tRNA synthetase	6.1.1.18	KOX_14370	atp + gln + trnagln -> amp + glntrna + ppi
ARGTRS	Aminoacyl-tRNA Biosynthesis	Arginyl-tRNA synthetase	6.1.1.19	KOX_23990(argS)	arg + atp + trnaarg -> amp + argtrna + ppi
TRPTRS	Aminoacyl-tRNA Biosynthesis	Tryptophanyl-tRNA synthetase	6.1.1.2	KOX_04580/KOX_09720	atp + trnatrp + trp -> amp + ppi + trptrna
PHETRS	Aminoacyl-tRNA Biosynthesis	Phenylalanyl-tRNA synthetase	6.1.1.20	KOX_22905(pheT)/KOX_22910(pheS)	atp + phe + trnaphe -> amp + phetrna + ppi
HISTRS	Aminoacyl-tRNA Biosynthesis	Histidyl-tRNA synthetase	6.1.1.21	KOX_27270(hisS)	atp + his + trnahis -> amp + histrna + ppi
THRTRS	Aminoacyl-tRNA Biosynthesis	Threonyl-tRNA synthetase	6.1.1.3	KOX_22930(thrS)	atp + thr + trnathr -> amp + ppi + thrtrna
LEUTRS	Aminoacyl-tRNA Biosynthesis	Leucyl-tRNA synthetase	6.1.1.4	KOX_14265(leuS)	atp + leu + trnaleu -> amp + leutrna + ppi
ILETRS	Aminoacyl-tRNA Biosynthesis	Isoleucyl-tRNA synthetase	6.1.1.5	KOX_10530(ileS)	atp + ile + trnaile -> amp + iletrna + ppi
LYSTRS	Aminoacyl-tRNA Biosynthesis	Lysyl-tRNA synthetase	6.1.1.6	KOX_02370(lysS)	atp + lys + trnalys -> amp + lystrna + ppi
ALATRS	Aminoacyl-tRNA Biosynthesis	Alanyl-tRNA synthetase	6.1.1.7	KOX_00530(alaS)	ala + atp + trnaala -> alatrna + amp + ppi
VALTRS	Aminoacyl-tRNA Biosynthesis	Valyl-tRNA synthetase	6.1.1.9	KOX_09325(valS)	atp + trnaval + val -> amp + ppi + valtrna
ACG6PD	Aminosugars metabolism	N-acetylglucosamine-6-phosphate deacetylase	3.5.1.25	KOX_14355(nagA)	naga6p -> ac + ga6p
GM6PD	Aminosugars metabolism	glucosamine-6-phosphate deaminase	3.5.99.6	KOX_14360(nagB)	ga6p -> f6p + nh4
UAEPGR	Aminosugars metabolism	UDP-N-acetylenolpyruvoylglucosamine reductase	1.1.1.158	KOX_07860(murB)	nadh + uaccg -> nad + udpnam
UAEPGRp	Aminosugars metabolism	UDP-N-acetylenolpyruvoylglucosamine reductase	1.1.1.158	KOX_07860(murB)	nadph + uaccg -> nadp + udpnam
GA1PACT	Aminosugars metabolism	glucosamine-1-phosphate N-acetyltransferase	2.3.1.157	KOX_06685(glmU)	accoa + ga1p -> naga1p + coa
UNAGCVT	Aminosugars metabolism	UDP-N-acetylglucosamine 1-carboxyvinyltransferase	2.5.1.7	KOX_03750	pep + udpnag -> pi + uaccg
GF6PT	Aminosugars metabolism	glutamine-fructose-6-phosphate transaminase	2.6.1.16	KOX_06680	f6p + gln -> ga6p + glu
UNAGDP	Aminosugars metabolism	UDP-N-acetylglucosamine diphosphorylase	2.7.7.23	KOX_06685(glmU)	naga1p + utp -> ppi + udpnag
PGAMT	Aminosugars metabolism	phosphoglucosamine mutase	5.4.2.10	KOX_03685(glmM)	ga1p <-> ga6p
UDPACG	Aminosugars metabolism	UDP-N-acetylglucosamine 4-epimerase	5.1.3.7		udpnag <-> udpacgal
NADMA6PE	Aminosugars metabolism	N-acetylmannosamine-6-phosphate 2-epimerase	5.1.3.9	KOX_03880	nadma6p -> naga6p
NAMUR6PE	Aminosugars metabolism	N-acetylmuramic acid 6-phosphate etherase	4.2.1.126	KOX_06285	namur6p <-> naga6p + lac
NAHEXM	Aminosugars metabolism	beta-N-acetylhexosaminidase	3.5.1.52	KOX_17360	chitobiose -> 2 naga
NAGAK	Aminosugars metabolism	N-acetylglucosamine kinase	2.7.1.59	KOX_17425	naga + atp <-> naga6p + adp
NADDAC	Aminosugars metabolism	NAD-dependent deacetylase	3.5.1.-	KOX_17430	ga6p <-> f6p + nh4
UDPNAGE1	Aminosugars metabolism	UDP-N-acetylglucosamine 2-epimerase	5.1.3.14	KOX_07565	udpnag -> nadma + udp
NADMAK	Aminosugars metabolism	N-acylmannosamine kinase	2.7.1.60	KOX_03875	nadma + atp -> nadma6p + adp
NANEUL	Aminosugars metabolism	N-acetylneuraminate lyase	4.1.3.3	KOX_03890	naneu -> nadma + pyr
UDPNAGE2	Aminosugars metabolism	UDP-N-acetylglucosamine 2-epimerase	5.1.3.14	KOX_07565	udpnag -> udpnadma
UDPNADMAD	Aminosugars metabolism	UDP-N-acetyl-D-mannosamine dehydrogenase	1.1.1.-	KOX_07570(wecC)	udpnadma + 2 nad -> udpnadmarn + 2 nadh
UDPGLCURD	Aminosugars metabolism	UDP-4-amino-4-deoxy-L-arabinose formyltransferase / UDP-glucuronic acid dehydrogenase (UDP-4-keto-hexauronic acid decarboxylating)	1.1.1.305	KOX_05070	udpglcur + nad -> udpara4o + co2 + nadh
UDPARAT	Aminosugars metabolism	UDP-4-amino-4-deoxy-L-arabinose-oxoglutarate aminotransferase	2.6.1.87	KOX_05080	udpara4o + glu -> udpara4n + akg
UDPARAFT	Aminosugars metabolism	UDP-4-amino-4-deoxy-L-arabinose formyltransferase / UDP-glucuronic acid dehydrogenase (UDP-4-keto-hexauronic acid decarboxylating)	2.1.2.13	KOX_05070	fthf + udpara4n -> thf + udpara4fn
UDPARA4FNT	Aminosugars metabolism	undecaprenyl-phosphate 4-deoxy-4-formamido-L-arabinose transferase	2.7.8.30	KOX_05075	udpara4fn + udcpp -> udcpara4fn + udp
ME1	Anaplerotic Reactions	malic enzyme (NAD)	1.1.1.38	KOX_20115/KOX_23885	mal + nad -> co2 + nadh + pyr
ME2	Anaplerotic Reactions	malic enzyme (NADP)	1.1.1.40	KOX_27030	mal + nadp -> co2 + nadph + pyr
OAADC	Anaplerotic Reactions	oxaloacetate decarboxylase	4.1.1.3	KOX_03945/KOX_03970/KOX_10610/KOX_10615	oaa -> pyr + co2
PPA1	Anaplerotic Reactions	inorganic diphosphatase	3.6.1.1	KOX_09090/KOX_20095	ppi -> 2 pi
PPA2	Anaplerotic Reactions	inorganic diphosphatase	3.6.1.1	KOX_09090/KOX_20095	ppi -> 2 pi + hext
PPC	Anaplerotic Reactions	phosphoenolpyruvate carboxylase	4.1.1.31	KOX_07360	pep + co2 -> oaa + pi
PPCK	Anaplerotic Reactions	phosphoenolpyruvate carboxykinase	4.1.1.49	KOX_04670/KOX_23370	atp + oaa -> adp + co2 + pep
ICL	Anaplerotic Reactions	Isocitrate lyase	4.1.3.1	KOX_08045	icit -> glx + succ
MALS	Anaplerotic Reactions	malate synthase	2.3.3.9	KOX_08040	accoa + glx -> coa + mal
P5CD1	Arginine and Proline Metabolism	1-pyrroline-5-carboxylate dehydrogenase	1.5.1.12	KOX_17015(putA)	p5c + nad -> glu + nadh
P5CR1	Arginine and Proline Metabolism	pyrroline-5-carboxylate reductase	1.5.1.2	KOX_12395/KOX_21070	p5c + nadph <-> nadp + pro
P5CR3	Arginine and Proline Metabolism	pyrroline-5-carboxylate reductase	1.5.1.2	KOX_12395/KOX_21070	p5c + nadh <-> nad + pro
PROD2	Arginine and Proline Metabolism	Proline dehydrogenase	1.5.99.8	KOX_17015(putA)	fad + pro -> p5c + fadh2
ORNCBT	Arginine and Proline Metabolism	ornithine carbamoyltransferase	2.1.3.3	KOX_09300	cap + orn <-> citr + pi

ARGSCL	Arginine and Proline Metabolism	argininosuccinate lyase	4.3.2.1	KOX_07380	argsucc <-> arg + fum
GLU5SDT	Arginine and Proline Metabolism	L-glutamate 5-semialdehyde dehydratase (spontaneous)	spontaneous		glugsal -> p5c
GLUDH4	Arginine and Proline metabolism	glutamate dehydrogenase	1.4.1.3	KOX_19525	glu + nadp <-> akg + nh4 + nadph
P5CD2	Arginine and Proline metabolism	1-pyrroline-5-carboxylate dehydrogenase	1.5.1.12	KOX_17015(putA)	glugsal + nad <-> glu + nadh
P5CD3	Arginine and Proline metabolism	1-pyrroline-5-carboxylate dehydrogenase	1.5.1.12	KOX_17015(putA)	4hglusa + nad -> e4hglu + nadh
PROD3	Arginine and Proline metabolism	Proline dehydrogenase	1.5.99.8	KOX_17015(putA)	4hglusa + nad -> e4hglu + nadh
P5CR2	Arginine and Proline metabolism	pyrroline-5-carboxylate reductase	1.5.1.2	KOX_12395/KOX_21070	l1p3h5c + nadh -> 4hpro + nad
P5CD4	Arginine and Proline metabolism	1-pyrroline-5-carboxylate dehydrogenase	1.5.1.12	KOX_17015(putA)	l1p3h5c + nad -> e4hglu + nadh
P5CD6	Arginine and Proline metabolism	1-pyrroline-5-carboxylate dehydrogenase	1.5.1.12	KOX_17015(putA)	l1p3h5c + nadp -> e4hglu + nadph
PTO4H	Arginine and Proline metabolism				l1p3h5c <-> 4hglusa
P5CD5	Arginine and Proline metabolism	1-pyrroline-5-carboxylate dehydrogenase	1.5.1.12	KOX_17015(putA)	4hglusa + nadh -> e4hglu + nad
PROD4	Arginine and Proline metabolism	Proline dehydrogenase	1.5.99.8	KOX_17015(putA)	4hglusa + nadh -> e4hglu + nad
ASPAM4	Arginine and Proline metabolism	aspartate aminotransferase	2.6.1.1	KOX_16370	e4hglu + akg -> hydroxyakg + glu
HOGAD	Arginine and Proline metabolism	4-hydroxy-2-oxoglutarate aldolase	4.1.2.14	KOX_23850	hydroxyakg <-> pyr + glx
OAADC2	Arginine and Proline metabolism	oxaloacetate decarboxylase	4.1.1.3	KOX_03945/KOX_03970/KOX_10610/KOX_10615	hydroxyakg <-> pyr + glx
CREAH	Arginine and Proline metabolism	creatinine amidohydrolase	3.5.2.10	KOX_20850	cretn <-> creatine
P5CD1p	Arginine and Proline Metabolism	1-pyrroline-5-carboxylate dehydrogenase	1.5.1.12	KOX_17015(putA)	p5c + nadp <-> glu + nadph
P5CR2p	Arginine and Proline metabolism	pyrroline-5-carboxylate reductase	1.5.1.2	KOX_12395/KOX_21070	l1p3h5c + nadph -> 4hpro + nadp
ACORND2	Arginine and Proline metabolism	acetylornithine deacetylase	3.5.1.16	KOX_07365/KOX_16395/KOX_19240	accitr -> ac + citr
UREAC	Arginine and Proline metabolism	urea carboxylase	6.3.4.6	KOX_20285	atp + urea + hco3 <-> adp + pi + u1car
ARGSUCCT	Arginine and Proline metabolism	arginine N-succinyltransferase	2.3.1.109	KOX_18145	succoa + arg -> coa + succarg
SUCCARGD	Arginine and Proline metabolism	succinylarginine dihydrolase	3.5.3.23	KOX_18155	succarg -> succorn + co2 + 2 nh4
SUCCORNAT	Arginine and Proline metabolism	succinylornithine aminotransferase	2.6.1.81	KOX_18140	succorn + akg -> succglusa + glu
SUCCGLUSAD	Arginine and Proline metabolism	succinylglutamic semialdehyde dehydrogenase	1.2.1.71	KOX_18150	succglusa + nad -> succglu + nadh
SUCCGLUDS	Arginine and Proline metabolism	succinylglutamate desuccinylase	3.5.1.96	KOX_18160	succglu -> glu + succ
PTRCAT	Arginine and Proline metabolism	putrescine aminotransferase	2.6.1.82	KOX_03220	akg + ptrc -> 4ab + glu
DIAMACT	Arginine and Proline metabolism	diamine N-acetyltransferase	2.3.1.57	KOX_21535	accoa + ptrc -> coa + acputs
SPRMDAT1	Arginine and Proline metabolism	Spermidine acetyltransferase	2.3.1.57	KOX_21535	accoa + sprmd -> n1acsprmd + coa
SPRMDAT2	Arginine and Proline metabolism	Spermidine acetyltransferase	2.3.1.57	KOX_21535	accoa + sprmd -> n8acsprmd + coa
GLUPTRCS	Arginine and Proline metabolism	gamma-glutamylputrescine synthase	6.3.1.11	KOX_16935	atp + glu + ptrc -> adp + pi + gluptrc
GLUPTRCOX	Arginine and Proline metabolism	gamma-glutamylputrescine oxidase	1.4.3.-	KOX_16915	gluptrc + o2 -> gluamibut + nh4 + h2o2
GLUAMIBUTD	Arginine and Proline metabolism	gamma-glutamyl-gamma-aminobutyraldehyde dehydrogenase	1.2.1.-	KOX_16920	gluamibut + nad -> gluamibutr + nadh
GLUAMIBUTRH	Arginine and Proline metabolism	gamma-glutamyl-gamma-aminobutyrate hydrolase	3.5.1.94	KOX_16930(puuD)	gluamibutr -> gaba + glu
ORNDC	Arginine and Proline metabolism	ornithine decarboxylase	4.1.1.17	KOX_02775	orn -> ptrc + co2
GLUGSALD	Arginine and Proline metabolism	L-glutamate 5-semialdehyde dehydratase (spontaneous)			glugsal <-> p5c
GLCRD1	Ascorbate and Aldarate metabolism	glucarate dehydratase	4.2.1.40	KOX_01175	dgluca <-> d4dg
GALCTDH	Ascorbate and Aldarate metabolism	galactarate dehydratase	4.2.1.42	KOX_03480	dgal -> d4dg
DGLUCAL1	Ascorbate and Aldarate metabolism	2-dehydro-3-deoxyglucarate aldolase	4.1.2.20	KOX_03470	d4dg <-> pyr + h3op
ADLD	Ascorbate and Aldarate metabolism	aldehyde dehydrogenase (NAD+)	1.2.1.3	KOX_00375	dglucl + nad <-> dgluca + nadh
GLCRD2	Ascorbate and Aldarate metabolism	glucarate dehydratase	4.2.1.40	KOX_01175	dgluca <-> d3dg
DGLUCAL2	Ascorbate and Aldarate metabolism	2-dehydro-3-deoxyglucarate aldolase	4.1.2.20	KOX_03470	d3dg <-> pyr + h3op
ASCB6PL	Ascorbate and Aldarate metabolism	L-ascorbate 6-phosphate lactonase	3.1.1.-	KOX_08920	ascb6p -> 3dhg6p
LRIB5P4E2	Ascorbate and Aldarate metabolism	L-ribulose-5-phosphate 4-epimerase	5.1.3.4	KOX_08950(sgaE)	lrl5p <-> xu5p
ARABNLAC	Ascorbate and Aldarate metabolism	L-arabinonolactonase	3.1.1.15	KOX_24440	arabnlac <-> arabn
AMDS6	Benzoate degradation via CoA ligation	amidase	3.5.1.4	KOX_09850/KOX_13720/KOX_2051	bzamid -> benzot + nh4
APPS3	Benzoate degradation via CoA ligation	acylphosphatase	3.6.1.7	KOX_16295	bzop -> benzot + pi
NITRH	Benzoate degradation via CoA ligation	nitrile hydratase	4.2.1.84	KOX_20500	bzonit -> bzamid
VANMOX	Benzoate degradation via CoA ligation	vanillate monooxygenase	1.14.13.82	KOX_16720	vanillate + o2 + nadh -> 34dhb + nad + formald
3HBCDH	Benzoate degradation via CoA ligation	3-hydroxybutyryl-CoA dehydrogenase	1.1.1.157	KOX_19455	3hbcoa + nadp -> aacoa + nadph
PHBZMN	Benzoate degradation via Hydroxylation	p-hydroxybenzoate 3-monooxygenase	1.14.13.2	KOX_10205	4hb + o2 + nadph -> 34dhb + nadp
PROTCC1	Benzoate degradation via Hydroxylation	protocatechuate 3,4-dioxygenase	1.13.11.3	KOX_19615/KOX_19620	34dhb + o2 -> carccm
4OXCTT	Benzoate degradation via Hydroxylation	4-oxalocrotonate tautomerase	5.3.2.-	KOX_02090/KOX_19920/KOX_20805	2hmuc -> zoe
3OXAPLC	Benzoate degradation via Hydroxylation	3-oxoadipate enol-lactonase	3.1.1.24	KOX_21740	2odhfac -> oadip
3OXAPT	Benzoate degradation via Hydroxylation	3-oxoadipate CoA-transferase	2.8.3.6	KOX_21755/KOX_21760	succoa + oadip -> succ + ooadpcoa
3OXADCT	Benzoate degradation via Hydroxylation	3-oxoadipyl-CoA thiolase	2.3.1.16	KOX_07830(fadA)/KOX_26660(fadI)	ooadpcoa + coa -> succoa + accoa
3CMUCC	Benzoate degradation via Hydroxylation	3-carboxy-cis,cis-muconate cycloisomerase	5.5.1.2	KOX_21745	carccm <-> gcarmclc
4CBMCLC	Benzoate degradation via Hydroxylation	4-carboxymuconolactone decarboxylase	4.1.1.44	KOX_02105/KOX_07320/KOX_20565/KOX_20900/ KOX_21735/KOX_24470	gcarmclc <-> 2odhfac + co2
PROTCC2	Benzoate degradation via Hydroxylation	protocatechuate 3,4-dioxygenase	1.13.11.3	KOX_19615/KOX_19620	gallate + o2 -> 2py46dc
CARHM2	Benzoate degradation via Hydroxylation	5-carboxymethyl-2-hydroxymuconate isomerase	5.3.3.10	KOX_10040	4c2hhd <-> 4obtc
DMRATT	Terpenoid backbone biosynthesis	geranyltranstransferase	2.5.1.1	KOX_12610	dmpp + ipp -> gpp + ppi
IPPDI	Terpenoid backbone biosynthesis	isopentenyl-diphosphate delta-isomerase	5.3.3.2	KOX_02365	ipp <-> dmpp
GRATT	Terpenoid backbone biosynthesis	geranyltranstransferase	2.5.1.10	KOX_12610	gpp + ipp -> frdp + ppi
CDPMDEK	Terpenoid backbone biosynthesis	4-(cytidine 5'-diphospho)-2-C-methyl-D-erythritol kinase	2.7.1.148	KOX_23200(ipk)	cdpmde + atp -> 2pcdpmde + adp
HMB4PPR	Terpenoid backbone biosynthesis	1-hydroxy-2-methyl-2-(E)-butenyl 4-diphosphate reductase (dmpp)	1.17.1.2	KOX_10545(ispH)	hmb4pp + nadh -> dmpp + nad
DOXRTI	Terpenoid backbone biosynthesis	1-deoxy-D-xylulose reductoisomerase	1.1.1.267	KOX_11465	dx5p + nadph -> mde4p + nadp
DOXPS	Terpenoid backbone biosynthesis	1-deoxy-D-xylulose 5-phosphate synthase	2.2.1.7	KOX_12605	g3p + pyr -> co2 + dx5p
HMB4DPR	Terpenoid backbone biosynthesis	1-hydroxy-2-methyl-2-(E)-butenyl 4-diphosphate reductase	1.17.1.2	KOX_10545(ispH)	hmb4pp + nadh -> ipp + nad
MECDPDHT	Terpenoid backbone biosynthesis	2C-methyl-D-erythritol 2,4 cyclodiphosphate dehydratase	1.17.7.1	KOX_27275(ispG)	mdecpp -> hmb4pp
MECDPS	Terpenoid backbone biosynthesis	2-C-methyl-D-erythritol 2,4-cyclodiphosphate synthase	4.6.1.12	KOX_01055(ispF)	2pcdpmde -> mdecpp + cmp
ME4PCT	Terpenoid backbone biosynthesis	2-C-methyl-D-erythritol 4-phosphate cytidylyltransferase	2.7.7.60	KOX_01060(ispD)	mde4p + ctp -> cdpmde + ppi
OCTPPS	Terpenoid backbone biosynthesis	Octaprenyl pyrophosphate synthase	2.5.1.90	KOX_03740	frdp + 5 ipp -> opp + 5 ppi
UDCPDPS	Terpenoid backbone biosynthesis	undecaprenyl diphosphate synthase	2.5.1.31	KOX_11470	frdp + 8 ipp -> udcpdp + 8 ppi
ALHD18	Limonene and pinene degradation	aldehyde dehydrogenase (NAD+)	1.2.1.3	KOX_00375	perllald + nad <-> perll + nadh
C2MIPCH	Limonene and pinene degradation	cis-2-Methyl-5-isopropylhexa-2,5-dienoyl-CoA hydro-lyase	4.2.1.17	KOX_07835(fadB)/KOX_19445/KOX_26655(fadJ)/	c2mipdcoa -> 3hdmmhcoa
T2MIPCH	Limonene and pinene degradation	trans-2-Methyl-5-isopropylhexa-2,5-dienoyl-CoA hydro-lyase	4.2.1.17	KOX_07835(fadB)/KOX_19445/KOX_26655(fadJ)/	t2mipdcoa -> 3hdmmhcoa
ISOCHORPL	Biosynthesis of siderophore group nonribosomal peptides	isochorismate pyruvate lyase	4.2.99.21	KOX_16850/KOX_20605	isochor -> salcyl + pyr
ISOCHORPH	Biosynthesis of siderophore group nonribosomal peptides	Isochorismate pyruvate-hydrolase	3.3.2.1	KOX_13960	isochor -> 23d23dhb + pyr
23D23DHBD	Biosynthesis of siderophore group nonribosomal peptides	2,3-dihydro-2,3-dihydroxybenzoate dehydrogenase	1.3.1.28	KOX_13965	23d23dhb + nad -> 23dhb + nadh
23DHBAS	Biosynthesis of siderophore group nonribosomal peptides	2,3-dihydroxybenzoate adenylate synthase	2.7.7.58	KOX_13955(entE)	23dhb + atp <-> 23dhba + ppi
AOXNS	Biotin Metabolism	8-amino-7-oxononanoate synthase	2.3.1.47	KOX_14920	ala + pmcoa <-> aona + co2 + coa
AMAOXNT	Biotin Metabolism	adenosylmethionine-8-amino-7-oxononanoate transaminase	2.6.1.62	KOX_14910	aona + sam <-> samob + danna
BIOTS1	Biotin Metabolism	Biotin synthase	2.8.1.6	KOX_14915	sam + dtb + s -> bt + da-5 + met
BIOTS2	Biotin Metabolism	Biotin synthase (ala-L producing)	2.8.1.6	KOX_14915	cys + dtb -> bt + ala
DTBTS	Biotin Metabolism	dethiobiotin synthase	6.3.3.3	KOX_14930(bioD)/KOX_21575	atp + co2 + danna -> adp + dtb + pi

BTNACL	Biotin metabolism	biotin-[acetyl-CoA-carboxylase] ligase	6.3.4.15	KOX_07865	atp + bt -> ppi + b5amp
BTNACL2	Biotin metabolism	biotin-[acetyl-CoA-carboxylase] ligase	6.3.4.15	KOX_07865	b5amp + apoCAB -> amp + holoCAB
MALDO	Butanoate metabolism	(R)-Malate:NAD+ oxidoreductase (decarboxylating)/D-malate dehydrogenase	1.1.1.83	KOX_16905	dmal + nad -> co2 + nadh + pyr
ACALDb	Butanoate metabolism	acetaldehyde dehydrogenase	1.2.1.10	KOX_22675/KOX_23025	butanal + coa + nad -> c040coa + nadh
AC4HB	Butanoate metabolism	acetyl-CoA: 4-hydroxybutanoate CoA transferase (two-step reaction, unclear reaction)			4hbt + accoa <-> 3btecoa + ac
ALHD5	Butanoate metabolism	aldehyde dehydrogenase (NAD+)	1.2.1.3	KOX_00375	3b1a + nad -> 3but + nadh
ALACDC	Butanoate metabolism	acetolactate decarboxylase	4.1.1.5	KOX_22365	alac-S -> acetoin + co2
ACETRD	Butanoate metabolism	acetoin reductase	1.1.1.4	KOX_01940/KOX_22375	acetoin + nadh <-> 23bdo + nad
DIACTS	Butanoate metabolism	Spontaneous			alac-S + o2 -> diacetyl + co2
DACETRD	Butanoate metabolism	diacetyl reductase	1.1.1.303	KOX_01940/KOX_22375	diacetyl + nadh -> acetoin + nad
SUCCS	C5-Branched Dibasic acid metabolism	succinyl-CoA synthetase	6.2.1.5	KOX_14600(sucC)/KOX_14605	atp + itcn + coa <-> adp + pi + itcncoa
METASPAL	C5-Branched Dibasic acid metabolism	methylaspartate ammonia-lyase	4.3.1.2	KOX_14665	thr3masp -> mesacon + nh4
MDH1	Citric Acid Cycle	malate dehydrogenase	1.1.1.37	KOX_04000	mal + nad <-> nadh + oaa
ICITDp	Citric Acid Cycle	isocitrate dehydrogenase (NADP)	1.1.1.42	KOX_17560	icit + nadp <-> akg + co2 + nadph
MDH2	Citric Acid Cycle	Malate dehydrogenase (ubiquinone 8 as acceptor)	1.1.5.4	KOX_21685/KOX_26070	mal + uq -> oaa + uqh2
MDH3	Citric Acid Cycle	Malate dehydrogenase (menaquinone 8 as acceptor)	1.1.5.4	KOX_21685/KOX_26070	mal + mk -> oaa + mqn
CITL	Citric Acid Cycle	Citrate lyase	4.1.3.6	KOX_10630/KOX_10635/KOX_10640/KOX_16575/KOX_16580	cit -> ac + oaa
FUMR	Citric Acid Cycle	fumarase	4.2.1.2	KOX_14640/KOX_21730/KOX_21890(fumC)/KOX_21895	fum <-> mal
ACONT1	Citric Acid Cycle	aconitase (citrate hydro-lyase)	4.2.1.3	KOX_10580/KOX_11075/KOX_18415	cit <-> acon-C
ACONT2	Citric Acid Cycle	aconitase (isocitrate hydro-lyase)	4.2.1.3	KOX_10580/KOX_11075/KOX_18415	acon-C <-> icit
SUCOAS	Citric Acid Cycle	succinyl-CoA synthetase (ADP-forming)	6.2.1.5	KOX_14600(sucC)/KOX_14605	atp + coa + succ -> succoa + adp + pi
AKGDH	Citric Acid Cycle	2-Oxogluterate dehydrogenase	1.2.4.2/2.3.1.61/1.8.1.4	KOX_14590(sucA)/KOX_14595/KOX_11055/KOX_20455	akg + coa + nad -> co2 + nadh + succoa
CITS	Citric Acid Cycle	citrate synthase	2.3.3.1	KOX_14565(gltA)/KOX_25260	accoa + oaa -> cit + coa
SUCCDf	Citric Acid Cycle	succinate dehydrogenase (irreversible)	1.3.99.1	KOX_08760/KOX_08765/KOX_08770/KOX_08775/KOX_14570(sdhC)/KOX_14575(sdhD)/KOX_14580(sdhA)/KOX_14585(sdhB)/KOX_21720	succ + fad -> fum + fadh2
SUCCDm	Citric Acid Cycle	succinate dehydrogenase (irreversible)	1.3.99.1	KOX_08760/KOX_08765/KOX_08770/KOX_08775/KOX_14570(sdhC)/KOX_14575(sdhD)/KOX_14580(sdhA)/KOX_14585(sdhB)/KOX_21720	fum + mqn -> succ + mk
SUCCDd	Citric Acid Cycle	succinate dehydrogenase (irreversible)	1.3.99.1	KOX_08760/KOX_08765/KOX_08770/KOX_08775/KOX_14570(sdhC)/KOX_14575(sdhD)/KOX_14580(sdhA)/KOX_14585(sdhB)/KOX_21720	fum + 2dmmql8 -> succ + 2dmmq8
GGMT1	Cyanoamino acid metabolism	gamma-glutamyltranspeptidase	2.3.2.2	KOX_04780(ggt)/KOX_16795	cyaala + glu -> ggbap + co2
GGMT2	Cyanoamino acid metabolism	gamma-glutamyltranspeptidase	2.3.2.2	KOX_04780(ggt)/KOX_16795	cyaala + glu -> ggbcya
TSULSFT	Cyanoamino acid metabolism	thiosulfate sulfurtransferase	2.8.1.1	KOX_04705(glpE)/KOX_18100/KOX_27315(sseA)	cn + tsul -> so3 + tcynt
SEROAT	Cysteine Metabolism	serine O-acetyltransferase	2.3.1.30	KOX_05805(cysE)/KOX_18685	accoa + ser <-> aser + coa
CYSST1	Cysteine Metabolism	cysteine synthase	2.5.1.47	KOX_18690/KOX_26885(cysM)	aser + h2s -> ac + cys
CYSTBL2	Cysteine metabolism	cystathionine beta-lyase	4.4.1.8	KOX_02875/KOX_21920	cyst -> pyr + nh4 + tcys
CYSTBL4	Cysteine metabolism	cystathionine beta-lyase	4.4.1.8	KOX_02875/KOX_21920	cys -> h2s + pyr + nh4
CYTTS6	Cysteine metabolism	cystathionine gamma-synthase	2.5.1.48	KOX_07310	aser + tsul -> sslcys + ac
CYSST2	Cysteine metabolism	cysteine synthase	2.5.1.47	KOX_18690/KOX_26885(cysM)	aser + tsul -> sslcys + ac
SERDHT2	Cysteine metabolism	L-serine dehydratase	4.3.1.17	KOX_01230/KOX_23635	ser -> 2aa
CYSST3	Cysteine metabolism	cysteine synthase	2.5.1.47	KOX_12795/KOX_18690/KOX_26835/KOX_26885(cysM)	aser + tsul + rthio -> cys + so3 + othio + ac
ASPAM1	Cysteine metabolism	aspartate aminotransferase	2.6.1.1	KOX_16370	mpyr + glu <-> cys + akg
ASPAM2	Cysteine metabolism	aspartate aminotransferase	2.6.1.1	KOX_16370	3slala + akg -> 3sfpyr + glu
ASPAM3	Cysteine metabolism	aspartate aminotransferase	2.6.1.1	KOX_16370	cysteate + akg -> 3spyr + glu
L-LACD5	Cysteine metabolism	L-lactate dehydrogenase	1.1.1.27	KOX_21270	mpyr + nadh -> 3mlac + nad
SADT2	Cysteine Metabolism	Sulfate adenyltransferase	2.7.7.4	KOX_01080(cysN)/KOX_01085	atp + selnt <-> ppi + aselnt
DCYSDS	Cysteine Metabolism	D-cysteine desulfhydrase	4.4.1.15	KOX_18740	dcys -> h2s + nh4 + pyr
MPYRST	Cysteine Metabolism	3-mercaptopyruvate sulfurtransferase	2.8.1.2	KOX_18100/KOX_27315(sseA)	mpyr + so3 -> tsul + pyr
HSERST	Cysteine Metabolism	homoserine O-succinyltransferase	2.3.1.46	KOX_08035	succoa + hser -> coa + oslhser
SRHCYSL	Cysteine Metabolism	S-ribosylhomocysteine lyase	4.4.1.21	KOX_00500	srlh -> 45dhpen + hcys
ALAALAL	D-alanine metabolism	D-alanine-D-alanine ligase	6.3.2.4	KOX_10935(ddl)/KOX_12350	2 dala + atp <-> adp + alaala + pi
GLUN2	D-glutamine and D-glutamate metabolism	glutaminase	3.5.1.2	KOX_14645/KOX_21250	dgln -> dglu + nh4
FABC120	Fatty acid biosynthesis	Fatty acid biosynthesis (dodecanoic acid; c12:0)	2.3.1.41 AND 2.3.1.179 AND 2.3.1.180 AND 1.1.1.100 AND 4.2.1.- AND 1.3.1.9 AND 1.3.1.-	KOX_05025/KOX_26605/KOX_00905/KOX_05040/KOX_17300/KOX_17280/KOX_00890/KOX_02015(fabG)/KOX_05035(fabG)/KOX_16425/KOX_17290(fabG)/KOX_11500(fabZ)/KOX_18475/KOX_18960	acACP + 5 malACP + 10 nadph -> 10 nadp + c120ACP + 5 co2 + 5 ACP
FABC130	Fatty acid biosynthesis	Fatty acid biosynthesis (tridecanoic acid; c13:0)	2.3.1.41 AND 2.3.1.179 AND 2.3.1.180 AND 1.1.1.100 AND 4.2.1.- AND 1.3.1.9 AND 1.3.1.-	KOX_05025/KOX_26605/KOX_00905/KOX_05040/KOX_17300/KOX_17280/KOX_00890/KOX_02015(fabG)/KOX_05035(fabG)/KOX_16425/KOX_17290(fabG)/KOX_11500(fabZ)/KOX_18475/KOX_18960	ppacp + 5 malACP + 10 nadph -> 10 nadp + c130ACP + 5 co2 + 5 ACP
FABC140	Fatty acid biosynthesis	Fatty acid biosynthesis (tetradecanoic acid; c14:0)	2.3.1.41 AND 2.3.1.179 AND 2.3.1.180 AND 1.1.1.100 AND 4.2.1.- AND 1.3.1.9 AND 1.3.1.-	KOX_05025/KOX_26605/KOX_00905/KOX_05040/KOX_17300/KOX_17280/KOX_00890/KOX_02015(fabG)/KOX_05035(fabG)/KOX_16425/KOX_17290(fabG)/KOX_11500(fabZ)/KOX_18475/KOX_18960	acACP + 6 malACP + 12 nadph -> 12 nadp + c140ACP + 6 co2 + 6 ACP
FABC141	Fatty acid biosynthesis	Fatty acid biosynthesis (tetradecanoic acid; c14:1)	2.3.1.41 AND 2.3.1.179 AND 2.3.1.180 AND 1.1.1.100 AND 4.2.1.- AND 1.3.1.9 AND 1.3.1.-	KOX_05025/KOX_26605/KOX_00905/KOX_05040/KOX_17300/KOX_17280/KOX_00890/KOX_02015(fabG)/KOX_05035(fabG)/KOX_16425/KOX_17290(fabG)/KOX_11500(fabZ)/KOX_18475/KOX_18960	acACP + 6 malACP + 11 nadph -> 11 nadp + c141ACP + 6 co2 + 6 ACP
FABC150	Fatty acid biosynthesis	Fatty acid biosynthesis (pentadecanoic acid; c15:0)	2.3.1.41 AND 2.3.1.179 AND 2.3.1.180 AND 1.1.1.100 AND 4.2.1.- AND 1.3.1.9 AND 1.3.1.-	KOX_05025/KOX_26605/KOX_00905/KOX_05040/KOX_17300/KOX_17280/KOX_00890/KOX_02015(fabG)/KOX_05035(fabG)/KOX_16425/KOX_17290(fabG)/KOX_11500(fabZ)/KOX_18475/KOX_18960	ppacp + 6 malACP + 12 nadph -> 12 nadp + 6 co2 + 6 ACP + c150ACP
FABC151	Fatty acid biosynthesis	Fatty acid biosynthesis (pentadecanoic acid; c15:1)	2.3.1.41 AND 2.3.1.179 AND 2.3.1.180 AND 1.1.1.100 AND 4.2.1.- AND 1.3.1.9 AND 1.3.1.-	KOX_05025/KOX_26605/KOX_00905/KOX_05040/KOX_17300/KOX_17280/KOX_00890/KOX_02015(fabG)/KOX_05035(fabG)/KOX_16425/KOX_17290(fabG)/KOX_11500(fabZ)/KOX_18475/KOX_18960	ppacp + 6 malACP + 11 nadph -> 11 nadp + 6 co2 + 6 ACP + c151ACP
FABC160	Fatty acid biosynthesis	Fatty acid biosynthesis (hexadecanoic acid; c16:0)	2.3.1.41 AND 2.3.1.179 AND 2.3.1.180 AND 1.1.1.100 AND 4.2.1.- AND 1.3.1.9 AND 1.3.1.-	KOX_05025/KOX_26605/KOX_00905/KOX_05040/KOX_17300/KOX_17280/KOX_00890/KOX_02015(fabG)/KOX_05035(fabG)/KOX_16425/KOX_17290(fabG)/KOX_11500(fabZ)/KOX_18475/KOX_18960	acACP + 7 malACP + 14 nadph -> 14 nadp + c160ACP + 7 co2 + 7 ACP
FABC161	Fatty acid biosynthesis	Fatty acid biosynthesis (hexadecanoic acid; c16:1)	2.3.1.41 AND 2.3.1.179 AND 2.3.1.180 AND 1.1.1.100 AND 4.2.1.- AND 1.3.1.9 AND 1.3.1.-	KOX_05025/KOX_26605/KOX_00905/KOX_05040/KOX_17300/KOX_17280/KOX_00890/KOX_02015(fabG)/KOX_05035(fabG)/KOX_16425/KOX_17290(fabG)/KOX_11500(fabZ)/KOX_18475/KOX_18960	acACP + 7 malACP + 13 nadph -> 13 nadp + c161ACP + 7 co2 + 7 ACP
FABC170	Fatty acid biosynthesis	Fatty acid biosynthesis (heptadecanoic acid; c17:0)	2.3.1.41 AND 2.3.1.179 AND 2.3.1.180 AND 1.1.1.100 AND 4.2.1.- AND 1.3.1.9 AND 1.3.1.-	KOX_05025/KOX_26605/KOX_00905/KOX_05040/KOX_17300/KOX_17280/KOX_00890/KOX_02015(fabG)/KOX_05035(fabG)/KOX_16425/KOX_17290(fabG)/KOX_11500(fabZ)/KOX_18475/KOX_18960	ppacp + 7 malACP + 14 nadph -> 14 nadp + 7 co2 + 7 ACP + c170ACP
FABC171	Fatty acid biosynthesis	Fatty acid biosynthesis (heptadecanoic acid; c17:1)	2.3.1.41 AND 2.3.1.179 AND 2.3.1.180 AND 1.1.1.100 AND 4.2.1.- AND 1.3.1.9 AND 1.3.1.-	KOX_05025/KOX_26605/KOX_00905/KOX_05040/KOX_17300/KOX_17280/KOX_00890/KOX_02015(fabG)/KOX_05035(fabG)/KOX_16425/KOX_17290(fabG)/KOX_11500(fabZ)/KOX_18475/KOX_18960	ppacp + 7 malACP + 13 nadph -> 13 nadp + 7 co2 + 7 ACP + c171ACP

FABC180	Fatty acid biosynthesis	Fatty acid biosynthesis (octadecanoic acid; c18:0)	2.3.1.41 AND 2.3.1.179 AND 2.3.1.180 AND 1.1.1.100 AND 4.2.1.- AND 1.3.1.9 AND 1.3.1.-	KOX_05025/KOX_26605/KOX_00905/KOX_05040/KOX_17300/KOX_17280/KOX_00890/KOX_02015(fabG)/KOX_05035(fabG)/KOX_16425/KOX_17290(fabG)/KOX_11500(fabZ)/KOX_18475/KOX_18960	acACP + 8 malACP + 16 nadph -> 16 nadp + c180ACP + 8 co2 + 8 ACP
FABC181	Fatty acid biosynthesis	Fatty acid biosynthesis (octadecanoic acid; c18:1)	2.3.1.41 AND 2.3.1.179 AND 2.3.1.180 AND 1.1.1.100 AND 4.2.1.- AND 1.3.1.9 AND 1.3.1.-	KOX_05025/KOX_26605/KOX_00905/KOX_05040/KOX_17300/KOX_17280/KOX_00890/KOX_02015(fabG)/KOX_05035(fabG)/KOX_16425/KOX_17290(fabG)/KOX_11500(fabZ)/KOX_18475/KOX_18960	acACP + 8 malACP + 15 nadph -> 15 nadp + c181ACP + 8 co2 + 8 ACP
FABC190	Fatty acid biosynthesis	Fatty acid biosynthesis (nonadecanoic acid; c19:0)	2.3.1.41 AND 2.3.1.179 AND 2.3.1.180 AND 1.1.1.100 AND 4.2.1.- AND 1.3.1.9 AND 1.3.1.-	KOX_05025/KOX_26605/KOX_00905/KOX_05040/KOX_17300/KOX_17280/KOX_00890/KOX_02015(fabG)/KOX_05035(fabG)/KOX_16425/KOX_17290(fabG)/KOX_11500(fabZ)/KOX_18475/KOX_18960	ppacp + 8 malACP + 16 nadph -> 16 nadp + 8 co2 + 8 ACP + c190ACP
MALADC	Fatty acid biosynthesis	Malonyl-ACP decarboxylase	4.1.1.87		malACP -> acACP + co2
ACCOAT	Fatty acid biosynthesis	Acetyl-CoA ACP transacylase	2.3.1.180	KOX_17280	ACP + accoa <-> acACP + coa
PPCOAT	Fatty acid biosynthesis	Propionyl-CoA ACP transacylase	2.3.1.180	KOX_17280	ACP + ppcoa <-> coa + ppacp
MALCOAT	Fatty acid biosynthesis	Malonyl-CoA-ACP transacylase	2.3.1.39	KOX_17285/KOX_21675	ACP + malcoa <-> coa + malACP
BKACPS1	Fatty acid biosynthesis	beta-ketoacyl-ACP synthase	2.3.1.41/2.3.1.179/2.3.1.180	KOX_05025/KOX_26605/KOX_00905/KOX_05040/KOX_17300/KOX_17280290(fabG)/KOX_11500(fabZ)/KOX_18475/KOX_18960	acACP + malACP -> ACP + actACP + co2
ACCOACB	Fatty acid biosynthesis	acetyl-CoA carboxylase	6.4.1.2	KOX_04110/KOX_04115/KOX_11525/KOX_26570	accoa + atp + hco3 -> adp + malcoa + pi
BITCB	Fatty acid biosynthesis	biotin carboxylase	6.3.4.14	KOX_04115	accoa + atp + hco3 -> adp + malcoa + pi
BKACPS2	Fatty acid biosynthesis	beta-ketoacyl-ACP synthase	2.3.1.41/2.3.1.179/2.3.1.180	KOX_05025/KOX_26605/KOX_00905/KOX_05040/KOX_17300/KOX_17280290(fabG)/KOX_11500(fabZ)/KOX_18475/KOX_18960	accoa + malACP -> actACP + co2 + coa
UDPGALPF	Fatty acid biosynthesis	UDP-D-galactopyranose furanomutase	5.4.99.9	KOX_25085	udpgal -> udpgalfur
UAGUPAGP	Fatty acid biosynthesis	UDP-N-acetyl-D-glucosamine:undecaprenyl-phosphate N-acetyl-D-glucosamine phosphotransferase	2.7.8.33	KOX_07555	udpnag + udcpp -> ump + acglcmpdp
FAMC120	Fatty acid metabolism	Fatty acid metabolism (dodecanoic acid; c12:0)	6.2.1.3 AND 1.3.99.- AND 4.2.1.17 AND 1.1.1.35 AND 2.3.1.16	KOX_23560/KOX_11675(fadE)/KOX_07835(fadB)/KOX_19445/KOX_26655(fadJ)/KOX_07835(fadB)/KOX_26655(fadJ)/KOX_07830(fadA)/KOX_26660(fadI)	c120 + 6 coa + 5 fad + 5 nad + atp -> 6 accoa + 5 fadh2 + 5 nadh + amp + ppi
FAMC130	Fatty acid metabolism	Fatty acid metabolism (tridecanoic acid; c13:0)	6.2.1.3 AND 1.3.99.- AND 4.2.1.17 AND 1.1.1.35 AND 2.3.1.16	KOX_23560/KOX_11675(fadE)/KOX_07835(fadB)/KOX_19445/KOX_26655(fadJ)/KOX_07835(fadB)/KOX_26655(fadJ)/KOX_07830(fadA)/KOX_26660(fadI)	c130 + 6 coa + 5 fad + 5 nad + atp -> 5 accoa + ppcoa + 5 fadh2 + 5 nadh + amp + ppi
FAMC140	Fatty acid metabolism	Fatty acid metabolism (tetradecanoic acid; c14:0)	6.2.1.3 AND 1.3.99.- AND 4.2.1.17 AND 1.1.1.35 AND 2.3.1.16	KOX_23560/KOX_11675(fadE)/KOX_07835(fadB)/KOX_19445/KOX_26655(fadJ)/KOX_07835(fadB)/KOX_26655(fadJ)/KOX_07830(fadA)/KOX_26660(fadI)	c140 + 7 coa + 6 fad + 6 nad + atp -> 7 accoa + 6 fadh2 + 6 nadh + amp + ppi
FAMC141	Fatty acid metabolism	Fatty acid metabolism (tetradecanoic acid; c14:1)	6.2.1.3 AND 1.3.99.- AND 4.2.1.17 AND 1.1.1.35 AND 2.3.1.16	KOX_23560/KOX_11675(fadE)/KOX_07835(fadB)/KOX_19445/KOX_26655(fadJ)/KOX_07835(fadB)/KOX_26655(fadJ)/KOX_07830(fadA)/KOX_26660(fadI)	c141 + 7 coa + 6 fad + 6 nad + atp -> 7 accoa + 6 fadh2 + 6 nadh + amp + ppi
FAMC150	Fatty acid metabolism	Fatty acid metabolism (pentadecanoic acid; c15:0)	6.2.1.3 AND 1.3.99.- AND 4.2.1.17 AND 1.1.1.35 AND 2.3.1.16	KOX_23560/KOX_11675(fadE)/KOX_07835(fadB)/KOX_19445/KOX_26655(fadJ)/KOX_07835(fadB)/KOX_26655(fadJ)/KOX_07830(fadA)/KOX_26660(fadI)	c150 + 7 coa + 6 fad + 6 nad + atp -> 6 accoa + ppcoa + 6 fadh2 + 6 nadh + amp + ppi
FAMC151	Fatty acid metabolism	Fatty acid metabolism (pentadecanoic acid; c15:1)	6.2.1.3 AND 1.3.99.- AND 4.2.1.17 AND 1.1.1.35 AND 2.3.1.16	KOX_23560/KOX_11675(fadE)/KOX_07835(fadB)/KOX_19445/KOX_26655(fadJ)/KOX_07835(fadB)/KOX_26655(fadJ)/KOX_07830(fadA)/KOX_26660(fadI)	c151 + 7 coa + 6 fad + 6 nad + atp -> 6 accoa + ppcoa + 6 fadh2 + 6 nadh + amp + ppi
FAMC160	Fatty acid metabolism	Fatty acid metabolism (hexadecanoic acid; c16:0)	6.2.1.3 AND 1.3.99.- AND 4.2.1.17 AND 1.1.1.35 AND 2.3.1.16	KOX_23560/KOX_11675(fadE)/KOX_07835(fadB)/KOX_19445/KOX_26655(fadJ)/KOX_07835(fadB)/KOX_26655(fadJ)/KOX_07830(fadA)/KOX_26660(fadI)	c160 + 8 coa + 7 fad + 7 nad + atp -> 8 accoa + 7 fadh2 + 7 nadh + amp + ppi
FAMC161	Fatty acid metabolism	Fatty acid metabolism (hexadecanoic acid; c16:1)	6.2.1.3 AND 1.3.99.- AND 4.2.1.17 AND 1.1.1.35 AND 2.3.1.16	KOX_23560/KOX_11675(fadE)/KOX_07835(fadB)/KOX_19445/KOX_26655(fadJ)/KOX_07835(fadB)/KOX_26655(fadJ)/KOX_07830(fadA)/KOX_26660(fadI)	c161 + 8 coa + 7 fad + 7 nad + atp -> 8 accoa + 7 fadh2 + 7 nadh + amp + ppi
FAMC170	Fatty acid metabolism	Fatty acid metabolism (heptadecanoic acid; c17:0)	6.2.1.3 AND 1.3.99.- AND 4.2.1.17 AND 1.1.1.35 AND 2.3.1.16	KOX_23560/KOX_11675(fadE)/KOX_07835(fadB)/KOX_19445/KOX_26655(fadJ)/KOX_07835(fadB)/KOX_26655(fadJ)/KOX_07830(fadA)/KOX_26660(fadI)	c170 + 8 coa + 7 fad + 7 nad + atp -> 7 accoa + ppcoa + 7 fadh2 + 7 nadh + amp + ppi
FAMC171	Fatty acid metabolism	Fatty acid metabolism (heptadecanoic acid; c17:1)	6.2.1.3 AND 1.3.99.- AND 4.2.1.17 AND 1.1.1.35 AND 2.3.1.16	KOX_23560/KOX_11675(fadE)/KOX_07835(fadB)/KOX_19445/KOX_26655(fadJ)/KOX_07835(fadB)/KOX_26655(fadJ)/KOX_07830(fadA)/KOX_26660(fadI)	c171 + 8 coa + 7 fad + 7 nad + atp -> 7 accoa + ppcoa + 7 fadh2 + 7 nadh + amp + ppi
FAMC180	Fatty acid metabolism	Fatty acid metabolism (octadecanoic acid; c18:0)	6.2.1.3 AND 1.3.99.- AND 4.2.1.17 AND 1.1.1.35 AND 2.3.1.16	KOX_23560/KOX_11675(fadE)/KOX_07835(fadB)/KOX_19445/KOX_26655(fadJ)/KOX_07835(fadB)/KOX_26655(fadJ)/KOX_07830(fadA)/KOX_26660(fadI)	c180 + 9 coa + 8 fad + 8 nad + atp -> 9 accoa + 8 fadh2 + 8 nadh + amp + ppi
FAMC181	Fatty acid metabolism	Fatty acid metabolism (octadecanoic acid; c18:1)	6.2.1.3 AND 1.3.99.- AND 4.2.1.17 AND 1.1.1.35 AND 2.3.1.16	KOX_23560/KOX_11675(fadE)/KOX_07835(fadB)/KOX_19445/KOX_26655(fadJ)/KOX_07835(fadB)/KOX_26655(fadJ)/KOX_07830(fadA)/KOX_26660(fadI)	c181 + 9 coa + 8 fad + 8 nad + atp -> 9 accoa + 8 fadh2 + 8 nadh + amp + ppi
FAMC190	Fatty acid metabolism	Fatty acid metabolism (nonadecanoic acid; c19:0)	6.2.1.3 AND 1.3.99.- AND 4.2.1.17 AND 1.1.1.35 AND 2.3.1.16	KOX_23560/KOX_11675(fadE)/KOX_07835(fadB)/KOX_19445/KOX_26655(fadJ)/KOX_07835(fadB)/KOX_26655(fadJ)/KOX_07830(fadA)/KOX_26660(fadI)	c190 + 9 coa + 8 fad + 8 nad + atp -> 8 accoa + ppcoa + 8 fadh2 + 8 nadh + amp + ppi
FAOb	Fatty acid metabolism	fatty acid oxidation (Butanoyl-CoA)			c040coa + fad + nad -> aacoa + fadh2 + nadh
AACPS1	Fatty acid metabolism	acyl-[acyl-carrier-protein] synthetase (n-C12:0)	6.2.1.20	KOX_01650	ACP + atp + c120 -> amp + c120ACP + ppi
AACPS2	Fatty acid metabolism	acyl-[acyl-carrier-protein] synthetase (n-C14:0)	6.2.1.20	KOX_01650	ACP + atp + c140 -> amp + c140ACP + ppi
AACPS3	Fatty acid metabolism	acyl-[acyl-carrier-protein] synthetase (n-C14:1)	6.2.1.20	KOX_01650	ACP + atp + c141 -> amp + c141ACP + ppi
AACPS4	Fatty acid metabolism	acyl-[acyl-carrier-protein] synthetase (n-C15:0)	6.2.1.20	KOX_01650	ACP + atp + c150 -> amp + c150ACP + ppi
AACPS5	Fatty acid metabolism	acyl-[acyl-carrier-protein] synthetase (n-C15:1)	6.2.1.20	KOX_01650	ACP + atp + c151 -> amp + c151ACP + ppi
AACPS6	Fatty acid metabolism	acyl-[acyl-carrier-protein] synthetase (n-C16:0)	6.2.1.20	KOX_01650	ACP + atp + c160 -> amp + c160ACP + ppi
AACPS7	Fatty acid metabolism	acyl-[acyl-carrier-protein] synthetase (n-C16:1)	6.2.1.20	KOX_01650	ACP + atp + c161 -> amp + c161ACP + ppi
AACPS8	Fatty acid metabolism	acyl-[acyl-carrier-protein] synthetase (n-C17:0)	6.2.1.20	KOX_01650	ACP + atp + c170 -> amp + c170ACP + ppi
AACPS9	Fatty acid metabolism	acyl-[acyl-carrier-protein] synthetase (n-C17:1)	6.2.1.20	KOX_01650	ACP + atp + c171 -> amp + c171ACP + ppi
AACPS10	Fatty acid metabolism	acyl-[acyl-carrier-protein] synthetase (n-C18:0)	6.2.1.20	KOX_01650	ACP + atp + c180 -> amp + c180ACP + ppi
AACPS11	Fatty acid metabolism	acyl-[acyl-carrier-protein] synthetase (n-C18:1)	6.2.1.20	KOX_01650	ACP + atp + c181 -> amp + c181ACP + ppi
AACPS12	Fatty acid metabolism	acyl-[acyl-carrier-protein] synthetase (n-C19:0)	6.2.1.20	KOX_01650	ACP + atp + c190 -> amp + c190ACP + ppi
AACPS13	Fatty acid metabolism	acyl-[acyl-carrier-protein] synthetase (n-C13:0)	6.2.1.20	KOX_01650	ACP + atp + c130 -> amp + c130ACP + ppi
HACOAD1	Fatty acid metabolism	3-hydroxyacyl-CoA dehydrogenase (acetoacetyl-CoA)	1.1.1.35/1.1.1.157	KOX_07835(fadB)/KOX_26655(fadJ)/KOX_19455	3hbcoa + nad -> aacoa + nadh
ACOADH1	Fatty acid metabolism	acyl-CoA dehydrogenase (butanoyl-CoA)	1.3.99.-	KOX_11675(fadE)	c040coa + fad <-> ccoa + fadh2
ACCOAAT1	Fatty acid metabolism	acetyl-CoA C-acetyltransferase	2.3.1.9	KOX_01800/KOX_02020/KOX_02110/	2 accoa <-> aacoa + coa
ENCOAH1	Fatty acid metabolism	enoyl-CoA hydratase	4.2.1.17	KOX_07835(fadB)/KOX_19445/KOX_26655(fadJ)/	3hbcoa <-> ccoa
3HBCOADH	Fatty acid metabolism	3-hydroxyacyl-CoA dehydrogenase / enoyl-CoA hydratase / 3-hydroxybutyryl-CoA epimerase / enoyl-CoA isomerase	5.1.2.3	KOX_07835(fadB)/KOX_26655(fadJ)/	3hbcoa <-> r3hbcoa
CMBLD6	Fluorobenzoate degradation	carboxymethylenebutenolidase	3.1.1.45	KOX_02855/KOX_07760	4fmuclac -> 2mac + hf
CMBLD7	Fluorobenzoate degradation	carboxymethylenebutenolidase	3.1.1.45	KOX_02855/KOX_07760	5fmuclac -> 2mac + hf
35DB4HBH	Fluorobenzoate degradation	3,5-dibromo-4-hydroxybenzamide hydro-lyase (nitrile-forming)	4.2.1.84	KOX_20500/KOX_20505	bromox -> 35dbr4hb
DHFR1	Folate Biosynthesis	dihydrofolate reductase	1.5.1.3	KOX_10680(folA)/KOX_21840	dhf + nadh <-> nad + thf
DHFR1p	Folate Biosynthesis	dihydrofolate reductase	1.5.1.3	KOX_10680(folA)/KOX_21840	dhf + nadph <-> nadp + thf
DHPS2	Folate Biosynthesis	dihydropteroate synthase	2.5.1.15	KOX_03690(folP)	paba + ahhmd -> dhpt + ppi
GTPCHI	Folate Biosynthesis	GTP cyclohydrolase I	3.5.4.16	KOX_25750(folE)	gtp -> ahdt + formate
FPGLUS1	Folate Biosynthesis	folylpolyglutamate synthase	6.3.2.17/6.3.2.12	KOX_26565	atp + dhpt + glu -> adp + dhf + pi

ABZS	Folate Biosynthesis	4-aminobenzoate synthase	4.1.3.38	KOX_17305	adchor -> paba + pyr
ADCMS	Folate Biosynthesis	4-amino-4-deoxychorismate synthase	2.6.1.85	KOX_04530/KOX_04530(pabB)	chor + gln -> adchor + glu
DHNPTA	Folate Biosynthesis	dihydroneopterin aldolase	4.1.2.25	KOX_03070(folB)	dhnpt -> ahhmp + glal
HMDPPK	Folate Biosynthesis	6-hydroxymethyl-dihydropterin pyrophosphokinase	2.7.6.3	KOX_11220	ahhmp + atp -> ahhmd + amp
6PYRTP	Folate Biosynthesis	6-pyruvoyl tetrahydrobiopterin synthase	4.2.3.12	KOX_01125	ahdt -> pythp + pppi
AKLPP	Folate Biosynthesis	alkaline phosphatase	3.1.3.1	KOX_12385	ahdt -> dhnpt + 3 pi
DHPS1	Folate Biosynthesis	dihydropteroate synthase	2.5.1.15	KOX_03690(folP)	ahhmp + paba -> dhpt
DHFR2	Folate Biosynthesis	dihydrofolate reductase	1.5.1.3	KOX_10680(folA)/KOX_21840	dhf + nad <-> fl + nadh
DHFR3	Folate Biosynthesis	dihydrofolate reductase	1.5.1.3	KOX_10680(folA)/KOX_21840	fl + 2 nadh -> thf + 2 nad
FPGLUS2	Folate Biosynthesis	folylpolyglutamate synthase	6.3.2.17	KOX_26565	atp + thf + glu <-> adp + pi + thfglu
DHFR2p	Folate Biosynthesis	dihydrofolate reductase	1.5.1.3	KOX_10680(folA)/KOX_21840	dhf + nadp <-> fl + nadph
DHFR3p	Folate Biosynthesis	dihydrofolate reductase	1.5.1.3	KOX_10680(folA)/KOX_21840	fl + 2 nadph -> thf + 2 nadp
METTHFD	Folate Metabolism	methylenetetrahydrofolate dehydrogenase (NADP)	1.5.1.5	KOX_13225	metthf + nadp <-> methf + nadph
FTHFDF	Folate Metabolism	formyltetrahydrofolate deformylase	3.5.1.10	KOX_23060(purU)	fthf -> formate + thf
GLYAMT	Folate Metabolism	aminomethyltransferase	2.1.2.10	KOX_02485	gly + nad + thf -> co2 + metthf + nadh + nh4
METTHFR	Folate Metabolism	5,10-methylenetetrahydrofolate reductase (NADH)	1.5.1.20	KOX_07330(metF)	metthf + nadh -> mthf + nad
METTHFRp	Folate Metabolism	5,10-methylenetetrahydrofolate reductase (NADH)	1.5.1.20	KOX_07330(metF)	metthf + nadph -> mthf + nadp
FRUK	Fructose and Mannose metabolism	fructokinase	2.7.1.4	KOX_00775/KOX_06115/KOX_12435/KOX_13375/ KOX_20965/KOX_27450	atp + fru -> adp + f6p
MAN6PI	Fructose and Mannose metabolism	mannose-6-phosphate isomerase	5.3.1.8	KOX_21900	man6p <-> f6p
SBT6PD	Fructose and Mannose metabolism	sorbitol-6-phosphate dehydrogenase	1.1.1.140	KOX_00590/KOX_08120	sbt6p + nad <-> f6p + nadh
MNT1PD	Fructose and Mannose metabolism	mannitol-1-phosphate 5-dehydrogenase	1.1.1.17	KOX_05765	mnt1p + nad <-> f6p + nadh
RMNI	Fructose and Mannose metabolism	L-rhamnose isomerase	5.3.1.14	KOX_07205	rmn <-> rml
RMLK	Fructose and Mannose metabolism	rhamnulokinase	2.7.1.5	KOX_07210(rhaB)	rml + atp -> rml1p + adp
RM1PA	Fructose and Mannose metabolism	rhamnulose-1-phosphate aldolase	4.1.2.19	KOX_07200	rml1p <-> dhap + llald
F1PK	Fructose and Mannose metabolism	6-phosphofructokinase	2.7.1.56	KOX_03500/KOX_25815(fruK)	f1p + atp <-> fdp + adp
FUCI	Fructose and Mannose metabolism	L-fucose isomerase	5.3.1.25	KOX_01280(fucI)	fuc <-> fucul
FCL1PA	Fructose and Mannose metabolism	L-fuculose phosphate aldolase	4.1.2.17	KOX_01270	fucul1p -> dhap + llald
2K3DRMNA	Fructose and Mannose metabolism	2-keto-3-deoxy-L-rhamnonate aldolase	4.1.2.-	KOX_26185	llald + pyr <-> 2d3drmn
RMNND	Fructose and Mannose metabolism	L-rhamnonate dehydratase	4.2.1.90	KOX_26195	2d3drmn <-> rmnn
FUCLK	Fructose and Mannose metabolism	L-fuculokinase	2.7.1.51	KOX_01285	fucul + atp -> fucul1p + adp
UDPG4E	Galactose metabolism	UDPglucose 4-epimerase	5.1.3.2	KOX_14785	udpg <-> udpgal
GALT1PD	Galactose metabolism	Galactitol-1-phosphate dehydrogenase	1.1.1.251	KOX_03535	galt1p + nad <-> nadh + t6p
GALCTND	Galactose metabolism	galactonate dehydratase	4.2.1.6	KOX_06525	dgalctn -> 2d3dgalctn
2D3DOXGALK	Galactose metabolism	2-dehydro-3-deoxygalactonokinase	2.7.1.58	KOX_06535	2d3dgalctn + atp -> 2d3dgalctn6p + adp
2D3D6PGA	Galactose metabolism	2-dehydro-3-deoxy-6-phosphogalactonate aldolase	4.1.2.21	KOX_06530/KOX_10095	2d3dgalctn6p -> g3p + pyr
TAG16PA	Galactose metabolism	tagatose 1,6-diphosphate aldolase	4.1.2.40	KOX_03495(gatY)	dhap + g3p <-> t16p
PFK2	Galactose metabolism	phosphofructokinase	2.7.1.11/2.7.1.144	KOX_06965/KOX_07260/KOX_22945/KOX_03515	t6p + atp <-> t16p + adp
GLACK	Galactose metabolism	galactokinase	2.7.1.6	KOX_14775	glac + atp -> glac1p + adp
UDPGHPU	Galactose metabolism	UDPglucose-hexose-1-phosphate uridylyltransferase	2.7.7.12	KOX_14780	glac1p + udpg <-> g1p + udpgal
GALCTS	Galactose metabolism	beta-D-galactosidase	3.2.1.23	KOX_03235(edgA)/KOX_03240(ebgC)	lactose -> glac + glc
GALACTAN	Galactose metabolism	beta-D-galactosidase	3.2.1.23	KOX_09055/KOX_12915	galactan -> galactan_1 + glac
AGALCTS1	Galactose metabolism	alpha-galactosidase	3.2.1.22	KOX_07045/KOX_16445	stachyose -> raffinose + glac
AGALCTS2	Galactose metabolism	alpha-galactosidase	3.2.1.22	KOX_07045/KOX_16445	galactinol <-> mi + glac
AGALCTS3	Galactose metabolism	alpha-galactosidase	3.2.1.22	KOX_07045/KOX_16445	melibt <-> sot + glac
AGALCTS4	Galactose metabolism	alpha-galactosidase	3.2.1.22	KOX_07045/KOX_16445	epimelib <-> man + glac
AGALCTS5	Galactose metabolism	alpha-galactosidase	3.2.1.22	KOX_07045/KOX_16445	glacgly <-> gl + glac
AGALCTS6	Galactose metabolism	alpha-galactosidase	3.2.1.22	KOX_07045/KOX_16445	meli <-> glac + glc
AGALCTS7	Galactose metabolism	alpha-galactosidase	3.2.1.22	KOX_07045/KOX_16445	mannot <-> meli + glac
AGALCTS8	Galactose metabolism	alpha-galactosidase	3.2.1.22	KOX_07045/KOX_16445	raffinose <-> suc + glac
FRUFUS1	Galactose metabolism	beta-fructofuranosidase	3.2.1.26	KOX_01765/KOX_07150/KOX_13360	stachyose -> mannot + fru
FRUFUS2	Galactose metabolism	beta-fructofuranosidase	3.2.1.26	KOX_01765/KOX_07150/KOX_13360	raffinose -> meli + fru
GLUCSD1	Galactose metabolism	alpha-glucosidase	3.2.1.20	KOX_12475/KOX_15200	suc -> glc + fru
GLUCSD2	Starch and sucrose metabolism	beta-fructofuranosidase	3.2.1.26	KOX_01765/KOX_07150/KOX_13360	suc -> glc + fru
CMBLD1	Chlorocyclohexane and chlorobenzene degradation	carboxymethylenebutenolidase	3.1.1.45	KOX_02855/KOX_07760	dchrocmo -> dchrooe
CMBLD2	Chlorocyclohexane and chlorobenzene degradation	carboxymethylenebutenolidase	3.1.1.45	KOX_02855/KOX_07760	tcmbo -> 2mac
4NPHP1	gamma-Hexachlorocyclohexane degradation	4-nitrophenyl phosphatase	3.1.3.2	KOX_08315(aphA)/KOX_14030/KOX_18070	ntphp -> pnp + pi
4NPHP2	gamma-Hexachlorocyclohexane degradation	4-nitrophenyl phosphatase	3.1.3.1	KOX_12385	ntphp -> pnp + pi
ACCOAA2	Geraniol degrdation	acetyl-CoA acyltransferase	2.3.1.16	KOX_07830(fadA)/KOX_26660(fadI)	7m3o6ocoa + coa -> 5mh4ecoa + accoa
ACCOAA3	Geraniol degrdation	acetyl-CoA acyltransferase	2.3.1.16	KOX_07830(fadA)/KOX_26660(fadI)	5m3o4hcoa + coa -> 3mccoa + accoa
SUCSD2	Glutamate metabolism	succinate-semialdehyde dehydrogenase (NAD)	1.2.1.16	KOX_09980/KOX_11825(gabD)/KOX_24465	nad + sucsal <-> nadh + succ
GABAT1	Glutamate metabolism	4-aminobutyrate transaminase	2.6.1.19	KOX_04875	gaba + akg -> glu + sucsal
CABPS	Glutamate metabolism	carbamoyl-phosphate synthase (glutamine-hydrolysing)	6.3.5.5	KOX_10655/KOX_10660(carB)	2 atp + gln + hco3 -> 2 adp + cap + glu + pi
GLUR	Glutamate metabolism	glutamate racemase	5.1.1.3	KOX_07425	dglu <-> glu
GGLUCYSS	Glutamate metabolism	gamma-glutamylcysteine synthetase	6.3.2.2	KOX_00505	atp + cys + glu -> adp + gcys + pi
GTHRDS	Glutamate metabolism	glutathione synthetase	6.3.2.3	KOX_02675	atp + gcys + gly -> adp + rgt + pi
GLUS1	Glutamate metabolism	glutamate synthase	1.4.1.13	KOX_03860(gltB)/KOX_03865(gltD)	akg + gln + nadph -> 2 glu + nadp
GLUS2	Glutamate metabolism	glutamate synthase	1.4.1.14	KOX_03860(gltB)/KOX_03865(gltD)	akg + gln + nadh -> 2 glu + nad
GLUDH2	Glutamate Metabolism	glutamate dehydrogenase	1.4.1.4	KOX_18085	glu + nadp <-> akg + nadph + nh4
GLUDH1	Glutamate Metabolism	glutamate dehydrogenase	1.4.1.3	KOX_19525	glu + nad <-> akg + nadh + nh4
GLUN1	Glutamate Metabolism	glutaminase	3.5.1.2	KOX_14645	gln -> glu + nh4
GLNST1	Glutamate metabolism	glutamine synthetase	6.3.1.2	KOX_06895(glnA)	atp + glu + nh4 -> adp + gln + pi
GABAT2	Glutamate Metabolism	4-aminobutyrate transaminase	2.6.1.19	KOX_04875	gaba + akg <-> glu + sucsal
GTADT1	Aminoacyl-tRNA biosynthesis	aspartyl-tRNA(Asn)/glutamyl-tRNA (Gln) amidotransferase	6.3.5.7	KOX_16785	glutrna + gln + atp -> glntrna + glu + pi + adp
MAAMPT	Glutathione Metabolism	membrane alanyl aminopeptidase	3.4.11.2	KOX_16115(pepN)	progly -> gly + pro
GTSPMDS	Glutathione Metabolism	glutathionylspermidine synthetase	6.3.1.8	KOX_02835	atp + rgt + sprmd -> adp + gtspmd + pi
GTHHR	Glutathione Metabolism	glutathione hydralase	2.3.2.2	KOX_04780(ggt)/KOX_16795	rgt -> cysgly + glu
ALAAPT	Glutathione Metabolism	alanyl aminopeptidase	3.4.11.1/3.4.11.2/3.4 .11.23/3.4.13.-	KOX_09335/KOX_21055/KOX_16115(pepN)/KOX_ 27325/KOX_11745	cysgly -> cys + gly
GTTPX	Glutathione Metabolism	glutathione peroxidase	1.11.1.9	KOX_21870/KOX_22885(btuE)	h2o2 + 2 rgt <-> ogt
GLUTHR	Glutathione Metabolism	glutathione reductase	1.8.1.7	KOX_05295	ogt + nadph -> 2 rgt + nadp
GTSPMDAS	Glutathione Metabolism	glutathionylspermidine amidase/synthetase	3.5.1.78	KOX_02835	gtspmd -> rgt + sprmd
GLYCK	Glycerolipid Metabolism	glycerol kinase	2.7.1.30	KOX_06075/KOX_13865	atp + gl -> adp + glyc3p
ALDCg	Glycerolipid Metabolism	alcohol dehydrogenase (glycerol)	1.1.1.1	KOX_19595/KOX_20090/KOX_23025	t3 + nad <-> gl + nadh
DHACK	Glycerolipid Metabolism	dihydroxyacetone kinase	2.7.1.29	KOX_03170/KOX_03175	glyn + atp -> dhap + adp
GLYDH	Glycerolipid Metabolism	glycerol dehydrogenase	1.1.1.6	KOX_03160/KOX_07340(gldA)/KOX_27465(gldA)	gl + nad <-> glyn + nadh
GLDHT	Glycerolipid Metabolism	glycerol dehydratase	4.2.1.30		gl -> 3hprop
13PDODH	Glycerolipid Metabolism	1,3-propanediol dehydrogenase	1.1.1.202		3hprop + nadh -> 13pdo + nad
PROPD	Glycerolipid Metabolism	propanediol dehydratase	4.2.1.28	KOX_01455(pduC)/KOX_01460(pduD)/KOX_0146 5(pduE)	12ppd-R -> propanal
PROPCOA	Glycerolipid Metabolism	propanal:NAD+ oxidoreductase (CoA-acylating)		KOX_01510	propanal + nad + coa -> ppcoa + nadh
PROPDH	Glycerolipid Metabolism	1-propanol dehydrogenase	1.1.1.202	KOX_01515	propanal + nadh -> 1propanol + nad
PGT	Glycerolipid Metabolism	phosphoglycerol transferase	2.7.8.20	KOX_10175	pg + dgdg -> dgr + glypgg
AGALCTS9	Glycerolipid Metabolism	alpha-galactosidase	3.2.1.22	KOX_07045/KOX_16445	dgaldg -> mgdg + glac
GL3PD	Glycerophospholipid Metabolism	glycerol-3-phosphate dehydrogenase (NAD)	1.1.1.94	KOX_05810(gpsA)	glyc3p + nad <-> dhap + nadh
GL3PDp	Glycerophospholipid Metabolism	glycerol-3-phosphate dehydrogenase (NADP)	1.1.1.94	KOX_05810(gpsA)	glyc3p + nadp <-> dhap + nadph
ETHAML	Glycerophospholipid Metabolism	Ethanolamine ammonia-lyase	4.3.1.7	KOX_12225/KOX_12230/KOX_26955/KOX_26960/ KOX_26965(eutA)	etha <-> acal + nh4
G3POAT	Glycerophospholipid Metabolism	glycerol-3-phosphate O-acyltransferase	2.3.1.15	KOX_03075/KOX_03075/KOX_17275	glyc3p + 0.06 c120ACP + 0.003 c130ACP + 0.144 c140ACP + 0.005 c151ACP + 0.378 c160ACP + 0.061 c161ACP + 0.005 c180ACP + 0.053 c181ACP + 0.256 c170ACP + 0.001 c171ACP + 0.034 c190ACP -> agl3p + ACP
AG3POAT	Glycerophospholipid Metabolism	1-acylglycerol-3-phosphate O-acyltransferase	2.3.1.51	KOX_02915	agl3p + 0.06 c120ACP + 0.003 c130ACP + 0.144 c140ACP + 0.005 c151ACP + 0.378 c160ACP + 0.061 c161ACP + 0.005 c180ACP + 0.053 c181ACP + 0.256 c170ACP + 0.001 c171ACP + 0.034 c190ACP -> pa + ACP
DGRK	Glycerophospholipid Metabolism	diacylglycerol kinase	2.7.1.107	KOX_08255	dgr + atp -> adp + pa

PPTCT	Glycerophospholipid Metabolism	phosphatidate cytidylyltransferase	2.7.7.41	KOX_11475(cdsA)	pa + ctp -> cdpdg + ppi
PPGS	Glycerophospholipid Metabolism	Phosphatidylglycerol synthase	2.7.8.5	KOX_24100	cdpdg + glyc3p -> cmp + pgp
PPSERS	Glycerophospholipid Metabolism	Phosphatidylserine syntase	2.7.8.8	KOX_27630(pssA)	cdpdg + ser -> cmp + ps
PLIPASA1C	Glycerophospholipid Metabolism	Phospholipase A1 (phosphatidylcholine)	3.1.1.32	KOX_07705	pc -> 2ag3pc + 0.06 c120 + 0.003 c130 + 0.144 c140 + 0.005 c151 + 0.378 c160 + 0.061 c161 + 0.256 c170 + 0.001 c171 + 0.005 c180 + 0.053 c181 + 0.034 c190
PLIPASA1S	Glycerophospholipid Metabolism	Phospholipase A1 (Phosphatidylserine)	3.1.1.32	KOX_07705	ps -> 2ag3ps + 0.06 c120 + 0.003 c130 + 0.144 c140 + 0.005 c151 + 0.378 c160 + 0.061 c161 + 0.256 c170 + 0.001 c171 + 0.005 c180 + 0.053 c181 + 0.034 c190
PLIPASA1E	Glycerophospholipid Metabolism	Phospholipase A1 (phosphatidylethanolamine)	3.1.1.32	KOX_07705	pe -> 2ag3pe + 0.06 c120 + 0.003 c130 + 0.144 c140 + 0.005 c151 + 0.378 c160 + 0.061 c161 + 0.256 c170 + 0.001 c171 + 0.005 c180 + 0.053 c181 + 0.034 c190
PLIPASA1g	Glycerophospholipid Metabolism	Phospholipase A1 (phosphatidylglycerol)	3.1.1.32	KOX_07705	pg -> 2ag3pg + 0.06 c120 + 0.003 c130 + 0.144 c140 + 0.005 c151 + 0.378 c160 + 0.061 c161 + 0.256 c170 + 0.001 c171 + 0.005 c180 + 0.053 c181 + 0.034 c190
AGPEACT	Glycerophospholipid Metabolism	2-Acyl-sn-glycero-3-phosphoethanolamine O-acyltransferase	2.3.1.40	KOX_01650	2ag3pe + aACP -> pe + ACP
LPLIPAc	Glycerophospholipid Metabolism	Lysophospholipase L (acyl-glycerophosphocholinel)	3.1.1.5	KOX_07730	2ag3pc -> g3pc + 0.06 c120 + 0.003 c130 + 0.144 c140 + 0.005 c151 + 0.378 c160 + 0.061 c161 + 0.256 c170 + 0.001 c171 + 0.005 c180 + 0.053 c181 + 0.034 c190
LPLIPAe	Glycerophospholipid Metabolism	Lysophospholipase L (acyl-glycerophosphoethanolamine)	3.1.1.5	KOX_07730	2ag3pe -> g3pe + 0.06 c120 + 0.003 c130 + 0.144 c140 + 0.005 c151 + 0.378 c160 + 0.061 c161 + 0.256 c170 + 0.001 c171 + 0.005 c180 + 0.053 c181 + 0.034 c190
LPLIPAs	Glycerophospholipid Metabolism	Lysophospholipase L (acyl-glycerophosphoserine)	3.1.1.5	KOX_07730	2ag3ps -> g3ps + 0.06 c120 + 0.003 c130 + 0.144 c140 + 0.005 c151 + 0.378 c160 + 0.061 c161 + 0.256 c170 + 0.001 c171 + 0.005 c180 + 0.053 c181 + 0.034 c190
LPLIPAg	Glycerophospholipid Metabolism	Lysophospholipase L (acyl-glycerophosphoglycerol)	3.1.1.5	KOX_07730	2ag3pg -> g3pg + 0.06 c120 + 0.003 c130 + 0.144 c140 + 0.005 c151 + 0.378 c160 + 0.061 c161 + 0.256 c170 + 0.001 c171 + 0.005 c180 + 0.053 c181 + 0.034 c190
PGRPP	Glycerophospholipid Metabolism	phosphatidylglycerol phosphate phosphatase	3.1.3.27	KOX_12595/KOX_18425	pgp -> pg + pi
GPPDPD1	Glycerophospholipid Metabolism	Glycerophosphodiester phosphodiesterase (Glycerophosphocholine)	3.1.4.46	KOX_04790(udpQ)/KOX_26160(glpQ)	g3pc -> choline + glyc3p
GPPDPD2	Glycerophospholipid Metabolism	Glycerophosphodiester phosphodiesterase (Glycerophosphoethanolamine)	3.1.4.46	KOX_04790(udpQ)/KOX_26160(glpQ)	g3pe -> etha + glyc3p
GPPDPD3	Glycerophospholipid Metabolism	Glycerophosphodiester phosphodiesterase (Glycerophosphoserine)	3.1.4.46	KOX_04790(udpQ)/KOX_26160(glpQ)	g3ps -> glyc3p + ser
GPPDPD4	Glycerophospholipid Metabolism	Glycerophosphodiester phosphodiesterase (Glycerophosphoglycerol)	3.1.4.46	KOX_04790(udpQ)/KOX_26160(glpQ)	g3pg -> gl + glyc3p
GPPDPD5	Glycerophospholipid Metabolism	Glycerophosphodiester phosphodiesterase (Glycerophosphoinositol)	3.1.4.46	KOX_04790(udpQ)/KOX_26160(glpQ)	g3pi -> glyc3p + mi
CDPDGP	Glycerophospholipid Metabolism	CDP-diacylglycerol pyrophosphatase	3.6.1.26	KOX_06055	cdpdg -> pa + cmp
PSERD	Glycerophospholipid Metabolism	Phosphatidylserine decarboxylase	4.1.1.65	KOX_08795(psd)	ps -> pe + co2
CLPNS1	Glycerophospholipid Metabolism	cardiolipin synthase	2.7.8.-	KOX_15005/KOX_22980(cls)	2 pg <-> clpn + gl
CLPNS2	Glycerophospholipid Metabolism	cardiolipin synthase	2.7.8.-	KOX_15005/KOX_22980(cls)	pg + cdpdg -> clpn + cmp
ETNP	Glycerophospholipid metabolism	ethanolaminephosphotransferase	2.7.8.1		pe + cmp <-> cdpetn + dgr
GALGCER	Glycerophospholipid metabolism	beta-D-galactosidase	3.2.1.23	KOX_21415(lacZ)	bgalgcer -> glucer + glac
AGALCTS10	Glycerophospholipid metabolism	alpha-galactosidase	3.2.1.22	KOX_07045	dgalcer -> galcer + glac
HPYRR	Glycine, Serine and threonine Metabolism	Hydroxypyruvate reductase (NADH)	1.1.1.79	KOX_00275/KOX_17100(ghrA)	hpyr + nadh -> glyc-R + nad
HPYRRp	Glycine, Serine and threonine Metabolism	Hydroxypyruvate reductase (NADPH)	1.1.1.81	KOX_00275/KOX_17100(ghrA)	hpyr + nadph <-> glyc-R + nadp
THRDH	Glycine, Serine and threonine Metabolism	L-threonine dehydrogenase	1.1.1.103	KOX_05850(tdh)	nad + thr -> 2aobut + nadh
PGLCED	Glycine, Serine and threonine Metabolism	D-3-phosphoglycerate dehydrogenase	1.1.1.95	KOX_02525/KOX_05145/KOX_14075/KOX_20995	3pg + nad <-> 3php + nadh
GLYHMT	Glycine, Serine and threonine Metabolism	glycine hydroxymethyltransferase	2.1.2.1	KOX_27430(glyA)	ser + thf -> gly + metthf
GLYCAT	Glycine, Serine and threonine Metabolism	glycine C-acetyltransferase	2.3.1.29	KOX_05855	accoa + gly <-> 2aobut + coa
PSERT	Glycine, Serine and threonine Metabolism	phosphoserine transaminase	2.6.1.52	KOX_15995	3php + glu <-> akg + pser
GLYCEK2	Glycine, Serine and threonine Metabolism	glycerate kinase	2.7.1.31	KOX_01170/KOX_03460	atp + glyc-R <-> 2pg + adp
PSERP	Glycine, Serine and threonine Metabolism	phosphoserine phosphatase (L-serine)	3.1.3.3	KOX_10335(serB)	pser -> pi + ser
SERD	Glycine, Serine and threonine Metabolism	L-serine deaminase	4.3.1.17	KOX_01230/KOX_23635	ser -> nh4 + pyr
DSERD	Glycine, Serine and threonine Metabolism	D-serine dehydratase	4.3.1.18	KOX_06460	dser <-> nh4 + pyr
MNAO1	Glycine, Serine and threonine Metabolism	monoamine oxidase	1.4.3.21	KOX_19410(tynA)	aact + o2 -> h2o2 + mtg + nh4
HSERD	Glycine, Serine and Threonine Metabolism	homoserine dehydrogenase (NADH)	1.1.1.3	KOX_07315(metL)/KOX_10410(thrA)	hser + nad <-> aspsa + nadh
HSERDp	Glycine, Serine and Threonine Metabolism	homoserine dehydrogenase (NADPH)	1.1.1.3	KOX_07315(metL)/KOX_10410(thrA)	hser + nadp <-> aspsa + nadph
ASPSAD	Glycine, Serine and Threonine Metabolism	aspartate-semialdehyde dehydrogenase	1.2.1.11	KOX_04740	aspsa + nadp + pi <-> basp + nadph
HSERK	Glycine, Serine and Threonine Metabolism	homoserine kinase	2.7.1.39	KOX_10415	atp + hser -> adp + phser
ASPK	Glycine, Serine and Threonine Metabolism	aspartate kinase	2.7.2.4	KOX_07315(metL)/KOX_08160/KOX_10410(thrA)	asp + atp <-> basp + adp
THRAD2	Glycine, Serine and Threonine Metabolism	L-allo-threonine aldolase	4.1.2.5	KOX_15770	athr <-> acal + gly
THRAD1	Glycine, Serine and Threonine Metabolism	threonine aldolase	4.1.2.5	KOX_15770	thr <-> acal + gly
THRS	Glycine, Serine and Threonine Metabolism	threonine synthase	4.2.3.1	KOX_10420	phser -> pi + thr
AOBUTDCs	Glycine, Serine and Threonine Metabolism	L-2-amino-3-oxobutanoate decarboxylation (spontaneous)			2aobut -> aact + co2
BETALDDH1	Glycine, Serine and Threonine Metabolism	betaine-aldehyde dehydrogenase	1.2.1.8	KOX_13815	bal + nad -> glyb + nadh
BETALDDH2	Glycine, Serine and Threonine Metabolism	betaine-aldehyde dehydrogenase	1.2.1.8	KOX_13815	bal + nadp -> glyb + nadph
SERDHT1	Glycine, Serine and Threonine Metabolism	L-serine dehydratase	4.3.1.19	KOX_07465/KOX_24645	ser -> nh4 + pyr
THRD_L	Glycine, Serine and Threonine Metabolism	threonine dehydratase	4.3.1.19	KOX_07465/KOX_24645	thr -> obut + nh4
GLYD	Glycine, Serine and Threonine Metabolism	glycine dehydrogenase	1.4.4.2	KOX_02480	gly + lipop -> sap + co2
AMTF1	Glycine, Serine and Threonine Metabolism	aminomethyltransferase	2.1.2.10	KOX_02490(gcvT)	sap + thf -> dlipop + metthf + nh4
DLPD	Glycine, Serine and Threonine Metabolism	dihydrolipoamide dehydrogenase	1.8.1.4	KOX_11055/KOX_20455	dlipop + nad -> lipop + nadh
CHOLD1	Glycine, Serine and Threonine Metabolism	choline dehydrogenase	1.1.99.1	KOX_13810/KOX_26150	choline + fad -> bal + fadh2
DABOT	Glycine, Serine and Threonine Metabolism	diaminobutyrate-2-oxoglutarate transaminase	2.6.1.76	KOX_20320	glu + aspsa -> akg + 24dab
CYSTHS1	Glycine, Serine and Threonine Metabolism	cystathionine beta-synthase	4.2.1.22	KOX_02795	ser + hcys -> llct
CYSTHS2	Glycine, Serine and Threonine Metabolism	cystathionine beta-synthase	4.2.1.22	KOX_02795	ser + h2s -> cys
24DABDC	Glycine, Serine and Threonine Metabolism	L-2,4-diaminobutyrate decarboxylase	4.1.1.86	KOX_20315	24dab -> 13dapro + co2
PGLCM	Glycolysis/Gluconeogenesis	phosphoglucomutase	5.4.2.2	KOX_14430	g1p <-> g6p
LACDH	Glycolysis/Gluconeogenesis	L-lactate dehydrogenase	1.1.1.27	KOX_21270	llac + nad -> pyr + nadh
GA3PD	Glycolysis/Gluconeogenesis	glyceraldehyde-3-phosphate dehydrogenase	1.2.1.12	KOX_17985(gapA)/KOX_19365/KOX_19540	g3p + nad + pi <-> 13pdg + nadh
GLK	Glycolysis/Gluconeogenesis	glucokinase	2.7.1.2	KOX_26735(glk)	atp + glc -> adp + g6p
PYK	Glycolysis/Gluconeogenesis	pyruvate kinase	2.7.1.40	KOX_22750/KOX_23870	adp + pep -> atp + pyr
PGK	Glycolysis/Gluconeogenesis	phosphoglycerate kinase	2.7.2.3	KOX_02585(pgk)	3pg + atp <-> 13pdg + adp
FBP	Glycolysis/Gluconeogenesis	fructose-bisphosphatase	3.1.3.11	KOX_06025(glpX)/KOX_09115	fdp -> f6p + pi
APPS1	Glycolysis/Gluconeogenesis	acylphosphatase	3.6.1.7	KOX_16295	13pdg -> 3pg + pi
FBA	Glycolysis/Gluconeogenesis	fructose-bisphosphate aldolase	4.1.2.13	KOX_02580/KOX_06120/KOX_06125/KOX_06980/ KOX_20975/KOX_25480	fdp <-> dhap + g3p
ENO	Glycolysis/Gluconeogenesis	enolase	4.2.1.11	KOX_01140(eno)	2pg <-> pep
TPI	Glycolysis/Gluconeogenesis	triose-phosphate isomerase	5.3.1.1	KOX_06045(tpiA)/KOX_17440(tpiA)	dhap <-> g3p
PGI	Glycolysis/Gluconeogenesis	glucose-6-phosphate isomerase	5.3.1.9	KOX_08170(pgi)	g6p <-> f6p
PGM	Glycolysis/Gluconeogenesis	phosphoglycerate mutase	5.4.2.1	KOX_05830/KOX_09675/KOX_10370/KOX_19325	2pg <-> 3pg
PDH1	Glycolysis/Gluconeogenesis	pyruvate dehydrogenase E1 component	1.2.4.1	KOX_11045 (aceE)/KOX_20465/KOX_20470	pyr + lipo -> adlipo + co2
PDH2	Glycolysis/Gluconeogenesis	pyruvate dehydrogenase E2 component (dihydrolipoamide acetyltransferase)	2.3.1.12	KOX_11050(aceF)/KOX_20460	coa + adlipo -> accoa + dlipo
PDH3	Glycolysis/Gluconeogenesis	dihydrolipoamide dehydrogenase	1.8.1.4	KOX_11055/KOX_20455	dlipo + nad -> lipo + nadh
PFK	Glycolysis/Gluconeogenesis	6-phosphofructokinase	2.7.1.11	KOX_06965/KOX_07260/KOX_22945	f6p + atp -> fdp + adp

GLC1P	Glycolysis/Gluconeogenesis	Glucose-1-phosphatase	3.1.3.10	KOX_16950	g1p -> glc + pi
ABT6PGH	Glycolysis/Gluconeogenesis	Arbutin 6-phosphate glucohydrolase	3.2.1.86	KOX_00650/KOX_02465/KOX_06100/KOX_09245/ KOX_15175/KOX_18215/KOX_25655/KOX_27145	abt6p -> hqn + g6p
SALC6PGH	Glycolysis/Gluconeogenesis	Salicin 6-phosphate glucohydrolase	3.2.1.86	KOX_00650/KOX_02465/KOX_06100/KOX_09245/ KOX_15175/KOX_18215/KOX_25655/KOX_27145	salc6p -> salchol + g6p
ABTpts	Glycolysis/Gluconeogenesis	Arbutin transport via PEP:Pyr PTS	2.7.1.69	KOX_00645/KOX_06485/KOX_26855	abt_e + pep -> abt6p + pyr
SALCpts	Glycolysis/Gluconeogenesis	Salicin transport via PEP:Pyr PTS	2.7.1.69	KOX_00645/KOX_26855	salc_e + pep -> salc6p + pyr
TARSAR	Glyoxylate and Dicarboxylate metabolism	tartronate semialdehyde reductase	1.1.1.60	KOX_03465(garR)	h3op + nadh <-> glyc-R + nad
LCTAD1	Glyoxylate and Dicarboxylate metabolism	lactaldehyde dehydrogenase	1.2.1.22	KOX_19535	llald + nad -> llac + nadh
LCTAD3	Glyoxylate and Dicarboxylate metabolism	lactaldehyde dehydrogenase	1.2.1.22	KOX_19535	llald + nad <-> llac + nadh
PGLYCP	Glyoxylate and Dicarboxylate metabolism	Phosphoglycolate phosphatase	3.1.3.18	KOX_04585	2ppg -> glycolate + pi
GLCALDD	Glyoxylate and Dicarboxylate metabolism	Glycolaldehyde dehydrogenase	1.2.1.21	KOX_19535	glal + nad -> glycolate + nadh
LALDR2	Glyoxylate and Dicarboxylate metabolism	lactaldehyde reductase	1.1.1.77	KOX_00865	glal + nadh <-> eglycol + nad
GLYCEK1	Glyoxylate and Dicarboxylate metabolism	glycerate kinase	2.7.1.31	KOX_01170/KOX_03460	atp + glyc-R -> 3pg + adp
GLYCDHp	Glyoxylate and Dicarboxylate metabolism	Glycolate dehydrogenase (NADP)	1.1.1.79	KOX_00275/KOX_17100(ghrA)	glx + nadph <-> glycolate + nadp
GLYCDH	Glyoxylate and Dicarboxylate metabolism	Glycolate dehydrogenase (NADP)	1.1.1.79	KOX_00275/KOX_17100(ghrA)	glx + nadh <-> glycolate + nad
FHL	Glyoxylate and Dicarboxylate metabolism	Formate-hydrogen lyase			formate -> co2 + h2
TARTRD	Glyoxylate and Dicarboxylate metabolism	(+)-tartrate dehydratase	4.2.1.32	KOX_03975/KOX_03980	tartr <-> oaa
TARTRDH	Glyoxylate and Dicarboxylate metabolism	tartrate dehydrogenase	1.1.1.93	KOX_16905	tartr + nad <-> oxglycolate + nadh
MTARTRDH	Glyoxylate and Dicarboxylate metabolism	tartrate dehydrogenase	1.1.1.93	KOX_16905	mtartr + nad <-> oxglycolate + nadh
HAKGA	Glyoxylate and Dicarboxylate metabolism	4-hydroxy-2-oxoglutarate aldolase	4.1.3.16	KOX_23850	hydroxyakg <-> pyr + glx
HISTDH	Histidine Metabolism	histidinol dehydrogenase	1.1.1.23	KOX_25035(hisD)	hisol + 2 nad -> his + 2 nadh
ATPPRT	Histidine Metabolism	ATP phosphoribosyltransferase	2.4.2.17	KOX_25030(hisG)	atp + prpp <-> ppi + prbatp
HISTPT	Histidine Metabolism	histidinol-phosphate transaminase	2.6.1.9	KOX_25040	glu + imACP -> akg + hisolp
HISTP	Histidine Metabolism	histidinol-phosphatase	3.1.3.15	KOX_25045	hisolp -> hisol + pi
PRAMPCH	Histidine Metabolism	phosphoribosyl-AMP cyclohydrolase	3.5.4.19	KOX_25065	prbamp -> prfp
PRATPPP	Histidine Metabolism	phosphoribosyl-ATP pyrophosphatase	3.6.1.31	KOX_25065	prbatp -> ppi + prbamp
IMGPDH	Histidine Metabolism	imidazoleglycerol-phosphate dehydratase	4.2.1.19	KOX_25045	dimgp -> imACP
PRMIIZCI	Histidine Metabolism	1-(5-phosphoribosyl)-5-[(5-phosphoribosylamino)methylideneamino)imidazole-4-carboxamide isomerase	5.3.1.16	KOX_25055	prfp -> prlp
IMG3PS	Histidine Metabolism	Imidazole-glycerol-3-phosphate synthase	4.1.3.-/2.4.2.-	KOX_25060/KOX_25050(hisH)	gln + prlp -> aicar + dimgp + glu
HISAL	Histidine metabolism	histidine ammonia-lyase	4.3.1.3	KOX_11930/KOX_14895	his -> urocan + nh4
UROCH	Histidine metabolism	urocanate hydratase	4.2.1.49	KOX_11955/KOX_14890	urocan -> 4i5p
IMZPP	Histidine metabolism	imidazolonepropionase	3.5.2.7	KOX_11965/KOX_14880	4i5p -> nfglu
NFGLUAH	Histidine metabolism	N-formylglutamate amidohydrolase	3.5.3.8	KOX_11970	nfglu -> glu + fa
ALHD7	Histidine metabolism	aldehyde dehydrogenase (NAD+)	1.2.1.3	KOX_00375	i4aa + nad -> i4ac + nadh
MSDHA	Inositol metabolism	malonate-semialdehyde dehydrogenase (acetylating)	1.2.1.18/1.2.1.27	KOX_09140/KOX_09360	3opp + coa + nad -> accoa + co2 + nadh
MI1P	Inositol Phosphate Metabolism	myo-inositol 1-phosphatase	3.1.3.25	KOX_14040/KOX_27375	dmi1p -> mi + pi
MI3P	Inositol Phosphate Metabolism	myo-inositol 3-phosphatase	3.1.3.25	KOX_14040/KOX_27375	dmi3p -> mi + pi
MI4P	Inositol Phosphate Metabolism	myo-inositol 4-phosphatase	3.1.3.25	KOX_14040/KOX_27375	dmi4p -> mi + pi
MI2DH	Inositol Phosphate Metabolism	myo-inositol 2-dehydrogenase	1.1.1.18	KOX_09385/KOX_13300	mi + nad <-> scinos + nadh
MIS2D	Inositol Phosphate Metabolism	myo-inosose-2 dehydratase	4.2.1.44	KOX_09395/KOX_20350	scinos <-> thcych12d
THCYCH12DH	Inositol Phosphate Metabolism	3D-(3,5/4)-trihydroxycyclohexane-1,2-dione hydrolase	3.7.1.-	KOX_09380	thcych12d -> 5dglucn
5DGLUCNI	Inositol Phosphate Metabolism	5-deoxy-glucuronate isomerase	5.3.1.-	KOX_09365	5dglucn -> 2d5kglcn
5DH2DGK	Inositol Phosphate Metabolism	5-dehydro-2-deoxygluconokinase	2.7.1.92	KOX_09375	2d5kglcn + atp -> 2d5kglcn6p + adp
MIHKP4P	Inositol Phosphate Metabolism	myo-inositol-hexakisphosphate 4-phosphohydrolase	3.1.3.26	KOX_14030	phytate -> in12356pkp + pi
UDPAGAT	Lipopolysaccharide Biosynthesis	UDP-N-acetylglucosamine acyltransferase	2.3.1.129	KOX_11505	3hmrsACP + udpnag <-> ACP + udpg2aa
LIPADSS	Lipopolysaccharide Biosynthesis	Lipid A disaccaride synthase	2.4.1.182	KOX_11510(lpxB)	lipidX + udpg23a -> lipidAds + udp
TADSK	Lipopolysaccharide Biosynthesis	Tetraacyldisaccharide 4'kinase	2.7.1.130	KOX_16030(lpxK)	atp + lipidAds -> adp + lipidA
KDOCTT	Lipopolysaccharide Biosynthesis	3-deoxy-manno-octulosonate cytidylyltransferase	2.7.7.38	KOX_16045	ctp + kdo -> ckdo + ppi
KDO8PP	Lipopolysaccharide Biosynthesis	3-deoxy-manno-octulosonate-8-phosphatase	3.1.3.45	KOX_03795	kdo8p -> kdo + pi
KDO8PS	Lipopolysaccharide Biosynthesis	2-dehydro-3-deoxyphosphooctonate aldolase (3-deoxy -D-manno-octulosonic -acid 8-phosphate synthase)	2.5.1.55	KOX_23165	a5p + pep -> kdo8p + pi
AGMHEP	Lipopolysaccharide Biosynthesis	ADP-D-glycero-D-manno-heptose epimerase	5.1.3.20	KOX_05860(rfaD)	adpdgdmhep -> adphep
EDOTXS1	Lipopolysaccharide Biosynthesis	Endotoxin Synthesis (lauroyl transferase)	2.3.1.-	KOX_17165	c120ACP + k2lipiv -> ACP + lk2lipiv
EDOTXS2	Lipopolysaccharide Biosynthesis	Endotoxin Synthesis (myristoyl transferase)	2.3.1.-	KOX_23895	lk2lipiv + c140ACP -> ACP + lipa
GMHEPADT	Lipopolysaccharide Biosynthesis	D-glycero-D-manno-hepose 1-phosphate adenyltransferase	2.7.7.70	KOX_03040	atp + dgdmh1p -> adpdgdmhep + ppi
GMHEPK	Lipopolysaccharide Biosynthesis	D-glycero-D-manno-heptose 7-phosphate kinase	2.7.1.167	KOX_03040	atp + dgdmh7p -> adp + dgdmh17bp
GMHEPBP	Lipopolysaccharide Biosynthesis	D-glycero-D-manno-heptose 1,7-bisphosphate phosphatase	3.1.3.82	KOX_11610	dgdmh17bp -> dgdmh1p + pi
GMHEPBPa	Lipopolysaccharide Biosynthesis	D-glycero-D-manno-heptose 1,7-bisphosphate phosphatase	3.1.3.83	KOX_11610	dgadmh17bp -> dgadmh1p + pi
DMOAT	Lipopolysaccharide Biosynthesis	3-deoxy-D-manno-octulosonic acid transferase	2.4.99.12	KOX_05915	ckdo + lipidA -> cmp + kdolipid4
DMOAT2	Lipopolysaccharide Biosynthesis	3-deoxy-D-manno-octulosonic acid transferase	2.4.99.13	KOX_05915	ckdo + kdolipid4 -> cmp + k2lipiv
S7PISM	Lipopolysaccharide Biosynthesis	sedoheptulose 7-phosphate isomerase	5.3.1.28	KOX_11680(gmhA)	s7p -> dgdmh7p
S7PISMa	Lipopolysaccharide Biosynthesis	sedoheptulose 7-phosphate isomerase	5.3.1.28	KOX_11680(gmhA)	s7p -> dgadmh7p
U3HGAAT	Lipopolysaccharide Biosynthesis	UDP-3-O-(3-hydroxymyristoyl)glucosamine acyltransferase	2.3.1.191	KOX_11495(lpxD)	3hmrsACP + u3hga -> ACP + udpg23a
U3AGDA	Lipopolysaccharide Biosynthesis	UDP-3-O-acetylglucosamine deacetylase	3.5.1.108	KOX_10955(lpxC)	udpg2aa -> ac + u3hga
UDPSH	Lipopolysaccharide Biosynthesis	UDP-sugar hydrolase	3.6.1.54	KOX_13205	udpg23a -> lipidX + ump
HCITS	Lysine Biosynthesis	homocitrate synthase	2.3.3.14	KOX_24970	accoa + akg -> hcit + coa
DHDCR	Lysine Biosynthesis	dihydrodipicolinate reductase (NADPH)	1.3.1.26	KOX_10650	dhdp + nadph -> nadp + tdhdp
THDPSUC	Lysine Biosynthesis	tetrahydrodipicolinate succinylase	2.3.1.117	KOX_11425(dapD)/KOX_1199	succoa + tdhdp -> coa + sl2a6o
SUCDPT	Lysine Biosynthesis	succinyldiaminopimelate transaminase	2.6.1.17	KOX_04525(argD)	akg + sl26da <-> glu + sl2a6o
SUCDPDS	Lysine Biosynthesis	succinyl-diaminopimelate desuccinylase	3.5.1.18	KOX_20740/KOX_27065	sl26da -> 26dap-LL + succ
DAPMDC	Lysine Biosynthesis	diaminopimelate decarboxylase	4.1.1.20	KOX_01685	26dap-M -> co2 + lys
DHDPCS	Lysine Biosynthesis	dihydrodipicolinate synthase	4.2.1.52	KOX_04820/KOX_11900/KOX_24090/KOX_27100	aspsa + pyr -> dhdp
DAPME	Lysine Biosynthesis	diaminopimelate epimerase	5.1.1.7	KOX_07665(dapF)	26dap-LL <-> 26dap-M
OGDH2	Lysine degradation	2-oxoglutarate dehydrogenase E2 component (dihydrolipoamide succinyltransferase)	2.3.1.61	KOX_14595	coa + sgdhl <-> glutcoa + dlipoe
OGDH1	Lysine degradation	2-oxoglutarate dehydrogenase E1 component	1.2.4.2	KOX_14590(sucA)	2oad + lipoe -> sgdhl + co2
ALHD17	Lysine degradation	aldehyde dehydrogenase (NAD+)	1.2.1.3	KOX_00375	4tmab + nad -> 4tmabn + nadh
LYSDC	Lysine degradation	lysine decarboxylase	4.1.1.18	KOX_11535/KOX_13270	lys -> 15dap + co2
ENCOAH3	Membrane Lipid Metabolism	enoyl-CoA hydratase	4.2.1.17	KOX_07835(fadB)/KOX_19445/KOX_26655(fadJ)	3mccoa <-> 3hivcoa
ENCOAH4	Membrane Lipid Metabolism	enoyl-CoA hydratase	4.2.1.17	KOX_07835(fadB)/KOX_19445/KOX_26655(fadJ)	2mp2ecoa -> 3hibcoa
ENCOAH5	Membrane Lipid Metabolism	enoyl-CoA hydratase	4.2.1.17	KOX_07835(fadB)/KOX_19445/KOX_26655(fadJ)	2m2ecoa -> s3h2mbcoa
HACOAD2	Membrane Lipid Metabolism	3-hydroxyacyl-CoA dehydrogenase (3-oxohexanoyl-CoA)	1.1.1.35	KOX_07835(fadB)/KOX_26655(fadJ)	s3h2mbcoa + nad <-> 2maaccoa + nadh
HACOAD3	Membrane Lipid Metabolism	3-hydroxyacyl-CoA dehydrogenase (3-oxooctanoyl-CoA)	1.1.1.35	KOX_07835(fadB)/KOX_26655(fadJ)	hibut + nad <-> mmsa + nadh
ENCOAH6	Membrane Lipid Metabolism	enoyl-CoA hydratase	4.2.1.17	KOX_07835(fadB)/KOX_19445/KOX_26655(fadJ)	carpcoa -> hadpcoa

HACOAD4	Membrane Lipid Metabolism	3-hydroxyacyl-CoA dehydrogenase (3-oxodecanoyl-CoA)	1.1.1.35	KOX_07835(fadB)/KOX_26655(fadJ)	hadpcoa + nad -> ooadpcoa + nadh
ENCOAH7	Membrane Lipid Metabolism	enoyl-CoA hydratase	4.2.1.17	KOX_07835(fadB)/KOX_19445/KOX_26655(fadJ)	2e5mhdcoa -> 3h5m4ecoa
HACOAD5	Membrane Lipid Metabolism	3-hydroxyacyl-CoA dehydrogenase (3-oxododecanoyl-CoA)	1.1.1.35	KOX_07835(fadB)/ KOX_26655(fadJ)	3h5m4ecoa + nad -> 5m3o4hcoa + nadh
3HMYSAS	Membrane Lipid Metabolism	3-hydroxy-myristoyl-ACP synthesis			c120ACP + malACP + nadph -> 3hmrsACP + ACP + co2 + nadp
FALDHD	Methane Metabolism	formaldehyde dehydrogenase	1.1.1.284	KOX_19595	hmgth + nad <-> fmgt + nadh
FGMTH	Methane Metabolism	S-formylglutathione hydrolase	3.1.2.12	KOX_25755	fmgt -> formate + rgt
CATL2	Methane Metabolism	catalase	1.11.1.6/1.11.1.21	KOX_18225(katE)/KOX_26215/KOX_20220	methanol + h2o2 -> formald
2KMBT	Methionine Metabolism	2-keto-4-methylthiobutyrate transamination	2.6.1.57	KOX_08310	2kmb + glu -> akg + met
METS	Methionine Metabolism	methionine synthase	2.1.1.13	KOX_08065(metH)	mthf + hcys <-> met + thf
METADT	Methionine Metabolism	methionine adenosyltransferase	2.5.1.6	KOX_02650	atp + met -> sam + pi + ppi
ADHCYSNS	Methionine Metabolism	S-adenosylhomocysteine nucleosidase	3.2.2.9	KOX_11395	sah -> ad + srlh
CYTTS1	Methionine Metabolism	cystathionine gamma-synthase	2.5.1.48	KOX_07310	cys + oslhser -> llct + succ
CYSTBL1	Methionine Metabolism	cystathionine b-lyase	4.4.1.8	KOX_02875/KOX_21920	llct -> hcys + nh4 + pyr
ADHC1	Methionine metabolism	adenosylhomocysteinase	3.3.1.1	KOX_24860	sah <-> adn + hcys
CYTTS2	Methionine metabolism	cystathionine gamma-synthase	2.5.1.48	KOX_07310	oslhser <-> obut + succ + nh4
CYTTS3	Methionine metabolism	cystathionine gamma-synthase	2.5.1.48	KOX_07310	oahser + cys <-> llct + ac
CYTTS4	Methionine metabolism	cystathionine gamma-synthase	2.5.1.48	KOX_07310	oahser + h2s -> hcys + ac
OAHSL1	Methionine metabolism	O-acetylhomoserine (thiol)-lyase	2.5.1.48	KOX_07310	oahser + tsul + rthio -> hcys + so3 + othio + ac
CYTTS5	Methionine metabolism	cystathionine gamma-synthase	2.5.1.48	KOX_07310	oslhser + h2s <-> hcys + succ
MTTGH	Methionine metabolism	5-methyltetrahydropteroyltriglutamate--homocysteine methyltransferase	2.1.1.14	KOX_07750/KOX_21350/KOX_21375	5mtglu + hcys -> tglu + met
HCYSMT	Methionine metabolism	homocysteine S-methyltransferase	2.1.1.10	KOX_12250(mmuM)	hcys + sam -> met + sah
DNACYSM	Methionine metabolism	DNA (cytosine-5-)-methyltransferase	2.1.1.37	KOX_15390/KOX_24195/KOX_24335	sam + dnacys -> sah + dna5mcys
SAMDC	Methionine metabolism	S-adenosylmethionine decarboxylase	4.1.1.50	KOX_11105	sam -> sama + co2
5MTAN	Methionine metabolism	5'-methylthioadenosine nucleosidase	3.2.2.9	KOX_11395	5mta -> ad + 5mtrib
5MTRIBK	Methionine metabolism	5-methylthioribose kinase	2.7.1.100	KOX_14020(mtnK)	5mtrib + atp -> 5mtribp + adp
5MTRIBPI	Methionine metabolism	methylthioribose-1-phosphate isomerase	5.3.1.23	KOX_14025	5mtribp -> 5mtribup
MTRIBUPD	Methionine metabolism	methylthioribulose-1-phosphate dehydratase	4.2.1.109	KOX_14065	5mtribup -> dikmtpenp
DIKMTPENPP	Methionine metabolism	2,3-diketo-5-methylthio-1-phosphopentane phosphatase	3.1.3.77	KOX_14115	dikmtpenp -> 12dhmetpeno + pi
12DHMETPEND1	Methionine metabolism	1,2-dihydroxy-3-keto-5-methylthiopentene dioxygenase	1.13.11.54	KOX_14110	12dhmetpeno + o2 -> 2kmb + formate
12DHMETPEND2	Methionine metabolism	1,2-dihydroxy-3-keto-5-methylthiopentene dioxygenase	1.13.11.53	KOX_14110	12dhmetpeno + o2 -> 3metpro + formate + co
SALCH1	Naphthalene and Anthracene degradation	salicylate hydroxylase	1.14.13.1	KOX_25675	hnaptho + nadh + o2 -> napth12d + co2 + nad
SALCH2	Dioxin degradation	salicylate hydroxylase	1.14.13.1	KOX_25675	salcyl + o2 + nadh -> catech + co2 + nad
NACMNP	Nicotinate and Nicotinamide metabolism	nicotinic acid mononucleotide pyrophosphorylase	2.4.2.11	KOX_16110	nac + prpp <-> nacn + ppi
NACNDP	Nicotinate and Nicotinamide metabolism	nicotinate-nucleotide diphosphorylase (carboxylating)	2.4.2.19	KOX_11010	prpp + qa -> co2 + nacn + ppi
NADK	Nicotinate and Nicotinamide metabolism	NAD kinase	2.7.1.23	KOX_00110(ppnK)	atp + nad -> adp + nadp
NAMNAT	Nicotinate and Nicotinamide metabolism	nicotinamide-nucleotide adenylyltransferase	2.7.7.18	KOX_14250(nadD)	atp + namn <-> nad + ppi
NACM	Nicotinate and Nicotinamide metabolism	nicotinamidase	3.5.1.19	KOX_18050	nam -> nac + nh4
NADDP1	Nicotinate and Nicotinamide metabolism	NAD diphosphatase	3.6.1.22	KOX_07975(nudC)	nad -> amp + namn
NADDP1e	Nicotinate and Nicotinamide metabolism	NAD diphosphatase	3.6.1.22	KOX_07975(nudC)	nad -> amp + namn_e
ASPOX2	Nicotinate and Nicotinamide metabolism	L-aspartate oxidase	1.4.3.16	KOX_27585	asp + o2 <-> h2o2 + iasp
ASPOX3	Nicotinate and Nicotinamide metabolism	L-aspartate oxidase	1.4.3.16	KOX_27585	asp + uq -> iasp + uqh2
ASPOX4	Nicotinate and Nicotinamide metabolism	L-aspartate oxidase	1.4.3.16	KOX_27585	asp + mk -> iasp + mqn
ASPOX5	Nicotinate and Nicotinamide metabolism	L-aspartate oxidase	1.4.3.16	KOX_27585	asp + fum -> iasp + succ
QULS	Nicotinate and Nicotinamide metabolism	quinolinate synthase	2.5.1.72	KOX_14740	dhap + iasp <-> pi + qa
NTNAT	Nicotinate and Nicotinamide metabolism	nicotinate-nucleotide adenylyltransferase	2.7.7.18	KOX_14250(nadD)	atp + nacn <-> dnad + ppi
NTD13	Nicotinate and Nicotinamide metabolism	5'-nucleotidase	3.1.3.5	KOX_01045(surE)KOX_09525/KOX_09680/KOX_10270/KOX_13075(ushA)/KOX_26410	namn -> namd + pi
NTD14	Nicotinate and Nicotinamide metabolism	5'-nucleotidase	3.1.3.5	KOX_01045(surE)KOX_09525/KOX_09680/KOX_10270/KOX_13075(ushA)/KOX_26410	nacn -> nacd + pi
NADDP2	Nicotinate and Nicotinamide metabolism	NAD diphosphatase	3.6.1.22	KOX_07975(nudC)	dnad -> amp + nacn
NADSYN	Nicotinate and Nicotinamide metabolism	NAD synthase	6.3.1.5	KOX_18185(nadE)	atp + dnad + nh4 -> amp + ppi + nad
PUNPP9	Nicotinate and Nicotinamide metabolism	purine-nucleoside phosphorylase	2.4.2.1	KOX_10320(deoD)	namd + pi <-> nam + r1p
PUNPP10	Nicotinate and Nicotinamide metabolism	purine-nucleoside phosphorylase	2.4.2.1	KOX_10320(deoD)	nacd + pi <-> nac + r1p
NODOX1	Nitrogen Metabolism	nitric oxide dioxygenase	1.14.12.17	KOX_27435	nadh + 2 no + 2 o2 -> nad + 2 no3
NODOX2	Nitrogen Metabolism	nitric oxide dioxygenase	1.14.12.17	KOX_27435	nadph + 2 no + 2 o2 -> nadp + 2 no3
NO3RUq1	Nitrogen Metabolism	Nitrate reductase (Ubiquinol-8)	1.7.99.4	KOX_19980/KOX_19985/KOX_19990/KOX_19995/KOX_23065/KOX_23070/KOX_23075/KOX_23080/KOX_23105	no3 + uqh2 -> no2 + uq + 2 hext
NITNMOX	Nitrogen Metabolism	nitronate monooxygenase	1.13.12.16	KOX_21140	ethnit + o2 + fmnh2 -> acal + no2 + fmn
NITRT1	Nitrogen metabolism	nitrite reductase	1.7.1.4	KOX_04560/KOX_04565(nirD)/KOX_23110	no2 + 3 nadh -> 3 nad + nh4
NITRT2	Nitrogen metabolism	nitrite reductase	1.7.1.4	KOX_04560/KOX_04565(nirD)/KOX_23110	no2 + 3 nadph -> 3 nadp + nh4
NITGEN	Nitrogen metabolism	nitrogenase	1.18.6.1	KOX_24920/KOX_24925/KOX_24930/KOX_24975/KOX_14410/KOX_24990/	16 atp + n2 + 8 rdferdx -> 16 pi + 16 adp + 8 oxferdx + 2 nh4 + h2
CAPK2	Nitrogen metabolism	carbamate kinase	2.7.2.2	KOX_23275	cabm + atp -> cap + adp
CARBONAH	Nitrogen metabolism	carbonic anhydrase	4.2.1.1	KOX_04135/KOX_11145/KOX_22245	h2co3 <-> co2
ADNK3	Nucleotide Salvage Pathway	adentylate kinase (GTP)	2.7.4.3	KOX_13050(adk)	amp + gtp <-> adp + gdp
ADNK4	Nucleotide Salvage Pathway	adentylate kinase (ITP)	2.7.4.3	KOX_13050(adk)	amp + itp <-> adp + idp
TDPDRHR	Nucleotide sugars metabolism	dTDP-4-dehydrorhamnose reductase	1.1.1.133	KOX_25130	dtdp4d6dm + nadph -> dtdprmn + nadp
UDPG6D	Nucleotide sugars metabolism	UDPglucose 6-dehydrogenase	1.1.1.22	KOX_25120	2 nad + udpg -> 2 nadh + udpglcur
G1PTT1	Nucleotide sugars metabolism	glucose-1-phosphate thymidylyltransferase	2.7.7.24	KOX_25135	dttp + g1p -> dtdpglu + ppi
UG1PUT	Nucleotide sugars metabolism	UTP-glucose-1-phosphate uridylyltransferase (irreversible)	2.7.7.9	KOX_23040/KOX_25220	g1p + utp -> ppi + udpg
TDPGLUD	Nucleotide sugars metabolism	dTDPglucose 4,6-dehydratase	4.2.1.46	KOX_25140	dtdpglu -> dtdp4d6dg
TDPDRHE	Nucleotide sugars metabolism	dTDP-4-dehydrorhamnose 3,5-epimerase	5.1.3.13	KOX_25125	dtdp4d6dg -> dtdp4d6dm
UDPG4E1	Nucleotide sugars metabolism	UDP-glucose 4-epimerase	5.1.3.2	KOX_14785	dtdpglu <-> dtdpgal
UDPG4E2	Nucleotide sugars metabolism	UDP-glucose 4-epimerase	5.1.3.2	KOX_14785	udpg <-> udpgal
AMTF2	One carbon pool by Folate	aminomethyltransferase	2.1.2.10	KOX_02490(gcvT)	methf -> 5fthf
METHFH	One carbon pool by Folate	5,10-Methenyltetrahydrofolate 5-hydrolase (decyclizing)	3.5.4.9	KOX_13225	methf <-> fthf
FORTF	One carbon pool by Folate	5-formyltetrahydrofolate cyclo-ligase	6.3.3.2	KOX_02520	5fthf + atp -> adp + pi + methf
LACDHq	Oxidative Phosphorylation	L-Lactate dehydrogenase (ubiquinone)	1.1.2.3	KOX_05795(lldD)	llac + uq -> pyr + uqh2
LACDHm	Oxidative Phosphorylation	L-Lactate dehydrogenase (menaquinone)	1.1.2.3	KOX_05795(lldD)	llac + mk -> pyr + mqn
PRGNFT2	One carbon pool by Folate	phosphoribosylglycinamide formyltransferase	2.1.2.2	KOX_23845(purT)/KOX_27160(purN)	methf + gar -> thf + fgam
GL3PDq	Oxidative Phosphorylation	glycerol-3-phosphate dehydrogenase (ubiquinone-8)	1.1.99.5		glyc3p + uq -> dhap + uqh2
GL3PDd	Oxidative Phosphorylation	glycerol-3-phosphate dehydrogenase (demethylmenaquinone-8)	1.1.99.5		glyc3p + 2dmmq8 -> dhap + 2dmmql8
GL3PDm	Oxidative Phosphorylation	glycerol-3-phosphate dehydrogenase (menaquinone-8)	1.1.99.5		glyc3p + mk -> dhap + mqn
HYDGq	Oxidative Phosphorylation	hydrogenase (ubiquinone-8: 2 protons)	1.18.99.1		uq + h2 -> uqh2 + 2 hext
HYDGd	Oxidative Phosphorylation	hydrogenase (demethylmenaquinone-8: 2 protons)	1.18.99.1		2dmmq8 + h2 -> 2dmmql8 + 2 hext
HYDGm	Oxidative Phosphorylation	hydrogenase (menaquinone-8: 2 protons)	1.18.99.1		mk + h2 -> mqn + 2 hext
FDHGq	Oxidative Phosphorylation	formate dehydrogenase (quinone-8)	1.2.2.1		formate + uq -> co2 + uqh2 + 2 hext
FDHGm	Oxidative Phosphorylation	formate dehydrogenase (menaquinone-8)	1.2.2.1		formate + mk -> co2 + mqn + 2 hext

POX	Oxidative Phosphorylation	pyruvate oxidase	1.2.5.1	KOX_15775	pyr + uq -> ac + co2 + uqh2
SUCCDq	Oxidative Phosphorylation	succinate dehydrogenase	1.3.99.1	KOX_08760/KOX_08765/KOX_08770/KOX_08775/ KOX_14570(sdhC)/KOX_14575(sdhD)/KOX_14580 (sdhA)/KOX_14585(sdhB)/KOX_21720	uq + succ -> fum + uqh2
NADPTH	Oxidative Phosphorylation	NAD(P) transhydrogenase	1.6.1.1/1.6.1.2	KOX_07395/KOX_21820(pntB)/KOX_21825(pntA)	nadh + nadp + 2 hext -> nad + nadph
NADHHq1	Oxidative Phosphorylation	NADH dehydrogenase (ubiquinone-8 & 3 protons)	1.6.5.3/1.6.99.3	KOX_26335/KOX_26340/KOX_26345/KOX_26350/ KOX_26355/KOX_26360/KOX_26365/KOX_26370/ KOX_26375/KOX_26380/KOX_26385/KOX_26390/ KOX_26395/KOX_17375	nadh + uq -> nad + uqh2 + 3 hext
NADHHd1	Oxidative Phosphorylation	NADH dehydrogenase (demethylmenaquinone-8 & 3 protons)	1.6.5.3/1.6.99.3	KOX_26335/KOX_26340/KOX_26345/KOX_26350/ KOX_26355/KOX_26360/KOX_26365/KOX_26370/ KOX_26375/KOX_26380/KOX_26385/KOX_26390/ KOX_26395/KOX_17375	nadh + 2dmmq8 -> nad + 2dmmql8 + 3 hext
NADHHm1	Oxidative Phosphorylation	NADH dehydrogenase (menaquinone-8 & 3 protons)	1.6.5.3/1.6.99.3	KOX_26335/KOX_26340/KOX_26345/KOX_26350/ KOX_26355/KOX_26360/KOX_26365/KOX_26370/ KOX_26375/KOX_26380/KOX_26385/KOX_26390/ KOX_26395/KOX_17375	nadh + mk -> nad + mqn + 3 hext
NADHHq2	Oxidative Phosphorylation	NADH dehydrogenase (ubiquinone-8)	1.6.5.3/1.6.99.3	KOX_26335/KOX_26340/KOX_26345/KOX_26350/ KOX_26355/KOX_26360/KOX_26365/KOX_26370/ KOX_26375/KOX_26380/KOX_26385/KOX_26390/ KOX_26395/KOX_17375	nadh + uq -> nad + uqh2
NADHHd2	Oxidative Phosphorylation	NADH dehydrogenase (demethylmenaquinone-8)	1.6.5.3/1.6.99.3	KOX_26335/KOX_26340/KOX_26345/KOX_26350/ KOX_26355/KOX_26360/KOX_26365/KOX_26370/ KOX_26375/KOX_26380/KOX_26385/KOX_26390/ KOX_26395/KOX_17375	nadh + 2dmmq8 -> nad + 2dmmql8
NADHHm2	Oxidative Phosphorylation	NADH dehydrogenase (menaquinone-8)	1.6.5.3/1.6.99.3	KOX_26335/KOX_26340/KOX_26345/KOX_26350/ KOX_26355/KOX_26360/KOX_26365/KOX_26370/ KOX_26375/KOX_26380/KOX_26385/KOX_26390/ KOX_26395/KOX_17375	nadh + mk -> nad + mqn
NO3RUq2	Oxidative Phosphorylation	Nitrate reductase (ubiquinol-8)	1.7.99.4	KOX_19980/KOX_19985/KOX_19990/KOX_19995/ KOX_23065/KOX_23070/KOX_23075/KOX_23080/ KOX_23105	no3 + uqh2 -> no2 + uq + 2 hext
NO3RUm	Oxidative Phosphorylation	Nitrate reductase (menaquinol-8)	1.7.99.4	KOX_19980/KOX_19985/KOX_19990/KOX_19995/ KOX_23065/KOX_23070/KOX_23075/KOX_23080/ KOX_23105	no3 + mqn -> no2 + mk + 2 hext
THIORp	Oxidative Phosphorylation	thioredoxin reductase (NADPH)	1.8.1.9	KOX_15885	nadph + othio -> nadp + rthio
ATPSYN	Oxidative Phosphorylation	ATP synthase (four protons for one ATP)	3.6.3.14	KOX_06705(atpC)/KOX_06710/KOX_06715/KOX_ 06720/KOX_06725/KOX_06730/KOX_06735/KOX_ 06740	adp + pi + 4 hext <-> atp
BTCRNCT	Oxidative Phosphorylation	gamma-butyrobetainyl-CoA: carnitine CoA transferase			bbtcoa + crn <-> crncoa + gbbtn
CTBTCRNCT	Oxidative Phosphorylation	crotonobetainyl-CoA: carnitine CoA transferase			crn + ctbtcoa <-> crncoa + ctbt
CRNCDH	Oxidative Phosphorylation	Carnityl-CoA dehydratse	4.2.1.89		crncoa <-> ctbtcoa
CRNCOAL	Oxidative phosphorylation	Carnitine-CoA Ligase			atp + coa + crn -> adp + crncoa + pi
CYTCOBDq	Oxidative Phosphorylation	cytochrome oxidase bd (ubiquinol-8: 2 protons)			0.5 o2 + uqh2 -> uq + 2 hext
CYTCOBOq3	Oxidative Phosphorylation	cytochrome oxidase bo3 (ubiquinol-8: 4 protons)			0.5 o2 + uqh2 -> uq + 4 hext
NADTRHG	Oxidative Phosphorylation	NAD transhydrogenase			nad + nadph -> nadh + nadp
NITRR	Oxidative Phosphorylation	nitrite Reductase (NADH)			3 nadh + no2 -> 3 nad + nh4
SUCCD2	Oxidative phosphorylation	succinate dehydrogenase			fadh2 + uq -> fad + uqh2
DMSORDm	Oxidative phosphorylation	Dimethyl sulfoxide reductase (Menaquinol 8)	1.8.5.3	KOX_15920	dmso + mqn -> dms + mk
DMSORDd	Oxidative phosphorylation	Dimethyl sulfoxide reductase (Demethylmenaquinol 8)	1.8.5.3	KOX_15920	dmso + 2dmmql8 -> dms + 2dmmq8
DMSORDme	Oxidative phosphorylation	Dimethyl sulfoxide reductase (Menaquinol 8)	1.8.5.3	KOX_15920	dmso_e + mqn -> dms_e + mk
DMSORDde	Oxidative phosphorylation	Dimethyl sulfoxide reductase (Demethylmenaquinol 8)	1.8.5.3	KOX_15920	dmso_e + 2dmmql8 -> dms_e + 2dmmq8
TMAORm	Oxidative phosphorylation	Trimethylamine N-oxide reductase (menaquinol 8)			tmao + mqn -> tma + mk
TMAORd	Oxidative phosphorylation	Trimethylamine N-oxide reductase (demethylmenaquinol 8)			tmao + 2dmmql8 -> tma + 2dmmq8
TMAORme	Oxidative phosphorylation	Trimethylamine N-oxide reductase (menaquinol 8)			hext + tmao_e + mqn -> tma_e + mk
TMAORde	Oxidative phosphorylation	Trimethylamine N-oxide reductase (demethylmenaquinol 8)			hext + tmao_e + 2dmmql8 -> tma_e + 2dmmq8
GLCDHe	Oxidative phosphorylation	Glucose dehydrogenase (ubiquinone-8 as acceptor)			glc_e + uq -> gluc_e + hext + uqh2
NADPHQRq	Oxidative phosphorylation	NADPH Quinone Reductase (Ubiquinone-8)			nadph + uq -> nadp + uqh2
NADPHQRd	Oxidative phosphorylation	NADPH Quinone Reductase (Demethylmenaquinone-8)			nadph + 2dmmq8 -> nadp + 2dmmql8
NADPHQRm	Oxidative phosphorylation	NADPH Quinone Reductase (Menaquinone-8)			nadph + mk -> nadp + mqn
PPK2	Oxidative phosphorylation	polyphosphate kinase	2.7.4.1	KOX_27185	atp + ppi <-> adp + pppi
PPK1	Oxidative phosphorylation	polyphosphate kinase	2.7.4.1	KOX_27185	atp + pi <-> adp + ppi
QUIMOXq	Oxidative phosphorylation	quinol monooxygenase (Ubiquinol-8)			2 o2 + uqh2 -> 2 o2s + uq
QUIMOXm	Oxidative phosphorylation	quinol monooxygenase (Menaquinol-8)			mqn + 2 o2 -> mk + 2 o2s
CYTCOBDm	Oxidative phosphorylation	cytochrome oxidase bd (menaquinol-8: 2 protons)			mqn + 0.5 o2 -> 2 hext + mk
DP2R	Pantothenate and CoA biosynthesis	2-dehydropantoate 2-reductase	1.1.1.169	KOX_12630/KOX_20880	dhpant + nadph -> nadp + pant
MOBHMT	Pantothenate and CoA biosynthesis	3-methyl-2-oxobutanoate hydroxymethyltransferase	2.1.2.11	KOX_11215(panB)	3mob + metthf -> dhpant + thf
DPCOAK	Pantothenate and CoA biosynthesis	dephospho-CoA kinase	2.7.1.24	KOX_10985(coaE)	atp + dpcoa -> adp + coa
PNTOK1	Pantothenate and CoA biosynthesis	pantothenate kinase	2.7.1.33	KOX_07870	atp + pnto -> 4ppnto + adp
PTHPAT	Pantothenate and CoA biosynthesis	pantetheine-phosphate adenylyltransferase	2.7.7.3	KOX_05925(coaD)	atp + 4ppnte <-> dpcoa + ppi
ACPS	Pantothenate and CoA biosynthesis	acyl-carrier protein synthase	2.7.8.7	KOX_27530(acpS)/KOX_05045	apoACP + coa -> ACP + pap
PPTCDC	Pantothenate and CoA biosynthesis	phosphopantothenoylcysteine decarboxylase	4.1.1.36	KOX_05950	4ppcys -> co2 + 4ppnte
PANTOS	Pantothenate and CoA biosynthesis	pantothenate synthase	6.3.2.1	KOX_11210(panC)	bala + atp + pant -> amp + pnto + ppi
PPNTCL1	Pantothenate and CoA biosynthesis	phosphopantothenate-cysteine ligase	6.3.2.5	KOX_05950	4ppnto + atp + cys -> 4ppcys + amp + ppi
PPNTCL2	Pantothenate and CoA biosynthesis	phosphopantothenate-cysteine ligase	6.3.2.5	KOX_05950	4ppnto + ctp + cys -> 4ppcys + cmp + ppi
PNTOK2	Pantothenate and Coa Biosynthesis	pantothenate kinase	2.7.1.33	KOX_07870	atp + pantcys -> adp + 4ppcys
PNTOK3	Pantothenate and Coa Biosynthesis	pantothenate kinase	2.7.1.33	KOX_07870	atp + ptt -> adp + 4ppnte
ACPPDE	Pantothenate and CoA biosynthesis	acyl carrier protein phosphodiesterase	3.1.4.14	KOX_12515	ACP -> 4ppnte + apoACP
ALTRNH	Pentose and glucuronate interconversion	altronate hydrolase	4.2.1.7	KOX_03375/KOX_04830	dalt -> kdg
TAGATNR	Pentose and glucuronate interconversion	tagaturonate reductase	1.1.1.58	KOX_10130/KOX_21230	tagatn + nadh -> dalt + nad
MANND	Pentose and glucuronate interconversion	mannonate dehydratase	4.2.1.8	KOX_11855	kdg <-> mann
MANNOX	Pentose and glucuronate interconversion	D-mannonate oxidoreductase	1.1.1.57	KOX_11850/KOX_21505	mann + nad <-> frutn + nadh
GLUCIS1	Pentose and glucuronate interconversion	glucuronate isomerase	5.3.1.12	KOX_03380	frutn <-> dgluc
GLUCIS2	Pentose and glucuronate interconversion	glucuronate isomerase	5.3.1.12	KOX_03380	dgalac -> tagatn
ARAIS	Pentose and glucuronate interconversion	L-arabinose isomerase	5.3.1.4	KOX_10760	larabinose <-> lrib
LRIBK	Pentose and glucuronate interconversion	L-ribulokinase	2.7.1.16	KOX_10765	lrib + atp <-> lrl5p + adp
LRIB5P4E	Pentose and glucuronate interconversion	L-ribulose-5-phosphate 4-epimerase	5.1.3.4	KOX_10755(araD)	lrl5p <-> xu5p
XYLK	Pentose and glucuronate interconversion	xylulokinase	2.7.1.17	KOX_05620/KOX_14070/KOX_25450	xu5p + adp <-> xylu + atp
XYLIS1	Pentose and glucuronate interconversion	xylose isomerase	5.3.1.5	KOX_05625	xylu <-> xyl
XYLIS2	Pentose and glucuronate interconversion	xylose isomerase	5.3.1.5	KOX_05625	fru -> glc
DRIBK	Pentose and glucuronate interconversion	ribulokinase	2.7.1.16	KOX_10765	drib + atp <-> rl5p + adp
XYL5P3E	Pentose and glucuronate interconversion	L-xylulose 5-phosphate 3-epimerase	5.-.-.-/5.1.3.22	KOX_05705	lxu5p <-> lrl5p
3DHG6PD	Pentose and glucuronate interconversion	3-dehydro-L-gulonate-6-phosphate decarboxylase	4.1.1.85	KOX_05700(sgbH)	3dhg6p -> lxu5p + co2
LXYLK	Pentose and glucuronate interconversion	L-xylulokinase	2.7.1.53	KOX_05695/KOX_14470/KOX_20985	lxylu + atp <-> lxu5p + adp
RMKx	Pentose and glucuronate interconversion	rhamnulokinase	2.7.1.5	KOX_07210(rhaB)	lxylu + atp <-> lxu1p + adp
RM1PAx	Pentose and glucuronate interconversion	rhamnulose-1-phosphate aldolase	4.1.2.19	KOX_07200	lxu1p <-> dhap + glal
ARBTD	Pentose and glucuronate interconversion	D-arabinitol dehydrogenase	1.1.1.11	KOX_25455	xylu + nadh <-> darbt + nad
3DHGXYLK	Pentose and glucuronate interconversion	L-xylulokinase	2.7.1.53	KOX_05695/KOX_14470/KOX_20985	3dhg + atp -> 3dhg6p + adp
DKGULNR	Pentose and glucuronate interconversion	2,3-diketo-L-gulonate reductase	1.1.1.130	KOX_05675	3dhg + nad <-> 23dkgul + nadh
GLCNK	Pentose Phosphate Pathway	gluconokinase	2.7.1.12	KOX_04750(gntK)	atp + gluc -> d6pgc + adp
DDGLCNK	Pentose Phosphate Pathway	2-dehydro-3-deoxygluconokinase	2.7.1.45	KOX_05360/KOX_05550	kdg + atp -> kdpg + adp
AB5PI	Pentose Phosphate Pathway	arabinose-5-phosphate isomerase	5.3.1.13	KOX_03790	rl5p <-> a5p
R15BPK	Pentose Phosphate Pathway	ribose-1,5-bisphosphokinase	2.7.4.23	KOX_08535	atp + r15bp -> adp + prpp
PRPPS	Pentose Phosphate Pathway	phosphoribosylpyrophosphate synthetase	2.7.6.1	KOX_04485/KOX_23205	atp + r5p <-> amp + prpp

G6PDH	Pentose Phosphate Pathway	glucose 6-phosphate dehydrogenase	1.1.1.49	KOX_23860	g6p + nadp <-> 6pgl + nadph
TRKT1	Pentose Phosphate Pathway	transketolase	2.2.1.1	KOX_02620/KOX_13875/KOX_13880/KOX_15060/KOX_15065/KOX_15945/KOX_15950/KOX_17475/KOX_26445/KOX_26450/KOX_27035	r5p + xu5p <-> g3p + s7p
TRKT2	Pentose Phosphate Pathway	transketolase	2.2.1.1	KOX_02620/KOX_13875/KOX_13880/KOX_15060/KOX_15065/KOX_15945/KOX_15950/KOX_17475/KOX_26445/KOX_26450/KOX_27035	e4p + xu5p <-> f6p + g3p
TRADL	Pentose Phosphate Pathway	transaldolase	2.2.1.2	KOX_10435/KOX_17470	g3p + s7p <-> e4p + f6p
PGL	Pentose Phosphate Pathway	6-phosphogluconolactonase	3.1.1.31	KOX_14825	6pgl -> d6pgc
EDA	Pentose Phosphate Pathway	2-dehydro-3-deoxy-phosphogluconate aldolase	4.1.2.14	KOX_23850	kdpg -> g3p + pyr
EDD	Pentose Phosphate Pathway	6-phosphogluconate dehydratase	4.2.1.12	KOX_23855	d6pgc -> kdpg
RPE	Pentose Phosphate Pathway	ribulose 5-phosphate 3-epimerase	5.1.3.1	KOX_04590	rl5p <-> xu5p
RPI	Pentose Phosphate Pathway	ribose-5-phosphate isomerase	5.3.1.6	KOX_02530/KOX_08520/KOX_17465	r5p <-> rl5p
GND	Pentose Phosphate Pathway	6-phosphogluconate dehydrogenase	1.1.1.44	KOX_25145	d6pgc + nadp -> rl5p + nadph + co2
QUIGDH	Pentose Phosphate Pathway	quinoprotein glucose dehydrogenase	1.1.5.2	KOX_11135	glc + uq -> g15l + uqh2
DOXPAD	Pentose Phosphate Pathway	deoxyribose-phosphate aldolase	4.1.2.4	KOX_01970/KOX_10305	g3p + acal <-> 2dr5p
RIBK1	Pentose Phosphate Pathway	ribokinase	2.7.1.15	KOX_06810/KOX_20705	2dr5p + adp <-> doxrib + atp
RIBK2	Pentose Phosphate Pathway	ribokinase	2.7.1.15	KOX_06810/KOX_20705	rib + atp <-> r5p + adp
GLUCDHp	Pentose Phosphate Pathway	gluconate 2-dehydrogenase	1.1.1.215	KOX_05560	2kgluc + nadph <-> gluc + nadp
GLUCDH	Pentose Phosphate Pathway	gluconate 2-dehydrogenase	1.1.1.215	KOX_05560	2kgluc + nadh <-> gluc + nad
PAMPPT	Peptidoglycan Biosynthesis	phospho-N-acetylmuramoyl-pentapeptide-transferase (meso-2,6-diaminopimelate)	2.7.8.13	KOX_10910(mraY)	udcpp + ugmda -> uagmda + ump
UDCPDP	Peptidoglycan Biosynthesis	undecaprenyl-diphosphatase	3.6.1.27	KOX_03065	udcpdp -> pi + udcpp
UAMAGDS	Peptidoglycan Biosynthesis	UDP-N-acetylmuramoyl-L-alanyl-D-glutamyl-meso-2,6-diaminopimelate synthetase	6.3.2.13	KOX_10900	26dap-M + atp + uamag -> adp + pi + ugmd
UAMAGDAS	Peptidoglycan Biosynthesis	UDP-N-acetylmuramoyl-L-alanyl-D-glutamyl-meso-2,6-diaminopimeloyl-D-alanyl-D-alanine synthetase	6.3.2.10	KOX_10905(murF)	alaala + atp + ugmd -> adp + pi + ugmda
UACMAS	Peptidoglycan Biosynthesis	UDP-N-acetylmuramoyl-L-alanine synthetase	6.3.2.8	KOX_10930(murC)	ala + atp + udpnam -> adp + pi + uama
UACMAGS	Peptidoglycan Biosynthesis	UDP-N-acetylmuramoyl-L-alanyl-D-glutamate synthetase	6.3.2.9	KOX_10915(murD)	atp + dglu + uama -> adp + pi + uamag
UAGMPUT	Peptidoglycan Biosynthesis	UDP-N-acetylglucosamine-N-acetylmuramyl-(pentapeptide)pyrophosphoryl-undecaprenol N-acetylglucosamine transferase	2.4.1.227	KOX_10925(murG)	udpnag + uagmda -> uaagmda + udp
GLNST2	Peptidoglycan Biosynthesis	glutamine synthetase	6.3.1.2		uaagmda + atp + nh4 -> uaagmmda + adp + pi
NACMAA	Peptidoglycan Biosynthesis	N-acetylmuramoyl-L-alanine amidase	3.5.1.28	KOX_01555/KOX_08830/KOX_15755/KOX_26930	acala -> acmur + ala
UNAMPLA	Peptidoglycan Biosynthesis	UDP-N-acetylmuramoylpentapeptide-lysine N6-alanyltransferase	2.3.2.10		5 gly + uaagmmda -> uaagmm5da
PGPS	Peptidoglycan Biosynthesis	peptidoglycan precursor synthesis			uaagmm5da -> udcpdp + ppeptido
DALAT	Peptidoglycan Biosynthesis	D-alanine transaminase	2.6.1.21		ppeptido + dala -> PEPTIDO + dala_e
ALHD3	Phenylalanine metabolism	aldehyde dehydrogenase (phenylacetaldehyde, NAD)	1.2.1.39		nad + pacald -> nadh + pac
OXP4EH	Toluene and Xylene degradation	2-oxopent-4-enoate hydratase	4.2.1.80	KOX_22680(mhpD)	op4en -> hopt
4H2OPPL	Toluene and Xylene degradation	4-hydroxy-2-oxopentanoate pyruvate-lyase (acetaldehyde-forming)	4.1.3.39	KOX_22670	hopt -> acal + pyr
PHEACL	Phenylalanine metabolism	phenylacetate-CoA ligase	6.2.1.30	KOX_07020	atp + coa + pac -> amp + phaccoa + ppi
3HCINNMH	Phenylalanine metabolism	3-hydroxycinnamate hydroxylase	1.14.13.127	KOX_22695(mhpA)	3hcinnm + nadh + o2 -> dhcinnm + nad
3HPPPNH	Phenylalanine metabolism	3-(3-hydroxy-phenyl)propionate hydroxylase	1.14.13.127	KOX_22695(mhpA)	3hpppn + nadh + o2 -> dhpppn + nad
4H2OPNTA	Phenylalanine metabolism	4-hydroxy-2-oxopentanoate aldolase	4.1.3.39	KOX_22670	hopt -> acal + pyr
HPPH	Phenylalanine metabolism	hippurate hydrolase	3.5.1.32	KOX_13405/KOX_18515/KOX_22550	benzot + gly -> hppr
CNTOB	Phenylalanine metabolism	unclear reaction			cinnm + nad -> benzot + ac + nadh
4HPHED2	Phenylalanine metabolism	4-hydroxyphenylpyruvate dioxygenase	1.13.11.27	KOX_22735	phpyr + o2 -> 2hpa + co2
MNAO9	Phenylalanine metabolism	monoamine oxidase	1.4.3.21	KOX_19410(tynA)	peamn + o2 -> pacald + nh4 + h2o2
DAAD2	Phenylalanine metabolism	D-Amino acid dehydrogenase	1.4.99.1	KOX_23495	dphe + fad -> phpyr + nh4 + fadh2
CATPRX	Phenylalanine metabolism	catalase-peroxidase	1.11.1.21	KOX_20220	phe + o2 -> pheact + co2
PHACCOAO	Phenylalanine metabolism	phenylacetate-CoA oxygenase	1.14.13.149	KOX_19420(paaA)/KOX_19425(paaB)/KOX_19430/KOX_19435/KOX_19440	phaccoa + o2 + nadph -> 2epoxaccoa + nadp
2EPOXACCOAI1	Phenylalanine metabolism	2-(1,2-epoxy-1,2-dihydrophenyl)acetyl-CoA isomerase	5.3.3.18	KOX_19450	2epoxaccoa <-> 2oxpyaccoa
OXPINCOAH	Phenylalanine metabolism	oxepin-CoA hydrolase	3.7.1.16	KOX_19415	2oxpyaccoa -> 3oxdscoasa
3OXDSCOASAD	Phenylalanine metabolism	3-oxo-5,6-dehydrosuberyl-CoA semialdehyde dehydrogenase	1.17.1.7	KOX_19415	3oxdscoasa + nadp -> 3oxdscoa + nadph
2EPOXACCOAI2	Phenylalanine metabolism	2-(1,2-epoxy-1,2-dihydrophenyl)acetyl-CoA isomerase	5.3.3.18	KOX_19450	3oxdscoa + coa -> carpcoa + accoa
ENCOAH8	Phenylalanine metabolism	enoyl-CoA hydratase	4.2.1.17	KOX_19445	carpcoa -> hadpcoa
3HACOADH	Phenylalanine metabolism	3-hydroxyacyl-CoA dehydrogenase	1.1.1.157	KOX_19455	hadpcoa + nad -> ooadpcoa + nadh
DHPHEPDO1	Phenylalanine metabolism	2,3-dihydroxyphenylpropionate 1,2-dioxygenase	1.13.11.16	KOX_22690	dhcinnm + o2 -> 2h6kated
DHPHEPDO2	Phenylalanine metabolism	2,3-dihydroxyphenylpropionate 1,2-dioxygenase	1.13.11.16	KOX_22690	dhpppn + o2 -> 2h6oxdiend
2H6KDH1	Phenylalanine metabolism	2-hydroxy-6-ketonona-2,4-dienedioic acid hydrolase	3.7.1.-	KOX_22685	2h6kated -> op4en + fum
2H6KDH2	Phenylalanine metabolism	2-hydroxy-6-ketonona-2,4-dienedioic acid hydrolase	3.7.1.-	KOX_22685	2h6oxdiend -> op4en + succ
SHKDH	Phenylalanine, Tyrosine and Tryptophan biosynthesis	shikimate dehydrogenase	1.1.1.25	KOX_04180(aroE)/KOX_08090/KOX_10830(aroE)/KOX_12685/KOX_22725	dhsk + nadph <-> nadp + sme
QTDH2	Phenylalanine, Tyrosine and Tryptophan biosynthesis	quinate dehydrogenase (pyrroloquinoline-quinone)	1.1.5.8	KOX_19605/KOX_22295	dhsk + pqqh2 <-> pqq + sme
PPNDHG	Phenylalanine, Tyrosine and Tryptophan biosynthesis	prephenate dehydrogenase	1.3.1.12	KOX_00040(tyrA)	nad + phen -> 4hpp + co2 + nadh
ANTPRT	Phenylalanine, Tyrosine and Tryptophan biosynthesis	anthranilate phosphoribosyltransferase	2.4.2.18	KOX_18340	an + prpp <-> ppi + npran
PSHKCVT	Phenylalanine, Tyrosine and Tryptophan biosynthesis	3-phosphoshikimate 1-carboxyvinyltransferase	2.5.1.19	KOX_16000	pep + skm5p <-> 3psme + pi
TYRTA1	Phenylalanine, Tyrosine and Tryptophan biosynthesis	tyrosine transaminase	2.6.1.9	KOX_25040	akg + tyr <-> 4hpp + glu
TYRTA2	Phenylalanine, Tyrosine and Tryptophan biosynthesis	tyrosine transaminase	2.6.1.57	KOX_08310	akg + tyr <-> 4hpp + glu
PHETA	Phenylalanine, Tyrosine and Tryptophan biosynthesis	phenylalanine transaminase	2.6.1.1/2.6.1.9/2.6.1.57	KOX_16370/KOX_25040/KOX_08310	akg + phe <-> glu + phpyr
SHKK	Phenylalanine, Tyrosine and Tryptophan biosynthesis	shikimate kinase	2.7.1.71	KOX_04610(aroK)/KOX_12410(aroL)	atp + sme -> adp + skm5p
IG3PS	Phenylalanine, Tyrosine and Tryptophan biosynthesis	indole-3-glycerol-phosphate synthase	4.1.1.48	KOX_18335	2cpr5p -> 3ig3p + co2
DOXPHS	Phenylalanine, Tyrosine and Tryptophan biosynthesis	3-deoxy-7-phosphoheptulonate synthase	2.5.1.54	KOX_00045	e4p + pep -> 3ddah7p + pi
ANTHS	Phenylalanine, Tyrosine and Tryptophan biosynthesis	anthranilate synthase	4.1.3.27	KOX_18340/KOX_18345	chor + gln -> an + glu + pyr
DHQND	Phenylalanine, Tyrosine and Tryptophan biosynthesis	3-dehydroquinate dehydratase	4.2.1.10	KOX_10820	dqt -> dhsk
TRPS1	Phenylalanine, Tyrosine and Tryptophan biosynthesis	tryptophan synthase	4.2.1.20	KOX_18325(trpA)/KOX_18330	3ig3p + ser -> g3p + trp
TRPS2	Phenylalanine, Tyrosine and Tryptophan biosynthesis	tryptophan synthase	4.2.1.20	KOX_18330	indole + ser -> trp
TRPS3	Phenylalanine, Tyrosine and Tryptophan biosynthesis	tryptophan synthase	4.2.1.20	KOX_18325(trpA)	3ig3p <-> g3p + indole
PPNDHT	Phenylalanine, Tyrosine and Tryptophan biosynthesis	prephenate dehydratase	4.2.1.51	KOX_00030(pheA)/KOX_00340	phen <-> co2 + phpyr
CHORS	Phenylalanine, Tyrosine and Tryptophan biosynthesis	chorismate synthase	4.2.3.5	KOX_26635	3psme -> chor + pi
PRANTI	Phenylalanine, Tyrosine and Tryptophan biosynthesis	phosphoribosylanthranilate isomerase	5.3.1.24	KOX_18335	npran <-> 2cpr5p
CHORM	Phenylalanine, Tyrosine and Tryptophan biosynthesis	chorismate mutase	5.4.99.5	KOX_00030(pheA)/KOX_00040(tyrA)/KOX_18655	chor -> phen

DHQTS	Phenylalanine, Tyrosine and Tryptophan biosynthesis	3-dehydroquinate synthase	4.2.3.4	KOX_04605(aroB)	3ddah7p -> dqt + pi
QTDH1	Phenylalanine, Tyrosine and Tryptophan biosynthesis	quinate dehydrogenase (pyrroloquinoline-quinone)	1.1.5.8	KOX_19605/KOX_22295	qt + pqq <-> dqt + pqqh2
TYRTM	Phenylalanine, Tyrosine and Tryptophan biosynthesis	aromatic-amino-acid transaminase	2.6.1.57	KOX_08310	phen + asp -> oaa + ag
AGDH1	Phenylalanine, Tyrosine and Tryptophan biosynthesis	arogenate/prephenate dehydratase	4.2.1.51	KOX_00030(pheA)/KOX_00340	ag -> phe + co2
AGDH2	Phenylalanine, Tyrosine and Tryptophan biosynthesis	arogenate/prephenate dehydratase	4.2.1.91	KOX_00340	ag -> phe + co2
AGDH3	Phenylalanine, Tyrosine and Tryptophan biosynthesis	arogenate/prephenate dehydratase	4.2.1.91	KOX_00340	phen -> phpyr + co2
PPACALDH	Phosphonate and phosphinate metabolism	phosphonoacetaldehyde hydrolase	3.11.1.1	KOX_06380	ppacald -> acal + pi
PPACH	Phosphonate and phosphinate metabolism	phosphonoacetate hydrolase	3.11.1.2	KOX_08585	ppac -> ac + pi
2AMEPPT	Phosphonate and phosphinate metabolism	2-aminoethylphosphonate-pyruvate transaminase	2.6.1.37	KOX_06375	2amepp + pyr <-> ppacald + ala
PPTCACT	Phosphonate and phosphinate metabolism	phosphinothricin acetyltransferase	2.3.1.183	KOX_19805/KOX_25625	dmppth + accoa -> nadpt + coa
CPPPGO	Porphyrin and Chlorophyll metabolism	coproporphyrinogen oxidase (O2 required)	1.3.3.3	KOX_26935	cpp + o2 -> 2 co2 + pphg
CPPPGOO	Porphyrin and Chlorophyll metabolism	oxygen-independent coproporphyrinogen III oxidase	1.3.99.22	KOX_02720/KOX_06875	cpp + 2 sam -> pphg + 2 co2 + 2 met + 2 da-5
PPHGO	Porphyrin and Chlorophyll metabolism	protoporphyrinogen oxidase (aerobic)	1.3.3.4		3 o2 + 2 pphg -> 2 ppix
PPHGOx	Porphyrin and Chlorophyll metabolism	protoporphyrinogen oxidase (anaerobic)	1.3.3.4		3 fum + pphg -> ppix + 3 succ
PPHGOmq	Porphyrin and Chlorophyll metabolism	menaquinone-dependent protoporphyrinogen oxidase	1.3.5.3	KOX_07855(hemG)	pphg + 3 mk -> ppix + 3 mqn
UPPMT	Porphyrin and Chlorophyll metabolism	uroporphyrinogen methyltransferase	2.1.1.107	KOX_01090/KOX_04575(cysG)/KOX_07615	2 sam + uppg3 -> 2 sah + dscl
CBIAT1	Porphyrin and Chlorophyll metabolism	Cobinamide adenyltransferase	2.5.1.17	KOX_14635/KOX_18380	atp + cbi <-> adcba + pppi
UPPDC1	Porphyrin and Chlorophyll metabolism	uroporphyrinogen decarboxylase (uroporphyrinogen III)	4.1.1.37	KOX_07980(hemE)	uppg3 -> 4 co2 + cpp
PPBINGS	Porphyrin and Chlorophyll metabolism	porphobilinogen synthase	4.2.1.24	KOX_12315	2 5aop -> pbg
HMBS	Porphyrin and Chlorophyll metabolism	hydroxymethylbilane synthase	2.5.1.61	KOX_07625(hemC)	4 pbg -> hmb + 4 nh4
UPPG3S	Porphyrin and Chlorophyll metabolism	uroporphyrinogen-III synthase	4.2.1.75	KOX_07620(hemD)	hmb -> uppg3
HMBUPPG	Porphyrin and Chlorophyll metabolism	spontaneous	spontaneous		hmb -> uppg1
FERCLT	Porphyrin and Chlorophyll metabolism	ferrochelatase	4.99.1.1	KOX_13055(hemH)	fe2 + ppix -> pth
GLU1SAT	Porphyrin and Chlorophyll metabolism	glutamate-1-semialdehyde aminotransferase	5.4.3.8	KOX_11370	glu1sa <-> 5aop
GLUTRS	Porphyrin and Chlorophyll metabolism	Glutamyl-tRNA synthetase	6.1.1.17	KOX_26785(gltX)	atp + glu + trnaglu -> amp + glutrna + ppi
ACOBPGT	Porphyrin and Chlorophyll metabolism	Adenosyl cobinamide phosphate guanyltransferase	2.7.7.62	KOX_01345(cobU)	adcbap + gtp -> agdpcba + ppi
ADCOBK	Porphyrin and Chlorophyll metabolism	Adenosyl cobinamide kinase	2.7.1.156	KOX_01345(cobU)	adcba + atp -> adcbap + adp
ADCOBPS	Porphyrin and Chlorophyll metabolism	Adenosylcobalamin 5'-phosphate synthase	2.7.8.26	KOX_24865(cobS)	agdpcba + rdmbzi -> adocbl + gmp
GLUTRR	Porphyrin and Chlorophyll metabolism	glutamyl-tRNA reductase	1.2.1.70	KOX_23190(hemA)	glutrna + nadph -> glu1sa + nadp + trnaglu
HEMEOS	Porphyrin and Chlorophyll metabolism	Heme O synthase	2.5.1.-	KOX_12700	frdp + pth -> hemeO + ppi
UPPDC2	Porphyrin and Chlorophyll metabolism	uroporphyrinogen decarboxylase	4.1.1.37	KOX_07980(hemE)	uppg1 -> cppi + 4 co2
SRHCC	Porphyrin and Chlorophyll metabolism	sirohydrochlorin cobaltochelatase	4.99.1.3	KOX_01380	shcl + cobalt2 -> cobtpc
CBIAT2	Porphyrin and Chlorophyll metabolism	Cobinamide adenyltransferase	2.5.1.17	KOX_14635/KOX_18380	cobacd + atp -> acda + pppi
ADNCOS	Porphyrin and Chlorophyll metabolism	adenosylcobyric acid synthase	6.3.5.10	KOX_01350	acda + 4 gln + 4 atp -> acha + 4 glu + 4 pi + 4 adp
COBBP1	Porphyrin and Chlorophyll metabolism	cobalamin biosynthetic protein CobC	6.3.1.10	KOX_01425(codD)	atp + acha + amppo -> adp + pi + adcba
COBBP2	Porphyrin and Chlorophyll metabolism	cobalamin biosynthetic protein CobC	6.3.1.10	KOX_01425(codD)	acha + d1ap2oop + atp -> adcbap + adp + pi
DSCLDH	Porphyrin and Chlorophyll metabolism	precorrin-2 dehydrogenase	1.3.1.76	KOX_01090/KOX_04575(cysG)	dscl + nad -> shcl + nadh
SHCLFC	Porphyrin and Chlorophyll metabolism	sirohydrochlorin ferrochelatase	4.99.1.4	KOX_01090/KOX_04575(cysG)	shcl + fe2 -> sheme
COFACMT	Porphyrin and Chlorophyll metabolism	cobalt-factor-2 C20-methyltransferase	2.1.1.151	KOX_01375	cobtpc + sam -> cofac3 + sah
COFACTOPC	Porphyrin and Chlorophyll metabolism	oxidoreduction incomplete reaction (unidentified donor)			cofac3 <-> cobtpc3
COBTPC3MT	Porphyrin and Chlorophyll metabolism	cobalt-precorrin-3B C(17)-methyltransferase	2.1.1.131	KOX_01390(cbiH)	cobtpc3 + sam -> cobtpc4 + sah
COBTPC4MT	Porphyrin and Chlorophyll metabolism	cobalt-precorrin-4 C(11)-methyltransferase	2.1.1.133	KOX_01400(cbiF)	cobtpc4 + sam -> cobtpc5a + sah
COBTPC5AH	Porphyrin and Chlorophyll metabolism	cobalt-precorrin 5A hydrolase	3.7.1.12	KOX_01395	cobtpc5a -> cobtpc5b + acal
COBTPC6S	Porphyrin and Chlorophyll metabolism	cobalt-precorrin-6A synthase	2.1.1.195	KOX_01415(cbiD)	cobtpc5b + sam -> cobtpc6 + sah
COBTPC6RD	Porphyrin and Chlorophyll metabolism	cobalt-precorrin-6x reductase	1.3.1.54	KOX_01385	cobtpc6 + nadph -> cobtpc6b + nadp
COBTPC6YM	Porphyrin and Chlorophyll metabolism	cobalt-precorrin-6Y C(5)-methyltransferase	2.1.1.132	KOX_01410	cobtpc6b + sam -> cobtpc7 + sah
COBTPC7M	Porphyrin and Chlorophyll metabolism	cobalt-precorrin-7 (C15)-methyltransferase	2.1.1.196	KOX_01405	cobtpc7 + sam -> cobtpc8 + sah
COBTPC8M	Porphyrin and Chlorophyll metabolism	cobalt-precorrin-8X methylmutase	5.4.1.2	KOX_01420(chiC)	cobtpc8 -> cobrin
COBRINDIS	Porphyrin and Chlorophyll metabolism	cobyrinic acid a,c-diamide synthase	6.3.5.11	KOX_01430	cobrin + 2 gln + 2 atp -> cobrindi + 2 glu + 2 adp + 2 pi
PC2M	Porphyrin and Chlorophyll metabolism	precorrin-2 C(20)-methyltransferase	2.1.1.130	KOX_01375	dscl + sam -> pc3a + sah
PC3M	Porphyrin and Chlorophyll metabolism	precorrin-3B C17-methyltransferase	2.1.1.131	KOX_01390(cbiH)	pc3b + sam -> pc4 + sah
PC4M	Porphyrin and Chlorophyll metabolism	precorrin-4 C11-methyltransferase	2.1.1.133	KOX_01400(cbiF)	pc4 + sam -> pc5 + sah
PC6XRD	Porphyrin and Chlorophyll metabolism	precorrin-6X reductase	1.3.1.54	KOX_01385	pc6a + nadph -> pc6b + nadp
PC8XMM	Porphyrin and Chlorophyll metabolism	precorrin-8X methylmutase	5.4.1.2	KOX_01420(cbiC)	pc8 -> hgenbyr
HGENBDS	Porphyrin and Chlorophyll metabolism	Hydrogenobyrinate a,c diamide synthase	6.3.5.9	KOX_01430	hgenbyr + 2 gln + 2 atp -> hgenbytdi + 2 pi + 2 glu + 2 adp
FEO2OX	Porphyrin and Chlorophyll metabolism	Fe2+:oxygen oxidoreductase	1.16.3.1	KOX_24070	o2 + 4 fe2 -> 4 fe3
THRPDC	Porphyrin and Chlorophyll metabolism	threonine-phosphate decarboxylase	4.1.1.81	KOX_01550	thro3p -> d1ap2oop + co2
THRAOT	Porphyrin and Chlorophyll metabolism	ATP:L-threonine O-phosphotransferase	2.7.1.-		thr + atp -> thro3p + adp
ETCOBADT	Porphyrin and Chlorophyll metabolism	ethanolamine utilization cobalamin adenosyltransferase	2.5.1.17	KOX_27010	cbl1 + atp -> adocbl + adp
AQCBLRD	Porphyrin and Chlorophyll metabolism	aquacobalamin reductase	1.16.1.3	KOX_07820(fre)	aqcbl3 + nadh -> cbl2 + nad
PPNAK	Propanoate metabolism	Propionate kinase	2.7.2.1	KOX_01540/KOX_26435	adp + ppap <-> atp + ppa
OBUTFL	Propanoate metabolism	2-Oxobutanoate formate lyase	2.3.1.54	KOX_09770/KOX_15225/KOX_15975/KOX_16885(pflD)	obut + coa -> formate + ppcoa
PACTF	Propanoate metabolism	Phosphate acetyltransferase	2.3.1.8	KOX_26440/KOX_01490(pduL)	pi + ppcoa <-> coa + ppap
ENCOAH2	Propanoate metabolism	enoyl-CoA hydratase	4.2.1.17	KOX_07835(fadB)/KOX_19445/KOX_26655(fadJ)	3hpcoa <-> ppecoa
ACCSYN1	Propanoate metabolism	acetyl-CoA synthetase	6.2.1.1	KOX_08465	ppcoa + amp <-> ppald + coa
ACCSYN2	Propanoate metabolism	acetyl-CoA synthetase	6.2.1.1	KOX_08465	ppald + ppi <-> ppa + atp
MMSDH	Propanoate metabolism	methylmalonate-semialdehyde dehydrogenase	1.2.1.27	KOX_09140/KOX_09360	mmsa + coa + nad -> ppcoa + co2 + nadh
L-LACD4	Propanoate metabolism	L-lactate dehydrogenase	1.1.1.27	KOX_21270	2hba + nad -> obut + nadh
PCT2	Propanoate metabolism	propionate CoA-transferase	2.8.3.1	KOX_02010	lactcoa + ppa <-> llac + ppcoa
MCTOP	Propanoate metabolism	unclear reaction			malcoa + nadp <-> 3oppcoa + o2 + nadph
OPTHP	Propanoate metabolism	unclear reaction			3oppcoa + nadph <-> 3hpcoa + nadp
ALHD16	Propanoate metabolism	aldehyde dehydrogenase (NAD+)	1.2.1.3	KOX_00375	2p1a + nad -> ppn + nadh
2MCITDT	Propanoate metabolism	2-methylisocitrate dehydratase	4.2.1.99	KOX_11075	maco <-> micit
MMCOADC	Propanoate metabolism	methylmalonyl-CoA decarboxylase	4.1.1.41	KOX_02550	mmcoa-S -> ppcoa + co2
MMALSADH1	Propanoate metabolism	methylmalonate-semialdehyde dehydrogenase	1.2.1.18	KOX_09140/KOX_09360	3opp + coa + nad -> accoa + co2 + nadh
MMALSADH2	Propanoate metabolism	methylmalonate-semialdehyde dehydrogenase	1.2.1.18	KOX_09140/KOX_09360	3opp + coa + nadp <-> malcoa + nadph
MMALSADH3	Propanoate metabolism	methylmalonate-semialdehyde dehydrogenase	1.2.1.18	KOX_09140/KOX_09360	mmsa + coa + nad -> mmcoa-R + nadh
PPENTM	Purine metabolism	phosphopentomutase	5.4.2.7/5.4.2.2	KOX_10315/KOX_14430	r1p <-> r5p
IMPCH	Purine metabolism	IMP cyclohydrolase	2.1.2.3	KOX_08025(purH)	imp <-> fprica
PRAIZC2	Purine and Pyrimidine Biosynthesis	phosphoribosylaminoimidazole carboxylase			cair <-> caiz
GARTFT	Purine and Pyrimidine Biosynthesis	GAR transformylase-T	2.1.2.-		atp + formate + gar -> adp + fgam + pi
GTPDPK	Purine metabolism	GTP diphosphokinase	2.7.6.5	KOX_01155(relA)	atp + gtp -> amp + pppgpp
G5TDPDP	Purine metabolism	guanosine-5'-triphosphate,3'-diphosphate diphosphatase	3.6.1.11/3.6.1.40	KOX_07535/KOX_27190	pppgpp -> pi + ppgpp
URGLYCH	Purine metabolism	Ureidoglycolate hydrolase	3.5.3.19	KOX_27445	urdglyc -> co2 + glx + 2 nh4
RNDPR1	Purine metabolism	ribonucleoside-diphosphate reductase (ADP)	1.17.4.1	KOX_00440/KOX_00445(nrdF)/KOX_26135/KOX_26140(nrdB)	adp + rthio -> dadp + othio
RNDPR2	Purine metabolism	ribonucleoside-diphosphate reductase (GDP)	1.17.4.1	KOX_00440/KOX_00445(nrdF)/KOX_26135/KOX_26140(nrdB)	gdp + rthio -> dgdp + othio

RNDPR3	Purine metabolism	ribonucleoside-diphosphate reductase (CDP)	1.17.4.1	KOX_00440/KOX_00445(nrdF)/KOX_26135/KOX_26140(nrdB)	cdp + rthio -> dcdp + othio
RNDPR4	Purine metabolism	ribonucleoside-diphosphate reductase (UDP)	1.17.4.1	KOX_00440/KOX_00445(nrdF)/KOX_26135/KOX_26140(nrdB)	rthio + udp -> dudp + othio
PUNPP1	Purine metabolism	purine-nucleoside phosphorylase (Adenosine)	2.4.2.1	KOX_10320(deoD)	adn + pi <-> ad + r1p
PUNPP2	Purine metabolism	purine-nucleoside phosphorylase (Deoxyadenosine)	2.4.2.1	KOX_10320(deoD)	da + pi <-> dr1p + ad
PUNPP3	Purine metabolism	purine-nucleoside phosphorylase (Guanosine)	2.4.2.1	KOX_10320(deoD)	gsn + pi <-> gn + r1p
PUNPP4	Purine metabolism	purine-nucleoside phosphorylase (Deoxyguanosine)	2.4.2.1	KOX_10320(deoD)	dg + pi <-> dr1p + gn
PUNPP5	Purine metabolism	purine-nucleoside phosphorylase (Inosine)	2.4.2.1	KOX_10320(deoD)	ins + pi <-> hyxn + r1p
PUNPP6	Purine metabolism	purine-nucleoside phosphorylase (Deoxyinosine)	2.4.2.1	KOX_10320(deoD)	din + pi <-> dr1p + hyxn
PUNPP7	Purine metabolism	purine-nucleoside phosphorylase (Xanthosine)	2.4.2.1/2.4.2.-	KOX_10320(deoD)/KOX_26800	pi + xtsine <-> r1p + xan
XANPRT	Purine metabolism	xanthine phosphoribosyltransferase	2.4.2.8/2.4.2.22	KOX_11140/KOX_11750	prpp + xan -> ppi + xmp
ADPRT1	Purine metabolism	adenine phosphoribosyltransferase	2.4.2.7	KOX_13025	ad + prpp -> amp + ppi
GNPRT	Purine metabolism	guanine phosphoribosyltransferase	2.4.2.8/2.4.2.22	KOX_11140/KOX_11750	gn + prpp -> gmp + ppi
HYXNPRT	Purine metabolism	hypoxanthine phosphoribosyltransferase (Hypoxanthine)	2.4.2.8	KOX_11140	hyxn + prpp -> imp + ppi
DADNK	Purine metabolism	deoxyadenylate kinase	2.7.4.3	KOX_13050(adk)	atp + damp <-> adp + dadp
ADNK1	Purine metabolism	adenylate kinase	2.7.4.3	KOX_13050(adk)	amp + atp <-> 2 adp
NUDPK1	Purine metabolism	nucleoside-diphosphate kinase (ATP:GDP)	2.7.4.6	KOX_27290(ndk)	atp + gdp <-> adp + gtp
NUDPK5	Purine metabolism	nucleoside-diphosphate kinase (ATP:dGDP)	2.7.4.6	KOX_27290(ndk)	atp + dgdp <-> adp + dgtp
NUDPK8	Purine metabolism	nucleoside-diphosphate kinase (ATP:dADP)	2.7.4.6	KOX_27290(ndk)	atp + dadp <-> adp + datp
DGNK	Purine metabolism	deoxyguanylate kinase (dGMP:ATP)	2.7.4.8	KOX_06000(gmk)	atp + dgmp <-> adp + dgdp
GKN	Purine metabolism	guanylate kinase (GMP:ATP)	2.7.4.8	KOX_06000(gmk)	atp + gmp <-> adp + gdp
NUTD10	Purine metabolism	5'-nucleotidase (XMP)	3.1.3.5	KOX_01045(surE)/KOX_10270/KOX_13075(ushA)/KOX_26410	xmp -> pi + xtsine
NUTD11	Purine metabolism	5'-nucleotidase (IMP)	3.1.3.5	KOX_01045(surE)/KOX_10270/KOX_13075(ushA)/KOX_26410	imp -> ins + pi
NUTD6	Purine metabolism	5'-nucleotidase (dAMP)	3.1.3.5	KOX_01045(surE)/KOX_10270/KOX_13075(ushA)/KOX_26410	damp -> da + pi
NUTD7	Purine metabolism	5'-nucleotidase (AMP)	3.1.3.5	KOX_01045(surE)/KOX_10270/KOX_13075(ushA)/KOX_26410	amp -> adn + pi
NUTD8	Purine metabolism	5'-nucleotidase (dGMP)	3.1.3.5	KOX_01045(surE)/KOX_10270/KOX_13075(ushA)/KOX_26410	dgmp -> dg + pi
NUTD9	Purine metabolism	5'-nucleotidase (GMP)	3.1.3.5	KOX_01045(surE)/KOX_10270/KOX_13075(ushA)/KOX_26410	gmp -> gsn + pi
RNTPR1	Purine metabolism	ribonucleoside-triphosphate reductase	1.17.4.2	KOX_09230	gtp + rthio -> dgtp + othio
RNTPR2	Purine metabolism	ribonucleoside-triphosphate reductase	1.17.4.2	KOX_09230	atp + rthio -> datp + othio
NUTPTP1	Purine metabolism	Nucleoside triphosphate tripolyhydrolase	3.1.5.1	KOX_11400(dgt)	dgtp -> dg + pppi
NUTPTP2	Purine metabolism	Nucleoside triphosphate tripolyhydrolase	3.1.5.1	KOX_11400(dgt)	gtp -> gsn + pppi
AMPNS	Purine metabolism	AMP nucleosidase	3.2.2.4	KOX_24315	amp -> ad + r5p
GNDA	Purine metabolism	guanine deaminase	3.5.4.3	KOX_16755	gn -> nh4 + xan
ADNA	Purine metabolism	Adenosine deaminase	3.5.4.4	KOX_21945	adn -> ins + nh4
ADPRDP	Purine metabolism	ADPribose diphosphatase	3.6.1.13/3.6.1.-	KOX_02975 (nudF)/KOX_04645(nudE)	adprib -> amp + r5p
NUTP1	Purine metabolism	nucleoside-triphosphatase (ATP)	3.6.1.8	KOX_01150(mazG)	atp -> adp + pi
NUTP2	Purine metabolism	nucleoside-triphosphatase (GTP)	3.6.1.8/3.6.1.19	KOX_01150(mazG)/KOX_02715	gtp -> gmp + ppi
NUTP3	Purine metabolism	nucleoside-triphosphatase (dITP)	3.6.1.19	KOX_02715	ditp -> dimp + ppi
NUTP4	Purine metabolism	nucleoside-triphosphatase (ITP)	3.6.1.8/3.6.1.19	KOX_01150(mazG)/KOX_02715	itp -> imp + ppi
NUTP5	Purine metabolism	nucleoside-triphosphatase (XTP)	3.6.1.19	KOX_02715	xtp -> xmp + ppi
NUTP6	Purine metabolism	nucleoside-triphosphatase (ATP)	3.6.1.8	KOX_01150(mazG)	atp -> amp + ppi
NUTP8	Purine metabolism	nucleoside-triphosphatase (ATP)	3.6.1.8	KOX_01150(mazG)	datp -> damp + ppi
NUTP7	Purine metabolism	nucleoside-triphosphatase (dGTP)	3.6.1.19	KOX_02715	dgtp -> dgmp + ppi
ADNCYC	Purine metabolism	adenylate cyclase	4.6.1.1	KOX_07630(cyaA)	atp -> camp + ppi
DANDA	Purine metabolism	Deoxyadenosine deaminase	3.5.4.4	KOX_21945	da -> din + nh4
IMPDH	Purine metabolism	IMP dehydrogenase	1.1.1.205	KOX_27220	imp + nad -> nadh + xmp
PRGNFT	Purine metabolism	phosphoribosylglycinamide formyltransferase	2.1.2.2	KOX_23845(purT)/KOX_27160(purN)	fthf + gar <-> fgam + thf
GARTFM	Purine metabolism	glycinamide ribonucleotide transformylase		KOX_23845(purT)	atp + formate + gar <-> adp + fgam + pi
PRAZCFT	Purine metabolism	phosphoribosylaminoimidazolecarboxamide formyltransferase	2.1.2.3	KOX_08025(purH)	fthf + aicar <-> fprica + thf
GLUPRPAT	Purine metabolism	glutamine phosphoribosyldiphosphate amidotransferase	2.4.2.14	KOX_26550	gln + prpp -> glu + ppi + pram
ADSUCL1	Purine metabolism	adenylsuccinate lyase	4.3.2.2	KOX_17535	asuc <-> amp + fum
ADSUCL2	Purine metabolism	adenylsuccinate lyase	4.3.2.2	KOX_17535	saicar <-> aicar + fum
PRASUCS	Purine metabolism	phosphoribosylaminoimidazolesuccinocarboxamide synthase	6.3.2.6	KOX_27090	cair + asp + atp -> saicar + adp + pi
PRAIZS	Purine metabolism	phosphoribosylaminoimidazole synthase	6.3.3.1	KOX_27155	atp + fpram -> adp + air + pi
PRGCS	Purine metabolism	phosphoribosylglycinamide synthase	6.3.4.13	KOX_08020	atp + gly + pram <-> adp + gar + pi
ADSUCS	Purine metabolism	adenylosuccinate synthase	6.3.4.4	KOX_08870	asp + gtp + imp -> asuc + gdp + pi
GMPS	Purine metabolism	GMP synthase	6.3.5.2	KOX_20300/KOX_26875/KOX_27215(guaA)	atp + gln + xmp -> amp + glu + gmp + ppi
PRFGAS	Purine metabolism	phosphoribosylformylglycinamidine synthase	6.3.5.3	KOX_27495	atp + fgam + gln -> adp + fpram + glu + pi
AIRC	Purine metabolism	phosphoribosylaminoimidazole carboxylase	6.3.4.18	KOX_13195	air + atp + hco3 -> caiz + adp + pi
CAIZM	Purine metabolism	5-(carboxyamino)imidazole ribonucleotide mutase	5.4.99.18	KOX_13200	caiz <-> cair
ADPRT2	Purine metabolism	adenine phosphoribosyltransferase	2.4.2.7	KOX_13025	aicar + ppi <-> 5a4ic + prpp
ADPRT3	Purine metabolism	adenine phosphoribosyltransferase	2.4.2.7	KOX_13025	gmp + ppi <-> gn + prpp
NUDPK9	Purine metabolism	nucleoside-diphosphate kinase (ATP:GDP)	2.7.4.6	KOX_27290(ndk)	atp + didp <-> adp + ditp
NUDPK10	Purine metabolism	nucleoside-diphosphate kinase (ATP:GDP)	2.7.4.6	KOX_27290(ndk)	atp + idp <-> adp + itp
ADPRT4	Purine metabolism	adenine phosphoribosyltransferase	2.4.2.8	KOX_13025	amp + ppi <-> ad + prpp
PYK1	Purine metabolism	pyruvate kinase	2.7.1.40	KOX_22750/KOX_23870	datp + pyr <-> dadp + pep
PYK2	Purine metabolism	pyruvate kinase	2.7.1.40	KOX_22750/KOX_23870	gtp + pyr <-> gdp + pep
PYK3	Purine metabolism	pyruvate kinase	2.7.1.40	KOX_22750/KOX_23870	dgdp + pep -> dgtp + pyr
ADNK2	Purine metabolism	adenylate kinase	2.7.4.3	KOX_13050(adk)	atp + damp <-> adp + dadp
ADNCYC1	Purine metabolism	adenylate cyclase	4.6.1.1	KOX_07630(cyaA)	gtp -> cgmp + ppi
23CNPDE1	Purine metabolism	2',3'-cyclic-nucleotide 2'-phosphodiesterase	3.1.4.16	KOX_09030(cpdB)	23cgmp -> 3gmp
23CNPDE2	Purine metabolism	2',3'-cyclic-nucleotide 2'-phosphodiesterase	3.1.4.16	KOX_09030(cpdB)	23camp -> 3amp
GSK	Purine metabolism	inosine/guanosine kinase	2.7.1.73	KOX_13060	gsn + atp <-> gmp + adp
INSK	Purine metabolism	inosine/guanosine kinase	2.7.1.73	KOX_13060	ins + atp <-> imp + adp
GMPRD	Purine metabolism	GMP reductase	1.7.1.7	KOX_10990	gmp + nadph -> imp + nadp + nh4
ADDA	Purine metabolism	adenine deaminase	3.5.4.2	KOX_25530	ad -> hyxn + nh4
SADT1	Purine metabolism	sulfate adenylyltransferase	2.7.7.4	KOX_01085	atp + so4 -> ppi + aps
UREA	Purine metabolism	urease	3.5.1.5	KOX_03085(ureA)/KOX_03090(ureB)/KOX_0309(ureC)	urea -> co2 + 2 nh4
5HISOUH	Purine metabolism	5-hydroxyisourate hydrolas	3.5.2.17	KOX_16750/KOX_23355	5hiu -> 5h2o4uic
ALLTD	Purine metabolism	allantoate deiminase	3.5.3.9	KOX_16830	alltt -> ureidogly + nh4 + co2
CAPK	Purine metabolism	carbamate kinase	2.7.2.2	KOX_23275	cap + adp -> atp + nh4 + co2
BTETP	Purine metabolism	bis(5'-nucleosyl)-tetraphosphatase (symmetrical)	3.6.1.41	KOX_10685(apaH)	appppa -> 2 adp

ALLTNtr	Putative Transporters	allantoin transport in via proton symport			alltn_e + hext <-> alltn
ARGORNt	Putative Transporters	arginine/ornithine antiporter			arg_e + orn <-> arg + orn_e
ACACt	Putative Transporters	acetoacetate transport via proton symport			acac_e + hext <-> acac
BUTtr	Putative Transporters	Butyrate transport via proton symport, reversible			1boh_e + hext <-> 1boh
GALCTtr	Putative Transporters	D-galactarte transport via proton symport, reversible			dgal_e + hext <-> dgal
DGLUCAt	Putative Transporters	D-glucarate transport via proton symport, reversible			dgluca_e + hext <-> dgluca
PPPNtr	Putative Transporters	3-phenylpropionate transport via proton symport, reversible			hext + pppn_e <-> pppn
HPPPNtr	Putative Transporters	3-(3-hydroxyphenyl)propionate transport via proton symport, reversible			3hpppn_e + hext <-> 3hpppn
HCINNMtr	Putative Transporters	3-hydroxycinnamic acid transport via proton symport, reversible			3hcinnm_e + hext <-> 3hcinnm
GLUABUTt	Putative Transporters	4-aminobutyrate/glutamate antiport			gaba + glu_e <-> gaba_e + glu
ALAtr	Putative Transporters	L-alanine reversible transport via proton symport			ala_e + hext <-> ala
URAtr	Putative Transporters	uracil transport in via proton symport, reversible			hext + ura_e <-> ura
GLYBtr	Putative Transporters	Glycine betaine transport via proton symport, reversible			glyb_e + hext <-> glyb
CHLabc	Putative Transporters	choline transport via ABC system			atp + choline_e -> adp + choline + pi
GLYBabc	Putative Transporters	Glycine betaine transport via ABC system			atp + glyb_e -> adp + glyb + pi
TARTRt	Putative Transporters	Tartrate/succinate antiporter			succ + tartr_e <-> succ_e + tartr
SUCCabc	Putative Transporters	Succinate transport via ABC system			atp + succ_e -> adp + pi + succ
GUAt2	Putative Transporters	guanine transport in via proton symport			gn_e + hext -> gn
XANt2	Putative Transporters	xanthine transport in via proton symport			hext + xan_e -> xan
IDONOX	Alternate Carbon Metabolism	L-Idonate:NAD oxidoreductase		KOX_00855	idon + nad <-> 5dhgluc + nadh
IDONOXp	Alternate Carbon Metabolism	L-Idonate:NADP oxidoreductase		KOX_00855	idon + nadp <-> 5dhgluc + nadph
5KDGR	Alternate Carbon Metabolism	5-keto-D-gluconate 5-reductase	1.1.1.169	KOX_00845/KOX_12365	5dhgluc + nadph <-> gluc + nadp
PCT4	Alternate Carbon Metabolism	propionate CoA-transferase	2.8.3.1	KOX_02010	ppcoa + succ -> ppa + succoa
5DHGOX	Alternate Carbon Metabolism	5-dehydro-D-gluconate:NADP+ 2-oxidoreductase	1.1.1.215	KOX_05560	25dhglucn + nadh -> 5dhgluc + nad
5DHGOXp	Alternate Carbon Metabolism	5-dehydro-D-gluconate:NADP+ 2-oxidoreductase	1.1.1.215	KOX_05560	25dhglucn + nadph -> 5dhgluc + nadp
THMDS	Pyrimidine metabolism	thymidylate synthase	2.1.1.45	KOX_01605(thyA)	dump + metthf -> dhf + dtmp
THMDPP	Pyrimidine metabolism	thymidine phosphorylase	2.4.2.4	KOX_10310(deoA)	pi + thymd <-> dr1p + thym
URAPRT	Pyrimidine metabolism	uracil phosphoribosyltransferase	2.4.2.9	KOX_27140	prpp + ura <-> ppi + ump
CYTDK1	Pyrimidine metabolism	cytidylate kinase (CMP)	2.7.4.14	KOX_16005(cmk)	atp + cmp <-> adp + cdp
CYTDK2	Pyrimidine metabolism	cytidylate kinase (dCMP)	2.7.4.14	KOX_16005(cmk)	atp + dcmp <-> adp + dcdp
UMPK	Pyrimidine metabolism	UMP kinase	2.7.4.14	KOX_16005(cmk)	atp + ump <-> adp + udp
NUDPK2	Pyrimidine metabolism	nucleoside-diphosphate kinase (ATP:UDP)	2.7.4.6	KOX_27290(ndk)	atp + udp <-> adp + utp
NUDPK3	Pyrimidine metabolism	nucleoside-diphosphate kinase (ATP:CDP)	2.7.4.6	KOX_27290(ndk)	atp + cdp <-> adp + ctp
NUDPK4	Pyrimidine metabolism	nucleoside-diphosphate kinase (ATP:dTDP)	2.7.4.6	KOX_27290(ndk)	atp + dtdp <-> adp + dttp
NUDPK6	Pyrimidine metabolism	nucleoside-diphosphate kinase (ATP:dUDP)	2.7.4.6	KOX_27290(ndk)	atp + dudp <-> adp + dutp
NUDPK7	Pyrimidine metabolism	nucleoside-diphosphate kinase (ATP:dCDP)	2.7.4.6	KOX_27290(ndk)	atp + dcdp <-> adp + dctp
DTMPK	Pyrimidine metabolism	dTMP kinase	2.7.4.9	KOX_17315(tmk)	atp + dtmp <-> adp + dtdp
NUTD1	Pyrimidine metabolism	5'-nucleotidase (dUMP)	3.1.3.5	KOX_01045(surE)/KOX_10270/KOX_13075(ushA)/ KOX_26410	dump -> du + pi
NUTD2	Pyrimidine metabolism	5'-nucleotidase (UMP)	3.1.3.5	KOX_01045(surE)/KOX_10270/KOX_13075(ushA)/ KOX_26410	ump -> pi + uri
NUTD3	Pyrimidine metabolism	5'-nucleotidase (dCMP)	3.1.3.5	KOX_01045(surE)/KOX_10270/KOX_13075(ushA)/ KOX_26410	dcmp -> dc + pi
NUTD4	Pyrimidine metabolism	5'-nucleotidase (CMP)	3.1.3.5	KOX_01045(surE)/KOX_10270/KOX_13075(ushA)/ KOX_26410	cmp -> cytd + pi
NUTD5	Pyrimidine metabolism	5'-nucleotidase (dTMP)	3.1.3.5	KOX_01045(surE)/KOX_10270/KOX_13075(ushA)/ KOX_26410	dtmp -> pi + thymd
CTDA	Pyrimidine metabolism	Cytosine deaminase	3.5.4.1	KOX_04555/KOX_14050	ct -> nh4 + ura
DCTPDA1	Pyrimidine metabolism	dCTP deaminase	3.5.4.13	KOX_25240(dcd)	dctp -> dutp + nh4
DCTPDA2	Pyrimidine metabolism	dCTP deaminase	3.5.4.13	KOX_25240(dcd)	ctp -> utp + nh4
DUTPDP	Pyrimidine metabolism	dUTP diphosphatase	3.6.1.23/3.6.1.19	KOX_05955(dut)/KOX_02715	dutp -> dump + ppi
PUNPP8	Pyrimidine metabolism	purine nucleoside phosphorylase	2.4.2.1	KOX_10320(deoD)	du + pi <-> dr1p + ura
URIDK1	Pyrimidine metabolism	uridylate kinase (dUMP)	2.7.4.9	KOX_17315(tmk)	atp + dump <-> adp + dudp
DOROAD	Pyrimidine metabolism	dihydroorotate dehydrogenase (fumarate)	1.3.98.1	KOX_16135	doroa + fum <-> oroa + succ
ASPCBT	Pyrimidine metabolism	aspartate carbamoyltransferase	2.1.3.2	KOX_09285/KOX_09290(pyrB)	asp + cap -> caasp + pi
OROPRT	Pyrimidine metabolism	orotate phosphoribosyltransferase	2.4.2.10	KOX_05965	omp + ppi <-> oroa + prpp
DHORT	Pyrimidine metabolism	dihydroorotase	3.5.2.3	KOX_09185/KOX_17195	doroa <-> caasp
OMPDC	Pyrimidine metabolism	orotidine-5'-phosphate decarboxylase	4.1.1.23	KOX_18440	omp -> co2 + ump
CTPS	Pyrimidine metabolism	CTP synthase (glutamine)	6.3.4.2	KOX_01145(pyrG)	atp + gln + utp -> adp + ctp + glu + pi
URIDK2	Pyrimidine metabolism	uridylate kinase	2.7.4.22	KOX_11455(pyrH)	atp + ump <-> adp + udp
DHPM	Pyrimidine metabolism	dihydropyrimidinase	3.5.2.2	KOX_23270	56dhu <-> 3urdpp
CYTDA	Pyrimidine metabolism	cytosine deaminase	3.5.4.1	KOX_04555/KOX_14050	5mc <-> thym + nh4
DHPMD	Pyrimidine metabolism	dihydropyrimidinase	3.5.2.2	KOX_23270	56dht <-> 3udsb
URIK	Pyrimidine metabolism	uridine kinase (GTP)	2.7.1.48	KOX_25245	gtp + uri -> gdp + ump
CYTDK	Pyrimidine metabolism	cytidine kinase (GTP)	2.7.1.48	KOX_25245	cytd + gtp -> cmp + gdp
RNTPR3	Pyrimidine metabolism	ribonucleoside-triphosphate reductase	1.17.4.2	KOX_09230	ctp + rthio -> dctp + othio
RNTPR4	Pyrimidine metabolism	ribonucleoside-triphosphate reductase	1.17.4.2	KOX_09230	utp + rthio -> dutp + othio
URITPP	Pyrimidine metabolism	Uridine triphosphate pyrophosphohydrolase	3.6.1.19	KOX_02715	utp -> ump + ppi
DTTPP	Pyrimidine metabolism	Deoxythymidine triphosphate pyrophosphohydrolase	3.6.1.19	KOX_02715	dttp -> dtmp + ppi
CYTDDA1	Pyrimidine metabolism	cytidine deaminase	3.5.4.5	KOX_25715	cytd -> uri + nh4
CYTDDA2	Pyrimidine metabolism	cytidine deaminase	3.5.4.5	KOX_25715	dc -> du + nh4
23CNPDE3	Pyrimidine metabolism	2',3'-cyclic-nucleotide 2'-phosphodiesterase	3.1.4.16	KOX_09030(cpdB)	23ccmp -> 3cmp
23CNPDE4	Pyrimidine metabolism	2',3'-cyclic-nucleotide 2'-phosphodiesterase	3.1.4.16	KOX_09030(cpdB)	23cump -> 3ump
DURIDK	Pyrimidine metabolism	deoxyuridine kinase (ATP:Deoxyuridine)	2.7.1.21	KOX_23030	du + atp -> dump + adp
THYMDK	Pyrimidine metabolism	deoxyuridine kinase (ATP:Thymidine)	2.7.1.21	KOX_23030	thym + atp -> dtmp + adp
URIPP	Pyrimidine metabolism	uridine phosphorylase	2.4.2.3	KOX_07495/KOX_07765	uri + pi <-> ura + r1p
ATPDP1	Pyrimidine metabolism	ATP diphosphatase	3.6.1.8	KOX_01150(mazG)	udpg_e -> ump + g1p
ATPDP2	Pyrimidine metabolism	ATP diphosphatase	3.6.1.8	KOX_01150(mazG)	utp_e -> ump + ppi
ATPDP3	Pyrimidine metabolism	ATP diphosphatase	3.6.1.8	KOX_01150(mazG)	ctp_e -> cmp + ppi
NUTP9	Pyrimidine metabolism	ATP diphosphatase	3.6.1.8	KOX_01150(mazG)	ctp -> cmp + ppi
NUTP10	Pyrimidine metabolism	Nucleoside triphosphate pyrophosphorylase	3.6.1.8	KOX_01150(mazG)	dctp -> dcmp + ppi
ALHD1	Pyruvate Metabolism	aldehyde dehydrogenase (acetaldehyde, NAD)	1.2.1.3	KOX_00375	acal + nad -> ac + nadh
PPS	Pyruvate Metabolism	phosphoenolpyruvate synthase	2.7.9.2	KOX_22850	atp + pyr -> amp + pep + pi
LALDR	Pyruvate Metabolism	lactaldehyde reductase	1.1.1.77	KOX_00865	llald + nadh <-> 12ppd-R + nad
HAGTH	Pyruvate Metabolism	hydroxyacylglutathione hydrolase	3.1.2.6	KOX_11650	ltg -> rgt + lac
MGXS	Pyruvate Metabolism	methylglyoxal synthase	4.2.3.3	KOX_16225	dhap -> mtg + pi
LGTTHL	Pyruvate Metabolism	lactoylglutathione lyase	4.4.1.5	KOX_02000/KOX_22100/KOX_22260	rgt + mtg -> ltg
MTGRD	Pyruvate Metabolism	gyoxylate/hydroxypyruvate reductase	1.1.1.79	KOX_00275/KOX_17100(ghrA)	mtg + nadh <-> dlald + nad
ALCDet	Pyruvate Metabolism	alcohol dehydrogenase (ethanol)	1.1.1.1	KOX_19595/KOX_20090/KOX_23025	eth + nad <-> acal + nadh
ADHEr	Pyruvate Metabolism	Acetaldehyde dehydrogenase			accoa + 2 nadh <-> coa + eth + 2 nad
DLDHD	Pyruvate Metabolism	D-lactate dehydrogenase	1.1.1.28	KOX_19165/KOX_25620	lac + nad <-> nadh + pyr
ACALDDH	Pyruvate Metabolism	acetaldehyde dehydrogenase (acetylating)	1.2.1.10	KOX_22675/KOX_23025/KOX_26990	acal + coa + nad <-> accoa + nadh
LCTAD2	Pyruvate Metabolism	lactaldehyde dehydrogenase	1.2.1.22	KOX_19535	mtg + nad <-> pyr + nadh

PTA	Pyruvate Metabolism	phosphotransacetylase	2.3.1.8	KOX_26440	accoa + pi <-> actp + coa
ACKA	Pyruvate Metabolism	acetate kinase	2.7.2.1	KOX_01540/KOX_26435	ac + atp <-> actp + adp
PCT1	Pyruvate Metabolism	propionate CoA-transferase	2.8.3.1	KOX_02010	accoa + ppa <-> ac + ppcoa
APPS2	Pyruvate Metabolism	acylphosphatase	3.6.1.7	KOX_16295	actp -> ac + pi
ACS	Pyruvate Metabolism	acetyl-CoA synthetase	6.2.1.1	KOX_08465	ac + atp + coa -> accoa + amp + ppi
ALHD1p	Pyruvate Metabolism	aldehyde dehydrogenase (acetaldehyde, NAD)	1.2.1.3		acal + nadp -> ac + nadph
PFL	Pyruvate Metabolism	formate C-acetyltransferase	2.3.1.54	KOX_09770/KOX_15225/KOX_15975/KOX_16885(pflD)	pyr + coa -> formate + accoa
POX2	Pyruvate Metabolism	pyruvate dehydrogenase (quinone)	1.2.5.1	KOX_15775	pyr + uq -> ac + uqh2 + co2
PTA2	Pyruvate Metabolism	phosphotransacetylase		KOX_27005(eutD)	accoa + pi <-> actp + coa
A6PRAUR	Rivoflavin metabolism	5-amino-6-(5-phosphoribosylamino)uracil reductase	1.1.1.193	KOX_12575(ribD)	a6rp5p + nadph -> a6rp5p2 + nadp
FLVRp	Rivoflavin Metabolism	riboflavin reductase (NADP)	1.5.1.30	KOX_07820(fre)	nadph + ribflav -> nadp + rbflvrd
FLVR	Rivoflavin Metabolism	riboflavin reductase (NAD)	1.5.1.30	KOX_07820(fre)	nadh + ribflav -> nad + rbflvrd
DMLZS	Rivoflavin metabolism	6,7-dimethyl-8-ribityllumazine synthase	2.5.1.78	KOX_12580(ribH)	a6rp + db4p -> dmlz + pi
RBFS	Rivoflavin metabolism	riboflavin synthase	2.5.1.9	KOX_22155	2 dmlz -> a6rp + ribflav
APPT	Rivoflavin metabolism	acid phosphatase	3.1.3.2	KOX_08315(aphA)/KOX_14030/KOX_18070	fmn -> ribflav + pi
RBFK	Rivoflavin metabolism	riboflavin kinase	2.7.1.26	KOX_10525	atp + ribflav -> adp + fmn
FMNANT	Rivoflavin metabolism	FMN adenylyltransferase	2.7.7.2	KOX_10525	atp + fmn -> fad + ppi
DHPPRAP	Rivoflavin metabolism	diaminohydroxyphosphoribosyl aminopryrimidine deaminase (25drapp)	3.5.4.26	KOX_12575(ribD)	25drapp -> a6rp5p + nh4
DHB4PS	Rivoflavin metabolism	3,4-Dihydroxy-2-butanone-4-phosphate synthase	4.1.99.12	KOX_03025(ribB)	rl5p -> db4p + formate
GTPCHII	Rivoflavin metabolism	GTP cyclohydrolase II	3.5.4.25	KOX_18420(ribA)	gtp -> 25drapp + formate + ppi
NNDMBZPT	Rivoflavin metabolism	Nicotinate-nucleotide dimethylbenzimidazole phosphoribosyltransferase	2.4.2.21	KOX_01340(cobT)	dmbzid + nacn -> 5prdmbz + nac
PMPP	Rivoflavin metabolism	pyrimidine phosphatase	3.1.3.-		a6rp5p2 -> a6rp + pi
RZ5PP	Rivoflavin metabolism	alpha-ribazole 5-phosphate phosphatase	3.1.3.73	KOX_14245	5prdmbz -> pi + rdmbzi
FMNRDp	Rivoflavin metabolism	NADPH-dependent FMN reductase	1.5.1.38	KOX_16130	fmn + nadph -> fmnh2 + nadp
FMNRD	Rivoflavin metabolism	NADH-dependent FMN reductase	1.5.1.41	KOX_07820(fre)	fmn + nadh -> fmnh2 + nad
SELNPS	Selenocompound metabolism	Selenophosphate synthase	2.7.9.3	KOX_18075	atp + seld -> amp + pi + selnp
CYSGS1	Selenocompound metabolism	cystathionine gamma-synthase	2.5.1.48	KOX_07310	ahser + scys -> sllct + ac
CYSGS2	Selenocompound metabolism	cystathionine gamma-synthase	2.5.1.48	KOX_07310	shser + scys -> sllct + succ
CYSGS3	Selenocompound metabolism	cystathionine gamma-synthase	2.5.1.48	KOX_07310	phser + scys -> sllct + pi
CYSTBL3	Selenocompound metabolism	cystathionine beta-lyase	4.4.1.8	KOX_02875/KOX_21920	sllct -> shcys + nh4 + pyr
ADHC2	Selenocompound metabolism	adenosylhomocysteinase	3.3.1.1	KOX_24860	seadseh -> adn + shcys
SADMET	Selenocompound metabolism	S-adenosylmethionine synthetase	2.5.1.6	KOX_02650	atp + smet -> pi + ppi + seasmet
METTRSS	Selenocompound metabolism	methionyl-tRNA synthetase	6.1.1.10	KOX_25560(metG)	atp + smet + trnamet -> amp + ppi + selmtrna
CYSST4	Selenocompound metabolism	cysteine synthase	2.5.1.47	KOX_12795/KOX_18690/KOX_26835/KOX_26885(cysM)	aser + seld -> scys + ac
SULFR	Selenocompound metabolism	sulfite reductase (NADPH)	1.8.1.9	KOX_15885	selt + 3 nadp -> seld + 3 nadph
ADSLFK2	Selenocompound metabolism	adenylyl-sulfate kinase	2.7.1.25	KOX_01075	atp + aselnt -> adp + ppadsel
SMTTGH	Selenocompound metabolism	5-methyltetrahydropteroyltriglutamate--homocysteine methyltransferase	2.1.1.14	KOX_07750/KOX_08065(metH)/KOX_21350/KOX_21375	shcys + 5mtglu -> smet + tglu
CYSTHL	Selenocompound metabolism	cystathionine gamma-lyase	4.4.1.1	KOX_02800	smet -> metseln + nh4 + obut
SELNCYS1	Selenocompound metabolism	selenocysteine lyase	2.8.1.7/4.4.1.16	KOX_18715/KOX_22770	mslencys -> pyr + nh4 + metseln
STRNAST	Selenocompound metabolism	L-seryl-tRNA(Ser) seleniumtransferase	2.9.1.1	KOX_02440/KOX_05740/KOX_09190	sectrna + selnp -> selncystrna + pi
SELNCYS2	Selenocompound metabolism	selenocysteine lyase	4.4.1.16	KOX_18715/KOX_22770	scys + fadh2 <-> seld + fad + ala
THRDOX	Selenocompound metabolism	thioredoxin reductase (NADPH)	1.8.1.9	KOX_15885	metselnt + 2 nadph -> metseln + 2 nadp
SELTOXR	Selenocompound metabolism	selenite:reduced acceptor oxidoreductase	1.97.1.9	KOX_21550/KOX_23285	selnt + fadh2 -> selt + fad
SELTGLUT	Selenocompound metabolism	spontaneous			selt + 4 rgt -> selngluth + ogt
SELNGLUT	Selenocompound metabolism	spontaneous			selngluth + rgt -> gluthsel + ogt
TRHPS	Starch and sucrose metabolism	alpha,alpha-trehalose-phosphate synthase (UDP-forming)	2.4.1.15	KOX_24010	g6p + udpg -> tre6p + udp
AMMALT1	Starch and sucrose metabolism	Amylomaltase (maltotriose)	2.4.1.25	KOX_04675(malQ)	mlt + mlttr -> glc + mltttr
AMMALT2	Starch and sucrose metabolism	Amylomaltase (maltotetraose)	2.4.1.25	KOX_04675(malQ)	mlt + mltttr -> glc + maltpt
AMMALT3	Starch and sucrose metabolism	Amylomaltase (maltopentaose)	2.4.1.25	KOX_04675(malQ)	mlt + maltpt -> glc + mlthx
AMMALT4	Starch and sucrose metabolism	Amylomaltase (maltohexaose)	2.4.1.25	KOX_04675(malQ)	mlt + mlthx -> glc + malthp
TRH6PP	Starch and sucrose metabolism	trehalose-phosphatase	3.1.3.12	KOX_24015	tre6p -> pi + tre
TREHL	Starch and sucrose metabolism	alpha,alpha-trehalase	3.2.1.28	KOX_05310(treF)/KOX_23470(treA)	tre -> 2 glc
TREHLe	Starch and sucrose metabolism	alpha,alpha-trehalase	3.2.1.28	KOX_05310(treF)/KOX_23470(treA)	tre_e -> 2 glc_e
GLCGP1	Starch and sucrose metabolism	glycogen phosphorylase	2.4.1.1	KOX_04680/KOX_04715	starch + pi -> amylose + g1p
GLCGP2	Starch and sucrose metabolism	glycogen phosphorylase	2.4.1.1	KOX_04680/KOX_04715	glycogen + pi -> g1p
GLCBRAN2	Starch and sucrose metabolism	1,4-alpha-glucan branching enzyme (glycogen -> bglycogen)	2.4.1.18	KOX_04735	glycogen -> bglycogen
MLTGCT	Starch and sucrose metabolism	maltose alpha-D-glucosyltransferase	5.4.99.16	KOX_22195	tre_e <-> mlt_e
LEVANS	Starch and sucrose metabolism	levanase	3.2.1.65	KOX_13550	levan_mn <-> levan_m + levan_n
FRUFSD	Starch and sucrose metabolism	beta-fructofuranosidase	3.2.1.26	KOX_01765/KOX_07150/KOX_13360	suc6p <-> fru + g6p
GLUSDASE1	Starch and sucrose metabolism	beta-glucosidase	3.2.1.21	KOX_00740/KOX_01670/KOX_18370/KOX_21960/KOX_23455/KOX_25615	glusd -> glc
GLUSDASE2	Starch and sucrose metabolism	beta-glucosidase	3.2.1.21	KOX_00740/KOX_01670/KOX_18370/KOX_21960/KOX_23455/KOX_25615	cellobiose -> 2 glc
GLUSDASE3	Starch and sucrose metabolism	beta-glucosidase	3.2.1.21	KOX_00740/KOX_01670/KOX_18370/KOX_21960/KOX_23455/KOX_25615	cellulose_n -> cellulose_n_1 + glc
ENDOGLUC	Starch and sucrose metabolism	endoglucanase	3.2.1.4		cellulose_n -> cellulose_n_1 + cellobiose
G1PADTRA	Starch and sucrose metabolism	glucose-1-phosphate adenylyltransferase	2.7.7.27	KOX_04725(glgC)	atp + g1p -> adpglc + ppi
GLYSYN1	Starch and sucrose metabolism	glycogen synthase/starch synthase	2.4.1.21	KOX_04720(glgA)	adpglc -> glycogen + adp
GLYSYN2	Starch and sucrose metabolism	glycogen synthase/starch synthase	2.4.1.21	KOX_04720(glgA)	adpglc -> amylose + adp
TRE6PH	Starch and sucrose metabolism	maltose-6'-phosphate glucosidase/trehalose-6-phosphate hydrolase	3.2.1.122/3.2.1.93	KOX_06480/KOX_09255	tre6p -> glc + g6p
MLT6PG	Starch and sucrose metabolism	maltose-6'-phosphate glucosidase	3.2.1.122	KOX_06480	mlt6p <-> glc + g6p
AMDS4	Styrene degradation	amidase	3.5.1.4	KOX_09850/KOX_13720/KOX_20510	pheact -> pac + nh4
PHEALDD	Styrene degradation	phenylacetaldehyde dehydrogenase	1.2.1.39	KOX_19195	pacald + nad -> pac + nadh
PHEACTHL	Styrene degradation	2-phenylacetamide hydro-lyase (nitrile-forming)	4.2.1.84	KOX_20500/KOX_20505	pheacnit -> pheact
ACLMHL	Styrene degradation	acrylamide hydro-lyase	4.2.1.84	KOX_20500/KOX_20505	aconit -> aclm
AMDS5	Styrene degradation	amidase	3.5.1.4	KOX_09850/KOX_13720/KOX_20510	aclm -> propen + nh4
PCT3	Styrene degradation	propionate CoA-transferase	2.8.3.1	KOX_02010	lactcoa + ac -> llac + accoa
SLFR	Sulfur Metabolism	sulfite reductase	1.8.1.2	KOX_01115/KOX_01120(cysJ)	3 nadph + so3 -> h2s + 3 nadp
PASR1	Sulfur Metabolism	phosphoadenylyl-sulfate reductase (thioredoxin)	1.8.4.8	KOX_01110	paps + rthio -> pap + so3 + othio
ADSLFK1	Sulfur Metabolism	adenylyl-sulfate kinase	2.7.1.25	KOX_01075	aps + atp -> adp + paps
BPNT	Sulfur Metabolism	3',5'-bisphosphate nucleotidase	3.1.3.7	KOX_09035	pap -> amp + pi
BPNT2	Sulfur Metabolism	3',5'-bisphosphate nucleotidase	3.1.3.7	KOX_09035	paps -> aps + pi
TAUDO	Taurine and Hypotaurine metabolism	Taurine dioxygenase	1.14.11.17	KOX_12300(tauD)	akg + o2 + taur -> aacald + co2 + so3 + succ
GLUTPEPT	Taurine and Hypotaurine metabolism	gamma-glutamyltranspeptidase	2.3.2.2	KOX_04780(ggt)/KOX_16795	pept + glutaur -> glupept + taur
TRAKGTA	Taurine and Hypotaurine metabolism	taurine---2-oxoglutarate transaminase	2.6.1.55	KOX_13775	taur + akg -> sulald + glu
THMPDP	Thiamine Metabolism	thiamine-phosphate diphosphorylase	2.5.1.3	KOX_07960	ahmpp + thzp -> ppi + thmp
HMPMK	Thiamine Metabolism	hydroxymethylpyrimidine kinase (ATP)	2.7.1.49	KOX_25505	ahm + atp -> 4ampm + adp
HETHZK	Thiamine Metabolism	hydroxyethylthiazole kinase	2.7.1.50	KOX_25510	4mhetz + atp -> thzp + adp
THMPK	Thiamine Metabolism	thiamine-phosphate kinase	2.7.4.16	KOX_12590	atp + thmp -> adp + thmpp
PMPMK	Thiamine Metabolism	phosphomethylpyrimidine kinase	2.7.4.7	KOX_25505	4ampm + atp -> ahmpp + adp
THMPT	Thiamine Metabolism	phosphatase	3.1.3.-		thiamin + pi <-> thmp
THMK	Thiamine Metabolism	thiamine kinase	2.7.1.89	KOX_17355(thiK)	thiamin + atp -> thmp + adp
THMB	Thiamine metabolism	thiamine biosynthesis protein ThiC	unclear reaction	KOX_07965	air -> ahm
THIZPSN	Thiamine Metabolism	thiazole phosphate synthesis			atp + cys + dx5p + tyr -> 4hbza + thzp + ala + amp + co2 + ppi
THMHD	Thiamine Metabolism	Thiamin hydrolase	3.5.99.2	KOX_20340/KOX_20695	thiamin -> ahm + 4mhetz
HMCMCTI	Toluene and Xylene degradation	2-Hydroxy-5-methyl-cis,cis-muconate 2-Oxo-5-methyl-cis-muconate isomerase	5.3.2.-	KOX_02090/KOX_19920/KOX_20805	hmcmct -> omcmc
KPENH	Toluene and Xylene degradation	2-keto-4-pentenoate hydratase	4.2.1.80	KOX_22680(mhpD)	hchdn -> hohx

4H2OVA	Toluene and Xylene degradation	4-hydroxy 2-oxovalerate aldolase	4.1.3.39	KOX_22670	hohx -> propanal + pyr
NITRDT1	Trinitrotoluene degradation	nitroreductase	1.-.-.-	KOX_13625/KOX_15665/KOX_22095	tnittol + 2 nadh -> 4hlmdnit + 2 nad
NITRDT2	Trinitrotoluene degradation	nitroreductase	1.-.-.-	KOX_13625/KOX_15665/KOX_22095	tnittol + 2 nadh -> 2hlmdnit + 2 nad
CARMNOXD	Trinitrotoluene degradation	carbon-monoxide dehydrogenase	1.2.99.2	KOX_23335	24danit -> 24da6hat
TRPPA1	Tryptophan metabolism	Tryptophanase (L-tryptophan)	4.1.99.1	KOX_09730(tnaA)	trp <-> indole + nh4 + pyr
TRPPA2	Tryptophan metabolism	Tryptophanase (L-cysteine)	4.1.99.1	KOX_09730(tnaA)	cys -> indole + h2s + pyr
IDPD	Tryptophan metabolism	indolepyruvate decarboxylase	4.1.1.74	KOX_26745	idpyr -> i3aa + co2
ALHD8	Tryptophan metabolism	aldehyde dehydrogenase (NAD+)	1.2.1.3	KOX_00375	i3aa + nad -> i3ac + nadh
ALHD9	Tryptophan metabolism	aldehyde dehydrogenase (NAD+)	1.2.1.3	KOX_00375	5hiaa + nad -> 5hiac + nadh
CATL	Tryptophan metabolism	catalase	1.11.1.6/1.11.1.21	KOX_18225(katE)/KOX_26215/KOX_20220	2 3han + 2 o2 -> cvn + 2 h2o2
OGDH3	Tryptophan metabolism	2-oxoglutarate dehydrogenase E1 component	1.2.4.2	KOX_14590(sucA)	2oad + nad + coa -> glutcoa + co2 + nadh
ID3ACTHL	Tryptophan metabolism	Indole-3-acetamide hydro-lyase	4.2.1.84	KOX_20500/KOX_20505	id3act -> idactn
4HPHEA1	Tyrosine metabolism	4-hydroxyphenylacetate-3-hydroxylase	1.14.14.9	KOX_10010(hpaC)/KOX_10015	hpheac + o2 + nadh -> 34dhpheac + nad
4HPHEA2	Tyrosine metabolism	4-hydroxyphenylacetate-3-hydroxylase	1.14.14.9	KOX_10010(hpaC)/KOX_10015	4hpheac + o2 + nadh -> 34dhpheac + nad
MNAO3	Tyrosine metabolism	monoamine oxidase	1.4.3.21	KOX_19410(tynA)	o2 + tym -> 4hac + h2o2 + nh4
34DHPACD	Tyrosine metabolism	3,4-dihydroxyphenylacetate 2,3-dioxygenase	1.13.11.15	KOX_10045	34dhpheac + o2 <-> 5cm2hmsa
5CM2HMSAD	Tyrosine metabolism	5-carboxymethyl-2-hydroxymuconic-semialdehyde dehydrogenase	1.2.1.60	KOX_10050	5cm2hmsa + nad <-> 5cm2hm + nadh
CARHM1	Tyrosine metabolism	5-carboxymethyl-2-hydroxymuconate isomerase	5.3.3.10		5cm2hm -> 5c2o3e
5C2O3EI	Tyrosine metabolism	5-oxopent-3-ene-1,2,5-tricarboxylate decarboxylas isomerase	4.1.1.68	KOX_10055/KOX_10060	5c2o3e -> 2hhpdd + co2
2HHPDDI	Tyrosine metabolism	2-hydroxyhepta-2,4-diene-1,7-dioate isomerase	4.1.1.68/5.3.3.-	KOX_10055/KOX_10060	2hhpdd <-> 2o3e
ACDH2	Tyrosine metabolism	acyl dehydratase	4.2.1.-	KOX_10035	2hhpdd -> 24dhhepd
ACDH3	Tyrosine metabolism	acyl dehydratase	4.2.1.-	KOX_10035	2o3e -> 24dhhepd
DHHED1	Tyrosine metabolism	2,4-dihydroxyhept-2-ene-1,7-dioic acid aldolase	4.1.2.-	KOX_10030	24dhhepd -> sucsal + pyr
MALAAC	Tyrosine metabolism	maleylacetoacetate isomerase	5.2.1.2	KOX_25680	4maac -> 4faac
4HPHED1	Tyrosine metabolism	4-hydroxyphenylpyruvate dioxygenase	1.13.11.27	KOX_22735	4hpp + o2 -> homogen + co2
ASPAM5	Tyrosine metabolism	aspartate aminotransferase	2.6.1.1	KOX_16370	akg + tyr <-> 4hpp + glu
MNAO15	Tyrosine metabolism	monoamine oxidase	1.4.3.21	KOX_19410(tynA)	dopa + o2 -> 34dhpac + nh4 + h2o2
ALCDd	Tyrosine metabolism	alcohol dehydrogenase	1.1.1.1	KOX_19595/KOX_20090/KOX_23025	34dhma + nadh <-> 34dhpeg + nad
GENDO	Tyrosine metabolism	gentisate 1,2-dioxygenase	1.13.11.4	KOX_25690	gensa + o2 -> malpyr
MALPYRI	Tyrosine metabolism	maleylpyruvate isomerase	5.2.1.4	KOX_25680	malpyr -> fumpyr
ACPYRH	Tyrosine metabolism	acylpyruvate hydrolase	3.7.1.5	KOX_25685	fumpyr -> fum + pyr
NADHDH2	Ubiquinone	NADH dehydrogenase	1.6.5.3	KOX_26335/KOX_26340/KOX_26345/KOX_26350/KOX_26355/KOX_26360/KOX_26365/KOX_26370/KOX_26375/KOX_26380/KOX_26385/KOX_26390/KOX_26395	uq + nadh -> uqh2 + nad
NAPTS	Ubiquinone Biosynthesis	naphthoate synthase	4.1.3.36	KOX_26270	sbzcoa -> d2naptcoa
CHMPL	Ubiquinone Biosynthesis	Chorismate pyruvate lyase	4.1.3.40	KOX_08240	chor <-> 4hb + pyr
DMUQMT	Ubiquinone Biosynthesis	3-Dimethylubiquinonol 3-methyltransferase	2.1.1.64	KOX_26130	2omhmbl + sam -> sah + uq
HBZOCPT	Ubiquinone Biosynthesis	Hydroxybenzoate octaprenyltransferase	2.5.1.-	KOX_08245(ubiA)	4hb + opp -> 3op4hb + ppi
OCPHPM	Ubiquinone Biosynthesis	2-octaprenyl-6-hydroxyphenol methylase	2.1.1.222	KOX_26130	2op6hp + sam -> 2opmp + sah
OCTMBZM	Ubiquinone Biosynthesis	2-Octaprenyl-6-methoxy-benzoquinol methylase	2.1.1.163	KOX_07775(ubiE)	2ombzl + sam -> 2ommbl + sah
OMMBZH	Ubiquinone Biosynthesis	2-octaprenyl-3-methyl-6-methoxy-1,4-benzoquinol hydroxylase	1.14.13.-	KOX_14330(ubiF)	2ommbl + o2 + nadph -> 2omhmbl + nadp
OCMPH1	Ubiquinone Biosynthesis	2-octaprenyl-6-methoxyphenol hydroxylase	1.14.13.-	KOX_02500	2opmp + o2 + nadph -> 2ombzl + nadp
OCMPH2	Ubiquinone Biosynthesis	2-octaprenyl-6-methoxyphenol hydroxylase	1.14.13.-	KOX_02500	2 2opmp + o2 -> 2 2ombzl
OCMPH3	Ubiquinone Biosynthesis	2-octaprenyl-6-methoxyphenol hydroxylase (anaerobic)	1.14.13.-	KOX_02500	2opmp + 2 atp + nad -> 2ombzl + 2 adp + nadh + 2 pi
OCHBZDC	Ubiquinone Biosynthesis	Octaprenyl-hydroxybenzoate decarboxylase	4.1.1.-	KOX_01005/KOX_02350/KOX_02355/KOX_07815/KOX_26545	3op4hb -> 2opp + co2
OCPPH2	Ubiquinone Biosynthesis	2-Octaprenylphenol hydroxylase		KOX_07785(ubiB)	2opp + o2 + nadph -> 2op6hp + nadp
OCPPH1	Ubiquinone Biosynthesis	2-Octaprenylphenol hydroxylase		KOX_07785(ubiB)	2 2opp + o2 -> 2 2op6hp
OCPPH3	Ubiquinone Biosynthesis	2-Octaprenylphenol hydroxylase (anaerobic)		KOX_07785(ubiB)	2opp + 2 atp + nad -> 2op6hp + 2 adp + nadh + 2 pi
UMBM1	Ubiquinone Biosynthesis	ubiquinone/menaquinone biosynthesis methyltransferase	2.1.1.163	KOX_07775(ubiE)	2dmmq8 + sam -> mk + sah
UMBM2	Ubiquinone Biosynthesis	ubiquinone/menaquinone biosynthesis methyltransferase	2.1.1.163	KOX_07775(ubiE)	pnpq + sam -> pq + sah
SPMS3	Ubiquinone Biosynthesis	spermidine synthase	2.5.1.16	KOX_11110	sama + sprmd -> 5mta + sprm
ALHD12	Ubiquinone Biosynthesis	aldehyde dehydrogenase (NAD+)	1.2.1.3	KOX_00375	bapa + nad -> bala + nadh
OMMBZHx	Ubiquinone Biosynthesis	2-octaprenyl-6-methoxyphenol hydroxylase	1.14.13.-	KOX_14330(ubiF)	2ommbl + 2 atp + nad -> 2omhmbl + 2 adp + nadh + 2 pi
ISOCHORS	Ubiquinone Biosynthesis	isochorismate synthase	5.4.4.2	KOX_13950/KOX_26285	chor <-> isochor
SUCEPHCS	Ubiquinone Biosynthesis	2-succinyl-5-enolpyruvyl-6-hydroxy-3-cyclohexene-1-carboxylate synthase	2.2.1.9	KOX_26280	isochor + akg -> sucephc + co2
SHCHCS	Ubiquinone Biosynthesis	2-succinyl-6-hydroxy-2,4-cyclohexadiene-1-carboxylate synthase	4.2.99.20	KOX_26275	sucephc -> shchc + pyr
OSUCCBS	Ubiquinone Biosynthesis	O-succinylbenzoate synthase	4.2.1.113	KOX_26265	shchc -> osuccbenz
OSUCCBCL	Ubiquinone Biosynthesis	O-succinylbenzoic acid--CoA ligase	6.2.1.26	KOX_26260	atp + osuccbenz + coa -> amp + ppi + sbzcoa
D2NAPCH	Ubiquinone Biosynthesis	1,4-dihydroxy-2-naphthoyl-CoA hydrolase	3.1.2.28		d2naptcoa -> dhn + coa
DHNOPT1	Ubiquinone Biosynthesis	1,4-dihydroxy-2-naphthoate octaprenyltransferase	2.5.1.74	KOX_07270	dhn + opp -> 2dmmq8 + ppi + co2
DHNOPT2	Ubiquinone Biosynthesis	1,4-dihydroxy-2-naphthoate octaprenyltransferase	2.5.1.-	KOX_07270	dhn + phppi -> pnpq + co2 + ppi
CAT	Unassigned	catalase	1.11.1.6	KOX_18225(katE)/KOX_26215	2 h2o2 -> 2 h2o + o2
MISRXN	unclear reaction	unclear reaction			g3p + pyr -> 4mhetz
ACGPR	Urea Cycle and Metabolism of amino groups	N-acetyl-g-glutamyl-phosphate reductase	1.2.1.38	KOX_07370(argC)	naglus + nadp + pi <-> acg5p + nadph
GLU5SD	Urea Cycle and Metabolism of amino groups	glutamate-5-semialdehyde dehydrogenase	1.2.1.41	KOX_12055(proA)	glu5p + nadph -> glugsal + nadp + pi
AGLUS	Urea Cycle and Metabolism of amino groups	N-acetylglutamate synthase	2.3.1.1	KOX_01560	accoa + glu -> naglu + coa
SPMS1	Urea Cycle and Metabolism of amino groups	spermidine synthase	2.5.1.16	KOX_11110	sama + ptrc -> 5mta + sprmd
AORNT	Urea Cycle and Metabolism of amino groups	acetylornithine transaminase	2.6.1.11	KOX_04525(argD)	naorn + akg <-> naglus + glu
GLU5K	Urea Cycle and Metabolism of amino groups	glutamate 5-kinase	2.7.2.11	KOX_12060	atp + glu -> adp + glu5p
AGLUK	Urea Cycle and Metabolism of amino groups	acetylglutamate kinase	2.7.2.8	KOX_07375	naglu + atp -> acg5p + adp
ACORND	Urea Cycle and Metabolism of amino groups	acetylornithine deacetylase	3.5.1.16	KOX_07365/KOX_16395/KOX_19240	naorn -> ac + orn
AGMT	Urea Cycle and Metabolism of amino groups	agmatinase	3.5.3.11	KOX_00245/KOX_02630	agmatine -> ptrc + urea
ARGDC	Urea Cycle and Metabolism of amino groups	arginine decarboxylase	4.1.1.19	KOX_02635	arg -> agmatine + co2
ALLPH	Urea cycle and metabolism of amino groups	allophanate hydrolase	3.5.1.54	KOX_20290	u1car -> 2 co2 + 2 nh4
ALHD10	Urea cycle and metabolism of amino groups	aldehyde dehydrogenase (NAD+)	1.2.1.3	KOX_00375	n4aab + nad -> 4aabut + nadh
ALHD11	Urea cycle and metabolism of amino groups	aldehyde dehydrogenase (NAD+)	1.2.1.3/1.2.1.19	KOX_00375/KOX_19750	4ab + nad -> gaba + nadh
ALHD11p	Urea cycle and metabolism of amino groups	aldehyde dehydrogenase (NAD+)	1.2.1.3	KOX_00375	4ab + nadp -> gaba + nadph
SPMS2	Urea cycle and metabolism of amino groups	spermidine synthase	2.5.1.16	KOX_11110	sama + ptrc -> 5mta + sprmd
AMDS3	Urea cycle and metabolism of amino groups	amidase	3.5.1.4	KOX_09850/KOX_13720/KOX_20510	guadbut -> guadbutn + nh4
3IPPMD1	Valine, Leucine and Isoleucine Biosynthesis	3-isopropylmalate/(R)-2-methylmalate dehydratase	4.2.1.35	KOX_10840(leuD)/KOX_10845	r2mm -> 2mm

3IPPMD2	Valine, Leucine and Isoleucine Biosynthesis	3-isopropylmalate/(R)-2-methylmalate dehydratase	4.2.1.33	KOX_10840(leuD)/KOX_10845	2mm -> e3mm
IPPMD	Valine, Leucine and Isoleucine Biosynthesis	3-isopropylmalate dehydrogenase	1.1.1.85	KOX_10850	e3mm + nad -> obut + co2 + nadh
ACCOAA1	Valine, Leucine and Isoleucine degradation	acetyl-CoA acyltransferase	2.3.1.16	KOX_26660(fadI)	coa + 2maaccoa -> ppcoa + accoa
ALHD6	Valine, Leucine and Isoleucine degradation	aldehyde dehydrogenase (NAD+)	1.2.1.3	KOX_00375	mmsa + nad -> mm + nadh
MMCS	Valine, Leucine and Isoleucine degradation	unclear reaction	unclear reaction		mm + coa -> mmcoa-R
MMCM	Valine, Leucine, and Isoleucine Degradation	Methylmalonyl-CoA mutase	5.4.99.2	KOX_02540	succoa -> mmcoa-R
IPMALD	Valine, Leucine, and Isoleucine Metabolism	3-isopropylmalate dehydrogenase	1.1.1.85	KOX_10850	3c2hmp + nad -> oicap + nadh
KARIS1	Valine, Leucine, and Isoleucine Metabolism	ketol-acid reductoisomerase	1.1.1.86	KOX_07475	dhmva + nadp <-> alac-S + nadph
KARIS2	Valine, Leucine, and Isoleucine Metabolism	ketol-acid reductoisomerase	1.1.1.86	KOX_07475	abut + nadph <-> dhmp + nadp
ILETA	Valine, Leucine, and Isoleucine Metabolism	isoleucine transaminase	2.6.1.42	KOX_07455/KOX_1652	akg + ile <-> 3mop + glu
LEUTA	Valine, Leucine, and Isoleucine Metabolism	leucine transaminase	2.6.1.42	KOX_07455/KOX_1652	4mop + glu <-> akg + leu
VALTA	Valine, Leucine, and Isoleucine Metabolism	valine transaminase	2.6.1.42	KOX_07455/KOX_1652	akg + val <-> 3mob + glu
IPPMS	Valine, Leucine, and Isoleucine Metabolism	2-isopropylmalate synthase	2.3.3.13	KOX_00490/KOX_10855	3mob + accoa -> 3c3hmp + coa
ACLACS	Valine, Leucine, and Isoleucine Metabolism	acetolactate synthase	2.2.1.6	KOX_06425/KOX_06430/KOX_07445/KOX_07450(ilvM)/KOX_10865/KOX_10870(ilvH)/KOX_22370	2 pyr -> alac-S + co2
IPMALD1	Valine, Leucine, and Isoleucine Metabolism	3-isopropylmalate dehydratase	4.2.1.33	KOX_10840(leuD)/KOX_10845	3c2hmp <-> 2ippm
IPMALD2	Valine, Leucine, and Isoleucine Metabolism	3-isopropylmalate dehydratase	4.2.1.33	KOX_10840(leuD)/KOX_10845	2ippm <-> 3c3hmp
DHADT1	Valine, Leucine, and Isoleucine Metabolism	dihydroxy-acid dehydratase	4.2.1.9	KOX_07460	dhmva -> 3mob
ACHBUTS	Valine, Leucine, and Isoleucine Metabolism	2-aceto-2-hydroxybutanoate synthase	2.2.1.6	KOX_06425/KOX_06430/KOX_07445/KOX_07450(ilvM)/KOX_10865/KOX_10870(ilvH)/KOX_22370	obut + pyr -> abut + co2
DHADT2	Valine, Leucine, and Isoleucine Metabolism	ihydroxy-acid dehydratase	4.2.1.9	KOX_07460	dhmp -> 3mop
OMCDC	Valine, Leucine, and Isoleucine Metabolism	2-Oxo-4-methyl-3-carboxypentanoate decarboxylation	spontaneous		oicap -> 4mop + co2
VALPYRAT	Valine, Leucine, and Isoleucine Metabolism	valine--pyruvate aminotransferase	2.6.1.66	KOX_05660(avtA)/KOX_26405	3mob + ala <-> val + pyr
PDX5PO	Vitamine B6 metabolism	pyridoxine 5'-phosphate oxidase	1.4.3.5	KOX_22030	o2 + pdx5p -> h2o2 + pydx5p
PYAM5PO	Vitamine B6 metabolism	pyridoxamine 5'-phosphate oxidase	1.4.3.5	KOX_22030	o2 + pyam5p -> h2o2 + nh4 + pydx5p
OHPBAKGT	Vitamine B6 metabolism	O-Phospho-4-hydroxy-L-threonine:2-oxoglutarate aminotransferase	2.6.1.52	KOX_15995	glu + ohpb <-> akg + pht
HTHRS	Vitamine B6 metabolism	4-Hydroxy-L-threonine synthase	4.2.3.1	KOX_10420	pht -> 4hlt + pi
DALATA	Vitamine B6 metabolism	D-alanine transaminase	2.6.1.54		dala + pydx5p -> pyam5p + pyr
E4PDH	Vitamine B6 metabolism	Erythrose 4-phosphate dehydrogenase	1.2.1.72	KOX_02590	e4p + nad <-> er4p + nadh
P5PPR	Vitamine B6 metabolism	pyridoxal-5'-phosphate phosphohydrolase	3.1.3.74		pyam5p -> pi + pdla
PX5PS	Vitamine B6 metabolism	Pyridoxine 5'-phosphate synthase	1.1.1.262/2.6.99.2	KOX_10700(pdxA)/KOX_11095(pdxA)/KOX_17445(pdxA)/KOX_27535	dx5p + nad + pht -> co2 + nadh + pdx5p + pi
ER4PD	Vitamine B6 metabolism	Erythronate 4-phosphate (4per) dehydrogenase	1.1.1.290	KOX_26590	er4p + nad <-> nadh + ohpb
PYR5OXM	Vitamine B6 metabolism	pyridoxamine 5'-phosphate oxidase	1.4.3.5	KOX_22030	pdla + o2 <-> pl + nh4 + h2o2
PYR5OXX	Vitamine B6 metabolism	pyridoxamine 5'-phosphate oxidase	1.4.3.5	KOX_22030	pydxn + o2 <-> pl + h2o2
HTHRPD	Vitamine B6 metabolism	4-hydroxythreonine-4-phosphate dehydrogenase	1.1.1.262	KOX_10700(pdxA)/KOX_11095(pdxA)/KOX_17445(pdxA)	pht + nad -> ao4pob + nadh
SPOTN	Vitamine B6 metabolism	spontaneous			ao4pob -> 3a2op + co2
PDLAK	Vitamine B6 metabolism	Pyridoxamine kinase	2.7.1.35	KOX_22020/KOX_26865(pdxK)	pdla + atp -> pyam5p + adp
PLK	Vitamine B6 metabolism	Pyrodoxal kinase	2.7.1.35	KOX_22020/KOX_26865(pdxK)	pl + atp -> pydx5p + adp
PYDXNK	Vitamine B6 metabolism	Pyridoxine kinase	2.7.1.35	KOX_22020/KOX_26865(pdxK)	pydxn + atp -> pdx5p + adp
13PDOt	Transport, Extracellular	Propane-1,3-diol facilitated transport			13pdo_e <-> 13pdo
23BDOt	Transport, Extracellular	(R,R)-Butane-2,3-diol facilitated transport			23bdo_e <-> 23bdo
12PDOt	Transport, Extracellular	S-Propane-1,2-diol facilitated transport			12ppd-S_e <-> 12ppd-S
NMNt7	Transport, Extracellular	NMN transport via NMN glycohydrolase			namn_e -> nam + r5p
ACALDt	Transport, Extracellular	acetaldehyde reversible transport			acal_e <-> acal
GUAt1	Transport, Extracellular	Guanine transport			gn_e <-> gn
HYXNt	Transport, Extracellular	Hypoxanthine transport			hyxn_e <-> hyxn
XANt1	Transport, Extracellular	xanthine reversible transport			xan_e <-> xan
NACUP	Transport, Extracellular	Nicotinic acid uptake			nac_e -> nac
ASNabc	Transport, Extracellular	L-asparagine transport via ABC system			asn_e + atp -> adp + asn + pi
ASNtr	Transport, Extracellular	L-asparagine reversible transport via proton symport			asn_e + hext <-> asn
DAPabc	Transport, Extracellular	M-diaminopimelic acid ABC transport			26dap-M_e + atp -> 26dap-M + adp + pi
CYSabc	Transport, Extracellular	L-cysteine transport via ABC system			atp + cys_e -> adp + cys + pi
ACtr	Transport, Extracellular	acetate reversible transport via proton symport			ac_e + hext <-> ac
ETOHtr	Transport, Extracellular	ethanol reversible transport via proton symport			eth -> eth_e + hext
PYRtr	Transport, Extracellular	pyruvate reversible transport via proton symport			hext + pyr_e <-> pyr
O2t	Transport, Extracellular	o2 transport (diffusion)			o2_e <-> o2
CO2t	Transport, Extracellular	CO2 transporter via diffusion			co2_e <-> co2
DHAt	Transport, Extracellular	Dihydroxyacetone transport via facilitated diffusion			glyn_e <-> glyn
NH3t	Transport, Extracellular	ammonia reversible transport			nh4_e <-> nh4
ARBtr	Transport, Extracellular	L-arabinose transport via proton symport			larabinose_e + hext -> larabinose
ARBabc	Transport, Extracellular	L-arabinose transport via ABC system			larabinose_e + atp -> adp + larabinose + pi
HIStr	Transport, Extracellular	L-histidine reversible transport via proton symport			hext + his_e <-> his
PHEtr	Transport, Extracellular	L-phenylalanine reversible transport via proton symport			hext + phe_e <-> phe
LEUtr	Transport, Extracellular	L-leucine reversible transport via proton symport			hext + leu_e <-> leu
VALtr	Transport, Extracellular	L-valine reversible transport via proton symport			hext + val_e <-> val
ILEtr	Transport, Extracellular	L-isoleucine reversible transport via proton symport			hext + ile_e <-> ile
CBL1abc	Transport, Extracellular	Cob(1)alamin transport via ABC system			atp + cbl1_e -> adp + cbl1 + pi
CADVt	Transport, Extracellular	Lysine/Cadaverine antiporter			15dap + hext + lys_e -> 15dap_e + lys
CRNt7	Transport, Extracellular	Carnitine/butyrobetaine antiporter			crn_e + gbbtn -> crn + gbbtn_e
NAt_1	Transport, Extracellular	sodium proton antiporter (H:NA is 1:1)			hext + na <-> na_e
CITtsuc	Transport, Extracellular	Citrate transport via succinate antiport			cit_e + succ -> cit + succ_e
CSNt2	Transport, Extracellular	cytosine transport in via proton symport			ct_e + hext -> ct
ACGApts	Transport, Extracellular	N-Acetyl-D-glucosamine transport via PEP:Pyr PTS	2.7.1.69	KOX_14365	naga_e + pep -> naga6p + pyr
DALAtr	Transport, Extracellular	D-alanine transport via proton symport			dala_e + hext <-> dala
DSERtr	Transport, Extracellular	D-serine transport via proton symport			hext + dser_e <-> dser
GLYtr	Transport, Extracellular	glycine reversible transport via proton symport			gly_e + hext <-> gly
SULabc	Transport, Extracellular	sulfate transport via ABC system			atp + so4_e -> adp + pi + so4

ASPt_2	Transport, Extracellular	Aspartate transport via proton symport (2 H)			asp_e + 2 hext -> asp
FUMt_2	Transport, Extracellular	Fumarate transport via proton symport (2 H)			fum_e + 2 hext -> fum
MALt_2	Transport, Extracellular	Malate transport via proton symport (2 H)			2 hext + mal_e -> mal
SUCCt_2	Transport, Extracellular	succinate transport via proton symport (2 H)			2 hext + succ_e -> succ
ASPt_3	Transport, Extracellular	L-asparate transport via proton symport (3 H)			asp_e + 3 hext -> asp
MALt_3	Transport, Extracellular	Malate transport via proton symport (3 H)			3 hext + mal_e -> mal
SUCCt_3	Transport, Extracellular	Succintate transport via proton symport (3 H)			3 hext + succ_e -> succ
SUCCet	Transport, Extracellular	Succinate efflux via proton symport			succ -> hext + succ_e
FUMt_3	Transport, Extracellular	Fumarate transport via proton symport (3 H)			fum_e + 3 hext -> fum
SUCFUMt	Transport, Extracellular	succinate:fumarate antiporter			fum_e + succ <-> fum + succ_e
GALCTNtr	Transport, Extracellular	D-galactonate transport via proton symport, reversible			dgalctn_e + hext <-> dgalctn
GALACtr	Transport, Extracellular	D-galacturonate transport via proton symport, reversible			dgalac_e + hext <-> dgalac
GLCURtr	Transport, Extracellular	D-glucuronate transport via proton symport, reversible			dgluc_e + hext <-> dgluc
OCDCAt	Transport, Extracellular	Octadecanoate transport via proton symport			hext + c180_e -> c180
HDCAt	Transport, Extracellular	Hexadecanoate transport via proton symport			hext + c160_e -> c160
TTDCAt	Transport, Extracellular	Tetradecanoate transport via proton symport			hext + c140_e -> c140
FE2abc	Transport, Extracellular	iron (II) transport via ABC system			atp + fe2_e -> adp + fe2 + pi
FORt	Transport, Extracellular	formate transport via diffusion			formate_e <-> formate
FUCt	Transport, Extracellular	L-fucose transport via proton symport			fuc_e + hext <-> fuc
ABUTt	Transport, Extracellular	4-aminobutyrate transport in via proton symport			gaba_e + hext -> gaba
GLACt	Transport, Extracellular	D-galactose transport in via proton symport			glac_e + hext -> glac
GLCt	Transport, Extracellular	D-glucose transport in via proton symport			glc_e + hext -> glc
GALTpts	Transport, Extracellular	Galactitol transport via PEP:Pyr PTS	2.7.1.69	KOX_03520/KOX_03525/KOX_03530/KOX_21000	galt_e + pep -> galt1p + pyr
MALTpts	Transport, Extracellular	Maltose transport via PEP:Pyr PTS	2.7.1.69	KOX_21915/KOX_26855	mlt_e + pep -> mlt6p + pyr
TREpts	Transport, Extracellular	Trehalose transport via PEP:Pyr PTS	2.7.1.69	KOX_09260/KOX_26855	tre_e + pep -> tre6p + pyr
SUCpts	Transport, Extracellular	Sucrose transport via PEP:Pyr PTS	2.7.1.69	KOX_13365	suc_e + pep -> suc6p + pyr
GLCpts	Transport, Extracellular	Glucose transport via PEP:Pyr PTS	2.7.1.69	KOX_26855	glc_e + pep -> g6p + pyr
NAMURpts	Transport, Extracellular	N-Acetylmuramate transport via PEP:Pyr PTS	2.7.1.69	KOX_06290(murP)/KOX_26855	namur_e + pep -> namur6p + pyr
GLNabc	Transport, Extracellular	L-glutamine transport via ABC system			atp + gln_e -> adp + gln + pi
GLYCt	Transport, Extracellular	glycerol transport via channel			gl <-> gl_e
GLYALDt	Transport, Extracellular	Glyceraldehyde facilitated diffusion			t3_e <-> t3
UREAt	Transport, Extracellular	Urea transport via facilitate diffusion			urea_e <-> urea
GLYC3Pt	Transport, Extracellular	Glycerol-3-phosphate : phosphate antiporter			glyc3p_e + pi -> glyc3p + pi_e
ASPabc	Transport, Extracellular	L-aspartate transport via ABC system			asp_e + atp -> adp + asp + pi
GLUabc	Transport, Extracellular	L-glutamate transport via ABC system			atp + glu_e -> adp + glu + pi
ASPt	Transport, Extracellular	L-aspartate transport in via proton symport			asp_e + hext -> asp
GLUtr	Transport, Extracellular	L-glutamate transport via proton symport, reversible			glu_e + hext <-> glu
GLUt	Transport, Extracellular	Na+/glutamate symport			glu_e + na_e -> glu + na
ORNabc	Transport, Extracellular	ornithine transport via ABC system			atp + orn_e -> adp + orn + pi
ARGabc	Transport, Extracellular	L-arginine transport via ABC system			arg_e + atp -> adp + arg + pi
HISabc	Transport, Extracellular	L-histidine transport via ABC system			atp + his_e -> adp + his + pi
LYSabc	Transport, Extracellular	L-lysine transport via ABC system			atp + lys_e -> adp + lys + pi
IDONtr	Transport, Extracellular	L-idonate transport via proton symport, reversible			hext + idon_e <-> idon
GLCNtr	Transport, Extracellular	D-gluconate transport via proton symport, reversible			gluc_e + hext <-> gluc
DDGLCNtr	Transport, Extracellular	2-dehydro-3-deoxy-D-gluconate transport via proton symport, reversible			kdg_e + hext <-> kdg
Kabc	Transport, Extracellular	Potassium ABC transporter			atp + k_e -> adp + k + pi
AKGt	Transport, Extracellular	2-oxoglutarate reversible transport via symport			akg_e + hext <-> akg
LCTSt	Transport, Extracellular	Lactose transport via proton symport			hext + lactose_e <-> lactose
ILEabc	Transport, Extracellular	L-isoleucine transport via ABC system			atp + ile_e -> adp + ile + pi
THRabc	Transport, Extracellular	L-threonine transport via ABC system			atp + thr_e -> adp + pi + thr
ALAabc	Transport, Extracellular	L-alanine transport via ABC system			ala_e + atp -> adp + ala + pi
VALabc	Transport, Extracellular	L-valine transport via ABC system			atp + val_e -> adp + pi + val
LEUabc	Transport, Extracellular	L-leucine transport via ABC system			atp + leu_e -> adp + leu + pi
DLACt	Transport, Extracellular	D-lactate transport via proton symport			hext + lac_e <-> lac
GLYCLTtr	Transport, Extracellular	glycolate transport via proton symport, reversible			glycolate_e + hext <-> glycolate
LLACtr	Transport, Extracellular	L-lactate reversible transport via proton symport			hext + llac_e <-> llac
LYStr	Transport, Extracellular	L-lysine reversible transport via proton symport			hext + lys_e <-> lys
MALTPTabc	Transport, Extracellular	maltopentaose transport via ABC system			atp + maltpt_e -> adp + maltpt + pi
MLTabc	Transport, Extracellular	maltose transport via ABC system			atp + mlt_e -> adp + mlt + pi
MALTTTRabc	Transport, Extracellular	maltotetraose transport via ABC system			atp + mltttr_e -> adp + mltttr + pi
MALTHXabc	Transport, Extracellular	maltohexaose transport via ABC system			atp + mlthx_e -> adp + mlthx + pi
MALTTRabc	Transport, Extracellular	Maltotriose transport via ABC system			atp + mlttr_e -> adp + mlttr + pi
FRUpts2	Transport, Extracellular	Fructose transport via PEP:Pyr PTS (f6p generating)			fru_e + pep -> f6p + pyr
MANpts	Transport, Extracellular	D-mannose transport via PEP:Pyr PTS	2.7.1.69	KOX_02420/KOX_02425/KOX_02430/KOX_02435/KOX_10140/KOX_10145/KOX_10150/KOX_10155/KOX_13540/KOX_13545/KOX_16345/KOX_16350/KOX_16355/KOX_16360/KOX_23650/KOX_23655/KOX_23660	man_e + pep -> man6p + pyr
GAMpts	Transport, Extracellular	D-glucosamine transport via PEP:Pyr PTS			gam_e + pep -> ga6p + pyr
MELIBt	Transport, Extracellular	melibiose transport in via symport			hext + meli_e -> meli
METabc	Transport, Extracellular	L-methionine transport via ABC system			atp + met_e -> adp + met + pi
METDabc	Transport, Extracellular	D-methionine transport via ABC system			atp + dmet_e -> adp + dmet + pi
GLACabc	Transport, Extracellular	D-galactose transport via ABC system			atp + glac_e -> adp + glac + pi
INDOLEtr	Transport, Extracellular	Indole transport via proton symport, reversible			hext + indole_e <-> indole

ACNAMt	Transport, Extracellular	N-acetylneuraminate proton symport			naneu_e + hext -> naneu
NO3t	Transport, Extracellular	nitrate transport in via nitrite antiport			no2 + no3_e -> no2_e + no3
NO2tr	Transport, Extracellular	nitrite transport in via proton symport, reversible			hext + no2_e <-> no2
NAt_2	Transport, Extracellular	sodium proton antiporter (H:NA is 2)			2 hext + na -> na_e
NAt_1.5	Transport, Extracellular	sodium proton antiporter (H:NA is 1.5)			3 hext + 2 na -> 2 na_e
GSNt	Transport, Extracellular	guanosine transport in via proton symport			gsn_e + hext -> gsn
DGSNt	Transport, Extracellular	deoxyguanosine transport in via proton symport			dg_e + hext -> dg
INSt	Transport, Extracellular	inosine transport in via proton symport			hext + ins_e -> ins
DINSt	Transport, Extracellular	deoxyinosine transport in via proton symport			din_e + hext -> din
ADNt	Transport, Extracellular	adenosine transport in via proton symport			adn_e + hext -> adn
URIt	Transport, Extracellular	uridine transport in via proton symport			hext + uri_e -> uri
CYTDt	Transport, Extracellular	cytidine transport in via proton symport			cytd_e + hext -> cytd
DCYTt	Transport, Extracellular	deoxycytidine transport in via proton symport			dc_e + hext -> dc
DURIt	Transport, Extracellular	deoxyuridine transport in via proton symport			du_e + hext -> du
DADNt	Transport, Extracellular	deoxyadenosine transport in via proton symport			da_e + hext -> da
THMDt	Transport, Extracellular	thymidine transport in via proton symport			hext + thymd_e -> thymd
PNTOt	Transport, Extracellular	Pantothenate sodium symporter			na_e + pnto_e -> na + pnto
PItr	Transport, Extracellular	phosphate reversible transport via symport			hext + pi_e <-> pi
NMNP	Transport, Extracellular	NMN permease			namn_e -> namn
PTRCabc	Transport, Extracellular	putrescine transport via ABC system			atp + ptrc_e -> adp + pi + ptrc
SPMDabc	Transport, Extracellular	spermidine transport via ABC system			atp + sprmd_e -> adp + pi + sprmd
PTRCORNt	Transport, Extracellular	putrescine/ornithine antiporter			orn + ptrc_e <-> orn_e + ptrc
PTRCtr	Transport, Extracellular	putrescine transport in via proton symport, reversible			hext + ptrc_e <-> ptrc
PROtr	Transport, Extracellular	L-proline reversible transport via proton symport			hext + pro_e <-> pro
PROabc	Transport, Extracellular	L-proline transport via ABC system			atp + pro_e -> adp + pi + pro
PIabc	Transport, Extracellular	phosphate transport via ABC system			atp + pi_e -> adp + 2 pi
ACMANApts	Transport, Extracellular	N-acetyl-D-mannosamine transport via PTS			nadma_e + pep -> nadma6p + pyr
MNLpts	Transport, Extracellular	mannitol transport via PEP:Pyr PTS	2.7.1.69	KOX_05760	mnt_e + pep -> mnt1p + pyr
FRUpts	Transport, Extracellular	D-fructose transport via PEP:Pyr PTS	2.7.1.69	KOX_03505/KOX_06130/KOX_25810/KOX_25820	fru_e + pep -> f1p + pyr
FRUabc	Transport, Extracellular	D-fructose transport via ABC system			atp + fru_e -> adp + pi + fru
PROt	Transport, Extracellular	Na+/Proline-L symporter			na_e + pro_e -> na + pro
RMNt	Transport, Extracellular	L-rhamnose transport via proton symport			hext + rmn_e -> rmn
TSULabc	Transport, Extracellular	thiosulfate transport via ABC system			atp + tsul_e -> adp + pi + tsul
SERtr	Transport, Extracellular	L-serine reversible transport via proton symport			hext + ser_e <-> ser
THMabc	Transport, Extracellular	thiamine transport via ABC system			atp + thiamin_e -> adp + pi + thiamin
SBTpts	Transport, Extracellular	D-sorbitol transport via PEP:Pyr PTS	2.7.1.69	KOX_00575/KOX_00580/KOX_00585/KOX_17460	pep + sot_e -> pyr + sbt6p
SORBpts	Transport, Extracellular	L-sorbose transport via PEP:Pyr PTS	2.7.1.69	KOX_08100/KOX_08105/KOX_08110	pep + sorb_e -> pyr + sb1p
SERt	Transport, Extracellular	L-serine via sodium symport			na_e + ser_e -> na + ser
THRt	Transport, Extracellular	L-threonine via sodium symport			na_e + thr_e -> na + thr
TAURabc	Transport, Extracellular	taurine transport via ABC system			atp + taur_e -> adp + pi + taur
THRtr	Transport, Extracellular	L-threonine reversible transport via proton symport			hext + thr_e <-> thr
TRPtr	Transport, Extracellular	L-tryptophan reversible transport via proton symport			hext + trp_e <-> trp
Ktr	Transport, Extracellular	potassium reversible transport via proton symport			hext + k_e <-> k
TYRtr	Transport, Extracellular	L-tyrosine reversible transport via proton symport			hext + tyr_e <-> tyr
GLYC3Pabc	Transport, Extracellular	sn-Glycerol 3-phosphate transport via ABC system			atp + glyc3p_e -> adp + glyc3p + pi
MAN6Pt_2	Transport, Extracellular	Mannose-6-phosphate transport via phosphate antiport			man6p_e + 2 pi -> man6p + 2 pi_e
G6Pt_2	Transport, Extracellular	Glucose-6-phosphate transport via phosphate antiport			g6p_e + 2 pi -> g6p + 2 pi_e
FUCPt_2	Transport, Extracellular	Fucose 1-phosphate transport via phosphate antiport			fuc1p_e + 2 pi -> fuc1p + 2 pi_e
URAt	Transport, Extracellular	uracil transport in via proton symport			hext + ura_e -> ura
XTSNtr	Transport, Extracellular	Xanthosine transport via proton symport			hext + xtsine_e <-> xtsine
INStr	Transport, Extracellular	inosine transport in via proton symport, reversible			hext + ins_e <-> ins
ADNtr	Transport, Extracellular	adenosine transport in via proton symport, reversible			adn_e + hext <-> adn
CYTDtr	Transport, Extracellular	cytidine transport in via proton symport, reversible			cytd_e + hext <-> cytd
THMDtr	Transport, Extracellular	thymidine transport in via proton symport, reversible			hext + thymd_e <-> thymd
URItr	Transport, Extracellular	uridine transport in via proton symport, reversible			hext + uri_e <-> uri
XYLt	Transport, Extracellular	D-xylose transport in via proton symport			hext + xyl_e -> xyl
XYLabc	Transport, Extracellular	D-xylose transport via ABC system			atp + xyl_e -> adp + xyl + pi
CHLtr	Transport, Extracellular	choline transport via proton symport, reversible			choline_e + hext <-> choline
ADEtr	Transport, Extracellular	adenine transport via proton symport (reversible)			ad_e + hext <-> ad
RIBabc	Transport, Extracellular	D-ribose transport via ABC system			atp + rib_e -> adp + pi + rib
CRNabc	Transport, Extracellular				atp + crn_e <-> adp + crn + pi
MOBDabc	Transport, Extracellular				atp + mobd_e <-> adp + pi + mobd
ASO3t1	Transport, Extracellular				aso3 <-> aso3_e
MG2t	Transport, Extracellular				mg2_e <-> mg2
COBTt	Transport, Extracellular				cobalt2 <-> cobalt2_e
ASPALAt	Transport, Extracellular				asp_e + ala <-> asp + ala_e
ASO3t2	Transport, Extracellular				aso3 -> aso3_e
NA1t	Transport, Extracellular				na_e <-> na + hext
NA1t2	Transport, Extracellular				na + hext <-> na_e
SUCCt	Transport, Extracellular				succ_e + hext -> succ
SUCCtr	Transport, Extracellular				na_e + succ_e -> na + succ
SO4t	Transport, Extracellular				so4_e + na_e -> so4 + na
LYSt	Transport, Extracellular				lys + hext -> lys_e
CITt	Transport, Extracellular				cit_e + hext <-> cit
4HBZt	Transport, Extracellular				4hb_e + hext <-> 4hb
PPAt	Transport, Extracellular				ppa_e + hext -> ppa
C181t	Transport, Extracellular				c181_e + hext -> c181
GENt	Transport, Extracellular				gensa_e + hext -> gensa
2PGt	Transport, Extracellular				2pg_e + hext -> 2pg
3PGt	Transport, Extracellular				3pg_e + hext -> 3pg
CATECHt	Transport, Extracellular				catech_e + hext -> catech
CLt	Transport, Extracellular				cl_e <-> cl
GLXt	Transport, Extracellular				glx_e + hext -> glx

ICITt	Transport, Extracellular				icit_e + hext <-> icit
ADIPt	Transport, Extracellular				adip_e + hext <-> adip
PACt	Transport, Extracellular				pac_e + hext <-> pac
CCMUCt	Transport, Extracellular				ccmuc_e + hext <-> ccmuc
MCLACTt	Transport, Extracellular				mclact_e + hext <-> mclact
KNTt	Transport, Extracellular				knt_e + hext <-> knt
ANt	Transport, Extracellular				an_e + hext <-> an
ACONCt	Transport, Extracellular				acon-C_e + hext -> acon-C
UROCANt	Transport, Extracellular				urocan_e + hext <-> urocan
2HBAt	Transport, Extracellular				2hba_e + hext -> 2hba
4HBTt	Transport, Extracellular				4hbt_e + hext -> 4hbt
4HPHEACt	Transport, Extracellular				4hpheac_e + hext -> 4hpheac
4HPHEAO	Transport, Extracellular				4hpheac + o2 + nadh -> homogen + nad
OBUTt	Transport, Extracellular				obut_e + hext -> obut
R3HBNt	Transport, Extracellular				r3hbn_e + hext -> r3hbn
4FLRBZt	Transport, Extracellular				4flrbz_e + hext -> 4flrbz
ACETOINt	Transport, Extracellular				acetoin -> acetoin_e
SPOXDM	Unassigned	superoxide dismutase	1.15.1.1	KOX_07230/KOX_22075/KOX_22125	2 h + o2 -> h2o2
TACONMT	Unassigned	trans-aconitate 2-methyltransferase	2.1.1.144	KOX_21215	acon-T + sam -> e3mcpen + sah
HCO3ER	Unassigned	Bicarbonate (HCO_3) equilibration reaction			co2 <-> hco3
ASCBpts	Transport, Inner Membrane	L-ascorbate transport via PEP:Pyr PTS (periplasm)	2.7.1.69	KOX_06970/KOX_06975/KOX_06990/KOX_08925(ulaA)/KOX_08930/KOX_08935/KOX_11160/KOX_15955/KOX_15960/KOX_20835(ulaA)/KOX_20840/KOX_20845/KOX_26455(ulaA)/KOX_26460/KOX_26465	ascb_e + pep -> ascb6p + pyr
IMLTAP	Transport, Outer Membrane	ATPase (isomaltose)	3.6.1.-		atp + imal_e -> imal + pi + adp
Maintenance					atp -> adp + pi
LPS	Lipopolysaccharide Biosynthesis / Recycling	Lipopolysaccharide biosynthesis			0.14 lipa + 0.42 adphep + 0.28 udpg + 0.28 cdpetn + 0.42 ckdo -> 0.42 adp + 0.28 udp + 0.42 cmp + 0.28 cdp + LPS
Protein					1.133 ala + 0.493 arg + 0.41 asn + 0.41 asp + 0.096 cys + 0.499 gln + 0.499 glu + 1.041 gly + 0.19 his + 0.436 ile + 0.768 leu + 0.448 lys + 0.238 met + 0.289 phe + 0.42 pro + 0.534 ser + 0.583 thr + 0.014 trp + 0.259 tyr + 0.666 val + 40 atp -> 40 adp + PROTEIN
DNA			2.7.7.7	KOX_06570/KOX_06860/KOX_09330/KOX_10260/KOX_11520/KOX_11665/KOX_13030/KOX_14255(holA)/KOX_17320/KOX_23820	0.711 datp + 0.907 dctp + 0.711 dttp + 0.907 dgtp + 4.4 atp -> 4.4 adp + 4.4 pi + DNA
RNA			2.7.7.6	KOX_04250/KOX_06005(rpoZ)/KOX_07910(rpoB)/KOX_07915	0.648 atp + 0.737 gtp + 0.98 ctp + 0.762 utp -> 1.25 adp + 1.25 pi + RNA
Phospholipid					1.186 pe + 0.062 pg + 0.027 ps + 0.077 pa + 0.048 clpn -> PHOSPHOLIPID
Cofactors and vitamins (CAV)					0.656 pydxn + 0.145 coa + 0.141 fad + 0.243 fmn + 0.14 uq + 0.167 nad + 0.149 nadp + 0.249 thf + 0.418 thiamin -> CAV
Carbohydrate (CARBO)					4.244 udpnag + 0.849 udgal -> 5.093 udp + CARBO
Biomass					0.521 PROTEIN + 0.023 DNA + 0.131 RNA + 0.035 PEPTIDO + 0.153 CARBO + 0.03 CAV + 0.034 LPS + 0.073 PHOSPHOLIPID + 71.7 atp -> BIOMASS + 71.7 adp + 71.7 pi
