# Supplementary material for: Genome-scale reconstruction and in silico analysis of Klebsiella oxytoca for 2,3-butanediol production
Source: Microb Cell Fact. 2013 Feb 23;12:20. doi: 10.1186/1475-2859-12-20 (PMC3602198; doi:10.1186/1475-2859-12-20)
Supplement: Additional file 2 — List of metabolites in the genome-scale metabolic model of Klebsiella oxytoca. [file 1475-2859-12-20-S2.pdf]

Additional file 2. List of metabolites in the genome-scale metabolic model of *Klebsiella oxytoca*

Abbreviation	Name	Name(sub)	Formula
12dhmetpeno	1,2-Dihydroxy-5-(methylthio)pent-1-en-3-one		C6H10O3S
12ppd-R	(R)-Propane-1,2-diol	(R)-1,2-Propanediol/(R)-Propylene glycol	C3H8O2
12ppd-S	(S)-Propane-1,2-diol	(S)-1,2-Propanediol/(S)-Propylene glycol	C3H8O2
13dapro	1,3-Diaminopropane	Trimethylenediamine/1,3-Propanediamine/Propane-1,3-diamine	C3H10N2
13pdg	3-Phospho-D-glyceroyl phosphate	1,3-Bisphospho-D-glycerate/(R)-2-Hydroxy-3-(phosphonooxy)-1-monoanhydride with phosphoric propanoic acid	C3H8O10P2
13pdo	Propane-1,3-diol	1,3-Propanediol/Trimethylene glycol	C3H8O2
15dap	1,5-Diaminopentane	Cadaverine/1,5-Pentanediamine/Pentamethylenediamine	C5H14N2
1boh	1-Butanol	n-Butanol	C4H10O
1propanol	Propane-1-ol	Propanol/1-Propanol/Propan-1-ol/Propyl alcohol/1-Hydroxypropane/Ethylcarbinol/n-Propanol/n-Propyl alcohol/Optal/Osmosol extra/UN 1274	C3H8O
23bdo	(R,R)-Butane-2,3-diol	(R,R)-2,3-Butanediol/(R,R)-2,3-Butylene glycol	C4H10O2
23camp	2',3'-Cyclic AMP		C10H12N5O6P
23ccmp	2',3'-Cyclic CMP		C9H12N3O7P
23cgmp	2',3'-Cyclic GMP		C10H12N5O7P
23cump	2',3'-Cyclic UMP		C9H11N2O8P
23d23dhb	(2S,3S)-2,3-Dihydro-2,3-dihydroxybenzoate	(2S,3S)-2,3-Dihydroxy-2,3-dihydroxybenzoate	C7H8O4
23dhb	2,3-Dihydroxybenzoate	2,3-Dihydroxybenzoic acid	C7H6O4
23dhba	(2,3-Dihydroxybenzoyl)adenylate		C17H18N5O10P
23dkgul	2,3-Diketo-L-gulonate	(4R,5S)-4,5,6-Trihydroxy-2,3-dioxohexanoate	C6H8O7
24da6hat	2,4-Diamino-6-hydroxylaminotoluene		C7H11N3O
24dab	L-2,4-Diaminobutanoate	alpha,gamma-Diaminobutyrate/L-2,4-Diaminobutyrate	C4H10N2O2
24danit	2,4-Diamino-6-nitrotoluene		C7H9N3O2
24dhhepd	2,4-Dihydroxyhept-2-enedioate	2,4-Dihydroxyhept-2-1,7-dioate/2,4-Dihydroxyhept-2-enedioic acid	C7H10O6
25dhglucn	2,5-Didehydro-D-gluconate	2,5-Diketogluconic acid	C6H8O7
25drapp	2,5-Diamino-6-(5'-phosphoribosylamino)-4-pyrimidineone		C9H16N5O8P
26dap-LL	LL-2,6-Diaminoheptanedioate	LL-2,6-Diaminopimelate/LL-2,6-Diaminopimelic acid	C7H14N2O4
26dap-M	meso-2,6-Diaminoheptanedioate	meso-2,6-Diaminopimelate/meso-2,6-Diaminopimelic acid/meso-Diaminoheptanedioate	C7H14N2O4
2aa	2-Aminoacrylate	Dehydroalanine	C3H5NO2
2ag3pc	2-Acyl-sn-glycero-3-phosphocholine	2-Acylglycero-3-phosphocholine/1-Lysophosphatidylcholine/1-Lysolecithin/3-Lysolecithin	C9H20NO7PR
2ag3pe	2-Acyl-sn-glycero-3-phosphoethanolamine	L-1-Lysophosphatidylethanolamine/O-(2-Acyl-sn-glycero-3-phospho)-ethanolamine/2-Acyl-sn-glycero-3-phosphoethanolamine	C6H13NO7PR
2ag3pg	2-Acyl-sn-glycero-3-phosphoglycerol		
2ag3ps	2-Acyl-sn-glycero-3-phosphoserine		C8H13NO10PR
2amepp	2-Aminoethylphosphonate	(2-Aminoethyl)phosphonate/Ciliatine	C2H8NO3P
2aobut	L-2-Amino-3-oxobutanoate	L-2-Amino-3-oxobutanoic acid/L-2-Amino-acetoacetate/(S)-2-Amino-3-oxobutanoic acid	C4H7NO3
2chmac	2-Chloromaleylacetate		C6H5ClO5
2cpr5p	1-(2-Carboxyphenylamino)-1'-deoxy-D-ribulose 5'-phosphate		C12H16NO9P
2d3dgalctn	2-Dehydro-3-deoxy-D-galactonate		C6H10O6
2d3dgalctn6p	2-Dehydro-3-deoxy-D-galactonate 6-phosphate		C6H11O9P
2d3drmn	2-Dehydro-3-deoxy-L-rhamnonate		C6H10O5
2d5kglcn	2-Deoxy-5-keto-D-gluconic acid	DKH	C6H10O6
2d5kglcn6p	2-Deoxy-5-keto-D-gluconic acid 6-phosphate	6-Phospho-5-dehydro-2-deoxy-D-gluconate/DKHP	C6H11O9P
2dmmq8	2-Demethylmenaquinone		C15H14O2(C5H8)n
2dmmql8	2-Demethylmenaquinol 8		C15H14O2(C5H8)n
2dr5p	2-Deoxy-D-ribose 5-phosphate		C5H11O7P
2e5mhdcoa	(2E)-5-Methylhexa-2,4-dienoyl-CoA		C28H44N7O17P3S
2epoxaccoa	2-(1,2-Epoxy-1,2-dihydrophenyl)acetyl-CoA		C29H42N7O18P3S
2h6kated	2-Hydroxy-6-ketononatrienedioate		C9H8O6
2h6oxdiend	2-Hydroxy-6-oxonona-2,4-diene-1,9-dioate		C9H10O6
2hba	2-Hydroxybutanoic acid	2-Hydroxybutyrate/2-Hydroxybutyric acid	C4H8O3
2hhpdd	2-Hydroxyhepta-2,4-dienedioate	2-Hydroxyhepta-2,4-diene-1,7-dioate	C7H8O5
2hlmdnit	2-Hydroxylamino-4,6-dinitrotoluene		C7H7N3O5
2hmuc	2-Hydroxymuconate		C6H6O5
2hpa	2-Hydroxyphenylacetate		C8H8O3

2ippm	2-Isopropylmaleate	beta-Isopropylmaleate	C7H10O4
2kgluc	2-Keto-D-gluconic acid	2-Dehydro-D-gluconate/2-Dehydro-D-gluconic acid/alpha-D-arabino-2-Hexulosonic acid	C6H10O7
2kmb	4-Methylthio-2-oxobutanoate	4-Methylthio-2-oxobutanoic acid	C5H8O3S
2m2ecoa	2-Methylbut-2-enoyl-CoATiglyl-CoA	(E)-2-Methylcrotonoyl-CoA/Methylcrotonoyl-CoA/Methylcrotonyl-CoA/Tigloyl-CoA/2-Methylcrotanoyl-CoA	C26H42N7O17P3S
2maaccoa	2-Methylacetoacetyl-CoA	2-Methyl-3-acetoacetyl-CoA	C26H42N7O18P3S
2mac	2-Maleylacetate	4-Oxohex-2-enedioate/Maleylacetate	C6H6O5
2mm	2-Methylmaleate	Citraconate/Citraconic acid/Methylmaleic acid	C5H6O4
2mp2ecoa	2-Methylprop-2-enoyl-CoA	Methacrylyl-CoA/Methylacrylyl-CoA	C25H40N7O17P3S
2napald	2-Naphthaldehyde	2-Naphthalenecarboxaldehyde	C11H8O
2napthm	(2-Naphthyl)methanol	2-Naphthalenemethanol/2-Hydroxymethylnaphthalene	C11H10O
2o3e	2-Oxohept-3-enedioate	2-Oxohept-3-ene-1,7-dioate	C7H8O5
2oad	2-Oxoadipate	2-Oxoadipic acid	C6H8O5
2odhfac	2-Oxo-2,3-dihydrofuran-5-acetate	3-Oxoadipate enol-lactone/4,5-Dihydro-5-oxofuran-2-acetate/5-Oxo-4,5-dihydrofuran-2-acetate	C6H6O4
2ombzl	2-Octaprenyl-6-methoxy-1,4-benzoquinol		C47H72O3
2omhmbl	2-Octaprenyl-3-methyl-5-hydroxy-6-methoxy-1,4-benzoquinone		C48H72O4
2ommbl	2-Octaprenyl-3-methyl-6-methoxy-1,4-benzoquinone		C48H72O3
2op6hp	2-Octaprenyl-6-hydroxyphenol		C46H70O2
2opmp	2-Octaprenyl-6-methoxyphenol		C47H72O2
2opp	2-Octaprenylphenol		C46H70O
2oxpyaccoa	2-Oxepin-2(3H)-ylideneacetyl-CoA		C29H42N7O18P3S
2p1a	2-Propyn-1-al		C3H2O
2pcdpmde	2-phospho-4-(cytidine 5'-diphospho)-2-C-methyl-D-erythritol		C14H26N3O17P3
2pg	D-Glycerate 2-phosphate	2-Phospho-D-glycerate	C3H7O7P
2ppg	2-Phosphoglycolate	Phosphoglycolic acid	C2H5O6P
2py46dc	2-Pyrone-4,6-dicarboxylate		C7H4O6
34dhb	3,4-Dihydroxybenzoate	3,4-Dihydroxybenzoic acid/Protocatechuate/Protocatechuic acid	C7H6O4
34dhma	3,4-Dihydroxymandelaldehyde		C8H8O4
34dhpac	3,4-Dihydroxyphenylacetaldehyde	Protocatechuatealdehyde	C8H8O3
34dhpeg	3,4-Dihydroxyphenylethyleneglycol		C8H10O4
34dhpheac	3,4-Dihydroxyphenylacetate	3,4-Dihydroxyphenylacetic acid/3,4-Dihydroxyphenyl acetate/3,4-Dihydroxyphenyl acetic acid/Homoprotocatechuate	C8H8O4
35dbr4hb	3,5-Dibromo-4-hydroxybenzamide		C7H5Br2NO2
3a2op	3-Amino-2-oxopropyl phosphate	1-Amino-3-(phosphohydroxy)propan-2-one	C3H8NO5P
3amp	3'-AMP	3'-Adenylic acid/3'-Adenosine monophosphate/Adenosine-3'-monophosphate/Adenosine 3'-phosphate/AMP 3'-phosphate	C10H14N5O7P
3b1a	3-Butyn-1-al		C4H4O
3btecoa	3-Butenoyl-CoA	Vinylacetyl-CoA/But-3-enoyl-CoA	C25H40N7O17P3S
3but	3-Butynoate		C4H4O2
3c2hmp	3-Carboxy-2-hydroxy-4-methylpentanoate	(2R,3S)-3-Isopropylmalate/3-Isopropylmalate/2-D-threo-Hydroxy-3-carboxy-isocaproate	C7H12O5
3c3hmp	3-Carboxy-3-hydroxy-4-methylpentanoate	(2S)-2-Isopropylmalate/2-Isopropylmalate/2-Isopropylmalic acid/3-Carboxy-3-hydroxy-isocaproate/3-Carboxy-3-hydroxyisocaproate/2-Hydroxy-2-isopropylbutanedioate/3-Hydroxy-4-methyl-3-carboxypentanoate	C7H12O5
3chroald	3-Chloroallyl aldehyde	trans-3-Chloroallyl aldehyde	C3H3ClO
3cmp	3'-CMP	Cytidine 3'-phosphate	C9H14N3O8P
3ddah7p	2-Dehydro-3-deoxy-D-arabino-heptonate 7-phosphate	3-Deoxy-D-arabino-hept-2-ulosonate 7-phosphate/3-Deoxy-arabino-heptulonate 7-phosphate/3-Deoxy-D-arabino-heptulosonic acid 7-phosphate/DAHP/2-Dahp	C7H13O10P
3dhg	3-Dehydro-L-gulonate		C6H10O7
3dhg6p	3-Dehydro-L-gulonate 6-phosphate		C6H11O10P
3gmp	Guanosine 3'-phosphate	3'-GMP/3'-Guanylic acid/Guo-3'-P/Gp	C10H14N5O8P
3h5m4ecoa	3-Hydroxy-5-methylhex-4-enoyl-CoA		C28H46N7O18P3S
3han	3-Hydroxyanthranilate	3-Hydroxyanthranilic acid	C7H7NO3
3hbcoa	(S)-3-Hydroxybutanoyl-CoA	(S)-3-Hydroxybutyryl-CoA	C25H42N7O18P3S
3hcinnm	trans-3-Hydroxycinnamate	3-Coumaric acid	C9H8O3
3hdmmhcoa	3-Hydroxy-2,6-dimethyl-5-methylene-heptanoyl-CoA		C31H52N7O18P3S
3hibcoa	(S)-3-Hydroxyisobutyryl-CoA		C25H42N7O18P3S
3hivcoa	3-Hydroxyisovaleryl-CoA	3-Hydroxyisovaleryl coenzyme A	C26H44N7O18P3S
3hmrsACP	(3R)-3-Hydroxytetradecanoyl-[acyl-carrier protein]	(R)-3-Hydroxytetradecanoyl-[acyl-carrier protein]/beta-Hydroxymyristyl-[acyl-carrier protein]/HMA	C14H27O2SR

3hpcoa	3-Hydroxypropionyl-CoA	3-Hydroxypropionyl coenzyme A/3-Hydroxypropanoyl-CoA/3-Hydroxypropanoyl coenzymeA	C24H40N7O18P3S
3hpppn	3-(3-Hydroxy-phenyl)-propanoic acid	Dihydro-3-coumaric acid/3-Hydroxyphenylpropanoate	C9H10O3
3hprop	3-Hydroxypropanal		C3H6O2
3ig3p	C1-(3-Indolyl)-glycerol 3-phosphate	Indoleglycerol phosphate/1-C-(Indol-3-yl)glycerol 3-phosphate/(3-Indolyl)-glycerol phosphate/(1S,2R)-1-C-(Indol-3-yl)glycerol 3-phosphate/Indole-3-glycerol phosphate	C11H14NO6P
3mccoa	3-Methylcrotonyl-CoA	3-Methylbut-2-enoyl-CoA/3-Methylcrotonoyl-CoA/Dimethylacryloyl-CoA	C26H42N7O17P3S
3metpro	3-(Methylthio)propionic acid	3-Methylthiopropionate	C4H8O2S
3mlac	3-Mercaptolactate		C3H5O3S
3mob	3-Methyl-2-oxobutanoate	3-Methyl-2-oxobutanoic acid/3-Methyl-2-oxobutyric acid/2-Oxo-3-methylbutanoate/2-Oxoisovalerate/2-Oxoisopentanoate/alpha-Ketovaline/2-Ketovaline/2-Keto-3-methylbutyric acid	C5H8O3
3mop	(S)-3-Methyl-2-oxopentanoate	(S)-3-Methyl-2-oxopentanoic acid/(3S)-3-Methyl-2-oxopentanoic acid/(3S)-3-Methyl-2-oxopentanoate	C6H10O3
3op4hb	3-Octaprenyl-4-hydroxybenzoate		C47H70O3
3opp	3-Oxopropanoate	Malonate semialdehyde	C3H4O3
3oppcoa	3-Oxopropionyl-CoA		C24H38N7O18P3S
3oxdscoa	3-Oxo-5,6-dehydrosuberyl-CoA		C29H44N7O20P3S
3oxdscoasa	3-Oxo-5,6-dehydrosuberyl-CoA semialdehyde		C29H44N7O19P3S
3pg	3-Phospho-D-glycerate	D-Glycerate 3-phosphate/3-Phospho-(R)-glycerate	C3H7O7P
3php	3-Phosphohydroxypyruvate	3-Phosphonooxypyruvate/3-Phosphonooxypyruvic acid/3-Phosphohydroxypyruvic acid	C3H5O7P
3psme	5-O-(1-Carboxyvinyl)-3-phosphoshikimate	O5-(1-Carboxyvinyl)-3-phosphoshikimate	C10H13O10P
3sfpyr	3-Sulfinylpyruvate	3-Sulfinopyruvate	C3H4O5S
3slala	3-Sulfino-L-alanine	L-Cysteinesulfinic acid/3-Sulphino-L-alanine/3-Sulfinoalanine	C3H7NO4S
3spyr	3-Sulfopyruvate	3-Sulfopyruvic acid	C3H4O6S
3udsb	3-Ureidoisobutyrate		C5H10N2O3
3ump	3'-UMP	Uridine 3'-monophosphate/Uridine 3'-phosphate	C9H13N2O9P
3urdpp	3-Ureidopropionate	3-Ureidopropanoate/beta-Ureidopropionic acid/N-Carbamoyl-beta-alanine	C4H8N2O3
45dhpen	(4S)-4,5-Dihydroxypentan-2,3-dione		C5H8O4
4aabut	4-Acetamidobutanoate	N4-Acetylaminobutanoate	C6H11NO3
4ab	4-Aminobutanal	4-Aminobutyraldehyde/Butyraldehyde, 4-amino-	C4H9NO
4ampm	4-Amino-2-methyl-5-phosphomethylpyrimidine	4-Amino-5-phosphomethyl-2-methylpyrimidine	C6H10N3O4P
4c2hhd	4-Carboxy-2-hydroxyhexa-2,4-dienedioate		C7H6O7
4faac	4-Fumarylacetoacetate	4-Fumarylacetoacetic acid/Fumarylacetoacetate	C8H8O6
4flrbz	4-Fluorobenzoate	4-Fluorobenzoic acid	C7H5FO2
4fmuclac	4-Fluoromuconolactone		C6H5FO4
4hac	4-Hydroxyphenylacetaldehyde	2-(4-Hydroxyphenyl)acetaldehyde	C8H8O2
4hb	4-Hydroxybenzoate	Hydroxybenzoic acid/4-Hydroxybenzoic acid/Hydroxybenzenecarboxylic acid	C7H6O3
4hbt	4-Hydroxybutanoic acid	4-Hydroxybutanoate/4-Hydroxybutyric acid	C4H8O3
4hbza	4-Hydroxybenzyl alcohol	p-Hydroxybenzyl alcohol/p-Methylolphenol	C7H8O2
4hglusa	L-4-Hydroxyglutamate semialdehyde		C5H9NO4
4hlmdnit	4-Hydroxylamino-2,6-dinitrotoluene		C7H7N3O5
4hlt	4-Hydroxy-L-threonine		C4H9NO4
4hmcatech	4-Hydroxymethylcatechol		C7H8O3
4hmsalc	4-Hydroxymethylsalicylate	2-Hydroxy-4-hydroxymethylbenzoic acid	C8H8O4
4hpheac	4-Hydroxyphenylacetate	4-Hydroxyphenylacetic acid	C8H8O3
4hpp	3-(4-Hydroxyphenyl)pyruvate	4-Hydroxyphenylpyruvate, p-Hydroxyphenylpyruvic acid	C9H8O4
4hpro	trans-4-Hydroxy-L-proline		C5H9NO3
4i5p	4-Imidazolone-5-propanoate	4-Imidazolone-5-propionic acid/4,5-Dihydro-4-oxo-5-imidazolepropanoate	C6H8N2O3
4maac	4-Maleylacetoacetate	4-Maleylacetoacetic acid	C8H8O6
4mctch	4-Methylcatechol	3,4-Dihydroxytoluene/1,2-Dihydroxy-4-methylbenzene/4-Methyl-1,2-benzenediol	C7H8O2
4mhetz	5-(2-Hydroxyethyl)-4-methylthiazole	4-Methyl-5-(2'-hydroxyethyl)-thiazole/4-Methyl-5-(2-hydroxyethyl)-thiazole	C6H9NOS
4mop	4-Methyl-2-oxopentanoate	2-Oxoisocaproate	C6H10O3
4obtc	(E)-4-Oxobut-1-ene-1,2,4-tricarboxylate	4-Oxalomesaconate/4-Oxalmesaconic acid	C7H6O7
4ppcys	N-[(R)-4'-Phosphopantothenoyl]-L-cysteine	(R)-4'-Phosphopantothenoyl-L-cysteine	C12H23N2O9PS
4ppnte	Pantetheine 4'-phosphate	4'-Phosphopantetheine/Phosphopantetheine/D-Pantetheine 4'-phosphate	C11H23N2O7PS
4ppnto	D-4'-Phosphopantothenate	(R)-4'-Phosphopantothenate	C9H18NO8P

4tmab	4-Trimethylammoniobutanal		C7H16NO
4tmabn	4-Trimethylammoniobutanoate		C7H16NO2
56dht	5,6-Dihydrothymine	Dihydrothymine/5,6-Dihydro-5-methyluracil	C5H8N2O2
56dhu	5,6-Dihydrouracil	2,4(1H,3H)-Pyrimidinedione, dihydro-/Dihydrouracile/Dihydrouracil/5,6-Dihydro-2,4-dihydroxypyrimidine/Hydrouracil	C4H6N2O2
5a4ic	5-Amino-4-imidazolecarboxyamide		C4H6N4O
5aop	5-Amino-4-oxopentanoate	5-Aminolevulinate/5-Amino-4-oxovaleric acid	C5H9NO3
5c2o3e	5-Carboxy-2-oxohept-3-enedioate	5-Oxopent-3-ene-1,2,5-tricarboxylate	C8H8O7
5cm2hm	5-Carboxymethyl-2-hydroxymuconate		C8H8O7
5cm2hmsa	2-Hydroxy-5-carboxymethylmuconate semialdehyde	5-Carboxymethyl-2-hydroxymuconate semialdehyde/5-Carboxymethyl-2-hydroxymuconic semialdehyde	C8H8O6
5dglucn	5-Deoxy glucuronic acid		C6H10O6
5dhgluc	5-Dehydro-D-gluconate	5-Dehydrogluconate	C6H10O7
5fmuclac	5-Fluoromuconolactone		C6H5FO4
5fthf	5-Formyltetrahydrofolate	L(-)-5-Formyl-5,6,7,8-tetrahydrofolic acid/Folinic acid	C20H23N7O7
5h2o4uic	5-Hydroxy-2-oxo-4-ureido-2,5-dihydro-1H-imidazole-5-carboxylate		C5H6N4O5
5hiaa	5-Hydroxyindoleacetaldehyde		C10H9NO2
5hiac	5-Hydroxyindoleacetate		C10H9NO3
5hiu	5-Hydroxyisourate		C5H4N4O4
5m3o4hcoa	5-Methyl-3-oxo-4-hexenoyl-CoA		C28H44N7O18P3S
5mc	5-Methylcytosine		C5H7N3O
5mh4ecoa	5-Methylhex-4-enoyl-CoA		C28H46N7O17P3S
5mta	5'-Methylthioadenosine	Methylthioadenosine/S-Methyl-5'-thioadenosine/5-Methylthioadenosine/5'-Deoxy-5'-(methylthio)adenosine/Thiomethyladenosine/MTA	C11H15N5O3S
5mtglu	5-Methyltetrahydropteroyltri-L-glutamate		C30H39N9O12
5mtrib	5-Methylthio-D-ribose	S-Methyl-5-thio-D-ribose	C6H12O4S
5mtribp	S-Methyl-5-thio-D-ribose 1-phosphate	S-Methyl-5-thio-alpha-D-ribose 1-phosphate/S-Methyl-5-thio-5-deoxy-D-ribose 1-phosphate	C6H13O7PS
5mtribup	S-Methyl-5-thio-D-ribulose 1-phosphate		C6H13O7PS
5prdmbz	N1-(5-Phospho-alpha-D-ribosyl)-5,6-dimethylbenzimidazole	alpha-Ribazole 5'-phosphate	C14H19N2O7P
6pgl	6-phospho-D-glucono-1,5-lactone	D-Glucono-1,5-lactone 6-phosphate	C6H11O9P
7m3o6ocoa	7-Methyl-3-oxo-6-octenoyl-CoA		C30H48N7O18P3S
a5p	D-Arabinose 5-phosphate		C5H11O8P
a6rp	4-(1-D-Ribitylamino)-5-aminouracil	4-(1-D-Ribitylamino)-5-amino-2,6-dihydroxypyrimidine	C9H16N4O6
a6rp5p	5-Amino-6-(5'-phosphoribosylamino)uracil	5-Amino-6-(ribosylamino)-2,4-(1H,3H)-pyrimidinedione 5'-phosphate/5-Amino-6-(5-phosphoribosylamino)uracil	C9H15N4O9P
a6rp5p2	5-Amino-6-(5'-phosphoribitylamino)uracil	5-Amino-2,6-dioxy-4-(5'-phosphoribitylamino)pyrimidine/5-Amino-6-(5-phosphoribitylamino)uracil	C9H17N4O9P
aacald	Aminoacetaldehyde		C2H5NO
aacoa	Acetoacetyl-CoA	Acetoacetyl coenzyme A/3-Acetoacetyl-CoA	C25H40N7O18P3S
aACP	Acyl-[acyl-carrier protein]	Long-chain-acyl-[acyl-carrier protein]	C3H4OSR2
aact	Aminoacetone	1-Amino-2-propanone	C3H7NO
abt6p	Arbutin 6-phosphate	Arbutin-6P	C12H17O10P
abut	(S)-2-Aceto-2-hydroxybutanoate	(S)-2-Hydroxy-2-ethyl-3-oxobutanoate	C6H10O4
ac	Acetate	Acetic acid/Ethanoic acid/Glacial acetic acid	C2H4O2
acac	Acetoacetate	3-Oxobutanoic acid/beta-Ketobutyric acid/Acetoacetic acid	C4H6O3
acACP	Acetyl-ACP		C13H23N2O8PRS
acal	Acetaldehyde	Ethanal	C2H4O
acala	N-Acetylmuramoyl-Ala	N-Acetyl-D-muramoyl-L-alanine	C14H24N2O9
accitr	N-Acetyl-L-citrulline		C8H15N3O4
accoa	Acetyl-CoA	Acetyl coenzyme A	C23H38N7O17P3S
acda	Adenosyl cobyrinate a,c diamide	Adenosyl cobyrinate diamide/Adenosylcob(Ⅲ)yrinic acid a,c-diamide/Adenosylcobyrinic acid a,c-diamide	C55H73CoN11O15
acetoin	(R)-Acetoin	(R)-2-Acetoin/(R)-3-Hydroxy-2-butanone/(R)-Dimethylketol/(R)-3-Hydroxybutan-2-one	C4H8O2
acg5p	N-Acetyl-L-glutamyl 5-phosphate	N-Acetyl-L-glutamate 5-phosphate	C7H12NO8P
acglcmpdp	N-Acetyl-D-glucosaminyldiphosphoundecaprenol	N-Acetyl-D-glucosaminyldiphospho-ditrans,octacis-undecaprenol	C63H105NO12P2
acha	Adenosyl cobyrinate hexaamide	Adenosylcobyric acid	C55H77CoN15O11
aclm	Acrylamide	2-Propenamide	C3H5NO
acmur	N-Acetyl-D-muramoate		C11H19NO8
acon-C	cis-Aconitate	cis-Aconitic acid	C6H6O6
aconit	Acrylonitrile	Propenenitrile/Vinyl cyanide	C3H3N
acon-T	trans-Aconitate	trans-Aconitic acid	C6H6O6

ACP	Acyl-carrier protein	ACP/[Acyl-carrier protein]/Holo-[acyl-carrie-protein]	HSR
acputs	N-Acetylputrescine		C6H14N2O
actACP	Acetoacetyl-[acp]	Acetoacetyl-[acyl-carrier protein]	C4H5O2SR
actp	Acetyl phosphate		C2H5O5P
ad	Adenine	6-Aminopurine	C5H5N5
adcba	Adenosyl cobinamide		C58H84CoN16O11
adcbap	Adenosyl cobinamide phosphate		C58H85CoN16O14P
adchor	4-amino-4-deoxychorismate	ADC	C10H11NO5
adip	Adipate	Hexanedioate/Hexan-1,6-dicarboxylate	C6H10O4
adlipo	S-Acetyldihydrolipoamide-E	[Dihydrolipoyllysine-residue acetyltransferase] S-acetyldihydrolipoyllysine	C10H18NO2S2R
adn	Adenosine		C10H13N5O4
adocbl	Adenosylcobalamin	Cobamide coenzyme/Deoxyadenosylcobalamin/Cobamamide/Vitamin B12 coenzyme/5,6-Dimethylbenzimidazolyl-5-deoxyadenosyl-cobamide/(5'-Deoxy-5'-adenosyl)cobamide coenzyme/(5,6-Dimethylbenzimidazolyl)cobamide coenzyme/alpha-(5,6-Dimethylbenzimidazolyl)cobamide coenzyme/5'-Deoxy-5'-adenosylcobalamin/5'-Deoxy-5'-adenosyl vitamin B12/5'-Deoxy-5'-adenosyl-5,6-dimethylbenzimidazolylcobamide/5,6-Dimethylbenzimidazolyl-Co-5'-deoxy-5'-adenosylcobamide/Calomide/Cobalamin coenzyme/Coenzyme B12/DMBC coenzyme/Dibencozide/Funacomide	C72H100CoN18O17P
adp	ADP	Adenosine 5'-diphosphate	C10H15N5O10P2
adpdgdmhep	ADP-D-glycero-D-manno-heptose		C17H27N5O16P2
adpglc	ADP-glucose	Adenosine diphosphoglucose	C16H25N5O15P2
adphep	ADP-L-glycero-D-manno-heptose		C17H27N5O16P2
adprib	ADP-D-ribose		C15H23N5O14P2
ag	L-Arogenate	L-Arogenic acid/Pretyrosine	C10H13NO5
agdpcba	Adenosine-GDP-cobinamide	Adenosylcobinamide-GDP	C68H97CoN21O21P2
agl3p	1-Acyl-sn-glycerol 3-phosphate		C4H8O7PR
agmatine	Agmatine	(4-Aminobutyl) guanidine	C5H14N4
ahdt	2-Amino-4-hydroxy-6-(erythro-1,2,3-trihydroxypropyl)dihydropteridine triphosphate	6-(L-erythro-1,2-Dihydroxypropyl 3-triphosphate)-7,8-dihydropterin/6-[(1S,2R)-1,2-Dihydroxy-3-triphosphooxypropyl]-7,8-dihydropterin	C9H16N5O13P3
ahhmd	2-Amino-7,8-dihydro-4-hydroxy-6-(diphosphooxymethyl)pteridine	2-Amino-4-hydroxy-6-hydroxymethyl-7,8-dihydropteridine diphosphate/7,8-Dihydropterin pyrophosphate	C7H11N5O8P2
ahhmp	2-Amino-4-hydroxy-6-hydroxymethyl-7,8-dihydropteridine		C7H9N5O2
ahm	4-Amino-5-hydroxymethyl-2-methylpyrimidine	Toxopyrimidine/4-Amino-2-methyl-5-pyrimidinemethanol	C6H9N3O
ahmpp	2-Methyl-4-amino-5-hydroxymethylpyrimidine diphosphate	4-Amino-2-methyl-5-diphosphomethylpyrimidine	C6H11N3O7P2
ahser	O-Acetylhomoserine		C6H11NO4
aicar	1-(5'-Phosphoribosyl)-5-amino-4-imidazolecarboxamide	5'-Phosphoribosyl-5-amino-4-imidazolecarboxamide/5'-Phospho-ribosyl-5-amino-4-imidazole carboxamide/AICAR/5-Aminoimidazole-4-carboxamide ribotide/5-Phosphoribosyl-4-carbamoyl-5-aminoimidazole/5-Amino-1-(5-phospho-D-ribosyl)imidazole-4-carboxamide	C9H15N4O8P
air	Aminoimidazole ribotide	AIR/1-(5'-Phosphoribosyl)-5-aminoimidazole/5'-Phosphoribosyl-5-aminoimidazole/1-(5-Phospho-D-ribosyl)-5-aminoimidazole/5-Amino-1-(5-phospho-D-ribosyl)imidazole	C8H14N3O7P
akg	2-Oxoglutarate	Oxoglutaric acid/2-Ketoglutaric acid/alpha-Ketoglutaric acid	C5H6O5
ala	L-Alanine	L-2-Aminopropionic acid/L-alpha-Alanine	C3H7NO2
alaala	D-Alanyl-D-alanine	D-Ala-D-Ala	C6H12N2O3
alac-S	(S)-2-Acetolactate	(S)-2-Hydroxy-2-methyl-3-oxobutanoate	C5H8O4
alatrna	L-Alanyl-tRNA	L-Alanyl-tRNA(Ala)	C13H22NO11PR2(C5H8O6PR)n
alltn	Allantoin	5-Ureidohydantoin/Glyoxyldiureide	C4H6N4O3
alltt	Allantoate	Allantoic acid	C4H8N4O4
amp	AMP	Adenosine 5'-monophosphate/Adenylic acid/Adenylate/5'-AMP/5'-Adenylic acid/5'-Adenosine monophosphate/Adenosine 5'-phosphate	C10H14N5O7P
amppo	(R)-1-Aminopropan-2-ol	(R)-1-Amino-2-propanol	C3H9NO
amylose	Amylose	Amylose chain/(1,4-alpha-D-Glucosyl)n/(1,4-alpha-D-Glucosyl)n+1/(1,4-alpha-D-Glucosyl)n-1/4-{(1,4)-alpha-D-Glucosyl}(n-1)-D-glucose/1,4-alpha-D-Glucan	(C6H10O5)n

an	Anthranilate	Anthranilic acid/o-Aminobenzoic acid/Vitamin L1/2-Aminobenzoate	C7H7NO2
ao4pob	2-Amino-3-oxo-4-phosphonooxybutyrate	L-2-Amino-3-oxo-4-phosphonooxybutyrate/(2S)-2-Amino-3-oxo-4-phosphonooxybutanoate	C4H8NO7P
aona	8-Amino-7-oxononanoate	8-Amino-7-oxononanoic acid	C9H17NO3
apoACP	apoprotein [acyl carrier protein]	Apo-[acyl-carrier-protein]	RHO
apoCAB	Apo-[carboxylase]		C7H13N3O2R2
appppa	P1,P4-Bis(5'-adenosyl) tetraphosphate	AppppA	C20H28N10O19P4
aps	Adenylyl sulfate	Adenosine 5'-phosphosulfate/APS/5'-Adenylyl sulfate	C10H14N5O10PS
aqcbl3	Aquacob(III)alamin	Aquacobalamin	C62H90CoN13O15P
arabn	L-Arabinonate		C5H10O6
arabnlac	L-Arabinono-1,4-lactone	L-Arabonolactone	C5H8O5
arg	L-Arginine	(S)-2-Amino-5-guanidinovaleric acid	C6H14N4O2
argsucc	N-(L-Arginino)succinate	N(omega)-(L-Arginino)succinate/L-Argininosuccinate/L-Argininosuccinic acid/L-Arginosuccinic acid	C10H18N4O6
argtrna	L-Arginyl-tRNA(Arg)	L-Arginyl-tRNA	C21H33N9O11PR(C5H8O6PR)n
ascb6p	L-Ascorbate 6-phosphate		C6H9O9P
aselnt	Adenylylselenate	Adenosine-5'-phosphoselenate	C10H14N5O10PSe
aser	O-Acetyl-L-serine	O3-Acetyl-L-serine	C5H9NO4
asn	L-Asparagine	2-Aminosuccinamic acid	C4H8N2O3
asntrna	L-Asparaginyl-tRNA(Asn)	Asn-tRNA(Asn)/Asparaginyl-tRNA(Asn)	C14H23N2O12PR2(C5H8O6PR)n
aso3	arsenite		AsO3
asp	L-Aspartate	L-Aspartic acid/2-Aminosuccinic acid	C4H7NO4
asp-D	D-Aspartate	D-Aspartic acid	C4H7NO4
aspsa	L-Aspartate 4-semialdehyde	Aspartate beta-semialdehyde/L-Aspartic 4-semialdehyde	C4H7NO3
asptrna	L-Aspartyl-tRNA(Asp)		C14H22NO13PR2(C5H8O6PR)n
asuc	N6-(1,2-Dicarboxyethyl)-AMP	Adenylosuccinate/Adenylosuccinic acid	C14H18N5O11P
athr	L-Allo-threonine	L-allo-Threonine	C4H9NO3
atp	ATP	Adenosine 5'-triphosphate	C10H16N5O13P3
b5amp	Biotinyl-5'-AMP		C20H28N7O9PS
bal	Betaine aldehyde		C5H12NO
bala	beta-Alanine	3-Aminopropionic acid/3-Aminopropanoate	C3H7NO2
bapa	beta-Aminopropion aldehyde	3-Aminopropanal	C3H7NO
basp	4-Phospho-L-aspartate	L-4-Aspartyl phosphate	C4H8NO7P
bbtcoa	gamma-butyrobetainyl-CoA		C28H46N8O17P3S
benzot	Benzoate	Benzoic acid/Benzenecarboxylic acid/Phenylformic acid/Dracylic acid	C7H6O2
bgalgcer	beta-D-Galactosyl-1,4-beta-D-glucosylceramide	Lactosylceramide/Gal-beta1->4Glc-beta1->1'Cer/LacCer/Lactosyl-N-acylsphingosine/D-Galactosyl-1,4-beta-D-glucosylceramide	C31H56NO13R
bglycogen	branching glycogen		(C6H10O5)n
bromox	Bromoxynil	3,5-Dibromo-4-hydroxybenzonitrile	C7H3Br2NO
bt	Biotin	D-Biotin/Vitamin H/Coenzyme R	C10H16N2O3S
butanal	Butanal	Butyraldehyde	C4H8O
bzamid	Benzamide		C7H7NO
bzonit	Benzonitrile	Phenyl cyanide/Cyanobenzene	C7H5N
bzop	Benzoyl phosphate		C7H7O5P
c040coa	Butanoyl-CoA	Butyryl-CoA	C25H42N7O17P3S
c120	Dodecanoate	Dodecanoic acid/Dodecylcarboxylate/Lauric acid	C12H24O2
c120ACP	Dodecanoyl-[acyl-carrier protein]	Dodecanoyl-[acp]/Lauroyl-[acyl-carrier protein]	C12H23OSR
c130	Tridecanoic acid	Tridecanoate	C13H26O2
c130ACP	Tridecanoyl-[acyl-carrier protein]		C13H25OSR
c140	Tetradecanoic acid	Tetradecanoate/Myristic acid	C14H28O2
c140ACP	Myristoyl-[acyl-carrier protein]	Tetradecanoyl-[acp]/Tetradecanoyl-[acyl-carrier protein]	C14H27OSR
c141	Myristoleic acid	(9Z)-Tetradecenoic acid/9-Tetradecenoic acid/(Z)-Tetradec-9-enoic acid	C14H26O2
c141ACP	cis-tetradec-7-enoyl-[acyl-carrier protein] (n-C14:1)		C25H45N2O8PRS
c150	pentadecanoic acid (C15:0)	Pentadecylic acid/n-Pentadecanoic acid	C15H30O2
c150ACP	pentadecanoyl-ACP (C15:0 ACP)		C26H49N2O8PRS
c151	pentadecenoic acid (C15:1)		C15H28O2
c151ACP	pentadecenoyl-ACP (C15:1 ACP)		C26H47N2O8PRS
c160	Hexadecanoate	Hexadecanoic acid/Hexadecylic acid/Palmitic acid/Palmitate/Cetylic acid	C16H32O2
c160ACP	Palmitoyl-ACP (n-C16:0ACP)	Hexadecanoyl-[acp]/Hexadecanoyl-[acyl-carrier protein]	C16H31OSR
c161	hexadecenoate (n-C16:1)	(9Z)-Hexadecenoic acid/ cis-9-Hexadecenoic acid/ Palmitoleic acid	C16H30O2
c161ACP	cis-hexadec-9-enoyl-[acyl-carrier protein] (n-C16:1)		C27H49N2O8PRS
c170	heptadecanoic acid (C17:0)		C17H34O2
c170ACP	heptadecanoyl-ACP (C17:0 ACP)		C28H53N2O8PRS

c171	Heptadecenoic acid (C17:1)		C17H32O2
c171ACP	heptadecenoyl-ACP (C17:1 ACP)		C28H51N2O8PRS
c180	Octadecanoic acid	Stearate/Stearic acid	C18H36O2
c180ACP	Octadecanoyl-ACP (n-C18:0ACP)		C29H55N2O8PRS
c181	(9Z)-Octadecenoic acid	(Z)-Octadec-9-enoic acid/Oleate/Oleic acid	C18H34O2
c181ACP	cis-octadec-11-enoyl-[acyl-carrier protein] (n-C18:1)		C29H53N2O8PRS
c190	nonadecanoic acid (C19:0)		C19H38O2
c190ACP	nonadecanoyl-ACP (C19:0 ACP)		C30H57N2O8PRS
c2ch4cmo	cis-2-Chloro-4-carboxymethylenebut-2-en-1,4-olide	cis-2-Chlorodienelactone	C6H3ClO4
c2mipdcoa	cis-2-Methyl-5-isopropylhexa-2,5-dienoyl-CoA		C31H50N7O17P3S
c3chp	cis-3-Chloro-2-propene-1-ol		C3H5ClO
c3chroald	cis-3-Chloroallyl aldehyde		C3H3ClO
c3chroc	cis-3-Chloroacrylic acid		C3H3ClO2
c4cmbo	cis-4-Carboxymethylenebut-2-en-4-olide	4-Carboxymethylenebut-2-en-4-olide	C6H4O4
caasp	N-Carbamoyl-L-aspartate		C5H8N2O5
cabm	Carbamate	Carbamic acid/Aminoformic acid	CH3NO2
cacac	cis-Acetylacrylate		C5H6O3
cair	1-(5-Phospho-D-ribosyl)-5-amino-4-imidazolecarboxylate	1-(5'-Phosphoribosyl)-5-amino-4-imidazolecarboxylate/1-(5'-Phosphoribosyl)-5-amino-4-carboxyimidazole/5'-Phosphoribosyl-5-amino-4-imidazolecarboxylate/1-(5'-Phosphoribosyl)-4-carboxy-5-aminoimidazole/5'-Phosphoribosyl-4-carboxy-5-aminoimidazole/5-Amino-1-(5-phospho-D-ribosyl)imidazole-4-carboxylate	C9H14N3O9P
caiz	5-carboxyamino-1-(5-phospho-D-ribosyl)imidazole		C9H14N3O9P
camp	cAMP	3',5'-Cyclic AMP/Cyclic adenylic acid/Cyclic AMP/Adenosine 3',5'-phosphate	C10H12N5O6P
cap	Carbamoyl phosphate		CH4NO5P
CARBO	carbohydrate		
carccm	3-Carboxy-cis,cis-muconate	beta-Carboxy-cis,cis-muconate/cis,cis-Butadiene-1,2,4-tricarboxylate	C7H6O6
carpcoa	5-Carboxy-2-pentenoyl-CoA		C27H42N7O19P3S
catech	Catechol	1,2-Benzenediol/o-Benzenediol/1,2-Dihydroxybenzene/Brenzcatechin/Pyrocatechol	C6H6O2
CAV	cofactors and vitamines		
cbi	Cobinamide		C48H72CoN11O8
cbl1	Cob(I)alamin	Cbl/Vitamin B12s	C62H89CoN13O14P
cbl2	Cob(II)alamin	Vitamin B12r	C62H88CoN13O14P
ccmuc	cis,cis-Muconate	cis,cis-Hexadienedioate/cis,cis-2,4-Hexadienedioic acid	C6H6O4
ccoa	Crotonoyl-CoA	Crotonyl-CoA/2-Butenoyl-CoA/trans-But-2-enoyl-CoA/But-2-enoyl-CoA	C25H40N7O17P3S
cdp	CDP	Cytidine 5'-diphosphate/Cytidine diphosphate	C9H15N3O11P2
cdpdg	CDP-diacylglycerol	CDP-1,2-diacylglycerol/1,2-Diacyl-sn-glycero-3-cytidine-5'-diphosphate	C14H19N3O15P2R2
cdpetn	CDP-ethanolamine		C11H20N4O11P2
cdpmde	4-(Cytidine 5'-diphospho)-2-C-methyl-D-erythritol		C14H25N3O14P2
cellobiose	Cellobiose	1-beta-D-Glucopyranosyl-4-D-glucopyranose	C12H22O11
cellulose_n	Cellulose(n)	(1,4-beta-D-Glucosyl)n/(1,4-beta-D-Glucosyl)n+1/(1,4-beta-D-Glucosyl)n-1/1,4-beta-D-Glucan/Microcrystalline cellulose	(C6H10O5)n
cellulose_n_1	Cellulose(n-1)	(1,4-beta-D-Glucosyl)n/(1,4-beta-D-Glucosyl)n+1/(1,4-beta-D-Glucosyl)n-1/1,4-beta-D-Glucan/Microcrystalline cellulose	(C6H10O5)n
cgmp	3',5'-Cyclic GMP	Guanosine 3',5'-cyclic monophosphate/Guanosine 3',5'-cyclic phosphate/Cyclic GMP/cGMP	C10H12N5O7P
chac	Chloroacetic acid	Chloroethanoic acid	C2H3ClO2
chacald	Chloroacetaldehyde	2-Chloroethanal	C2H3ClO
chitobiose	Chitobiose	Diacetylchitobiose/N,N'-Diacetylchitobiose	C16H28N2O11
choline	Choline	Bilineurine	C5H14NO
chor	chorismate	Chorismic acid	C10H10O6
cinnm	trans-Cinnamate	trans-Cinnamic acid/(E)-Cinnamate	C9H8O2
cit	Citrate	Citric acid/2-Hydroxy-1,2,3-propanetricarboxylic acid/2-Hydroxytricarballylic acid	C6H8O7
citr	L-Citrulline	2-Amino-5-ureidovaleric acid/Citrulline	C6H13N3O3
ckdo	CMP-3-deoxy-D-manno-octulosonate	CMP-KDO	C17H26N3O15P
cl	Chloride		Cl
clpn	cardiolipin	Cardiolipin/Diphosphatidylglycerol/1',3'-Bis(1,2-diacyl-sn-glycero-3-phospho)-sn-glycerol	C13H18O17P2R4
cmp	CMP	Cytidine-5'-monophosphate/Cytidylic acid	C9H14N3O8P
cn	Cyanide	Prussiate/CN-/Cyano	CN
co	CO	Carbon monoxide	CO

co2	CO2	Carbon dioxide	CO2
coa	Coenzyme A	CoA/CoA-SH	C21H36N7O16P3S
cobacd	Cob(I)yrinate a,c diamide	Cob(I)yrinate diamide/Cob(I)yrinic acid a,c-diamide	C45H61CoN6O12
cobalt2	Co2+	Cobalt	Co
cobrin	Cobyrinate	Cobyrinic acid/Cob(II)yrinate/Cob(II)yrinic acid	C45H59CoN4O14
cobrindi	Cob(II)yrinate a,c diamide	Cob(II)yrinate diamide/Cob(II)yrinic acid a,c-diamide/Cobyrinate a,c-diamide/Cobyrinic acid a,c-diamide	C45H61CoN6O12
cobtpc	Cobalt-precorrin 2	Cobalt-sirohydrochlorin	C42H46CoN4O16
cobtpc3	Cobalt-precorrin 3		C43H48CoN4O16
cobtpc4	Cobalt-precorrin 4		C44H50CoN4O16
cobtpc5a	Cobalt-precorrin 5A		C45H52CoN4O16
cobtpc5b	Cobalt-precorrin 5B		C43H50CoN4O16
cobtpc6	Cobalt-precorrin 6	Cobalt-precorrin 6A	C44H53CoN4O16
cobtpc6b	Cobalt-dihydro-precorrin 6	Cobalt-precorrin 6B	C44H55CoN4O16
cobtpc7	Cobalt-precorrin 7		C45H57CoN4O16
cobtpc8	Cobalt-precorrin 8		C45H59CoN4O14
cofac3	Cobalt-factor III		C43H46CoN4O16
cpp	Coproporphyrinogen III		C36H44N4O8
cppi	Coproporphyrinogen I		C36H44N4O8
creatine	Creatine	alpha-Methylguanidino acetic acid/Methylglycocyamine	C4H9N3O2
cretn	Creatinine	1-Methylglycocyamidine	C4H7N3O
crn	L-Carnitine	L-gamma-Trimethyl-beta-hydroxybutyrobetaine/Vitamin BT/3-Carboxy-2-hydroxy-N,N,N-trimethyl-1-propanaminium hydroxide, inner salt/Levocarnitine/(R)-Carnitine	C7H15NO3
crncoa	L-Carnitinyl-CoA		C28H46N8O18P3S
ct	Cytosine		C4H5N3O
ctbt	4-(Trimethylammonio)but-2-enoate		C7H14NO2
ctbtcoa	crotonobetainyl-CoA		C28H44N8O17P3S
ctp	CTP	Cytidine 5'-triphosphate/Cytidine triphosphate	C9H16N3O14P3
cvn	Cinnavalininate		C14H8N2O6
cyaala	3-Cyano-L-alanine	L-3-Cyanoalanine/L-beta-Cyanoalanine	C4H6N2O2
cys	L-Cysteine	L-2-Amino-3-mercaptopropionic acid	C3H7NO2S
cysgly	Cys-Gly	L-Cysteinylglycine	C5H10N2O3S
cyst	L-Cystine	L-Dicysteine/L-alpha-Diamino-beta-dithiolactic acid	C6H12N2O4S2
cysteate	L-Cysteate	L-Cysteic acid/3-Sulfoalanine/2-Amino-3-sulfopropionic acid	C3H7NO5S
cystrna	L-Cysteinyl-tRNA(Cys)		C18H26N6O11PSR(C5H8O6PR)n
cytd	Cytidine		C9H13N3O5
d1ap2oop	D-1-Aminopropan-2-ol O-phosphate	(R)-1-Aminopropan-2-yl phosphate	C3H10NO4P
d2naptcoa	1,4-Dihydroxy-2-naphthoyl-CoA		C32H42N7O19P3S
d3dg	2-Dehydro-3-deoxy-D-glucarate		C6H8O7
d4dg	5-Dehydro-4-deoxy-D-glucarate		C6H8O7
d6pgc	6-Phospho-D-gluconate		C6H13O10P
da	Deoxyadenosine	2'-Deoxyadenosine	C10H13N5O3
da-5	5'-Deoxyadenosine		C10H13N5O3
dadp	dADP	2'-Deoxyadenosine 5'-diphosphate	C10H15N5O9P2
dala	D-Alanine	D-2-Aminopropionic acid/D-Ala	C3H7NO2
dalt	D-Altronate		C6H12O7
damp	dAMP	2'-Deoxyadenosine 5'-phosphate/2'-Deoxyadenosine 5'-monophosphate/Deoxyadenylic acid/Deoxyadenosine monophosphate	C10H14N5O6P
danna	7,8-Diaminononanoate		C9H20N2O2
darbt	D-Arabitol	D-Arabinitol/D-Arabinol/D-Lyxitol	C5H12O5
datp	dATP	2'-Deoxyadenosine 5'-triphosphate/Deoxyadenosine 5'-triphosphate/Deoxyadenosine triphosphate	C10H16N5O12P3
db4p	3,4-dihydroxy-2-butanone 4-phosphate		C4H9O6P
dc	Deoxycytidine	2'-Deoxycytidine	C9H13N3O4
dcdp	dCDP	2'-Deoxycytidine diphosphate/2'-Deoxycytidine 5'-diphosphate	C9H15N3O10P2
dchrocmo	2,5-Dichloro-carboxymethylenebut-2-en-4-olide		C6H2Cl2O4
dchrooe	2,5-Dichloro-4-oxohex-2-enedioate		C6H4Cl2O5
dcmp	dCMP	Deoxycytidylic acid/Deoxycytidine monophosphate/Deoxycytidylate/2'-Deoxycytidine 5'-monophosphate	C9H14N3O7P
dctp	dCTP	Deoxycytidine 5'-triphosphate/Deoxycytidine triphosphate/2'-Deoxycytidine 5'-triphosphate	C9H16N3O13P3
dcys	D-Cysteine	D-Amino-3-mercaptopropionic acid	C3H7NO2S
dg	Deoxyguanosine	2'-Deoxyguanosine	C10H13N5O4
dgadmh17bp	D-glycero-alpha-D-manno-Heptose 1,7-bisphosphate		C7H16O13P2

dgadmh1p	D-glycero-alpha-D-manno-Heptose 1-phosphate		C7H15O10P
dgadmh7p	D-glycero-alpha-D-manno-Heptose 7-phosphate		C7H15O10P
dgal	D-Galactarate	D-Mucic acid/D-Galactaric acid	C6H10O8
dgalac	D-Galacturonate	D-Galacturonic acid	C6H10O7
dgalcer	Digalactosylceramide	Gal-alpha1->4Gal-beta1->1'Cer	C31H56NO13R
dgalctn	D-Galactonate	D-Galactonic acid	C6H12O7
dgaldg	Digalactosyl-diacylglycerol	DGDG/Gal-alpha1->6Gal-beta1->3acyl2Gro/Galalpha1->6Galbeta1->3acyl2Gro/1,2-Diacyl-3-O-(alpha-D-galactosyl(1->6)-O-beta-D-galactosyl)-sn-glycerol/3-[alpha-D-Galactosyl-(1->6)-beta-D-galactosyl]-1,2-diacyl-sn-glycerol/2,3-Di-O-acyl-1-O-(6-O-alpha-D-galactosyl-beta-D-galactosyl)-D-glycerol	C17H26O15R2
dgdg	Diglucosyl-diacylglycerol	Glc-beta1->6Glc-beta1->3acyl2Gro/Glcbeta1->6Glcbeta1->3acyl2Gro/1,2-Diacyl-3-O-(beta-D-glucopyranosyl(1->6)-O-beta-D-glucopyranosyl)-sn-glycerol	C17H26O15R2
dgdmh17bp	D-Glycero-D-manno-heptose 1,7-bisphosphate		C7H16O13P2
dgdmh1p	D-Glycero-D-manno-heptose 1-phosphate		C7H15O10P
dgdmh7p	D-Glycero-D-manno-heptose 7-phosphate	D-glycero-beta-D-manno-Heptose 7-phosphate	C7H15O10P
dgdp	dGDP	2'-Deoxyguanosine 5'-diphosphate	C10H15N5O10P2
dgln	D-Glutamine	D-2-Aminoglutaramic acid	C5H10N2O3
dglu	D-Glutamate	D-Glutamic acid/D-Glutaminic acid/D-2-Aminoglutaric acid	C5H9NO4
dgluc	D-Glucuronate	Glucuronic acid/Glucuronate	C6H10O7
dgluca	D-Glucarate	D-Glucaric acid/L-Gularic acid/d-Saccharic acid/D-Glucosaccharic acid	C6H10O8
dglucl	D-Glucuronolactone	Glucurone/Glucofuranurono-6,3-lactone/D-Glucurono-3,6-lactone/D-Glucurone	C6H8O6
dgmp	dGMP	2'-Deoxyguanosine 5'-monophosphate/2'-Deoxyguanosine 5'-phosphate/Deoxyguanylic acid/Deoxyguanosine monophosphate	C10H14N5O7P
dgr	1,2-Diacyl-sn-glycerol	1,2-Diacylglycerol/D-1,2-Diacylglycerol	C5H6O5R2
dgtp	dGTP	2'-Deoxyguanosine 5'-triphosphate/Deoxyguanosine 5'-triphosphate/Deoxyguanosine triphosphate	C10H16N5O13P3
dhap	Glycerone phosphate	Dihydroxyacetone phosphate	C3H7O6P
dhcinnm	trans-2,3-Dihydroxycinnamate		C9H8O4
dhdp	2,3-Dihydrodipicolinate	L-2,3-Dihydrodipicolinate/Dihydrodipicolinic acid/Dihydrodipicolinate	C7H7NO4
dhf	Dihydrofolate	Dihydrofolic acid/7,8-Dihydrofolate/7,8-Dihydrofolic acid/7,8-Dihydropteroylglutamate	C19H21N7O6
dhmp	(R)-2,3-Dihydroxy-3-methylpentanoate	(R)-2,3-Dihydroxy-3-methylvalerate/(2R,3R)-2,3-Dihydroxy-3-methylpentanoate	C6H12O4
dhmva	(R)-2,3-Dihydroxy-3-methylbutanoate	(R)-2,3-Dihydroxy-isovalerate/(R)-2,3-Dihydroxy-isovaleric acid/(2R)-2,3-Dihydroxy-3-methylbutanoate	C5H10O4
dhn	1,4-Dihydroxy-2-naphthoate		C11H8O4
dhnpt	Dihydroneopterin	2-Amino-4-hydroxy-6-(D-erythro-1,2,3-trihydroxypropyl)-7,8-dihydropteridine	C9H13N5O4
dhpant	2-Dehydropantoate		C6H10O4
dhpppn	3-(2,3-Dihydroxyphenyl)propanoate	2,3-Dihydroxyphenylpropanoate	C9H10O4
dhpt	Dihydropteroate	7,8-Dihydropteroate	C14H14N6O3
dhsk	3-Dehydroshikimate		C7H8O5
dhtolen	2,3-Dihydroxytoluene	3-Methylcatechol	C7H8O2
diacetyl	Diacetyl	Biacetyl/Dimethylglyoxal/2,3-Butanedione	C4H6O2
didp	dIDP	2'-Deoxyinosine-5'-diphosphate/2'-Deoxyinosine 5'-diphosphate	C10H14N4O10P2
dikmtpenp	2,3-Diketo-5-methylthiopentyl-1-phosphate	5-(Methylthio)-2,3-dioxopentyl phosphate	C6H11O6PS
dimgp	D-erythro-1-(Imidazol-4-yl)glycerol 3-phosphate	D-erythro-Imidazole-glycerol 3-phosphate/D-erythro-Imidazole-glycerol phosphate	C6H11N2O6P
dimp	dIMP	2'-Deoxyinosine 5'-phosphate	C10H13N4O7P
din	Deoxyinosine		C10H12N4O4
ditp	dITP	2'-Deoxyinosine-5'-triphosphate/2'-Deoxyinosine 5'-triphosphate	C10H15N4O13P3
dlald	D-Lactaldehyde	(R)-Lactaldehyde/D-2-Hydroxypropionaldehyde	C3H6O2
dlipo	Dihydrolipoamide-E	Enzyme N6-(dihydrolipoyl)lysine	C8H16NOS2R
dlipoe	Dihydrolipoamide	Dihydrothioctamide	C8H17NOS2
dlipop	Dihydrolipoylprotein	[Protein]-dihydrolipoyllysine	C8H16NOS2R
dmal	D-Malate	(R)-Malate/D-Malic acid	C4H6O5
dmbzid	5,6-Dimethylbenzimidazole	Dimethylbenzimidazole	C9H10N2
dmet	D-Methionine	D-2-Amino-4-(methylthio)butyric acid	C5H11NO2S

dmi1p	Inositol 1-phosphate	myo-Inositol 1-phosphate/1D-myo-Inositol 1-phosphate/D-myo-Inositol 1-phosphate/1D-myo-Inositol 1-monophosphate	C6H13O9P
dmi3p	1D-myo-Inositol 3-phosphate	D-myo-Inositol 3-phosphate/myo-Inositol 3-phosphate/Inositol 3-phosphate/1D-myo-Inositol 3-monophosphate/D-myo-Inositol 3-monophosphate/myo-Inositol 3-monophosphate/Inositol 3-monophosphate/1L-myo-Inositol 1-phosphate/L-myo-Inositol 1-phosphate	C6H13O9P
dmi4p	myo-Inositol 4-phosphate	D-myo-Inositol 4-phosphate/1D-myo-Inositol 4-phosphate/1D-myo-Inositol 4-monophosphate/Inositol 4-phosphate	C6H13O9P
dmlz	6,7-Dimethyl-8-(1-D-ribityl)lumazine		C13H18N4O6
dmpp	Dimethylallyl diphosphate	Prenyl diphosphate/2-Isopentenyl diphosphate/delta2-Isopentenyl diphosphate/delta-Prenyl diphosphate/DMAPP	C5H12O7P2
dmppth	Demethylphosphinothricin	(2S)-2-Amino-4-(hydroxyphosphinyl)butanoic acid	C4H10NO4P
dms	Dimethyl sulfide	Methyl sulfide/Methyl thioether	C2H6S
dmso	Dimethyl sulfoxide	DMSO	C2H6OS
DNA	DNA		
dna5mcys	DNA 5-methylcytosine	DNA containing 5-methylcytosine/5-Methylcytosine (in DNA)	C10H16N3O7P(C5H8O5PR)n(C5H8O5PR)n
dnacys	DNA cytosine	Cytosine (in DNA)	C9H14N3O7P(C5H8O5PR)n(C5H8O5PR)n
dnad	Deamino-NAD+	Deamido-NAD+/Deamido-NAD	C21H27N6O15P2
dopa	Dopamine	4-(2-Aminoethyl)-1,2-benzenediol/4-(2-Aminoethyl)benzene-1,2-diol/3,4-Dihydroxyphenethylamine/2-(3,4-Dihydroxyphenyl)ethylamine	C8H11NO2
doroa	(S)-Dihydroorotate	(S)-4,5-Dihydroorotate/L-Dihydroorotate/L-Dihydroorotic acid/Dihydro-L-orotic acid	C5H6N2O4
doxrib	Deoxyribose	2-Deoxy-D-erythro-pentose/Thyminose/2-Deoxy-D-ribose	C5H10O4
dpcoa	Dephospho-CoA		C21H35N7O13P2S
dphe	D-Phenylalanine	D-alpha-Amino-beta-phenylpropionic acid	C9H11NO2
dqt	3-Dehydroquinate	5-Dehydroquinate/3-Dehydroquinic acid/5-Dehydroquinic acid	C7H10O6
dr1p	2-Deoxy-D-ribose 1-phosphate	2-Deoxy-alpha-D-ribose 1-phosphate	C5H11O7P
drib	D-Ribulose	D-erythro-2-Pentulose/D-Arabinoketose/D-Arabinulose/D-Riboketose	C5H10O5
dscl	dihydrosirohydrochlorin	Precorrin 2	C42H48N4O16
dser	D-Serine		C3H7NO3
dtb	Dethiobiotin	Desthiobiotin	C10H18N2O3
dtdp	dTDP	Deoxythymidine 5'-diphosphate	C10H16N2O11P2
dtdp4d6dg	dTDP-4-dehydro-6-deoxy-alpha-D-glucose	dTDP-4-oxo-6-deoxy-alpha-D-glucose	C16H24N2O15P2
dtdp4d6dm	dTDP-4-dehydro-6-deoxy-L-mannose	dTDP-4-oxo-6-deoxy-L-mannose/dTDP-4-oxo-L-rhamnose	C16H24N2O15P2
dtdpgal	dTDP-galactose	dTDP-D-galactose	C16H26N2O16P2
dtdpglu	dTDP-glucose	dTDP-D-glucose	C16H26N2O16P2
dtdprmn	dTDP-L-rhamnose	dTDP-6-deoxy-L-mannose	C16H26N2O15P2
dtmp	dTMP	Thymidine 5'-phosphate/Deoxythymidine 5'-phosphate/Thymidylic acid/5'-Thymidylic acid/Thymidine monophosphate/Deoxythymidylic acid/Thymidylate	C10H15N2O8P
dttp	dTTP	Deoxythymidine triphosphate/Deoxythymidine 5'-triphosphate/TTP	C10H17N2O14P3
du	Deoxyuridine	2-Deoxyuridine/2'-Deoxyuridine	C9H12N2O5
dudp	dUDP	2'-Deoxyuridine 5'-diphosphate	C9H14N2O11P2
dump	dUMP	Deoxyuridylic acid/Deoxyuridine monophosphate/Deoxyuridine 5'-phosphate/2'-Deoxyuridine 5'-phosphate	C9H13N2O8P
dutp	dUTP	2'-Deoxyuridine 5'-triphosphate	C9H15N2O14P3
dx5p	1-Deoxy-D-xylulose 5-phosphate		C5H11O7P
e3mcpen	(E)-3-(Methoxycarbonyl)pent-2-enedioate		C7H8O6
e3mm	D-erythro-3-Methylmalate		C5H8O5
e4hglu	L-erythro-4-Hydroxyglutamate		C5H9NO5
e4p	D-Erythrose 4-phosphate		C4H9O7P
eglycol	Ethylene glycol	1,2-Ethanediol/Ethane-1,2-diol	C2H6O2
epimelib	Epimelibiose	6-O-(alpha-D-Galactopyranosyl)-D-mannopyranose	C12H22O11
er4p	4-Phospho-D-erythronate	4-Phosphoerythronate	C4H9O8P
eth	Ethanol	Ethyl alcohol/Methylcarbinol/Dehydrated ethanol	C2H6O
etha	Ethanolamine	Aminoethanol/2-Hydroxyethylamine	C2H7NO
ethnit	Ethylnitronate		C2H4NO2
f1p	D-Fructose 1-phosphate		C6H13O9P
f6p	D-Fructose 6-phosphate	D-Fructose 6-phosphoric acid/Neuberg ester	C6H13O9P

fa	Formamide	Methanamide	CH3NO
fad	FAD	Flavin adenine dinucleotide	C27H33N9O15P2
fadh2	FADH2		C27H35N9O15P2
fdp	D-Fructose 1,6-bisphosphate		C6H14O12P2
fe2	Fe2+	Fe(II)/Ferrous ion/Iron(2+)	Fe
fe3	Fe3+	Fe(III)/Ferric ion/Iron(3+)	Fe
fgam	N2-Formyl-N1-(5-phospho-D-ribosyl)glycinamide	5'-Phosphoribosyl-N-formylglycinamide/N-Formyl-GAR/N-Formylglycinamide ribonucleotide	C8H15N2O9P
fl	Folate	Pteroylglutamic acid/Folic acid	C19H19N7O6
fmettrna	N-Formylmethionyl-tRNA		C16H26NO12PSR2(C5H8O6PR)n
fmgt	S-Formylglutathione		C11H17N3O7S
fmn	FMN	Riboflavin-5-phosphate/Flavin mononucleotide	C17H21N4O9P
fmnh2	Reduced FMN	FMNH2	C17H23N4O9P
formald	Formaldehyde	Methanal/Oxomethane/Oxomethylene/Methylene oxide/Formalin	CH2O
formate	Formate	Methanoic acid/Formic acid	CH2O2
fpram	2-(Formamido)-N1-(5-phospho-D-ribosyl)acetamidine	2-(Formamido)-N1-(5'-phosphoribosyl)acetamidine/1-(5'-Phosphoribosyl)-N-formylglycinamidine/5'-Phosphoribosyl-N-formylglycinamidine/5'-Phosphoribosylformylglycinamidine	C8H16N3O8P
fprica	5-Formamido-1-(5-phospho-D-ribosyl)imidazole-4-carboxamide	1-(5'-Phosphoribosyl)-5-formamido-4-imidazolecarboxamide/5'-Phosphoribosyl-5-formamido-4-imidazolecarboxamide/5-Formamido-1-(5-phosphoribosyl)imidazole-4-carboxamide	C10H15N4O9P
frdp	Farnesyl diphosphate	trans,trans-Farnesyl diphosphate/Farnesyl pyrophosphate/2-trans,6-trans-Farnesyl diphosphate	C15H28O7P2
fru	D-Fructose	Levulose/Fruit sugar/D-arabino-Hexulose	C6H12O6
frutn	D-Fructuronate	D-Fructuronic acid	C6H10O7
fthf	10-Formyltetrahydrofolate	10-Formyl-THF	C20H23N7O7
fuc	L-Fucose	6-Deoxy-L-galactose	C6H12O5
fuc1p	L-Fucose 1-phosphate	6-Deoxy-L-galactose 1-phosphate/beta-L-Fucose 1-phosphate	C6H13O8P
fucul	L-Fuculose	6-Deoxy-L-tagatose	C6H12O5
fucul1p	L-Fuculose 1-phosphate		C6H13O8P
fum	Fumarate	Fumaric acid/trans-Butenedioic acid	C4H4O4
fumpyr	3-Fumarylpyruvate		C7H6O6
g15l	D-Glucono-1,5-lactone	Gluconic lactone/Gluconic acid lactone/1,5-Gluconolactone/delta-Gluconolactone/D-Gluconolactone/Gluconolactone/D-Aldonolactone	C6H10O6
g1p	D-Glucose 1-phosphate	alpha-D-Glucose 1-phosphate/Cori ester/D-Glucose alpha-1-phosphate	C6H13O9P
g3p	D-Glyceraldehyde 3-phosphate	(2R)-2-Hydroxy-3-(phosphonooxy)-propanal	C3H7O6P
g3pc	sn-Glycero-3-phosphocholine	Glycerophosphocholine	C8H21NO6P
g3pe	sn-Glycero-3-phosphoethanolamine	Glycerophosphoethanolamine	C5H14NO6P
g3pg	Glycerophosphoglycerol		C6H15O8P
g3pi	sn-Glycero-3-phospho-1-inositol	1-(sn-glycero-3-Phospho)-1D-myo-inositol	C9H19O11P
g3ps	Glycerophosphoserine		C6H13NO8P
g6p	D-Glucose 6-phosphate	Glucose 6-phosphate/Robison ester	C6H13O9P
ga1p	D-Glucosamine 1-phosphate	alpha-D-Glucosamine 1-phosphate	C6H14NO8P
ga6p	D-Glucosamine 6-phosphate	D-Glucosamine phosphate	C6H14NO8P
gaba	4-Aminobutanoate	4-Aminobutanoic acid/4-Aminobutyrate/4-Aminobutyric acid/gamma-Aminobutyric acid/4-Aminobutylate	C4H9NO2
galactan	Galactan		(C12H20O11)n
galactan_1	Galactan-Galactose		(C12H20O11)n - (C6H12O6)
galactinol	alpha-D-Galactosyl-(1->3)-1D-myo-inositol	O-alpha-D-Galactosyl-(1->3)-1D-myo-inositol/3-O-alpha-D-Galactosyl-1D-myo-inositol/1-alpha-D-Galactosyl-myo-inositol/Galactinol	C12H22O11
galcer	Galactosylceramide	Galactocerebroside/D-Galactosyl-N-acylsphingosine/Cerebroside/D-Galactosylceramide	C25H46NO8R
gallate	Gallate	Gallic acid/3,4,5-Trihydroxybenzoic acid/3,4,5-Trihydroxybenzoate/Pyrogallol-5-carboxylic acid	C7H6O5
galt1p	Galactitol 1-phosphate	D-Galactitol 1-phosphate/L-Galactitol 6-phosphate	C6H15O9P
gar	5'-Phosphoribosylglycinamide	GAR/N1-(5-Phospho-D-ribosyl)glycinamide/Glycinamide ribonucleotide	C7H15N2O8P
gbbtn	gamma-butyrobetaine		C7H15NO2
gcarmclc	gamma-Carboxymuconolactone	5-Carboxy-2,5-dihydro-2-oxofuran-5-acetate/4-Carboxymuconolactone/2-Carboxy-2,5-dihydro-5-oxofuran-2-acetate	C7H6O6
gcys	gamma-L-Glutamyl-L-cysteine	L-gamma-Glutamylcysteine/5-L-Glutamyl-L-cysteine/gamma-Glutamylcysteine	C8H14N2O5S

gdp	GDP	Guanosine 5'-diphosphate/Guanosine diphosphate	C10H15N5O11P2
gensa	2,5-Dihydroxybenzoate	Gentisic acid/Hydroquinonecarboxylic acid/Gentisate	C7H6O4
ggbap	gamma-Glutamyl-beta-aminopropiononitrile	gamma-Glutamyl-3-aminopropiononitrile	C8H13N3O3
ggbcya	gamma-Glutamyl-beta-cyanoalanine		C9H13N3O5
gl	Glycerol	Glycerin/1,2,3-Trihydroxypropane/1,2,3-Propanetriol	C3H8O3
glac	D-Galactose		C6H12O6
glac1p	alpha-D-Galactose 1-phosphate	alpha-D-Galactopyranose 1-phosphate	C6H13O9P
glacgly	Galactosylglycerol	3-beta-D-Galactosyl-sn-glycerol	C9H18O8
glal	Glycolaldehyde	Hydroxyacetaldehyde	C2H4O2
glc	D-Glucose	Grape sugar/Dextrose	C6H12O6
gln	L-Glutamine	L-2-Aminoglutaramic acid	C5H10N2O3
glntrna	Glutaminyl-tRNA	L-Glutaminyl-tRNA(Gln)/Glutaminyl-tRNA(Gln)/Gln-tRNA(Gln)	C20H29N7O12PR(C5H8O6PR)n
glu	L-Glutamate	L-Glutamic acid/L-Glutaminic acid	C5H9NO4
glu1sa	L-Glutamate 1-semialdehyde	(S)-4-Amino-5-oxopentanoate	C5H9NO3
glu5p	L-Glutamate 5-phosphate	L-Glutamyl 5-phosphate	C5H10NO7P
gluamibut	gamma-Glutamyl-gamma-aminobutyraldehyde		C9H16N2O4
gluamibutr	gamma-Glutamyl-gamma-aminobutyrate	4-(Glutamylamino)butanoate/gamma-Glutamyl-GABA	C9H16N2O5
gluc	D-Gluconate	D-Gluconic acid/D-gluco-Hexonic acid	C6H12O7
glucer	Glucosylceramide	Glucocerebroside/D-Glucosyl-N-acylsphingosine	C25H46NO8R
glugsal	L-Glutamate 5-semialdehyde	L-Glutamate gamma-semialdehyde	C5H9NO3
glupept	(5-L-Glutamyl)-peptide		C7H11N2O5R(C2H2NOR)n
gluptrc	gamma-L-Glutamylputrescine		C9H19N3O3
glusd	D-Glucoside		C6H11O6R
glutaur	5-L-Glutamyl-taurine	5-Glutamyl-taurine/Glutaurine	C7H14N2O6S
glutcoa	Glutaryl-CoA		C26H42N7O19P3S
gluthsel	Glutathioselenol	GSSeH	C10H17N3O6SSe
glutrna	L-Glutamyl-tRNA(Glu)		C20H28N6O13PR(C5H8O6PR)n
glx	Glyoxylate	Glyoxalate/Glyoxylic acid	C2H2O3
gly	Glycine	Aminoacetic acid/Gly	C2H5NO2
glyb	Glycine betaine	Betaine/Trimethylaminoacetate/N,N,N-Trimethylglycine/Trimethylammonioacetate	C5H11NO2
glyc3p	Glycerol 3-phosphate	sn-Glycerol 3-phosphate/Glycerophosphoric acid/sn-Gro-1-P	C3H9O6P
glycogen	Glycogen		C24H42O21
glycolate	Glycolate	Glycolic acid/Hydroxyacetic acid	C2H4O3
glyc-R	(R)-Glycerate	D-Glycerate/Glycerate/Glyceric acid	C3H6O4
glyn	Glycerone	Dihydroxyacetone/1,3-Dihydroxyacetone/1,3-Dihydroxy-2-propanone/1,3-Dihydroxypropan-2-one	C3H6O3
glypgg	Glycerophosphoglycoglycerolipid	(sn-Gro-1-P)->6Glc-beta1->6Glc-alpha1->3acyl2Gro/(sn-Gro-1-P)->6Glcbeta1->6Glcalpha1->3acyl2Gro	C20H33O20PR2
glytrna	Glycyl-tRNA(Gly)		C12H20NO11PR2(C5H8O6PR)n
gmp	GMP	Guanosine 5'-phosphate/Guanosine monophosphate/Guanosine 5'-monophosphate/Guanylic acid	C10H14N5O8P
gn	Guanine	2-Amino-6-hydroxypurine	C5H5N5O
gpp	Geranyl diphosphate		C10H20O7P2
gsn	Guanosine		C10H13N5O5
gtp	GTP	Guanosine 5'-triphosphate	C10H16N5O14P3
gtspmd	Glutathionylspermidine	N1-(gamma-L-Glutamyl-L-cysteinyl-glycyl)-spermidine	C17H34N6O5S
guadbut	4-Guanidinobutanamide		C5H12N4O
guadbutn	4-Guanidinobutanoate		C5H11N3O2
h	H+		H
h2	H2		H2
h2co3	Carbonic acid	Dihydrogen carbonate/ H2CO3	H2CO3
h2o	H2O	Water	H2O
h2o2	H2O2	Hydrogen peroxide/Oxydol	H2O2
h2s	Hydrogen sulfide	Hydrogen-sulfide/H2S	H2S
h3op	2-Hydroxy-3-oxopropanoate	Tartronate semialdehyde	C3H4O4
hadpcoa	(3S)-3-Hydroxyadipyl-CoA		C27H44N7O20P3S
hchdn	2-Hydroxy-cis-hex-2,4-dienoate		C6H8O3
hcit	Homocitrate	(R)-2-Hydroxybutane-1,2,4-tricarboxylate/Homocitric acid/3-Hydroxy-3-carboxyadipic acid/(R)-2-Hydroxy-1,2,4-butanetricarboxylic acid	C7H10O7
hco3	Bicarbonate	HCO3-/Hydrogencarbonate/Acid carbonate	HCO3
hcys	L-Homocysteine	L-2-Amino-4-mercaptobutyric acid	C4H9NO2S

hemeO	Heme O		C49H58FeN4O5
hext	extracellular hydrogen		H
hf	Hydrofluoric acid		HF
hgenbyr	Hydrogenobyrinate	Hydrogenobyrinic acid	C45H60N4O14
hgenbytdi	Hydrogenobyrinate a,c diamide	Hydrogenobyrinate diamide/Hydrogenobyrinic acid a,c-diamide	C45H62N6O12
hibut	(S)-3-Hydroxyisobutyrate		C4H8O3
his	L-Histidine	(S)-alpha-Amino-1H-imidazole-4-propionic acid	C6H9N3O2
hisol	L-Histidinol		C6H11N3O
hisolp	L-Histidinol phosphate		C6H12N3O4P
histrna	L-Histidyl-tRNA(His)		C16H24N3O11PR2(C5H8O6PR)n
hmb	Hydroxymethylbilane		C40H46N4O17
hmb4pp	1-Hydroxy-2-methyl-2-butenyl 4-diphosphate	(E)-4-Hydroxy-3-methylbut-2-en-1-yl diphosphate	C5H12O8P2
hmcmct	2-Hydroxy-5-methyl-cis,cis-muconate		C7H8O5
hmgth	S-(Hydroxymethyl)glutathione		C11H19N3O7S
hmnapth	1-Hydroxymethylnaphthalene	1-Naphthalenemethanol	C11H10O
hnaptho	1-Hydroxy-2-naphthoate	1-Hydroxy-2-naphthoic acid/1-Naphthol-2-carboxylic acid	C11H8O3
hohx	4-Hydroxy-2-oxohexanoic acid	4-Hydroxy-2-oxohexanoate	C6H10O4
holoCAB	Holo-[carboxylase]	Biotin-carboxyl-carrier protein	C17H27N5O4SR2
homogen	Homogentisate	Homogentisic acid/2,5-Dihydroxyphenylacetic acid/2,5-Dihydroxyphenylacetate	C8H8O4
hopt	4-Hydroxy-2-oxopentanoate	4-Hydroxy-2-oxovalerate	C5H8O4
hpheac	3-Hydroxyphenylacetate		C8H8O3
hppr	Hippurate	Hippuric acid/N-Benzoylglycine/Benzoylaminoacetic acid	C9H9NO3
hpyr	Hydroxypyruvate	Hydroxypyruvic acid/3-Hydroxypyruvate/3-Hydroxypyruvic acid	C3H4O4
hqn	p-Benzenediol	Hydroquinone/1,4-Benzenediol/1,4-Dihydroxybenzene/Benzene-1,4-diol/Quinol/4-Hydroxyphenol	C6H6O2
hser	L-Homoserine	2-Amino-4-hydroxybutyric acid	C4H9NO3
hydroxyakg	D-4-Hydroxy-2-oxoglutarate		C5H6O6
hyxn	Hypoxanthine	Purine-6-ol	C5H4N4O
i3aa	Indole-3-acetaldehyde	2-(Indol-3-yl)acetaldehyde/Indoleacetaldehyde	C10H9NO
i3ac	Indole-3-acetate	Indole-3-acetic acid/(Indol-3-yl)acetate/Indoleacetate/Indoleacetic acid	C10H9NO2
i4aa	Imidazole-4-acetaldehyde	Imidazole acetaldehyde	C5H6N2O
i4ac	Imidazole-4-acetate	Imidazoleacetic acid/4-Imidazoleacetate	C5H6N2O2
iasp	Iminoaspartate		C4H5NO4
icit	Isocitrate	Isocitric acid/1-Hydroxytricarballylic acid/1-Hydroxypropane-1,2,3-tricarboxylic acid	C6H8O7
id3act	Indole-3-acetamide		C10H10N2O
idactn	3-Indoleacetonitrile	Indol-3-ylacetonitrile/Indole-3-acetonitrile/(Indol-3-yl)acetonitrile	C10H8N2
idon	L-Idonate		C6H12O7
idp	IDP	Inosine 5'-diphosphate/Inosine diphosphate	C10H14N4O11P2
idpyr	Indolepyruvate	Indolepyruvic acid/(Indol-3-yl)pyruvate/Indole-3-pyruvate/3-(Indol-3-yl)pyruvate	C11H9NO3
ile	L-Isoleucine	2-Amino-3-methylvaleric acid	C6H13NO2
iletrna	L-Isoleucyl-tRNA(Ile)		C21H32N6O11PR(C5H8O6PR)n
imACP	3-(Imidazol-4-yl)-2-oxopropyl phosphate	Imidazole-acetol phosphate	C6H9N2O5P
imal	Isomaltose	Brachiose	C12H22O11
imp	IMP	Inosinic acid/Inosine monophosphate/Inosine 5'-monophosphate/Inosine 5'-phosphate/5'-Inosinate/5'-Inosinic acid/5'-Inosine /onophosphate/5'-IMP	C10H13N4O8P
in12356pkp	Inositol 1,2,3,5,6-pentakisphosphate	myo-Inositol 1,2,3,5,6-pentakisphosphate/1D-myo-Inositol 1,2,3,5,6-pentakisphosphate/1L-myo-Inositol 1,2,3,4,5-pentakisphosphate	C6H17O21P5
indole	Indole	2,3-Benzopyrrole	C8H7N
ins	Inosine		C10H12N4O5
ipp	Isopentenyl diphosphate	delta3-Isopentenyl diphosphate/delta3-Methyl-3-butenyl diphosphate	C5H12O7P2
isochor	Isochorismate	Isochorismic acid	C10H10O6
itcn	Itaconate	Itaconic acid/Methylenesuccinic acid	C5H6O4
itcncoa	Itaconyl-CoA		C26H40N7O19P3S
itp	ITP	Inosine 5'-triphosphate/Inosine triphosphate/Inosine tripolyphosphate	C10H15N4O14P3
k	potassium	K+	K
k2lipiv	Di[3-deoxy-D-manno-octulosonyl]-lipid IV(A)	KDO2-lipid IV(A)	C84H154N2O37P2
kdg	2-Dehydro-3-deoxy-D-gluconate		C6H10O6
kdo	3-Deoxy-D-manno-octulosonate	KDO/2-Dehydro-3-deoxy-D-octonate/3-Deoxy-D-manno-2-octulosonate/3-Deoxyoctulosonic acid	C8H14O8

kdo8p	3-Deoxy-D-manno-octulosonate 8-phosphate	2-Dehydro-3-deoxy-D-octonate 8-phosphate	C8H15O11P
kdolipid4	3-Deoxy-D-manno-octulosonyl-lipid IV(A)	KDO-lipid IV(A)/3-Deoxy-D-manno-octulosonyl-2',3',2',3'-tetrakis(beta-hydroxymyristoyl)-D-glucosaminyl-1,6-beta-D-glucosamine 1,4'-bisphosphate	C76H142N2O30P2
kdpg	2-Dehydro-3-deoxy-6-phospho-D-gluconate	6-Phospho-2-dehydro-3-deoxy-D-gluconate/2-Keto-3-deoxy-6-phosphogluconate/2-Dehydro-3-deoxy-D-gluconate 6-phosphate	C6H11O9P
knt	Kynurenate	4-Hydroxy-2-quinolinecarboxylic acid/Kynurenic acid	C10H7NO3
l1p3h5c	L-1-Pyrroline-3-hydroxy-5-carboxylate	3-Hydroxy-L-1-pyrroline-5-carboxylate	C5H7NO3
lac	D-Lactate	(R)-Lactate/D-Lactic acid/D-2-Hydroxypropanoic acid/D-2-Hydroxypropionic acid	C3H6O3
lactcoa	Lactoyl-CoA		C24H40N7O18P3S
lactose	Lactose	1-beta-D-Galactopyranosyl-4-alpha-D-glucopyranose/Milk sugar/alpha-Lactose/Anhydrous lactose	C12H22O11
larabinose	L-Arabinose	L-Arabinopyranose	C5H10O5
leu	L-Leucine	2-Amino-4-methylvaleric acid/(2S)-alpha-2-Amino-4-methylvaleric acid/(2S)-alpha-Leucine	C6H13NO2
leutrna	L-Leucyl-tRNA(Leu)	L-Leucyl-tRNA	C21H32N6O11PR(C5H8O6PR)n
levan_m	Levan(m)	(2,6-beta-D-Fructosyl)n; Levan n/2,6-beta-D-Fructan/(2,6-beta-D-Fructosyl)n+1	C12H22O11(C6H10O5)n
levan_mn	Levan(m+n)	(2,6-beta-D-Fructosyl)n; Levan n/2,6-beta-D-Fructan/(2,6-beta-D-Fructosyl)n+1	C12H22O11(C6H10O5)n
levan_n	Levan(n)	(2,6-beta-D-Fructosyl)n; Levan n/2,6-beta-D-Fructan/(2,6-beta-D-Fructosyl)n+1	C12H22O11(C6H10O5)n
lipa	Di[3-deoxy-D-manno-octulosonyl]-lipid A	KDO2-lipid (A)	C110H202N2O39P2
lipidA	2,3,2'3'-Tetrakis(3-hydroxytetradecanoyl)-D-glucosaminyl-1,6-beta-D-glucosamine 1,4'-bisphosphate	2,3,2'3'-Tetrakis(beta-hydroxymyristoyl)-D-glucosaminyl-1,6-beta-D-glucosamine 1,4'-bisphosphate/Lipid A disaccharide bisphosphate/Lipid IV(A)	C68H130N2O23P2
lipidAds	2,3,2',3'-Tetrakis(3-hydroxytetradecanoyl)-D-glucosaminyl-1,6-beta-D-glucosamine 1-phosphate	2,3-Bis(3-hydroxytetradecanoyl)-D-glucosaminyl-1,6-beta-D-2,3-bis(3-hydroxytetradecanoyl)-beta-D-glucosaminyl 1-phosphate/2,3-Bis-(beta-hydroxymyristoyl)-D-glucosaminyl-(beta-D-1,6)-2,3-bis(beta-hydroxymyristoyl)-D-glucosaminyl beta-phosphate/2,3-Bis-(3-hydroxytetradecanoyl)-D-glucosaminyl-(beta-D-1,6)-2,3-bis(3-hydroxy-tetradecanoyl)-D-glucosaminyl beta-phosphate/Lipid A disaccharide	C68H129N2O20P
lipidX	2,3-Bis(3-hydroxytetradecanoyl)-beta-D-glucosaminyl 1-phosphate	2,3-Bis(beta-hydoroxymyristoyl)-beta-D-glucosaminyl 1-phosphate/Lipid X	C34H66NO12P
lipo	Lipoamide-E	Enzyme N6-(lipoyl)lysine	C8H14NOS2R
lipoe	Lipoamide	Thioctic acid amide	C8H15NOS2
lipop	Lipoylprotein	H-Protein-lipoyllysine	C8H14NOS2R
lk2lipiv	Lauroyl-KDO2-lipid IV(A)		C96H176N2O38P2
llac	D-Lactate	(S)-Lactate/L-Lactic acid	C3H6O3
llald	L-Lactaldehyde	(S)-Lactaldehyde/L-2-Hydroxypropionaldehyde	C3H6O2
llct	L-Cystathionine		C7H14N2O4S
LPS	lipopolisaccharide		
lrib	L-Ribulose	L-erythro-Pentulose/L-Arabinoketose/L-Arabinulose/L-Riboketose	C5H10O5
lrl5p	L-Ribulose 5-phosphate		C5H11O8P
ltg	(R)-S-Lactoylglutathione		C13H21N3O8S
lxu1p	L-Xylulose 1-phosphate		C5H11O8P
lxu5p	L-Xylulose 5-phosphate		C5H11O8P
lxylu	L-Xylulose	L-threo-Pentulose/L-Lyxulose	C5H10O5
lys	L-Lysine	Lysine acid/2,6-Diaminohexanoic acid	C6H14N2O2
lystrna	L-Lysine-tRNA (Lys)	L-Lysyl-tRNA	C16H29N2O11PR2(C5H8O6PR)n
maco	cis-2-Methylaconitate	Z)-But-2-ene-1,2,3-tricarboxylate	C7H8O6
mal	L-Malate	(S)-Malate/L-Apple acid/L-Malic acid/L-2-Hydroxybutanedioic acid	C4H6O5
malACP	Malonyl-[acyl-carrier protein]		C3H3O3SR
malcoa	Malonyl-CoA	Malonyl coenzyme A	C24H38N7O19P3S
malpyr	Maleylpyruvate	Maleylpyruvic acid/3-Maleylpyruvate	C7H6O6
malthp	Celloheptaose		C42H72O36
maltpt	Cellopentaose		C30H52O26
man	D-Mannose	Mannose/Seminose/Carubinose	C6H12O6
man6p	D-Mannose 6-phosphate		C6H13O9P
mann	D-Mannonate		C6H12O7

mannot	Manninotriose	D-Gal alpha 1->6D-Gal alpha 1->6D-Glucose/D-Gal-alpha1->6D-Gal-alpha1->6D-Glucose	C18H32O16
mclact	(S)-5-Oxo-2,5-dihydrofuran-2-acetate	(+)-Muconolactone	C6H6O4
mde4p	2-C-methyl-D-erythritol 4-phosphate		C5H13O7P
mdecpp	2-C-methyl-D-erythritol 2,4-cyclodiphosphate	3-Methyl-1,2,3,4-tetrahydroxybutane-1,3-cyclic bisphosphate	C5H12O9P2
meli	Melibiose	6-O-(alpha-D-Galactopyranosyl)-D-glucopyranose/D-Gal-alpha1->6D-Glucose	C12H22O11
melibt	Melibiitol	6-O-alpha-D-Galactosyl-D-glucitol	C12H24O11
mesacon	Mesaconate	2-Methylfumarate/Mesaconic acid/Methylfumaric acid/(E)-2-Methyl-2-butenedioic acid	C5H6O4
met	L-Methionine	Methionine/L-2-Amino-4methylthiobutyric acid	C5H11NO2S
methanol	Methanol	Methyl alcohol	CH4O
methf	5,10-Methenyltetrahydrofolate		C20H22N7O6
metseln	Methaneselenol	Methylselenol	CH4Se
metselnt	Methylselenic acid	Methylseleninate	CH4O2Se
metthf	5,10-Methylenetetrahydrofolate	(6R)-5,10-Methylenetetrahydrofolate/5,10-Methylene-THF	C20H23N7O6
mettrna	L-Methionyl-tRNA (Met)	L-Methionyl-tRNA	C20H30N6O11PSR(C5H8O6PR)n
mg2	Magnesium	Mg2+	Mg
mgdg	1,2-Diacyl-3-beta-D-galactosyl-sn-glycerol	D-Galactosyldiacylglycerol/3-beta-D-Galactosyl-1,2-diacylglycerol/Monogalactosyldiglyceride/Monogalactosyl-diacylglycerol/MGDG/Galbeta1->3acyl2Gro/3-(beta-D-Galactosyl)-1,2-diacyl-sn-glycerol	C11H16O10R2
mi	myo-Inositol	D-myo-Inositol/1D-myo-Inositol/L-myo-Inositol/1L-myo-Inositol/meso-Inositol/Inositol/Dambose/Cyclohexitol/Meat sugar/Bios I	C6H12O6
micit	methylisocitrate	(2S,3R)-3-Hydroxybutane-1,2,3-tricarboxylate/Methylisocitric acid	C7H10O7
mk	Menaquinone	Menatetrenone	C16H16O2(C5H8)n
mlt	Maltose	Malt sugar/1-alpha-D-Glucopyranosyl-4-alpha-D-glucopyranose	C12H22O11
mlt6p	Maltose 6'-phosphate		C12H23O14P
mlthx	Maltohexaose		C36H62O31
mlttr	Maltotriose	Amylotriose	C18H32O16
mltttr	Maltotetraose		C24H42O21
mm	Methylmalonate	Methylmalonic acid	C4H6O4
mmcoa-R	(R)-Methylmalonyl-CoA	(R)-2-Methyl-3-oxopropanoyl-CoA/(R)-2-Methyl-3-oxopropionyl-CoA/(R)-3-Oxo-2-methylpropanoyl-CoA	C25H40N7O19P3S
mmcoa-S	(S)-Methylmalonyl-CoA	(S)-2-Methyl-3-oxopropanoyl-CoA/(S)-2-Methyl-3-oxopropionyl-CoA/(S)-Methylmalonyl-coenzyme A/(S)-3-Oxo-2-methylpropanoyl-CoA	C25H40N7O19P3S
mmsa	(S)-Methylmalonate semialdehyde		C4H6O3
mnt1p	D-Mannitol 1-phosphate		C6H15O9P
mobd	Molybdate	Sodium molybdate/Sodium molybdate(VI)	MoO4. 2Na
mpyr	Mercaptopyruvate	3-Mercaptopyruvate	C3H4O3S
mqn	Menaquinol	Reduced menaquinone/Vitamin K2 hydroquinone/Reduced vitamin K2	C16H18O2(C5H8)n
msalc	3-Methylsalicylate		C8H8O3
msalc4	4-Methylsalicylate	m-Cresotic acid/2-Hydroxy-4-methylbenzoic acid	C8H8O3
mslencys	Se-Methyl-L-selenocysteine		C4H9NO2Se
mtartr	meso-Tartaric acid	meso-Tartrate	C4H6O6
mtg	Methylglyoxal	Pyruvaldehyde/Pyruvic aldehyde/2-Ketopropionaldehyde/2-Oxopropanal	C3H4O2
mthf	5-Methyltetrahydrofolate		C20H25N7O6
n1acsprmd	N1-Acetylspermidine		C9H21N3O
n2	nitrogen	N2	N2
n4aab	N4-Acetylaminobutanal		C6H11NO2
n8acsprmd	N8-Acetylspermidine		C9H21N3O
na	Sodium	Na+	Na
nac	Nicotinate	Nicotinic acid/Niacin/3-Pyridinecarboxylic acid	C6H5NO2
nacd	Nicotinate D-ribonucleoside		C11H14NO6
nacn	Nicotinate D-ribonucleotide	beta-Nicotinate D-ribonucleotide/Nicotinate ribonucleotide/Nicotinic acid ribonucleotide	C11H15NO9P
nad	Nicotinamide adenine dinucleotide	NAD+/NAD/DPN/Diphosphopyridine nucleotide/Nadide	C21H28N7O14P2
nadh	Nicotinamide adenine dinucleotide - reduced	NADH/DPNH	C21H29N7O14P2
nadma	N-Acetyl-D-mannosamine	2-Acetamido-2-deoxy-D-mannose	C8H15NO6
nadma6p	N-Acetyl-D-mannosamine 6-phosphate	N-Acetylmannosamine 6-phosphate	C8H16NO9P
nadp	Nicotinamide adenine dinucleotide phosphate	NADP+/NADP/beta-Nicotinamide adenine dinucleotide phosphate/TPN/Triphosphopyridine nucleotide	C21H29N7O17P3

nadph	Nicotinamide adenine dinucleotide phosphate - reduced	NADPH/TPNH	C21H30N7O17P3
nadpt	N-Acetyldemethylphosphinothricin	N-Adpt	C6H12NO5P
naga	N-Acetyl-D-glucosamine	N-Acetylchitosamine/2-Acetamido-2-deoxy-D-glucose/GlcNAc	C8H15NO6
naga1p	N-Acetyl-D-glucosamine 1-phosphate		C8H16NO9P
naga6p	N-Acetyl-D-glucosamine 6-phosphate		C8H16NO9P
naglu	N-Acetyl-L-glutamate	N-Acetyl-L-glutamic acid	C7H11NO5
naglus	N-Acetyl-L-glutamate 5-semialdehyde	2-Acetamido-5-oxopentanoate	C7H11NO4
nam	Nicotinamide	Nicotinic acid amide/Niacinamide/Vitamin PP	C6H6N2O
namd	N-Ribosylnicotinamide	1-(beta-D-Ribofuranosyl)nicotinamide/Nicotinamide-beta-riboside	C11H15N2O5
namn	Nicotinamide D-ribonucleotide	NMN/Nicotinamide mononucleotide/Nicotinamide ribonucleotide/Nicotinamide nucleotide/beta-Nicotinamide D-ribonucleotide/beta-Nicotinamide ribonucleotide/beta-Nicotinamide mononucleotide	C11H15N2O8P
namur6p	N-Acetylmuramic acid 6-phosphate	N-Acetylmuramate 6-phosphate/MurNAc 6-phosphate	C11H20NO11P
naneu	N-Acetylneuraminate	N-Acetylneuraminic acid/5-Acetamido-3,5-dideoxy-D-glycero-D-galacto-2-nonulosonic acid/Neu5Ac	C11H19NO9
naorn	N2-Acetyl-L-ornithine	N-Acetylornithine	C7H14N2O3
napth12d	Naphthalene-1,2-diol	1,2-Naphthalenediol/beta-Naphthohydroquinone/1,2-Dihydroxynaphthalene	C10H8O2
napthah	1-Naphthaldehyde	1-Formylnaphthalene	C11H8O
nfglu	N-Formimino-L-glutamate	N-Formimidoyl-L-glutamate	C6H10N2O4
nh4	Ammonium		NH4
no	Nitric oxide	NO/Nitrogen monoxide	NO
no2	Nitrite		HNO2
no3	Nitrate	Nitric acid	HNO3
npran	N-(5-Phospho-D-ribosyl)anthranilate	N-(5-Phospho-beta-D-ribosyl)anthranilate/N-(5-Phosphoribosyl)anthranilic acid	C12H16NO9P
ntphp	4-Nitrophenyl phosphate		C6H6NO6P
o2	Oxygen	O2	O2
o2s	Superoxide anion	O2-	O2
oaa	Oxaloacetate	Oxalacetic acid/Oxaloacetic acid/2-Oxobutanedioic acid/Oxosuccinic acid/keto-Oxaloacetate	C4H4O5
oadip	3-Oxoadipate	3-Oxoadipic acid/3-Keto-adipate	C6H8O5
oahser	O-Acetyl-L-homoserine		C6H11NO4
obut	2-Oxobutanoate	2-Ketobutyric acid/2-Oxobutyric acid/2-Oxobutyrate/2-Oxobutanoic acid/alpha-Ketobutyric acid/alpha-Ketobutyrate	C4H6O3
ogt	Oxidized glutathione	Glutathione disulfide/GSSG/Oxiglutatione	C20H32N6O12S2
ohpb	2-Oxo-3-hydroxy-4-phosphobutanoate	alpha-Keto-3-hydroxy-4-phosphobutyrate/(3R)-3-Hydroxy-2-oxo-4-phosphonooxybutanoate	C4H7O8P
oicap	3-Carboxy-4-methyl-2-oxopentanoate	(2S)-2-Isopropyl-3-oxosuccinate/2-Oxo-4-methyl-3-carboxypentanoate	C7H10O5
omcmc	2-Oxo-5-methyl-cis-muconate		C7H8O5
omp	Orotidine 5'-phosphate	Orotidylic ac	C10H13N2O11P
ooadpcoa	3-Oxoadipyl-CoA		C27H42N7O20P3S
op4en	2-Oxopent-4-enoate	2-Hydroxy-2,4-pentadienoate/cis-2-Hydroxypenta-2,4-dienoate/Oxopent-4-enoate/2-Hydroxypenta-2,4-dienoate	C5H6O3
opp	all-trans-Octaprenyl diphosphate	Farnesylfarnesylgeraniol	C40H68O7P2
orn	L-Ornithine	(S)-2,5-Diaminovaleric acid/(S)-2,5-Diaminopentanoic acid/(S)-2,5-Diaminopentanoate	C5H12N2O2
oroa	Orotate	Orotic acid/Uracil-6-carboxylic acid	C5H4N2O4
oslhser	O-Succinyl-L-homoserine		C8H13NO6
osuccbenz	2-Succinylbenzoate	o-Succinylbenzoate/Succinylbenzoate	C11H10O5
othio	Oxidized thioredoxin	Thioredoxin disulfide/Thioredoxin sulfide	C10H12N4O4S2R4
oxferdx	Oxidized ferredoxin		
oxglycolate	Oxaloglycolate	2-Hydroxy-3-oxosuccinate	C4H4O6
p5c	1-Pyrroline-5-carboxylate	(S)-1-Pyrroline-5-carboxylate/L-1-Pyrroline-5-carboxylate	C5H7NO2
pa	Phosphatidate	Phosphatidic acid/1,2-Diacyl-sn-glycerol 3-phosphate/3-sn-Phosphatidate	
paba	4-Aminobenzoate	ABEE/4-Aminobenzoic acid/p-Aminobenzoate	C7H7NO2
pac	Phenylacetic acid	Benzylformic acid/Phenylacetate/Benzeneacetiic acid	C8H8O2
pacald	Phenylacetaldehyde	alpha-Tolualdehyde	C8H8O
pant	(R)-Pantoate	Pantoate/Pantoic acid	C6H12O4
pantcys	N-((R)-Pantothenoyl)-L-cysteine	D-Pantothenoyl-L-cysteine/N-Pantothenoylcysteine	C12H22N2O6S
pap	Adenosine 3',5'-bisphosphate	PAP/3'-Phosphoadenylate/Phosphoadenosine phosphate	C10H15N5O10P2

paps	3'-Phosphoadenylyl sulfate	3'-Phosphoadenosine 5'-phosphosulfate/3'-Phospho-5'-adenylyl sulfate/PAPS	C10H15N5O13P2S
pbg	Porphobilinogen		C10H14N2O4
pc	phosphatidylcholine	Lecithin/Phosphatidyl-N-trimethylethanolamine/1,2-Diacyl-sn-glycero-3-phosphocholine/Choline phosphatide/3-sn-Phosphatidylcholine	C10H18NO8PR2
pc3a	Precorrin 3A		C43H50N4O16
pc3b	Precorrin 3B		C43H50N4O17
pc4	Precorrin 4		C44H52N4O17
pc5	Precorrin 5		C45H54N4O17
pc6a	Precorrin 6A	Precorrin 6X	C44H54N4O16
pc6b	Precorrin 6B	Precorrin 6Y	C44H56N4O16
pc8	Precorrin 8X	Precorrin 8	C45H60N4O14
pdla	Pyridoxamine	PM	C8H12N2O2
pdx5p	Pyridoxine 5'-phosphate	Pyridoxine phosphate/Pyridoxine 5-phosphate	C8H12NO6P
pe	Phosphatidylethanolamine	(3-Phosphatidyl)ethanolamine/(3-Phosphatidyl)-ethanolamine/Cephalin/O-(1-beta-Acyl-2-acyl-sn-glycero-3-phospho)ethanolamine/1-Acyl-2-acyl-sn-glycero-3-phosphoethanolamine	C7H12NO8PR2
peamn	Phenethylamine	2-Phenylethylamine/beta-Phenylethylamine/Phenylethylamine	C8H11N
pep	Phosphoenolpyruvate	Phosphoenolpyruvic acid/PEP	C3H5O6P
pept	Peptide		C2H4NO2R(C2H2NOR)n
PEPTIDO	peptidoglycan		
perll	Perillic acid		C10H14O2
perllald	Perillyl aldehyde	Perillaldehyde	C10H14O
pg	Phosphatidylglycerol	3-(3-sn-Phosphatidyl)glycerol/3(3-Phosphatidyl-)glycerol/PtdGro	C8H13O10PR2
pgp	Phosphatidylglycerophosphate	3(3-sn-Phosphatidyl)-sn-glycerol 1-phosphate/3(3-Phosphatidyl-)L-glycerol 1-phosphate/1,2-Diacyl-sn-glycero-3-phospho-sn-glycerol 3'-phosphate	C8H14O13P2R2
phaccoa	Phenylacetyl-CoA		C29H42N7O17P3S
phe	L-Phenylalanine	(S)-alpha-Amino-beta-phenylpropionic acid	C9H11NO2
pheacnit	Phenylacetonitrile	Benzyl cyanide	C8H7N
pheact	2-Phenylacetamide		C8H9NO
phen	Prephenate	Prephenic acid	C10H10O6
phetrna	L-Phenylalanyl-tRNA(Phe)		C19H26NO11PR2(C5H8O6PR)n
PHOSPHOLIPID	phospholipid		
phppi	Phytyl diphosphate		C20H42O7P2
phpyr	Phenylpyruvate	Phenylpyruvic acid/alpha-Ketohydrocinnamic acid/keto-Phenylpyruvate/3-Phenyl-2-oxopropanoate	C9H8O3
phser	O-Phospho-L-homoserine		C4H10NO6P
pht	O-Phospho-4-hydroxy-L-threonine	4-(Phosphonooxy)-threonine/4-(Phosphonooxy)-L-threonine	C4H10NO7P
phytate	Phytic acid	Phytate/myo-Inositol hexakisphosphate/1D-myo-Inositol 1,2,3,4,5,6-hexakisphosphate/D-myo-Inositol 1,2,3,4,5,6-hexakisphosphate/myo-Inositol 1,2,3,4,5,6-hexakisphosphate/Inositol 1,2,3,4,5,6-hexakisphosphate/1D-myo-Inositol hexakisphosphate	C6H18O24P6
pi	Phosphate	Orthophosphate/Phosphoric acid/Orthophosphoric acid	H3PO4
pl	Pyridoxal		C8H9NO3
pmcoa	Pimeloyl-CoA	6-Carboxyhexanoyl-CoA	C28H46N7O19P3S
pnp	4-Nitrophenol	p-Nitrophenol/PNP/Niphen/4-Hydroxynitrobenzene	C6H5NO3
pnpq	2-Phytyl-1,4-naphthoquinone	Demethylphylloquinone	C30H44O2
pnto	(R)-Pantothenate	Pantothenate/Pantothenic acid	C9H17NO5
ppa	Propionate	Propanoate/Propanoic acid/Propionic acid	C3H6O2
ppac	Phosphonoacetate	Phosphonoacetic acid/Fosfonet	C2H5O5P
ppacald	Phosphonoacetaldehyde	2-Phosphonoacetaldehyde/2-Oxoethylphosphonate	C2H5O4P
ppacp	propionyl-[acyl carrier protein]		C14H26N2O8PRS
ppadsel	3'-Phosphoadenylylselenate	3'-Phosphoadenosine-5'-phosphoselanate	C10H16N5O13P2Se
ppald	Propinol adenylate	Propionyladenylate	C13H18N5O8P
ppap	Propanoyl phosphate	Propionyl phosphate	C3H7O5P
ppcoa	Propanoyl-CoA	Propionyl-CoA/Propionyl coenzyme A	C24H40N7O17P3S
ppecoa	Propenoyl-CoA	Acryloyl-CoA/Acrylyl-CoA	C24H38N7O17P3S
ppeptido	peptidoglycan precursor		
ppgpp	Guanosine 3',5'-bis(diphosphate)	Guanosine 3'-diphosphate 5'-diphosphate/Guanosine 5'-diphosphate,3'-diphosphate	C10H17N5O17P4
pphg	Protoporphyrinogen IX		C34H40N4O4
ppi	Diphosphate	Pyrophosphate/Pyrophosphoric acid/PPi	P2H4O7
ppix	Protoporphyrin	Protoporphyrin IX/Porphyrinogen IX	C34H34N4O4

ppn	Propynoate	Propiolic acid/Acetylenecarboxylic acid/Acetylenemonocarboxylate	C3H2O2
pppgpp	Guanosine 3'-diphosphate 5'-triphosphate	Guanosine 5'-triphosphate,3'-diphosphate	C10H18N5O20P5
pppi	Inorganic triphosphate		P3H5O10
pppn	Phenylpropanoate	3-Phenyl-propionic acid/3-Phenylpropanoic acid	C9H10O2
pq	Phylloquinone	Vitamin K1/Phytonadione/2-Methyl-3-phytyl-1,4-naphthoquinone	C31H46O2
pqq	PQQ	Pyrrolo-quinoline quinone/Pyrroloquinoline-quinone/Pyrroloquinoline quinone/4,5-Dioxo-4,5-dihydro-1H-pyrrolo[2,3-f]quinoline-2,7,9-tricarboxylate	C14H6N2O8
pqqh2	PQQH2	Reduced pyrroloquinoline-quinone	C14H8N2O8
pram	5-Phosphoribosylamine	5-Phospho-beta-D-ribosylamine/5-Phospho-D-ribosylamine/5-Phosphoribosyl-1-amine	C5H12NO7P
prbamp	1-(5-Phosphoribosyl)-AMP	Phosphoribosyl-AMP/N1-(5-Phospho-D-ribosyl)-AMP	C15H23N5O14P2
prbatp	1-(5-Phosphoribosyl)-ATP	Phosphoribosyl-ATP/N1-(5-Phospho-D-ribosyl)-ATP	C15H25N5O20P4
prfp	1-(5-Phosphoribosyl)-5-[(5-phosphoribosylamino)methylideneamino]imidazole-4-carboxamide	-(5-Phospho-D-ribosylaminoformimino)-1-(5-phosphoribosyl)-imidazole-4-carboxamide/N-(5'-Phospho-D-ribosylformimino)-5-amino-1-(5''-phospho-D-ribosyl)-4-imidazolecarboxamide/N-(5'-Phosphoribosylformimino)-5-amino-1-(5''-phosphoribosyl)-4-imidazolecarboxamide Phosphoribosyl-formimino-AICAR-phosphate	C15H25N5O15P2
prlp	5-[(5-Phospho-1-deoxyribulos-1-ylamino)methylideneamino]-1-(5-phosphoribosyl)imidazole-4-carboxamide	N-(5'-Phospho-D-1'-ribulosylformimino)-5-amino-1-(5''-phospho-D-ribosyl)-4-imidazolecarboxamide/Phosphoribulosyl-formimino-AICAR-phosphate	C15H25N5O15P2
pro	L-Proline	2-Pyrrolidinecarboxylic acid	C5H9NO2
progly	L-Prolinylglycine		C7H12N2O3
propanal	Propanal	Propionaldehyde	C3H6O
propen	Propenoate	Acrylic acid/Acrylate/2-Propenoic acid/Vinylformic acid	C3H4O2
PROTEIN	protein		
protmn	Protoanemonin	4-Methylenebut-2-en-4-olide/cis-4-Methylenebut-2-en-4-olide	C5H4O2
protrna	L-Prolyl-tRNA(Pro)		C15H24NO11PR2(C5H8O6PR)n
prpp	5-Phospho-alpha-D-ribose 1-diphosphate	5-Phosphoribosyl diphosphate/5-Phosphoribosyl 1-pyrophosphate/PRPP	C5H13O14P3
ps	Phosphatidylserine	Phosphatidyl-L-serine/1,2-Diacyl-sn-glycerol 3-phospho-L-serine/3-O-sn-Phosphatidyl-L-serine/O3-Phosphatidyl-L-serine	C8H12NO10PR2
pser	O-Phospho-L-serine	L-O-Phosphoserine/3-Phosphoserine	C3H8NO6P
pth	Protoheme	Heme/Haem/Heme B/Protoheme IX	C34H32FeN4O4
ptrc	Putrescine	1,4-Butanediamine/1,4-Diaminobutane/Tetramethylenediamine	C4H12N2
ptt	Pantetheine	(R)-Pantetheine	C11H22N2O4S
pyam5p	Pyridoxamine 5'-phosphate	Pyridoxamine phosphate/Pyridoxamine 5-phosphate	C8H13N2O5P
pydx5p	Pyridoxal 5'-phosphate	Pyridoxal 5-phosphate/Pyridoxal phosphate	C8H10NO6P
pydxn	Pyridoxine	Pyridoxol	C8H11NO3
pyr	Pyruvate	Pyruvic acid/2-Oxopropanoate/2-Oxopropanoic acid/Pyroracemic acid	C3H4O3
pythp	6-Pyruvoyltetrahydropterin	6-(1,2-Dioxopropyl)-5,6,7,8-tetrahydropterin/6-Pyruvoyl-5,6,7,8-tetrahydropterin	C9H11N5O3
qa	Quinolinate	Pyridine-2,3-dicarboxylate/Quinolinic acid/2,3-Pyridinedicarboxylic acid	C7H5NO4
qt	Quinate	Quinic acid/Kinic acid/Chinic acid/L-Quinic acid/L-Quinate/(-)-Quinic acid	C7H12O6
r15bp	D-Ribose 1,5-bisphosphate	Ribose 1,5-bisphosphate	C5H12O11P2
r1p	alpha-D-Ribose 1-phosphate	Ribose 1-phosphate/D-Ribose 1-phosphate	C5H11O8P
r2mm	(R)-2-Methylmalate	(R)-2-Methylmalic acid/(3R)-Citramalate/(3R)-Citramalic acid/(3R)-alpha-Hydroxypyrotartaric acid/D-Citramalate/D-Citramalic acid/D-alpha-Hydroxypyrotartaric acid/(2R)-2-Hydroxy-2-methylbutanedioate	C5H8O5
r3hbcoa	(R)-3-Hydroxybutanoyl-CoA	(3R)-3-Hydroxybutanoyl-CoA	C25H42N7O18P3S
r3hbn	(R)-3-Hydroxybutanoate	(R)-3-Hydroxybutanoic acid/(R)-3-Hydroxybutyric acid	C4H8O3
r5p	alpha-D-Ribose 5-phosphate		C5H11O8P
raffinose	Raffinose	Melitose/Melitriose/Gossypose/6G-alpha-D-galactosylsucrose	C18H32O16
rbflvrd	Reduced riboflavin		C17H22N4O6
rdferdx	Reduced ferredoxin		

rdmbzi	N1-(alpha-D-ribosyl)-5,6-dimethylbenzimidazole	alpha-Ribazole	C14H18N2O4
rgt	Reduced glutathione	Glutathione/5-L-Glutamyl-L-cysteinylglycine/N-(N-gamma-L-Glutamyl-L-cysteinyl)glycine/gamma-L-Glutamyl-L-cysteinyl-glycine/GSH	C10H17N3O6S
rib	D-Ribose		C5H10O5
ribflav	Riboflavin	Lactoflavin/7,8-Dimethyl-10-ribitylisoalloxazine/Vitamin B2	C17H20N4O6
rl5p	D-Ribulose 5-phosphate		C5H11O8P
rml	L-Rhamnulose		C6H12O5
rml1p	L-Rhamnulose 1-phosphate		C6H13O8P
rmn	L-Rhamnose	6-Deoxy-L-mannose/L-Mannomethylose	C6H12O5
rmnn	L-Rhamnonate	6-Deoxy-L-mannonic acid	C6H12O6
RNA	RNA		
rthio	Reduced thioredoxin	Thioredoxin	C10H14N4O4S2R4
s	Sulfur	S/Sulfur, precipitated	S
s3h2mbcoa	(S)-3-Hydroxy-2-methylbutyryl-CoA	(2S,3S)-3-Hydroxy-2-methylbutanoyl-CoA	C26H44N7O18P3S
s7p	Sedoheptulose 7-phosphate	altro-Heptulose 7-phosphate	C7H15O10P
sah	S-Adenosyl-L-homocysteine	S-Adenosylhomocysteine	C14H20N6O5S
saicar	1-(5'-Phosphoribosyl)-5-amino-4-(N-succinocarboxamide)-imidazole	1-(5'-Phosphoribosyl)-4-(N-succinocarboxamide)-5-aminoimidazole/5'-Phosphoribosyl-4-(N-succinocarboxamide)-5-aminoimidazole/(S)-2-[5-Amino-1-(5-phospho-D-ribosyl)imidazole-4-carboxamido]succinate/SAICAR	C13H19N4O12P
salc6p	Salicin 6-phosphate	Salicin-6P	C13H19O10P
salchol	Salicyl alcohol	Saligenin/2-Hydroxybenzyl alcohol/2-(Hydroxymethyl)phenol	C7H8O2
salcyl	Salicylate	o-Hydroxybenzoic acid/Salicylic acid	C7H6O3
sam	S-Adenosyl-L-methionine	S-Adenosylmethionine/Acylcarnitine	C15H23N6O5S
sama	S-Adenosylmethioninamine	(5-Deoxy-5-adenosyl)(3-aminopropyl)methylsulfonium salt	C14H23N6O3S
samob	S-Adenosyl-4-methylthio-2-oxobutanoate		C15H20N5O6S
sap	S-Aminomethyldihydrolipoylprotein	[Protein]-S8-aminomethyldihydrolipoyllysine/H-Protein-S-aminomethyldihydrolipoyllysine	C9H19N2OS2R
sb1p	Sorbose 1-phosphate	L-Sorbose 1P/L-xylo-Hexulose 1-phosphate/L-Sorbose 1-phosphate	C6H13O9P
sbt6p	D-Sorbitol 6-phosphate	D-Sorbitol 6-phosphate	C6H15O9P
sbzcoa	O-Succinylbenzoyl-CoA	2-Succinylbenzoyl-CoA/Succinylbenzoyl-CoA	C32H44N7O20P3S
scinos	scyllo-Inosose	2,4,6/3,5-Pentahydroxycyclohexanone/2-Inosose	C6H10O6
scys	Selenocysteine	L-Selenocysteine	C3H7NO2Se
seadseh	Se-Adenosylselenohomocysteine		C14H20N6O5Se
seasmet	Se-Adenosylselenomethionine		C15H24N6O5Se
sectrna	L-Seryl-tRNA(Sec)		C13H22NO12PR2(C5H8O6PR)n
seld	Selenide	Hydrogen selenide	H2Se
selmtrna	Selenomethionyl-tRNA(Met)		C20H30N6O11PSeR(C5H8O6PR)n
selncystrna	L-Selenocysteinyl-tRNA(Sec)		C13H22NO11PSeR2(C5H8O6PR)n
selngluth	Selenodiglutathione	GSSeSG	C20H32N6O12S2Se
selnp	Selenophosphate	Selenophosphoric acid	PH3SeO3
selnt	Selenate	Selenic acid	H2SeO4
selt	Selenite		SeO3
ser	L-Serine	L-2-Amino-3-hydroxypropionic acid/L-3-Hydroxy-alanine	C3H7NO3
sertrna	L-Seryl-tRNA(Ser)		C13H22NO12PR2(C5H8O6PR)n
sgdhl	S-Glutaryldihydrolipoamide		C13H23NO4S2
shchc	(1R,6R)-6-Hydroxy-2-succinylcyclohexa-2,4-diene-1-carboxylate	(1R,6R)-2-Succinyl-6-hydroxy-2,4-cyclohexadiene-1-carboxylate/SHCHC	C11H12O6
shcl	Sirohydrochlorin		C42H46N4O16
shcys	Selenohomocysteine		C4H9NO2Se
sheme	Siroheme		C42H44FeN4O16
shser	O-Succinylhomoserine		C8H13NO6
skm5p	Shikimate 5-phosphate	Shikimate 3-phosphate	C7H11O8P
sl26da	N-Succinyl-LL-2,6-diaminoheptanedioate	N-Succinyl-LL-2,6-diaminopimelate/N-Succinyl-L-2,6-diaminoheptanedioate/N-Succinyl-L-2,6-diaminopimelate	C11H18N2O7
sl2a6o	N-Succinyl-2-L-amino-6-oxoheptanedioate	N-Succinyl-L-2-amino-6-oxoheptanedioate/N-Succinyl-L-2-amino-6-oxopimelate/N-Succinyl-2-amino-6-oxo-L-pimelic acid/N-Succinyl-epsilon-keto-L-aminopimelic acid/(S)-2-(Succinylamino)-6-oxoheptanedioate	C11H15NO8
sllct	Selenocystathionine		C7H14N2O4Se
sme	Shikimate	Shikimic acid/3,4,5-Trihydroxy-1-cyclohexenecarboxylic acid	C7H10O5
smet	Selenomethionine		C5H11NO2Se
so3	Sulfite		H2SO3

so4	Sulfate	Sulfuric acid	H2SO4
sot	D-Sorbitol	D-Glucitol/L-Gulitol/Sorbitol	C6H14O6
sprm	Spermine	N,N'-Bis(3-aminopropyl)-1,4-butanediamine	C10H26N4
sprmd	Spermidine	N-(3-Aminopropyl)-1,4-butane-diamine	C7H19N3
srlh	S-Ribosyl-L-homocysteine	S-D-Ribosyl-L-homocysteine/Ribose-5-S-homocysteine/S-Ribosylhomocysteine/S-(5-Deoxy-D-ribos-5-yl)-L-homocysteine	C9H17NO6S
sslcys	S-Sulfo-L-cysteine		C3H7NO5S2
stachyose	Stachyose		C24H42O21
starch	starch		(C12H20O10)n
suc	sucrose	Cane sugar/Saccharose/1-alpha-D-Glucopyranosyl-2-beta-D-fructofuranoside	C12H22O11
suc6p	Sucrose 6-phosphate	6-Phosphosucrose/6-O-Phosphonosucrose/beta-D-Fructofuranosyl-6-O-phosphono-alpha-D-glucopyranoside	C12H23O14P
succ	Succinate	Succinic acid/Butanedionic acid/Ethylenesuccinic acid	C4H6O4
succarg	N2-Succinyl-L-arginine	(2S)-2-(3-Carboxypropanoylamino)-5-(diaminomethylideneamino)pentanoic acid	C10H18N4O5
succglu	N-Succinyl-L-glutamate	(2S)-2-(3-Carboxypropanoylamino)pentanedioic acid	C9H13NO7
succglusa	N-Succinyl-L-glutamate 5-semialdehyde	(2S)-2-(3-Carboxypropanoylamino)-5-oxopentanoic acid	C9H13NO6
succoa	Succinyl-CoA	Succinyl coenzyme A	C25H40N7O19P3S
succorn	N2-Succinyl-L-ornithine	(2S)-5-Amino-2-(3-carboxypropanoylamino)pentanoic acid	C9H16N2O5
sucephc	2-Succinyl-5-enolpyruvyl-6-hydroxy-3-cyclohexene-1-carboxylate	5-Enolpyruvoyl-6-hydroxy-2-succinylcyclohex-3-ene-1-carboxylate	C14H16O9
sucsal	Succinic semialdehyde	Succinate semialdehyde	C4H6O3
sulald	Sulfoacetaldehyde	2-Sulfoacetaldehyde	C2H4O4S
t16p	D-Tagatose 1,6-bisphosphate		C6H14O12P2
t2mipdcoa	trans-2-Methyl-5-isopropylhexa-2,5-dienoyl-CoA		C31H50N7O17P3S
t3	D-Glyceraldehyde		C3H6O3
t3chc	trans-3-Chloroacrylic acid		C3H3ClO2
t3chp	trans-3-Chloro-2-propene-1-ol	trans-3-Chloroallyl alcohol	C3H5ClO
t6p	D-Tagatose 6-phosphate		C6H13O9P
tagatn	D-Tagaturonate	D-Tagaturonic acid	C6H10O7
tartr	L-Tartaric acid	(R,R)-Tartaric acid/(R,R)-Tartrate/Tartaric acid/Tartrate/2,3-Dihydroxybutanedioic acid/(2R,3R)-Tartaric acid/(+)-Tartaric acid	C4H6O6
taur	Taurine	2-Aminoethanesulfonic acid/Aminoethylsulfonic acid	C2H7NO3S
tcmbo	trans-4-Carboxymethylenebut-2-en-4-olide		C6H4O4
tcynt	Thiocyanate	Thiocyanic acid	CHNS
tcys	Thiocysteine		C3H7NO2S2
tdhdp	2,3,4,5-Tetrahydrodipicolinate	delta1-Piperidine-2,6-dicarboxylate/L-2,3,4,5-Tetrahydrodipicolinate/(S)-2,3,4,5-Tetrahydropyridine-2,6-dicarboxylate	C7H9NO4
tglu	Tetrahydropteroyltri-L-glutamate		C29H37N9O12
thcych12d	3D-(3,5/4)-Trihydroxycyclohexane-1,2-dione	D-2,3-Diketo-4-deoxy-epi-inositol/(3R,4S,5R)-3,4,5-Trihydroxy-1,2-cyclohexanedione/3,5/4-Trihydroxycyclohexa-1,2-dione	C6H8O5
thf	5,6,7,8-Tetrahydrofolate	Tetrahydrofolate/Tetrahydrofolic acid/THF/(6S)-Tetrahydrofolate/(6S)-Tetrahydrofolic acid/(6S)-THFA	C19H23N7O6
thfglu	THF-L-glutamate	Tetrahydrofolyl-[Glu](2)	C24H30N8O9
thiamin	Thiamin	Thiamine/Vitamin B1/Aneurin/Antiberiberi factor	C12H17N4OS
thmp	Thiamin monophosphate	Thiamine monophosphate/Thiamin phosphate/Thiamine phosphate/TMP	C12H18N4O4PS
thmpp	Thiamine diphosphate	Thiamine diphosphate/Thiamin pyrophosphate/TPP/ThPP	C12H19N4O7P2S
thr	L-Threonine	2-Amino-3-hydroxybutyric acid	C4H9NO3
thr3masp	L-threo-3-Methylaspartate		C5H9NO4
thro3p	L-Threonine O-3-phosphate	L-Threonine phosphate	C4H10NO6P
thrtrna	L-Threonyl-tRNA(Thr)		C14H24NO12PR2(C5H8O6PR)n
thym	Thymine	5-Methyluracil	C5H6N2O2
thymd	Thymidine	Deoxythymidine	C10H14N2O5
thzp	4-Methyl-5-(2-phosphoethyl)-thiazole	4-Methyl-5-(2-phosphono-oxyethyl)-thiazole	C6H10NO4PS
tma	Trimethylamine	(CH3)3N/N,N-Dimethylmethanamine	C3H9N
tmao	Trimethylamine N-oxide	(CH3)3NO	C3H9NO
tnittol	Trinitrotoluene	2,4,6-Trinitrotoluene	C7H5N3O6
tre	Trehalose	alpha,alpha-Trehalose/alpha,alpha'-Trehalose	C12H22O11
tre6p	alpha,alpha'-Trehalose 6-phosphate	Trehalose 6-phosphate	C12H23O14P
trnaala	tRNA(Ala)		C10H17O10PR2(C5H8O6PR)n

trnaarg	tRNA(Arg)		C15H21N5O10PR(C5H8O6PR)n
trnaasn	tRNA(Asn)		0
trnaasp	tRNA(Asp)		C10H17O10PR2(C5H8O6PR)n
trnacys	tRNA(Cys)		C15H21N5O10PR(C5H8O6PR)n
trnagln	tRNA(Gln)		C15H21N5O10PR(C5H8O6PR)n
trnaglu	tRNA(Glu)		C15H21N5O10PR(C5H8O6PR)n
trnagly	tRNA(Gly)		C10H17O10PR2(C5H8O6PR)n
trnahis	tRNA(His)		C10H17O10PR2(C5H8O6PR)n
trnaile	tRNA(Ile)		C15H21N5O10PR(C5H8O6PR)n
trnaleu	tRNA(Leu)		C15H21N5O10PR(C5H8O6PR)n
trnalys	tRNA(Lys)		C10H17O10PR2(C5H8O6PR)n
trnamet	tRNA(Met)		C15H21N5O10PR(C5H8O6PR)n
trnaphe	tRNA(Phe)		C10H17O10PR2(C5H8O6PR)n
trnapro	tRNA(Pro)		C10H17O10PR2(C5H8O6PR)n
trnasec	tRNA(Sec)		
trnaser	tRNA(Ser)		C10H17O10PR2(C5H8O6PR)n
trnathr	tRNA(Thr)		C10H17O10PR2(C5H8O6PR)n
trnatrp	tRNA(Trp)		C15H21N5O10PR(C5H8O6PR)n
trnatyr	tRNA(Tyr)		C15H21N5O10PR(C5H8O6PR)n
trnaval	tRNA(Val)		C15H21N5O10PR(C5H8O6PR)n
trp	L-Tryptophan	Tryptophan/(S)-alpha-Amino-beta-(3-indolyl)-propionic acid	C11H12N2O2
trptrna	L-Tryptophanyl-tRNA(Trp)		C26H31N7O11PR(C5H8O6PR)n
tsul	Thiosulfate	Hyposulfite	HS2O3
tym	Tyramine	2-(p-Hydroxyphenyl)ethylamine	C8H11NO
tyr	L-Tyrosine	(S)-3-(p-Hydroxyphenyl)alanine/(S)-2-Amino-3-(p-hydroxyphenyl)propionic acid	C9H11NO3
tyrtrna	L-Tyrosyl-tRNA(Tyr)		C24H30N6O12PR(C5H8O6PR)n
u1car	Urea-1-carboxylate	Allophanate/Allophanic acid	C2H4N2O3
u3hga	UDP-3-O-(3-hydroxytetradecanoyl)-D-glucosamine	UDP-3-O-(beta-hydroxymyristoyl)-D-glucosamine	C29H51N3O18P2
uaagmda	Undecaprenyl-diphospho-N-acetylmuramoyl-(N-acetylglucosamine)-L-alanyl-D-glutamyl-meso-2,6-diaminopimeloyl-D-alanyl-D-alanine		C95H156N8O28P2
uaagmm5da	Undecaprenyl-diphospho-N-acetylmuramoyl-(N-acetylglucosamine)-L-alanyl-D-glutaminyl-meso-2,6-diaminopimeloyl-(glycyl)5-D-alanyl-D-alanine		C105H172N14O32P2
uaagmmda	Undecaprenyl-diphospho-N-acetylmuramoyl-(N-acetylglucosamine)-L-alanyl-D-glutaminyl-meso-2,6-diaminopimeloyl-D-alanyl-D-alanine		C95H157N9O27P2
uaccg	UDP-N-acetyl-3-(1-carboxyvinyl)-D-glucosamine	UDP-N-acetyl-3-O-(1-carboxyvinyl)-D-glucosamine/UDP-N-acetylglucosamine-3-O-pyruvateether/UDP-N-acetylglucosamine enolpyruvate	C20H29N3O19P2
uagmda	Undecaprenyl-diphospho-N-acetylmuramoyl-L-alanyl-D-glutamyl-meso-2,6-diaminopimeloyl-D-alanyl-D-alanine		C87H143N7O23P2
uama	UDP-N-acetylmuramoyl-L-alanine		C23H36N4O20P2
uamag	UDP-N-acetylmuramoyl-L-alanyl-D-glutamate		C28H43N5O23P2
udcpara4fn	Undecaprenyl phosphate alpha-L-Ara4FN		C61H100NO8P

udcpdp	Undecaprenyl diphosphate	di-trans,poly-cis-Undecaprenyl diphosphate/Bactoprenyl diphosphate/ditrans,octacis-Undecaprenyl diphosphate	C55H92O7P2
udcpp	Undecaprenyl phosphate	di-trans,poly-cis-Undecaprenyl phosphate/ditrans,octacis-Undecaprenyl phosphate	C55H91O4P
udp	UDP	Uridine 5'-diphosphate	C9H14N2O12P2
udpacgal	UDP-N-acetyl-D-galactosamine		C17H27N3O17P2
udpara4fn	UDP-L-Ara4FN	Uridine 5'-diphospho-beta-(4-deoxy-4-formamido-L-arabinose)/UDP-4-deoxy-4-formamido-beta-L-arabinopyranose	C15H23N3O16P2
udpara4n	UDP-L-Ara4N	UDP-4-amino-4-deoxy-L-arabinose/UDP-4-amino-4-deoxy-beta-L-arabinopyranose	C14H23N3O15P2
udpara4o	UDP-L-Ara4O	UDP-4''-ketopentose/Uridine 5'-diphospho-beta-(L-threo-pentapyranosyl-4''-ulose)/UDP-beta-L-threo-pentapyranos-4-ulose	C14H20N2O16P2
udpg	UDP-glucose	UDPglucose/UDP-D-glucose/Uridine diphosphate glucose/UDP-alpha-D-glucose	C15H24N2O17P2
udpg23a	UDP-2,3-bis(3-hydroxytetradecanoyl)glucosamine	UDP-2,3-bis(beta-hydroxymyristoyl)-D-glucosamine/UDP-2,3-bis(3-hydroxytetradecanoyl)-D-glucosamine	C43H77N3O20P2
udpg2aa	UDP-3-O-(3-hydroxytetradecanoyl)-N-acetylglucosamine	UDP-3-O-(beta-hydroxymyristoyl)-N-acetylglucosamine	C31H53N3O19P2
udpgal	UDP-D-galactose	UDP-galactose/UDP-D-galactopyranose	C15H24N2O17P2
udpgalfur	UDP-alpha-D-galacto-1,4-furanose	UDP-D-galacto-1,4-furanose	C15H24N2O17P2
udpglcur	UDP-D-glucuronate	UDP-glucuronate/UDPglucuronate/UDP-alpha-D-glucuronate	C15H22N2O18P2
udpnadma	UDP-N-acetyl-D-mannosamine		C17H27N3O17P2
udpnadmarn	UDP-N-acetyl-D-mannosaminouronate	UDP-N-acetyl-2-amino-2-deoxy-D-mannuronate/UDP-N-acetyl-D-mannosaminuronic acid	C17H25N3O18P2
udpnag	UDP-N-acetyl-D-glucosamine	UDP-N-acetylglucosamine	C17H27N3O17P2
udpnam	UDP-N-acetylmuramate	UDP-N-acetylmuramic acid/UDP-MurNAc	C20H31N3O19P2
ugmd	UDP-N-acetylmuramoyl-L-alanyl-D-gamma-glutamyl-meso-2,6-diaminopimelate	UDP-N-acetylmuramoyl-L-alanyl-D-gamma-glutamyl-meso-2,6-diamino-heptanedioate	C35H55N7O26P2
ugmda	UDP-N-acetylmuramoyl-L-alanyl-D-glutamyl-6-carboxy-L-lysyl-D-alanyl-D-alanine	UDP-N-acetylmuramoyl-L-alanyl-D-glutamyl-meso-2,6-diaminopimeloyl-D-alanyl-D-alanine	C41H65N9O28P2
ump	UMP	Uridylic acid/Uridine monophosphate/Uridine 5'-monophosphate/5'Uridylic acid	C9H13N2O9P
uppg1	Uroporphyrinogen I		C40H44N4O16
uppg3	Uroporphyrinogen III		C40H44N4O16
uq	Ubiquinone	Coenzyme Q/CoQ/Q	C14H18O4(C5H8)n
uqh2	Ubiquinol	QH2/CoQH2	C14H20O4(C5H8)n
ura	Uracil		C4H4N2O2
urdglyc	(-)-Ureidoglycolate	(S)-Ureidoglycolate	C3H6N2O4
urea	Urea	Carbamide	CH4N2O
ureidogly	(S)-Ureidoglycine		C3H7N3O3
uri	Uridine		C9H12N2O6
urocan	Urocanate	Urocanic acid	C6H6N2O2
utp	UTP	Uridine 5'-triphosphate/Uridine triphosphate	C9H15N2O15P3
val	L-Valine	2-Amino-3-methylbutyric acid	C5H11NO2
valtrna	L-Valyl-tRNA(Val)		C20H30N6O11PR(C5H8O6PR)n
vanillate	Vanillate	Vanillic acid/4-Hydroxy-3-methoxybenzoate/4-Hydroxy-3-methoxybenzoic acid	C8H8O4
xan	Xanthine		C5H4N4O2
xmp	Xanthosine 5'-phosphate	Xanthylic acid/XMP/(9-D-Ribosylxanthine)-5'-phosphate	C10H13N4O9P
xtp	XTP		C10H15N4O15P3
xtsine	Xanthosine		C10H12N4O6
xu5p	D-Xylulose 5-phosphate		C5H11O8P
xyl	D-Xylose	Wood sugar	C5H10O5
xylu	D-Xylulose	D-threo-Pentulose/D-Lyxulose	C5H10O5
zoe	(Z)-5-Oxohex-2-enedioate	gamma-Oxalocrotonate/4-Oxalocrotonate	C6H6O5
