# Supplementary material for: Genome-scale reconstruction and in silico analysis of Klebsiella oxytoca for 2,3-butanediol production
Source: Microb Cell Fact. 2013 Feb 23;12:20. doi: 10.1186/1475-2859-12-20 (PMC3602198; doi:10.1186/1475-2859-12-20)
Supplement: Additional file 3 — Biomass composition of Klebsiella oxytoca. [file 1475-2859-12-20-S3.pdf]

Additional file 3. Biomass composition of *Klebsiella oxytoca*.

Table 3-1. Macromolecular composition[a]

Component	Composition (g/g DCW)	Comments and References
**Protein**	0.521	[1]
**DNA**	0.023	[1]
**RNA**	0.131	[1]
**Phospholipid**	0.073	[2]
**Cofactors and vitamins**	0.030	Assumption (Small molecules compose less than 3% of cell dry weight) [3]
**Cell wall**	0.222	
Lipopolysaccharide	0.034	[4]
Carbohydrate	0.153	Determined by this study and Liao's study [1]
Peptidoglycan	0.035	Carbohydrates made about 15.3 % of the cell wall in this study. The rest was assumed to be peptidoglycan.

[a]Calculated for an average macromolecular composition of *K. oxytoca* in minimal medium with D-glucose. Biomass composition was experimentally measured at the exponential growth phase of batch cultivation, which is the average of three samples. MW of 1 water was subtracted from MW of each molecule to account for esterification or peptide bonding.

Table 3-2. Protein composition

The amino acid composition was analyzed by Hewlett Packard 1100 series HPLC systems equipped with Waters Nova-Pak C18 4 um column (3.9 x 300 mm) (Korea Basic Science Institute, Daejeon, Korea).

Amino acids	mmol/g protein
Alanine	1.133
Arginine	0.493
Asparagine	0.410
Aspartate	0.410
Cysteine	0.096
Glutamate	0.499
Glutamine	0.499
Glycine	1.041
Histidine	0.190
Isoleucine	0.436
Leucine	0.768
lysine	0.448
Methionine	0.238
Phenylalanine	0.289
Proline	0.420
Serine	0.534
Threonine	0.583
Tryptophane	0.014
Tyrosine	0.259
Valine	0.666

Table 3-3. DNA composition

The DNA composition was determined from the genomic sequence of *K. oxytoca*. GC content of *K. oxytoca* is 56.05 %.

Nucleotide	mol/mol, DNA	MW, g/mol	mmol/g DNA
dAMP	0.220	313.200	0.711
dCMP	0.280	289.200	0.907
dTMP	0.220	304.200	0.711
dGMP	0.280	329.200	0.907

Table 3-4. RNA composition

It was assumed that RNA consists of 5% mRNA, 80% rRNA, and 15% tRNA [5].

Nucleotide	mol/mol RNA			MW, g/mol	mol/mol RNA	mmol/g RNA
	mRNA	rRNA	tRNA			
	0.050	0.800	0.150			
AMP	0.220	0.204	0.223	329.200	0.207	0.648
GMP	0.280	0.225	0.279	345.200	0.236	0.737
CMP	0.280	0.314	0.320	305.200	0.313	0.980
UMP	0.220	0.257	0.179	306.200	0.244	0.762

Table 3-5. Phospholipids composition

The composition of phospholipids was taken from Wassef's study [2].

Component	g/g phospholipids	mmol/g phospholipids
Phosphatidylethanolamine	0.820	1.186
Phosphatidylglycerol	0.045	0.062
Phosphatidyl serine	0.020	0.027
Phosphatidic acid	0.050	0.077
Diphosphatidylglycerol (Cardiolipin)	0.065	0.048

Table 5.1 Molecular weights of phospholipids components

Constituent	MW, g/mol		
	backbone	# of fatty acids residues	total
Phosphatidylethanolamine	181.128	2	691.63
Phosphatidylglycerol	212.139	2	722.64
Phosphatidylserine	225.138	2	735.64
Phosphatidic acid	138.06	2	648.56
Cardiolipin	332.183	4	1353.18

Table 3-6. Composition of fatty acids in phospholipids

The fatty acid composition was determined by Sherlock microbial identification system of Sherlock version 6.1 (Korea Research Institute of Bioscience and Biotechnology, Daejeon, Korea).

Fatty acid	g/g total fatty acids	MW, g/mol	mmol/g total fatty acids	mol/mol total fatty acids
c12	0.047	200.318	0.234	0.060
c13	0.002	214.344	0.011	0.003
c14:1	0.000	226.355	0.000	0.000
c14	0.129	228.371	0.564	0.144
c15:1	0.005	240.382	0.019	0.005
c15	0.000	242.398	0.000	0.000
c16:1	0.061	254.408	0.240	0.061
c16	0.380	256.424	1.482	0.378
c17:1	0.001	268.435	0.004	0.001
c17	0.271	270.451	1.001	0.256
c18:1	0.059	282.461	0.208	0.053
c18	0.006	284.477	0.021	0.005
c19	0.039	298.504	0.131	0.034

Table 3-7. Cofactors and vitamins incorporated in the biomass

Cofactors and vitamins are assumed to be same ratio (w/w)

Molecule	MW, g/mol	g/g cofactors and vitamins	mmol/g cofactors and small molecules
Pyridoxine	169.178	0.111	0.656
Coenzyme A	767.535	0.111	0.145
Flavin adenine dinucleotide	785.550	0.111	0.141
Flavin mononucleotide	456.344	0.111	0.243
Ubiquinone	794.623	0.111	0.140
NAD	664.433	0.111	0.167
NADP	744.413	0.111	0.149
Tetrahydrofolate	445.430	0.111	0.249
Thiamin	265.356	0.111	0.418

Table 3-8. Carbohydrate composition

The carbohydrates composition was analyzed by ICD-5000 (Dionex, Sunnyvale, CA, USA) equipped with CarboPac PA10 (4.5 x 250 mm, Dionex) and CarboPac PA10 cartridge (4 x 50 mm) (Korea Basic Science Institute, Daejeon, Korea).

Component	Molar ratio	MW, g/mol	mmol/g carbohydrate
N-acetylglucosamine	5.000	203.194	4.244
Galactose	1.000	162.156	0.849

Table 3-9. Lipopolysaccharide composition

The composition of lipopolysaccharide was assumed to be the same as in *Escherichia coli* [4].

Component	Molar ratio	MW, g/mol	mmol/g LPS
KDO(2)-lipid A	1.000	1624.910	0.140
ADP-L-glycero-D-manno-heptose	3.000	619.370	0.420
UDPglucose	2.000	566.050	0.280
CDP-Ethanolamine	2.000	446.06	0.280
CMP-2-keto-3-deoxyoctanoate	3.000	543.109	0.420

References

1. Yu-Chieh Liao, Tzu-Wen Huang, Feng-Chi Chen, Pep Charusanti, Jay S. J. Hong, Hwan-You Chang, Shih-Feng Tsai, Bernhard O. Palsson and Chao A. Hsiung **An Experimentally Validated Genome-Scale Metabolic Reconstruction of Klebsiella pneumoniae MGH 78578, iYL1228.** J. Bacteriol. 2011, **193(7):**1710-1717.

2. Wassef M.K.: **Lipids of Klebsiella pneumoniae: the presence of phosphatidyl choline in succinate-grown cells** Lipids. 1976, **11(5):**364-369.

3. Ingraham JL, Maalee O, Neidhardt FC: *Growth of the Bacterial Cell*. Sunderland: Sinauer Associates; 1983.

4. Neidhardt FC, Curtiss R, Ingraham JL, Lin ECC, Low KB, Magasanik B, Reznikoff WS, Riley M, Schaechter M, Umbarger HE: *Escherichia coli and Salmonella* , Washington D.C.: ASM press; 1996.

5. Brown TA: *Genomes 2nd* . New York: Wiley-Liss; 2002.
